# CenH3 evolution reflects meiotic symmetry as predicted by the centromere drive model

**DOI:** 10.1038/srep33308

**Published:** 2016-09-15

**Authors:** František Zedek, Petr Bureš

**Affiliations:** 1 Department of Botany and Zoology, Masaryk University, Kotlarska 2, 611 37 Brno, Czech Republic

## Abstract

The centromere drive model explaining rapid evolution of eukaryotic centromeres predicts higher frequency of positive selection acting on centromeric histone H3 (CenH3) in clades with asymmetric meiosis compared to the clades with only symmetric meiosis. However, despite the impression one might get from the literature, this key prediction of the centromere drive model has not only never been confirmed, but it has never been tested, because all the previous studies dealt only with the presence or absence instead of the frequency of positive selection. To provide evidence for or against different frequencies of positively selected CenH3 in asymmetrics and symmetrics, we have inferred the selective pressures acting on CenH3 in seventeen eukaryotic clades, including plants, animals, fungi, ciliates and apicomplexa, using codon-substitution models, and compared the inferred frequencies between asymmetrics and symmetrics in a quantitative manner. We have found that CenH3 has been evolving adaptively much more frequently in clades with asymmetric meiosis compared with clades displaying only symmetric meiosis which confirms the prediction of centromere drive model. Our findings indicate that the evolution of asymmetric meiosis required CenH3 to evolve adaptively more often to counterbalance the negative consequences of centromere drive.

Centromeric histone H3 (CenH3) is the cornerstone of the kinetochore, which ensures the proper segregation of chromosomes during cell division by mediating chromosomal attachment to spindle microtubules. This conserved function implies that the kinetochore should be evolving under strong purifying selection (i.e., it should be maintained as is) over evolutionary time, but multiple reports have suggested that CenH3 has been evolving adaptively (i.e., changing under positive selection) in various lineages of plants and animals^1^,^2^,^3^,^4^,^5^,^6^,^7^,^8^. This surprising paradox has been attributed to the recurrent evolutionary conflict between CenH3 and centromeric repeats over centromere control^9^,^10^. This conflict is central to the centromere drive theory, which explains the rapid evolution of eukaryotic centromeres and karyotypes and the establishment of reproductive barriers^9^,^10^,^11^,^12^,^13^. Under the model of centromere drive, centromeric repeats selfishly exploit the asymmetry of female meiosis (in which only one meiotic product survives, whereas male meiosis is usually symmetric with four surviving products) to secure preferential transmission to the egg at the expense of their homologous counterpart^9^,^10^. Expansions (or contractions) of centromeric repeats may lead to increased (or decreased) recruitment of CenH3 and thus to a larger (or smaller) kinetochore, which attracts more (or fewer) microtubules, resulting in the capture of such a kinetochore by the meiotic egg pole when it emanates more (or fewer) microtubules^9^,^10^. This driving centromere then rapidly spreads through a population, along with potential negative effects and hitchhiking mutations^9^,^10^. Therefore, CenH3 mutations that balance the binding capacity between homologous centromeres are positively selected because they suppress centromere drive along with the associated negative effects^9^,^10^.

The prediction that follows is that adaptively evolving CenH3 should be more frequent in clades with asymmetric meiosis than in clades with only symmetric meiosis, where there is no opportunity for conflict because all four meiotic products survive^9^,^14^. Although the prediction of differential CenH3 evolution between asymmetric and symmetric clades is a central pillar of the centromere drive theory, it has actually never been tested. Previous studies had suggested that CenH3 is indeed evolving adaptively only in lineages with asymmetric meiosis and under purifying selection in lineages with symmetric meiosis. However, data on CenH3 evolution in lineages with symmetric meiosis are scarce; so far only two clades of yeasts, two species of *Plasmodium* and two species of *Ostreococcus* have been analyzed^15^,^16^,^17^. Moreover, since there are also studies that detected a lack of positive selection in asymmetric clades^15^,^18^, the lack of detection of positive selection so far reported from species with symmetric meiosis is not sufficient to draw a solid conclusion on the differential CenH3 evolution in symmetrics and asymmetrics.

Taken together, the centromere drive model predicts higher frequency of positive selection episodes in the evolution of CenH3 in eukaryotic clades with asymmetric meiosis (from now on referred to as asymmetrics) than in clades with only symmetric meiosis (from now on referred to as symmetrics). Since not just centromere drive, but other factors may affect the incidence of positive selection events and thus mask the effects of centromere drive, it is necessary to analyze a large enough and representative dataset to detect statistically significant difference in positive selection frequency between the asymmetrics and symmetrics. To achieve such representativeness, we analyzed 191 CenH3 sequences (75% of which is analyzed here for the first time) sequences from a total of seventeen eukaryotic clades of plants, animals, fungi, ciliates and apicomplexa differing in meiotic symmetry (8 asymmetrics and 9 symmetrics; Table 1) by a unified methodical approach.

## Methods

We obtained all 191 CenH3 sequences from GenBank and Joint Genome Institute databases^19^,^20^,^21^ using BLAST searches. The sources and accession numbers for all sequences are supplied in Supplementary File S1.

Because the quality of the alignment is absolutely crucial for selection analyses, we used BAli-Phy software, which accounts for alignment uncertainties and avoids problems with biasing alignments towards guide trees because alignments and phylogenetic trees are estimated simultaneously^22^,^23^. The codon alignments and phylogenetic trees were jointly inferred in BAli-Phy v2.3.5 using M0 substitution model and RS07 model for indels. Because ciliate nuclear code is not implemented in BAli-Phy v2.3.5, *Tetrahymena* sequences were aligned at amino acid level using LG substitution model and RS07 model for indels and then backtranslated to nucleotide sequences. For each of the seventeen clades, we ran ten independent chains until they converged and then pooled the results. We masked each codon with a reliability score below 80% as “NNN” prior to selection analyses, and we used maximum *a posteriori* tree for all selection analyses, except primates, where a known species tree was used. All BAli-Phy alignments of CenH3 before and after masking the unreliable residues are supplied in Supplementary File S2. When only a partial sequence of CenH3 was available, we treated the missing part of the gene as missing data, and the gaps were replaced with “?”. The alignments and phylogenetic trees that we used for selection analyses are supplied in Supplementary File S3.

Once we had alignments and phylogenetic trees of the CenH3 sequences for each of the seventeen clades (Supplementary Files S2 and S3), we employed codon substitution models to infer the selective pressures acting on a protein from the non-synonymous/synonymous substitution rate ratio (dN/dS = ω). Non-synonymous substitutions in a codon lead to amino acid changes, while synonymous substitutions do not. If there is no selective pressure (neutral evolution), non-synonymous and synonymous substitutions are expected to occur at the same rate, with ω = 1. Purifying selection, which keeps the protein as it is, is indicated by ω < 1, and positive selection favoring substitutions that change the amino acids in a protein is indicated by ω > 1. If purifying selection is relaxed, ω tends to be elevated towards 1. Likewise, if positive selection is relaxed, ω tends to decrease towards 1.

We inferred three positive selection measures for each of the 16 analyzed clades: (i) the proportion of positively selected branches in the tree, (ii) the proportion of positively selected codon in the alignments and (iii) the overall ω ratio. To determine the frequency of positively selected branches, we ran the data through the branch-site random effects likelihood (BS-REL) model of codon substitution^24^. BS-REL allows ω to vary across both codons and branches and infers selective regimes independently for each branch of a given phylogeny, pooling information across all codons^24^,^25^. To assess the frequency of positively selected codons, we analyzed CenH3 from each of the seventeen clades using the mixed effects model of evolution (MEME) model of codon substitution that is capable of identifying instances of positive selection at the level of individual codons^25^. Both BS-REL and MEME analyses were performed using BS-REL and MEME modules as implemented on the DataMonkey web server^26^. Finally, we examined the overall ω ratio to evaluate CenH3 evolution across all codons and branches for each clade using a one-ratio (M0) model with codon frequencies option set to F3 × 4 in the codeml module of PAML4.7 27.

**Figure 1:**
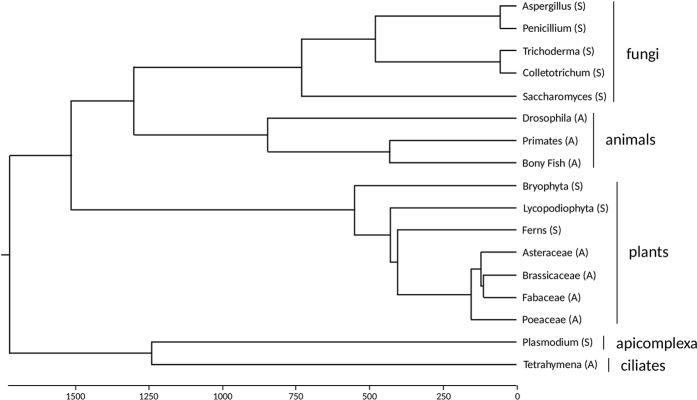
Phylogenetic tree of analyzed clades. A dated phylogenetic tree of all seventeen analyzed clades. The dating is based on divergence times obtained from TIMETREE. Scale axes shows time in millions of years. Clades having asymmetric meiosis are labeled with (A), while clades with symmetric meiosis are labeled with (S).

To assess whether asymmetrics and symmetrics significantly differ in the frequency of positively selected branches and codons and the overall ω ratio, we employed Mann-Whitney U test. However, it is possible that the phylogenetic relationships between analyzed clades may violate statistical independence of analyzed values required for Mann-Whitney U test. Therefore, we have also assessed the differences between asymmetrics and symmetrics using phylogenetically corrected statistical analyses. For that purpose, we have constructed a dated phylogenetic tree of all seventeen clades using divergence times from TIMETREE (Fig. 1;^28^,^29^). Statistical significances of phylogenetically corrected differences between asymmetrics and symmetrics were inferred using phylogenetic generalized linear models (*pgls*) as implemented in *caper* R-package (*pgls* function^30^). *Pgls* analyses were performed in R 3.2.3 31. The R-script used for *pgls* analyses is supplied in Supplementary File S4.

## Results and Discussion

**Figure 2:**
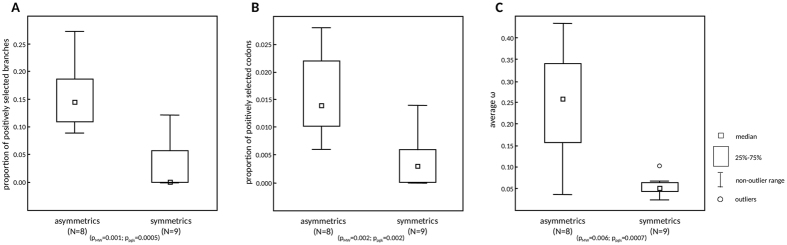
Distinct CenH3 evolution patterns in the asymmetrics and symmetrics. Box plots show differences between asymmetrics and symmetrics in (**A**) the proportion of positively selected branches after the correction for multiple testing; (**B**) the proportion of positively selected codons and (**C**) the average ω. N – the number of clades analyzed in each group (see Table 1 for details), p_MW_ – significance from Mann-Whitney U Test, p_pgls_ – significance from *pgls* analysis.

To determine the frequency of positively selected branches, we have run the BS-REL analysis for each of the seventeen clades. After correction for multiple testing, BS-REL detected positively selected branches in five clades (clades with p_corr_ ≤ 0.05), all of them from the asymmetrics, while no positively selected branches were detected in the symmetrics (Table 1). When compared, the frequency of positively selected branches in the symmetrics was significantly lower than in the asymmetrics (p_MW_ = 0.027, p_pgls_ = 0.012), which is in accordance with the prediction of the centromere drive model. However, such a result does not rule out positive selection from symmetrics because the corrections for multiple testing may be too conservative and may lead to false-negative results. Therefore, we have also used uncorrected p-values and compared the proportions of positively selected branches between asymmetrics and symmetrics (Table 1). Again, symmetrics had a significantly lower proportion of branches under positive selection than asymmetrics (p_MW_ = 0.001, p_pgls_ = 0.0005; Fig. 2A).

To assess the frequency of positively selected codons (from all codons in the alignment) in asymmetrics and symmetrics, we analyzed CenH3 from each of the seventeen clades using MEME (see Methods for details). The symmetrics had a significantly lower proportion of positively selected codons than asymmetrics (p_MW_ = 0.002, p_pgls_ = 0.002; Fig. 2B) as predicted by the centromere drive model. Moreover, the asymmetrics and symmetrics differed also in the distribution of positively selected codons across functional domains of the CenH3 protein (N-terminus and histone fold domain - HFD).

Since the HFD is required for centromere targeting and directly interacts with centromeric DNA^28^, it is thought to play a prominent role in the centromere drive^3^,^13^ and asymmetrics might thus be expected to experience a higher frequency of positive selection events in the HFD than symmetrics. In agreement with this expectation, in symmetrics, only two (Bryophyta and *Trichoderma*) of nine clades (22%) showed positively selected codon in the HFD, while in asymmetrics, five of eight (63%) displayed positively selected codons in this domain (Table 1).

Finally, to evaluate whether and how the asymmetrics and symmetrics differ in CenH3 evolution on a global scale, i.e., across all codons and branches, we examined the overall ω ratio for each clade. In each clade, CenH3 showed an average ω < 1 (Table 1), suggesting that most codons have been evolving under purifying selection for most of their evolutionary history. This observation may be expected because positive selection usually acts on only a few codons for a limited amount of time^30^. However, we detected a striking difference in the average ω between asymmetrics and symmetrics (Fig. 2C). The symmetrics showed significantly lower ω than the asymmetrics (p_MW_ = 0.006; p_pgls_ = 0.0007; Fig. 2C). Such a low ω implies that CenH3 has been evolving under much stronger purifying selection over evolutionary time in the symmetrics than in the asymmetrics and/or that the asymmetrics experienced more episodes of positive selection which is in accordance with the BS-REL and MEME analyses (see above).

Using three different models of codon substitution, we have shown that CenH3 has been evolving under different selective pressures in clades with opportunities for centromere drive (asymmetrics) compared with clades without such opportunities (symmetrics). In accordance with the predictions of the centromere drive model and previous reports based on smaller datasets^15^,^16^,^17^, episodes of positive selection acting on CenH3 appear to be much less frequent in symmetrics (Fig. 2A–C).

Interestingly, we have detected adaptively evolving CenH3 in the ciliate genus *Tetrahymena* from asymmetrics (Table 1) where the previous study reported the absence of positive selection^18^. In contrast to Elde *et al*. who suggested unsuppressed centromere drive in *Tetrahymena*^18^, our results indicate that centromere drive might have been suppressed in these ciliates. On the other hand, in CenH3 of *Drosophila* and Primates, we have detected positive selection only in N-terminus (Table 1), while previous studies reported positively selected codons in both N-terminus and HFD^1^,^5^,^7^. This discrepancies may be attributed to the fact that, for the detection of positively selected codons, we have used branch-site models of codon substitution instead of site models employed in the previous studies. Although the branch-site models and site models may perform differently because they have different assumptions^32^, it is important that we have used the same branch-site models consistently in all our analyses, an approach allowing us to compare CenH3 evolution in symmetrics and asymmetrics.

A recent study suggested that the evolution of centromeric DNA repeats and CenH3 might be driven by selection of centromere-linked genes rather than by centromere drive in inbred lines of maize^33^. However, selection on centromere-linked genes and centromere drive are not mutually exclusive evolutionary mechanisms. If the selection on centromere-linked genes was the only mechanism responsible for the evolution of centromeres and kinetochore proteins, there should be no competition for surviving meiotic products and symmetrics and asymmetrics should not differ in the frequency of positive selection acting on CenH3. The observation of different frequency of positively selected CenH3 between symmetrics and asymmetrics (Table 1; Fig. 1) suggests that centromere drive has indeed been operating in asymmetrics. Furthermore, in *Saccharomyces* (symmetrics), centromeric DNA has been rapidly evolving due to very high mutation rate^34^,^35^, yet their CenH3 does not show almost any signs of positive selection^16^,^17^ (Table 1). Since there is no opportunity for centromere drive in *Saccharomyces* due their symmetric meiosis, these findings suggest that meiotic asymmetry may indeed be the main factor responsible for the adaptive evolution of CenH3.

In the light of the centromere drive model and the results of our and previous studies, it is likely that before asymmetric meiosis appeared on the evolutionary scene, CenH3 had been evolving under strong purifying selection with occasional episodes of positive selection. After meiotic asymmetry appeared, positive selection occurred at a higher frequency because CenH3 was forced to counteract the negative consequences of centromere drive. Interestingly, some asymmetrics have evolved holokinetic chromosomes^36^ that have been hypothesized to suppress centromere drive^10^,^17^. The absence of positively selected CenH3 recently found in holokinetic plant genus *Luzula*^37^ and the fact that holokinetic chromosomes have so for been reported only in asymmetrics support this hypothesis.

## Additional Information

**How to cite this article**: Zedek, F. and Bureš, P. CenH3 evolution reflects meiotic symmetry as predicted by the centromere drive model. *Sci. Rep.* **6**, 33308; doi: 10.1038/srep33308 (2016).

## Supplementary Material

Supplementary File S1

Supplementary File S2

Supplementary File S3

Supplementary File S4

## Figures and Tables

**Table 1:** Results of CenH3 selection analyses for analyzed clades.

	No. of seqs	Mean ω	%PosBr (corr)^a^	%PosB.r^b^	%PosCod^c^	N/H^d^	Tree length^e^
Asymmetrics							
*Asteraceae*	7	0.187	0	0.272	0.013	1/1	1.698
Bony Fish	11	0.126	0.053	0.158	0.013	1/1	4.856
*Brassicaceae*	20	0.433	0	0.132	0.028	4/2	3.179
*Drosophila*	16	0.268	0.069	0.172	0.024	7/0	4.065
*Fabaceae*	17	0.262	0.032	0.129	0.015	1/2	4.189
*Poaceae*	12	0.247	0	0.095	0.020	1/3	3.854
Primates	14	0.412	0.040	0.200	0.007	1/0	1.116
*Tetrahymena*	13	0.036	0.043	0.087	0.006	1/0	8.465
Symmetrics							
Bryophyta	10	0.103	0	0.059	0.014	1/1	2.242
Ferns	8	0.067	0	0	0	NA	7.587
Lycopodiophyta	5	0.024	0	0	0	NA	6.045
*Aspergillus*	18	0.062	0	0.121	0.006	2/0	8.725
*Colletotrichum*	7	0.041	0	0	0.007	1/0	1.096
*Penicillium*	11	0.056	0	0.053	0.003	1/0	5.814
*Trichoderma*	6	0.042	0	0.111	0.006	0/1	1.750
*Saccharomyces*	9	0.050	0	0	0.003	1/0	6.921
*Plasmodium*	7	0.043	0	0	0	NA	3.247

^a,b^% of positively selected branches with p < 0.05 after (a) and prior to (b) the correction for multiple testing.

^c^% of positively selected codons with p < 0.05.

^d^Number of positively selected codons in N (N-tail) and H (HFD); NA - not applicable.

^e^Tree lengths defined as number of nucleotide substitutions per codon inferred in PAML 4.
