# Supplementary material for: CenH3 evolution reflects meiotic symmetry as predicted by the centromere drive model
Source: Sci Rep. 2016 Sep 15;6:33308. doi: 10.1038/srep33308 (PMC5024113; doi:10.1038/srep33308)
Supplement: Supplementary File S2 [file srep33308-s2.pdf]

CenH3 evolution reflects meiotic symmetry as predicted by the centromere drive model

František Zedek, Petr Bureš

Supplementary File S2 – CenH3 alignments before and after masking unreliable codons

Aspergillus – CenH3 alignment before masking unreliable residues inferred in Bali-Phy

>Aspergillus_fumigatus
ATGCCGCCGAAGACTGGA---------CGAGGACGTAAAGCAATT---GGGGCT-----------------------CCTCGAACCCAACCTCGA---GATGACGGAGGT---------------------------------GCAGGTCCA------TCTAAC------------------GCT---GCGGCTACA-----------------------GCATCACCT---TCT---
ACTCGGGGACAGCCGCCCAAATCTCGC---GGG---GGA-----------ACAAGG-----------------GGGGGAAAGCGACCAGCCCAC---GCCCCG--AGGCCG---TCAGACGTC-----------------
CAACCTGGGGACCCAACCCCTCAAGGTCGCCGACACCGGTACAAGCCGGGAACCGTCGCATTGAAAGAGATCCGCAGATACCAGCGCTCATATGACCTCCTTATTCAGAAGCTACCCTTTGCGCGACTCGTACGCGAGGTCGCATTGGAACTTCTTCCCGCCGAAGTAGGCGCGGAGCTACGGTGGCAGTCGCACGCTATTCAGGCGCTACAGGAGGCCGCCGAAGCTTTCCTCGTCCACCTTTTCGAGGATACCAATCTCTGTGCTTTGCATGCTAAGCGTGTAACGATTATGCAGAAAGATATCCA
GCTCGCCCGAAGAATACGCGGTATTTGGGGCGGTCTGGGT
>Aspergillus_clavatus
ATGCCACCAAAGACAGGA---------CGGGGCCGGAAGGCAGTC---AGCGCTGGAAGA-----------------CCTCGA-----------AAA--GAC---------GAA-----------------------------TCTGGCCCC------TCAGAC------------------GCA---CCTGCGACT-----------------------GCATCACCT---TCG---ACCCGAGGCCAG--
-CGCAAGTCAAGC---GGA---GCG-----------ACAAGG------------------GGGGGCAAGCGACCGGCCGGC---GCACCG--AGAAAA---TCAGACGTT-----------------
CAACCTGGAGACCCAACCCCTCAAGGTCGCCCACGCCGATACCGACCGGGAACGGTTGCCCTGAAAGAGATCCGCAAATATCAGCGCTCGTACGATCTGCTCATCCAAAAGTTACCCTTTGCGCGACTCGTCCGCGAGGTCGCCTTGGACCTCCTCCCCACCGAGGTCGGCGCCCAATTGCGATGGCAGTCGCATGCAATCCAAGCGCTCCAGGAAGCTGCTGAAGCTTTCCTGGTCCATCTGTTCGAGGACACCAATCTCTGCGCCATCCATGCTAAGCGCGTAACAATCATGCAGAAAGATATTCA
GCTTGCTCGAAGAATTCGCGGTGCTTGGGGTGGCCTGGGT
>Aspergillus_terreus
ATGCCACCCAAAACAGGG---------CGAGGCCACAAGATCACC---GCCGCG-----------------------CCCCGG---------------ACAGAGGCAAGCAGCAGTCGTACCACC---GCC---------GCCGCCGCCGGCCCG------TCG---------------------------------ACG-----------------------GCGTCGCCG---TCC---
GCCCACAGGGTG---CGCAAATCCACC---GGA---CCA-----------TCCAGA-----------------GGCGGGAAGCGACCGGCCGGC---GCGCCG--AGAGAC---TCCGACGTT-----------------
CAACCCGGCGATCCCACCCCGACCGGTCGTCGTCGCCGCTACCGACCTGGCACCGTCGCCCTCAAGGAAATCCGCAAGTACCAACGGTCATATGACCTCCTCATTCAGAAGCTCCCCTTCGCGCGACTCGTCCGTGAAGTCGCACTCGATATCCTCCCCGCCGAGCGCGGCGCCGAGCTCCGTTGGCAGTCGCATGCCATCCAGGCCCTCCAGGAGGCCGCCGAGGCGTTCCTGGTGCACCTGTTCGAGGACACGAACTTGTGCGCCCTGCACGCGAAACGAGTCACCATCATGCAGAAGGATATCC
AGCTTGCGCGACGAATTCGCGGTGTGTGGGGTGGTTTGGGC
>Aspergillus_kawachii
ATGCCCCCCAAGACAGGCGGTCGCGGTCGCGGTCGCAAGAGCGTTACGGCGGCG-----------------------CCGCGA---CAATCAACAGCT---GGCGACCGTGCT------------------------------GCCGCAAGCTCC------TCAACA------------------GCA---GCTATAACG-----------------------GCCTCGCCG---GCG---
GCCAAGAAAGTG---CGCAAATCCACT---GGA---GCG-----------ACTAGG-----------------GGAGGGAAAAGGCCTGCTGGAAAGGCACCC--AGGGAG---TCCGATGTT-----------------
CAGCCTGGTGACCCTACACCACAAGGACGTGTACGCCGCTACAAGCCCGGAACCGTGGCTCTCAAGGAGATCCGCAAGTACCAACGCTCGTACGATCTGCTTATCCAAAAGCTTCCTTTCGCTCGGCTGGTCCGCGAGGTCGCGCTTGACCTCCTCCCTTCAGAAGTTGGCGCCGAGCTGCGGTGGCAGTCGCATGCGATCCAAGCGCTCCAGGAAGCAGCCGAAGCTTTCCTTGTACATCTCTTCGAGGACACCAACCTCTGCGCGATTCACGCCAAGCGCGTGACCATCATGCAGAAAGATATCCA
ACTTGCTCGACGCATCCGTGGTGTCTGGGGTGGTTTGGGC
>Aspergillus_niger
ATGCCTCCCAAGACAGGCGGT------CGCGGTCGCAAGAGCATTGTGGCGGCG-----------------------CCGCGA---CAATCAACAGCT---GGCGACCGTGCT------------------------------GCCGCAAGCTCG------TCGAAA------------------GCA---GCTGCAACG-----------------------ACGTCGCCG---GCA---
GCTAAAAAAGTG---CGCAAATCCACT---GGA---CCG-----------GCTAGG-----------------GGAGGGAAAAGGCCTGCTGGAAAGGCACCC--AGAGAG---TCCGACGTT-----------------
CAGCCTGGTGATCCCACACCACAAGGACGTGTACGCCGCTACAAGCCTGGAACCGTGGCTCTCAAAGAGATCCGCAAGTACCAGCGCTCTTATGATCTGCTTATCCAAAAGCTTCCTTTTGCTCGGCTGGTCCGCGAGGTCGCTCTTGACCTCCTCCCTTCAGAAGTTGGCGCCGAGCTGCGGTGGCAGTCGCATGCGATCCAAGCGCTCCAAGAAGCAGCCGAAGCTTTCCTTGTACATCTCTTCGAGGACACCAACCTCTGTGCTATTCACGCCAAGCGCGTGACCATCATGCAGAAAGATATCCAA
CTTGCTCGACGCATTCGTGGTGTCTGGGGTGGTTTGGGC
>Aspergillus_brasiliensis
ATGCCCCCCAAGACAGGCGGT------CGCGGTCGCAAGAGCATTGTGGCGGCG-----------------------CCGCGA---CAATCAACAGCT---GGCGACCGTGCT------------------------------GCCGCAAGCTCC------TCGAAA------------------GCA---GCTGCAACG-----------------------ACATCGCCG---GCG---
GCGAAGAAAGTG---CGCAAATCCACT---GGA---CCG-----------GCTAGG-----------------GGAGGGAAAAGACCTGCTGGAAAGGCGCCC--AGACAG---TCCGATGTT-----------------
CAGCCTGGTGACCCCACGCCACAAGGACGTGTGCGCCGCTACAAGCCCGGAACTGTGGCTCTTAAAGAGATCCGCAAGTACCAGCGCTCGTACGATCTGCTTATTCAAAAGCTTCCTTTTGCTCGGCTGGTCCGCGAGGTCGCGCTTGACCTCCTCCCTTCAGAAGTTGGCGCTGAGCTGCGGTGGCAGTCACATGCGATCCAAGCGCTTCAGGAAGCAGCCGAAGCTTTCCTCGTACATCTCTTTGAGGATACCAATCTCTGTGCTATCCACGCCAAGCGCGTGACCATCATGCAGAAAGATATCCA
ACTTGCTCGACGCATCCGTGGTGTCTGGGGTGGTTTGGGC
>Aspergillus_acidus
ATGCCCCCCAAGACAGGCGGTCGCGGTCGCGGTCGCAAGAGCGTTACGGCGGCG-----------------------CCGCGA---CAATCAACAGCT---GGCGACCGTGCT------------------------------GCCGCAAGCTCC------TCCACA------------------GCA---GCTACAACG-----------------------GCCTCGCCG---GCG---
GCCAAGAAAGTG---CGCAAATCCACT---GGA---GCG-----------ACTAGG-----------------GGAGGGAAAAGGCCTGCTGGAAAGGCACCC--AGAGAG---TCCGATGTT-----------------
CAGCCTGGTGACCCTACACCACAAGGACGTGTACGCCGCTACAAGCCCGGAACCGTGGCTCTCAAGGAGATCCGCAAGTACCAACGCTCGTACGATCTGCTTATCCAAAAGCTTCCTTTCGCTCGGCTGGTCCGCGAGGTCGCGCTTGACCTCCTCCCTTCAGAAGTTGGCGCCGAGCTGCGGTGGCAGTCGCATGCGATCCAAGCGCTCCAGGAAGCAGCCGAAGCTTTCCTTGTACATCTCTTCGAGGACACCAACCTCTGCGCGATTCACGCCAAGCGCGTGACCATCATGCAGAAAGATATCCA
ACTTGCTCGACGCATCCGTGGTGTCTGGGGTGGTTTGGGC
>Aspergillus_tubingensis
ATGCCCCCCAAGACAGGCGGTCGCGGTCGCGGTCGCAAGAGCGTTACGGCGGCG-----------------------CCGCGA---CAATCAACAGCT---GGCGACCGTGCT------------------------------GCCGCAAGCTCC------TCAACA------------------GCA---GCTACAACG-----------------------GCATCGCCG---GCG---
GCCAAGAAAGTG---CGCAAATCCACT---GGA---GCG-----------ACTAGG-----------------GGTGGGAAAAGGCCTGCTGGAAAGGCACCC--AGAGAG---TCCGATGTT-----------------
CAGCCTGGTGACCCCACACCACAAGGACGTGTACGCCGCTACAAGCCCGGAACCGTGGCTCTCAAGGAGATCCGCAAGTACCAGCGCTCGTACGATTTGCTTATCCAAAAGCTTCCTTTCGCTCGGCTGGTCCGCGAGGTCGCGCTTGACCTCCTCCCTTCAGAAGTTGGCGCCGAGCTGCGGTGGCAGTCGCATGCGATCCAAGCGCTCCAGGAAGCAGCCGAAGCTTTCCTTGTACATCTCTTCGAGGACACCAACCTCTGTGCGATTCACGCCAAGCGCGTGACCATCATGCAGAAAGATATCCA
ACTTGCTCGACGCATCCGTGGTGTCTGGGGTGGTTTGGGC
>Aspergillus_wentii
ATGCCACCAAAGACCGGT---------CGAGGCAATAAGATTATC---GCTGCT-----------------------CCCAGA---GCAAAG---GCT--------------GCAAAGGAAGAGGCGGAGGCAGGTCCA------TCAAGA------------------GCAAGCCCTGCGACC-----------------------GCGTCGCCA---GCA---
ACCCGAGGCCGA---CGCAAGTCCGCT---GGT---GCA-----------AGCAGA-----------------GGAGGAAAGAAACCCGCTGGA---GTATCGAAA---CCCAAAGCAAAACCATCA--------------AAACAA---ACAGATGTC-----------------
CAGCCCGGAGACCCTACCCCTCAAGGTCGTCGTCATCGATACAAGCCAGGAACCGTTGCCCTGAAAGAGATCCGTAAATACCAGCGCTCCTATGATCTGCTGATCCAGAAGCTTCCCTTTGCGCGACTCGTGCGTGAGGTTGCGTTGGAGCTTCTCCCCGCCGACGTGGGAGCAGAGCTGCGATGGCAGTCACATGCGATCCAGGCCCTGCAGGAAGCTGCAGAGGCTTTCCTTGTCCATCTCTTCGAGGATACAAACCTGTGCGCTCTACACGCTAAGCGTGTTACCATCATGCAGAAGGATATTCA
GCTTGCCCGAAGAATCCGCGGTGCCTGGGGTGGATTGGGT
>Aspergillus_aculeatus
ATGCCGCCTAAGACAGGG---------CGCGGTCGCAAGTCAATT---GCTGGG-----------------------CCACGG---AAATCAACGGCC------GCCGCCGCCGCCGCCGCCGCCACTACTGCTGCTGCTACT--------------GATGATGGTGCC---------------GCC---------GCTGCTGCCGGCCCG------TCAACT------------------GCC---CCAGCTACGACGACCACAACC------------------CCC---GCC--------------------------
-----------------------GCTCGC---GGG---CGTAAACCTGCG---GGT---GTC-----------ACCAGA--------------------GGCAAGCGACCGGCTAAA---GCGCCC--AGGACA---TCCAATGTT-----------------
GAACCGGGCGATCCCACCCCCACAGGTCGTCGTCGCCGGTACAAACCTGGAACGGTTGCCCTGAAGGAGATCCGCCATTACCAACGCTCATTCGACCTGCTCATCCGAAAGCTGCCATTCGCGCGACTGGTCCGCGAGGTTGCCTTGGATCTTCTCCCCGCTGAAGTCGGCTCCGAGCTGCGCTGGCAATCGCATGCGATCTTGGCCCTCCAGGAAGCCGCCGAGGCGTTCCTGGTCCATCTTTTCGAGGACACCAATCTCTGTGCCATTCATGCCAAGCGCGTGACCATCATGCAAAAGGATATCCA
GCTTGCTCGACGCATCCGGGGTGTCTGGGGTGGTCTGGGT
>Aspergillus_flavus
ATGCCACCGAAGACAGGA---------GGAGGTCGCAAGGTAATC---GCCGCA-----------------------TCGCGA---GCAAAG---GCGGACGGTGCC---------------GGAAAAGAAAGCGCA--GCTGCAAAA-----------------------GGGTCCCCT---TCG---ACTCGAGGA-----
-CGCAAGTCCGCT---GGA---GGT-----------GCCAGA-----------------GCGGGGAAGCGACCGGCTGGT---GCATCG--AGGAAA---TCAGATGTC-----------------
CAACCTGGCGATCCTACCCCTCAGGGCCGCCATCGCCGTTACAGACCGGGCACTGTTGCCCTCAAAGAAATCCGCAAATACCAACGTTCTTATGACCTCCTTATACGAAAGCTCCCATTCGCAAGACTTGTGCGCGAAGTTGCATTGGATCTACTCCCCGCAGATGTAGGATCCGAATTGCGCTGGCAGTCGCAAGCCATCATGGCGCTGCAGGAGGCCGCGGAAGCCTTTCTTGTTCATCTATTCGAGGACACGAACCTGTGTGCTCTACACGCGAAGCGAGTGACTATCATGCAGAAAGATATCCA
GCTTGCTCGGAGAATCCGTGGCGCTTGGGGTGGATTAGGC
>Aspergillus_glaucus
ATGCCACCAAAGACAGGA---------AAAGGCCGTAAAGTCGTC---TCAGAA-----------------------CCGCGA---TCGCGCCCCAGCACCGGCGACGGAGAA---------------------------------GCAGGCCCA------TCA---TCAAAACCGCAACAA--------------ACC-----------------------GCGTCGCCA---GCAAACAAAAGGAAACCCAAGGCTTCC------------------
--------ACT-----------------------TCCGGAGAAGCAAGA-----------------------------------GACAAGCGGCCATCGGGA---GTAACAAAAACACCAAAG-----------GCAGCAAAGACAGCAATAACA---TCAGACGTC-----------------
CAACCCGGCGACCCAACCCCCCAAGGCCGTTCACGCCGCTACAAACCCGGAACCGTCGCTCTAAAAGAAATCCGCAAATACCAACGCTCCTTCGACCTCCTCATCCAAAAGCTCCCCTTCGCGCGCCTCGTCCGTGAAGTCGCCCTGGAACTGCTCCCCGCAGACGTAGGCGAACAGCTCCGGTGGCAGTCGCATGCGATCCAAGCACTACAAGAAGCCGCAGAGGCATTCCTCGTCCATTTATTCGAAGATACTAATTTGTGTGCTCTGCATGCTAAGCGCGTGACTATTATGCAAAAGGATATCCAG
CTTGCACGGAGGATTCGTGGTGCTTGGGGTGGATTGGGA
>Aspergillus_versicolor
ATGCCTCCGAAA------------------GGACGGAAG--------------------CCGGCCTCG---GCCACAGCCGGACCC------TCGAAA------------------ACG---CCCGCA--------------------------GCAGGCCCC---TCC--AAATCCGCC---GGA---
GTG---AAAAAAGGTCCCAAA------------------GGGGGGAAGCGACCA--AGGACA---TCAGACGTT------
CAACCAGATGTTCAACCCGGAGACCCAACCCCCAAAGGCCGCCGCCACCGGTACAAGCCCGGCACCGTCGCCCTCAAGGAAATCCGCAAGTACCAACGCTCATATGATCTTCTCCTCCGAAAACTCCCCTTCGCACGATTAGTGCGCGAGGTCGCATTGGACCTCCTCCCCGCAGAAGTCGGCGCGGAGCTACGGTGGCAGTCGCATGCAATCCTTGCGCTCCAAGAAGCCGCCGAGGCCTTCCTGGTACATTTGTTCGAAGACACGAATTTATGCGCCATCCACGCGAAACGGGTTACGATCATGC
AGAAGGATATCCAACTTGCGCGTAGAATTCGCGGGGCGTGGGGTGGTCTAGGC
>Aspergillus_sydowii
ATGCCTCCGAAA------------------GGACGGAAG--------------------CCAGCCTCG---GCCACCGCCGGGCCC------TCG------------------------------------ACAACAACAACGACAACAACAGGCGCAGGCCCC---GCT--AAATCCGCC---
GGA---GTG---AAAAAAGGTGCCAAGGGAGTCCAGGGAACCACGGGGGGGAAGCGACCA-----------CCA--AGGACA---TCAGACGCT------
CAATCAGATGTTCAACCCGGAGACCCAACCCCCAAAGGCCGCCGTCACCGGTACAAGCCCGGCACCGTCGCCCTGAAGGAAATCCGCAAGTACCAGCGCTCATACGATCTCCTCCTCCGAAAACTTCCCTTTGCACGATTAGTGCGCGAGGTCGCATTGGACCTTCTCCCCGCAGAAGTCGGCGCGGAGCTGCGGTGGCAGTCGCATGCAATCCTTGCGCTCCAAGAGGCTGCCGAGGCCTTCCTAGTACACTTGTTCGAGGACACCAATCTATGCGCCATCCACGCGAAACGGGTTACGATCATGC
AGAAGGATATCCAGCTTGCGCGCAGAATTCGTGGGGCGTGGGGTGGTCTAGGC
>Aspergillus_nidulans
ATGCCCCCAAAA------------------GGACGAAAG--------------------CCATCCTCG--GCA---------GCTACCGCAGGTACCGGAAGCTCAAAA---------------GCGACA---TCCGCAAAGACAACCTCCACCACAACCTCAGGCGCCTCTCCAAAGGTAACTAAGAGCTCCAGAACATTAGGGTCAAAGGCATTGGCATCGGCATCGGTGTCG------------------------
---AAATCT-----------------------------AAG------------------GGAAGTAAGCGACCA-----------CCG--ACGGCACCATCAGACGAT------
CAATCCAGCATCCAACCCGGCGACCCAACCCCCAAAGGCCGTCGTCACCGGTACAAACCTGGTACCGTTGCCCTCAAAGAAATCCGCAAGTACCAACGCTCCTACGATCTGCTTCTCCGAAAATTGCCATTTGCACGTCTCGTCCGCGAAGTTGCTCTCGACATCCTCCCCGCAGACGTTGGCTCCGAGCTGCGATGGCAGTCGCATGCAATCCAGGCGCTCCAGGAAGCCGCTGAAGCCTTTTTGGTACACTTGTTCGAGGACACGAATCTTTGCGCCATCCACGCGAAACGAGTTACGATAATGCAG
AAGGATATCCAGCTTGCGCGTAGGATTCGCGGAGCTTGGGGTGGTCTTGGC
>Aspergillus_sojae
ATGCCACCGAAGACAGGA---------AGAGGTCGCAAGGTAATC---GCCGCA-----------------------TCGCGA---GCAAAG---GCAGACGGTGCC---------------GGAAAAGAAAGCGCAGCTGCAGGCCCA------TCCGGT------------------GCA---GCTGCAAAA-----------------------GACTCCCCT---TCG---
ACTCGAGGA------CGCAAGTCCACT---GGA---GGT-----------GCCAGA-----------------GCGGGGAAGCGACCGGCTGGT---GCATCG--AGGAAA---TCAGATGTC-----------------
CAACGTGGCGATCCTACCCCTAAGGGCCGCCATCGCCGTTACAGACCGGGCACTGTTGCCCTCAAAGAAATCCGCAAATACCAACGTTCTTATGACCTCCTTTTACGGAAGCTCCCATTCGCAAGACTTGTGCGCGAAGTTGCATTGGATCTACTCCCCGCAGATGTAGGATCCGAATTGCGCTGGCAATCGCAAGCCATCATGGCGCTGCAAGAGGCCGCGGAGGCCTTCCTTGTTCATCTATTCGAGGACACGAACCTGTGTGCTCTACACGCGAAGCGAGTGACTATCATGCAGAAGGATATCCA
GCTTGCTCGGAGAATCCGTGGCGCTTGGGGTGGACTGGGC
>Neosartorya_fischeri
ATGCCGCCGAAGACTGGA---------CGAGGACGTAAAGCAATT---GGGGCT-----------------------CCTCGAACGCAACCTCGA---GATGACGGAGGT---------------------------------GCAGGTCCA------TCTAAT------------------GCT---GCGGCCACT-----------------------GCATCACCT---TCC---
ACTCGGGGACGGCCGCCCAAGTCCCGC---GGA---GGA-----------ACAAGG-----------------GGGGCCAAGCGACCAGCCCGC---GCCCCA--AGGCCG---TCAGACGTC-----------------
CAACCTGGGGACCCAACCCCTCAAGGTCGCCGACACCGGTACAAGCCGGGCACAGTCGCATTGAAAGAGATCCGCAAATACCAGCGCTCATATGACCTCCTTATTCAGAAGCTACCGTTTGCGCGACTCGTACGCGAGGTCGCATTGGAACTTCTCCCCGCTGAAGTAGGCGCGGAGCTACGGTGGCAGTCGCACGCTATTCAGGCGCTACAGGAGGCCGCCGAAGCTTTCCTCGTCCATCTTTTCGAGGATACCAATCTCTGTGCTTTGCATGCTAAGCGTGTAACGATTATGCAGAAAGATATCCA
GCTCGCCCGAAGAATACGCGGTGTTTGGGGCGGTCTGGGA
>Aspergillus_zonatus
ATGCCGCCTAAGACGGGG---------CGAGGCCGCAAGATGCTT---GCGGCG---------------CAAGCGCGCCCACGG---CCTGCAACAGGC---GACGGCGGCGCT---GACGGTGATGGGGCT---------GCTGCCGCGGGTCCG------TCG--
TCGCGCAACGATGGCGCAACGAAACCCGGTCGAGGAGGT---CGCAAGTCG------GGA---GGA-----------ACGAGG-----------------GGAGGGAAACGTCCC-----------CCT--GGGACG-----------
AGCGGGCAATCGAACATCCAACCGGGCGATCCCACCCCGAAAGGCCGTCGTCACCGATACAAGCCAGGCACCGTCGCCCTGAAGGAAATCCGAAAGTACCAGCGCTCATATGACCTACTGTTGCGCAAGCTGCCCTTTGCACGGCTTGTCCGCGAGGTCGCCCTGGAGCTATTACCGGCCAACGTGGGCGCCGAATTGCGCTGGCAGTCGCACGCGATCCAGGCTCTGCAGGAAGCCGCCGAGGCCTTCCTGGTCCATCTCTTCGAAGACACCAATCTGTGCGCCCTGCACGCCAAGCGCGTTACC
ATAATGCAAAAGGATATCCAGCTAGCGCGGAGAATACGTGGTGTATGGGGCGGCCTGGGT

Colletotrichum – CenH3 alignment before masking unreliable residues inferred in Bali-Phy

```
>Colletotrichum_eremochloae
ATGCCTCCCAAGAAGAGACAGTCAGACGCTCCGCGTCGGAAATCTCGTCAGAGCGACGTCCAACCTGGCGACCCTATCCCCAACCGAGGAAGGCGACGTTACAAGCCCGGTACAGTTGCTCTCAGGGAGATCCGAAAGTATCAAAGTGGCACTCAGCTTCTGCTGCGCCAGCTTCCATTCTCTCGCCTTGTACGCGAGATCGCCGAATCGGTGAGACCCCGAGGCGAAGCGATGCGATGGCAATCACAAGCGATACAAGCACTACAAGAAGCTGCGGAGGCTTTCTTGGTACACCTCTTCGAAGAC
ACCAACCTTTGCGCTATCCACGCCAAGAGAGTAACCATAATGCAAAAAGATATTCAGCTCGCAAGGCGCATTCGGGGCGTCTGGGGTGGCCTAGGT
>Colletotrichum_falcatum
ATGCCCTCCAAGAAGAGACAGTCAGACGCTCCGCGTCGGAAATCCCGTCAGAGCGACGTTCAACCTGGTGATCCTATTCCCAACCGAGGAAAGCGACGCTACAAGCCTGGTACAGTTGCTCTCAGGGAGATCCGAAAGTACCAAAGTGGCACTCAACTTTTGCTGCGTCAACTTCCATTCTCCCGCCTTGTACGCGAGATTGCCGAGTCGGTGAGACCCCGAGGCGAAGCAATGCGATGGCAATCACAAGCGATACAAGCACTACAAGAAGCTGCGGAGGCTTTCTTGGTACACCTCTTCGAAGAC
ACCAACCTCTGCGCTATCCACGCCAAGAGAGTAACCATAATGCAAAAAGATATTCAGCTCGCAAGGCGCATTCGGGGCGTCTGGGGTGGGCTAGGC
>Colletotrichum_higginsianum
ATGCCTCCTAGGAAGAGACAGTCGGACGCTTCACGTCGAAAATCGCGCCAGAGTGATGTACAACCTGGTGACCCGATTCCCAACCGAGGCAAGCGACGCTACAGACCCGGCACAGTTGCTCTCAGGGAAATCCGAAAATACCAGAGCGGCACCCAGCTTTTGCTGCGTCAGCTTCCCTTCTCTCGACTTGTCCGCGAAATTGCCGAGTCGGTGAGACCCCGGGGAGAGGCGATGCGGTGGCAATCACAAGCAATACAGGCACTTCAAGAAGCCGCCGAGGCTTTCTTGGTTCACCTATTTGAAGAT
ACAAACCTCTGCGCTATCCACGCCAAGAGAGTGACCATTATGCAAAAGGATATTCAGCTTGCCAGGCGCATTCGGGGCGTCTGGGGTGGATTGGGT
>Colletotrichum_somersetensis
ATGGCCCCCAAGAAGAGACTGTCAGACGCTCCGCGTCGGAAATCCCGTCAAAGCGACGTACAACCTGGCGATCCTATTCCCAACCGAGGAAAGCGACGCTACAAGCCTGGTACAGTTGCCCTCAGGGAGATCCGAAAGTATCAAAGTGGCACTCAGCTTCTGCTGCGCCAACTTCCATTCTCCCGGCTTGTACGCGAAATTGCCGAGTCGGTGAGACCCCGAGGCGAAGCAATGCGATGGCAATCACAAGCGATACAGGCGCTCCAAGAAGCTGCAGAGGCCTTCTTGGTACACCTCTTCGAAGAC
ACCAACCTCTGCGCTATCCACGCCAAGAGAGTAACCATAATGCAAAAAGATATTCAACTCGCGAGGCGCATTCGGGGCGTCTGGGGTGGGCTAGGC
>CColletotrichum_graminicola
ATGGCCCCCAAAAAGAGACATTCAGACGCTTCGCATCGGAAATCCCGTCAGAGCGACGTGCAACCCGGTGACCCTATTCCCAACCGAGGAAAGCGACGCTACAAGCCTGGTACAGTCGCTCTCAGGGAGATCCGAAAGTATCAAAGTGGCACTCAGCTTCTGCTGCGTCAACTTCCATTCTCTCGTCTTGTACGCGAAATTGCCGAGTCGGTGAGACCCCGAGGCGAAGCAATGCGATGGCAATCGCAAGCGATACAGGCACTCCAAGAAGCTGCGGAGGCCTTCTTAGTACACCTCTTCGAAGAC
ACCAACCTCTGCGCTATCCATGCCAAGAGAGTAACCATAATGCAAAAAGATATTCAGCTCGCAAGGCGCATTCGGGGCGTCTGGGGTGGGCTAGGC
>Colletotrichum_sublineola
ATGCCTCCCAAGAAGAGACAGTCAGACGCTCCGCGTCGGAAATCTCGTCAGAGCGACGTCCAACGTGGCGACCCTATCCCCAACCGAGGAAGGCGACGTTACAAGCCCGGTACAGTTGCTCTCAGGGAGATCCGAAAGTATCAAAGTGGCACTCAGCTTCTGCTGCGCCAGCTTCCATTCTCTCGCCTTGTACGCGAGATCGCCGAATCGGTGAGACCCCGAGGCGAAGCGATGCGATGGCAATCACAAGCGATACAAGCACTACAAGAAGCTGCGGAGGCTTTCTTGGTACACCTCTTCGAAGAC
ACCAACCTTTGCGCTATCCACGCCAAGAGAGTAACCATAATGCAAAAAGATATTCAGCTCGCAAGGCGCATTCGGGGCGTCTGGGGTGGCCTAGGT
>Colletotrichum_zoysiae
ATGGCCCCCAAGAAGAGACAGTCAGACGCTCCGCGTCGGAAATCCCGTCAAAGCGACGTACAACCTGGCGACCCTATTCCCAACCGAGGAAAGCGACGCTACAAGCCTGGCACAGTTGCCCTCAGGGAGATCCGAAAGTATCAAAGTGGCACTCAGCTTCTGCTGCGCCAACTTCCATTCTCCCGGCTTGTACGCGAAATTGCCGAGTCGGTGAGACCCCGAGGCGAGGCAATGCGATGGCAATCACAAGCGATACAGGCGCTCCAAGAAGCTGCAGAGGCCTTCTTGGTACACCTCTTCGAAGA
CACCAACCTCTGCGCTATCCACGCCAAGAGAGTAACCATAATGCAAAAAGATATTCAGCTCGCGAGGCGCATTCGGGGCGTCTGGGGTGGGCTAGGC
```

Penicillium – CenH3 alignment before masking unreliable residues inferred in Bali-Phy

>Penicillium_marneffei
ATGCCACCAAAAACAGGCCGAGGGCGCTCTTCCAAAGCC------------------------------ACCACCACCAGCAGCGCT------------CCAAACTCTCCCAGAGCAAGCAGAGCTGGCCCATCATCCCGTCAAGCACAGGAAGAAACAACCACAACATCAAGTCCCATCACAAGCCCA--AGATCACCAACTACCGCC---------------AGAAAT---
ACGAAGCAGCCAATGTCCCCAACAGCG------------------GCAAAA------ACAGCGGCAAAAACAACAAATAGAGTAACCAAGCGACCAGCCGGCAAAGCCCCG---------------------
CGATCATCAAACATACAACCGGGCGACCCAACCCCCATCGGCAAACGCCGCCGCTACAAGCCCGGCACCGTCGCCCTCAAAGAAATCCGCAAATACCAACGCTCCTTCGATTTGTTGATTAGCAAACTCCCCTTTGCCCGCTTAGTTCGCGAAGTCGCACTAGACCTGCTACCAGCCGAAGTAGGCGCCGAACTACGATGGCAATCGCACGCAATTATGGCGCTGCAAGAAGCTGCAGAGGCGTTTTTGGTGCATCTGTTTGAGGATACGAATCTGTGCGCCATTCATGCGAAGAGGGTTACGATTAT
GCAGAAGGATATTCAGCTTGCCAGGCGGATTAGGGGGATGTGGGCTGGTTTGGGC
>Penicillium_bilaiae
ATGCCCCCC---AAAGCCCGC------------AAA---------------------------------ACA------------GGC------------CCA------------GCAAGC---------CCA------------GCTCAA---GCG---------------CGCAAA---ACC---GCAACCAAGTCCCCCAAG----------------------------------
AAAGCAGCCTCAAAG------
CGCATGTCCAATGTCCAACCCGGTGATCCCACACCCCAAGGTCGCCGCCGGCGCTACAAGCCCGGCACCGTCGCCTTGAAAGAAATCCGCCGTTACCAACGCTCCTACGACCTATTAATCGCCAAACTGCCCTTTGCGCGCTTAGTCCGCGAAGTCGCACTGGATCTCCTCCCCGCAGAAGTTGGCGCCGAGCTACGATGGCAATCACAAGCTATCCAGGCGCTGCAAGAAGCCGCGGAGGCCTTCATGGTGCATTTATTCGAAGATACAAATCTGTGCGCGATACATGCGAAGCGCGTGACGATTAT
GCAGAAGGATATTCAGCTTGCGCGTCGGATTCGAGGAGCGTGGGGTGGTCTAGGC
>Penicillium_brevicompactum
ATGCCACCGAAGATGGCG---------------------AAGATCTTGAAGAAGAAAACCACCGAT---AGC-------------------------CCACAAAAG-------------------------------ACC---AAAGGTCGCAGACCAATCGCAAAA--GATGGCGATGGCTCACCA---------AATGCCGTG------CGCAAA---TCC-------------------AAG---CCC---------------GGAAAACAA---
AAAGCCAACAAGGCC--
GCAGATGTCCAACCGGGTGATCCAACCCCCACAGGCCGCCGCCGCCGCTACAAGCCTGGCACCGTGGCACTCAAAGAGATCCGCAAATACCAACGCTCCTACGACCTCCTCATAGCTAAGCTGCCGTTCGCGCGACTCGTTCGCGAAGTCGCTCTCGATCTTCTGCCCGCCGACACGGGCGCCGAACTGCGTTGGCAGTCACATGCGATCCTGGCACTGCAAGAAGCCGCCGAGGCATTCCTGGTTCATTTGTTCGAGGACACAAATCTGTGTGCGATCCACGCCAAGCGCGTCACAATTATGCAAA
AGGATATCCAGCTTGCGCGGAGAATTCGCGGGGTCTGGGCTGGTCTGGGT
>Penicillium_canescens
ATGCCACCCAAAATGGCACGA------------AAG----------------------------------TCA------------GCA------------CCA------------GGCAGC-------------------------GCACAAAAG--GGTCGCAAAACAGTCGCGACA--GCTGCCGCA----------------------GATGAC------TCACCAATACAAGCG---------------CGCAAAATCGCG-------------------AAA----------------------GGGAAGCAACCC------
GCAAAGACAGCA--
GCCGATGTCCAACCGGGCGACCCAACCCCTACAGGGCGCCGCCGCCGCTACAAACCAGGCACAAAAGCCCTAAAAGAAATCCGCCAATACCAACGCGGCTACGAGCTGCTCATCGCGAAACTACCCTTTGCCCGCTTAGTCCGTGAAGTGGCGCTCGATCTTCTCCCCGCCGACGTCGGTGCCGAATTGCGCTGGCAGTCGCATGCTATTCAAGCACTGCAGGAGGCTGCCGAGGCTTTCCTTGTTCATCTATTTGAGGATACGAATCTTTGCGCATTGCATGCTAAGCGGGTTACTATTATGCAGAA
AGATATTCAGCTTGCGAGGAGAATTCGGGGTGTGTGGGGTGGTTTAGGG
>Penicillium_chrysogenum
ATGCCACCCAAGCTAGCCAGC------------AAG----------------------------------ACA------------GCA------------CCA------------GGCAGC-------------------------GCACAAAAA-------------------------------AAC---AAAGTGCGCAAGACAGTCGCTGCG---CCGGCCGCC-------------------------GCCGATGGAACACCAACCAAAGCA---------------CAGAAA---ACG-------------------AAGGGA------------------
GCAAAGCAATCAAAAGTCGCAAAGACCACC---
GCCAACGTCCAACCCGGAGACCCAACCCCCACAGGCCGCCGACGCCGCTATAAGCCCGGCACCGTCGCTTTGAAAGAAATCCGCCGCTACCAACGCTCATACGACCTACTCATCGCTAAACTTCCCTTTGCGCGCTTAGTCCGCGAAGTCGCGCTCGATCTCCTCCCCGCCGACGTCGGCGCCGAGTTGCGCTGGCAGTCGCATGCCATCCAAGCTCTGCAGGAGGCCGCTGAAGCTTTCCTCGTCCACCTCTTCGAGGATACGAATCTATGCGCGCTACATGCGAAGCGCGTAACTATTATGCAGAA
GGATATCCAGCTTGCGCGGAGAATTCGCGGTGTCTGGGCTGGGCTTGGT
>Penicillium_expansum
ATGCCACCCAAGACAGCTCGC------------AAG----------------------------------ACA------------GGG------------CCG------------GGCAGC-------------------------GCGCAAAAA-------------------------------GGC---AAAGTGCGCAAGACAACCGCCGCG---CCCGCCGCC----------------------GATGGAGATGGAACACCAACAAAAGCA---------------CGCAAG---ACG-------------------AAGGGA------------------
GGAAAGCAATCAAAAGTC---------AAC---
GCCAACGTCCAACCCGGAGACCCAACCCCCACAGGCCGGCGCCGGCGCTACAAGCCCGGCACCGTCGCATTGAAAGAAATCCGCCGCTACCAACGCTCCTACGACCTCCTAATCGCCAAACTCCCCTTTGCACGCTTAGTCCGCGAAGTCGCACTCGATCTCCTCCCCGCCGACGTCGGCGCAGAGTTGCGCTGGCAGTCGCATGCTATCCAAGCTCTGCAGGAGGCCGCCGAGGCTTTCCTTGTTCATCTTTTCGAGGATACAAATCTCTGCGCGCTGCACGCGAAGCGCGTTACTATTATGCAGAA
GGATATCCAGCTTGCGCGGAGAATTCGCGGTGTCTGGGCTGGGCTTGGT
>Penicillium_fellutanum
ATGGCCACCAAGACAGCAATC------------AAG-------------------------------GCGCCC------------GCG------------CCG------------GCCAGC---------CCG------------GCGCAAAAT-------------------------------AGCCCA-------------------------GCGCAGAAGCGTAAGCGCGGACCGACTGTATCATCGCCTTCCAAACAGAAG--AAG---ACA-------------------ACG------GGA-------------------------------
GCAAAGACAACCAAA----------------------------------AAGCCAGCG------------
AGAGCATCAAATGTCCAGCCCGGCGACCCAACGCCCTCTGGTAAACGCCGCCGCTACAAGCCCGGCACTGTTGCCCTCAAGGAAATCCGACGCTACCAACGCTCTTACGATCTCTTAATCGCGAAACTCCCCTTTGCCCGACTCGTCCGCGAGGTCGCACTCGACCTTCTCCCCGCTGAAGTCGGCTCCGAGCTGCGCTGGCAGTCGCACGCTATCCAGGCATTGCAAGAAGCCGCCGAGGCATTTCTCGTGCATCTCTTCGAAGATACCAACCTCTGTGCGCTACATGCCAAGCGCGTCACAATCATG
CAGAAGGATATCCAGCTTGCGCGGCGGATAAGAGGCGTGTGGGGAGGTCTTGGA
>Penicillium_glabrum
ATGCCCACCAAATCAGCC---AAACGATTGATTCCT------------GGGAGC---------CCT------------GCA---AAG-------------------------------AGCCCT---GGAAGACCTCGCAAGAGCGCACCAGCTGCAGAAACAACCGAA-------------------GGATCACCGAGCGCAGCG---------------CGCAAG---TCA-------------------ACAGCT-------------------GGCAAA------
AAAGTGACAAAACCCAAC---
GCAAAGCGAACATCCAATGTCCAACCCGGTGATCCCACACCCACAGGCCGTCGTCGCCGATACAAGCCCGGCACCGTCGCATTGAAGGAGATTCGCCGCTACCAACGCTCTTACGACCTCTTGATTGCTAAGCTTCCATTCGCCCGTCTCGTCCGTGAAGTCGCACTCGACCTCCTCCCCGCAGAGGTGGGCGGCACGTTGCGCTGGCAATCCATGGCAATCCAGGCATTGCAGGAGGCTGCAGAAGCTTTCCTGGTGCATTTATTCGAGGATACCAACCTTTGTGCGCTGCATGCGAAGCGAGTTAC
CATTATGCAGAAGGATATTCAGCTTGCGCGCAGAATTCGGGGAGTTTGGGCAGGTCTCGGT
>Penicillium_janthinellum
ATGCCCCCA---ACAGCAAAA------------AAG----------------------------------ACC------------GCC------------GCC------------GCCGGC--GCG---------------CGCAAG---ACA-------------------ATAGCA------AAGAAAGCCGTCGGGAAA------AAAGCCGCA---
----
AATGTACAACCCGGCGACCCAGCCCCGACAGGCCGCAAACGCCGCTACAAGCCCGGCACCGTCGCCCTAAAAGAAATCCGCCGCTACCAACGCTCCTACGACCTTCTCATCGCCAAGCTGCCCTTCGCCCGACTGGTCCGCGAAGTCGCGCTGGACCTGCTCCCCGCCGAAGTTGGCGCCGAGCTCCGGTGGCAGTCGCATGCGATTCAGGCGCTTCAGGAAGCCGCTGAGGCGTTTATGGTGCATTTATTCGAGGACACGAATTTGTGTGCGATTCATGCGAAGCGGGTTACGATTATGCAGAAG
GATATTCAGCTTGCGAGGAGGATTCGCGGGGTTTGGGGGGGATTGGGT
>Penicillium_lanosocoeruleum
ATGCCACCCAAGTTAGCCAGC------------AAG----------------------------------GCA------------GTA------------CCA------------GGCGGC-------------------------GCGCAAAAA-------------------------------AGC---AAAGCGCGCAAGACAGTCGCTGCG--CCGGCTGCC-------------------------GCCGATGGAACACCAACCAAAGCA---------------CAGAAT---ATG-------------------AGGGGA------------------
GGAAAGCAATCAAAAGTCGGAAAAATCACC---
GCGACCGTCCAACCCGGAGATCCAACCCCCACAGGCCGCCGACGCCGATATAAGCCCGGCACCGTCGCTTTGAAGGAAATCCGCCGCTACCAACGCTCATACGACTTACTCATCGCTAAACTTCCCTTTGCGCGCTTAGTCCGCGAAGTCGCGCTCGATCTCCTCCCCGCAGATGTCGGCGCCGAGTTGCGCTGGCAGTCGCATGCCATCCAAGCTCTGCAGGAGGCCGCTGAAGCTTTCCTCGTCCACCTCTTTGAGGATACGAACCTCTGCGCGCTACATGCGAAGCGCGTAACTATTATGCAGAA
GGATATCCAGCTTGCGCGGAGAATTCGCGGTGTCTGGGCTGGGCTTGGT
>Penicillium_raistrickii
ATG------------GCCCGC------------AAA----------------------------------ACC------------GCA------------CCT------------GGAAGC-------------------------GCTCAAAAA-------------------------------GCC---AAAGCTCGCAAGACAGCCGCAACC--CCCGCAGCC------------AACGACGGCGACGGCGACGGGTCCCCA-------------------GTCATCCACAAA---ACA-------------------AAA----------------------GGGAAACAA---
AAAGTCACCAAGACC--
TCCAACGTCCAGCCGGGCGATCCAACCCCCACAGGCCGCCGTCGCCGCTACAAACCCGGTACCGTGGCACTGAAAGAAATCCGCAAATATCAACGATCCTACGACCTGCTTATCGCCAAGCTCCCATTCGCGCGACTGGTGCGCGAAGTCGCACTCGACCTCCTCCCCGCCGATACAGGGTCTGAGCTGCGCTGGCAATCGCATGCAATTCTGGCGCTGCAGGAGGCGGCCGAGGCATTCCTGGTTCATTTGTTCGAGGATACGAATCTGTGCGCTATTCACGCTAAGCGGGTTACTATTATGCAAAA
GGATATTCAGCTCGCGCGGAGAATACGCGGTGTTTGGGCTGGATTGGGC

Saccharomyces – CenH3 alignment before masking unreliable residues inferred in Bali-Phy

```
>S_arb
ATGTCAAGCAAACAGCAATGGGCT---------ATCCAAAGTGATTCGAGT----------------------------------------------------------------------------------------------------GGGAGATCTCTCAGTAATGTCAATAGGCTTGCA---------------------------------------------GGAGAT------------CAACAGTCTATTAACGACCGTGCGCTATCTTTATTGCAAAGGACAAGAGCAAGGAAGAATCTTTTCCCAAGAAGGGAGGAAAAGAGACGATATGAAGGTCCA------------CAGGATGACATAGATTTTGAAGCA-----
------------------------------------------------------------GAGTATGGAAACCAGGCAGGAAATTTGGAAACTGAGACAGAAAATGAGGATGAAACTGAGATGGAAACTGAAGCACCAACTGCCACGATAACTCATTCTTATGCCCTAGATAGGTACGTTAGGCAA------------------------------------------------------AAAAGAAGACAAAAGCAAAGAAGGCAAGACCTGAAG---------------------------------------------------CGCGTCGAA----------------------------------------------------------------
-------------------------------------------------------
AAGAAGTATAGCCCTAGTGAGTTAGCTCTGTATGAAATTCGAAAATACCAGCGTTCTACAGATTTACTTATCTCCAAAATTCCATTCGCAAGGTTAGTGAAGGAAGTTACGGATGAATTTACAACTAAAGACCAAGATTTGCGTTGGCAGTCAATGGCGATCATGGCGTTACAAGAAGCAAGTGAAGCATACTTGGTGGGGTTATTGGAGCACACCAACCTCTTGGCGTTGCATGCGAAAAGAATCACCATAATGAAGAAGGATATGCAACTCGCAAGGAGAATCAGAGGACAGTTTATT
>S_bay
ATGTCTAGCAAACAAGAATGGGCTAACTCGGCCATTCAAAGTGAATCGAGT----------------------------------------------------------------------------------------------------GGAAGATCACTCAGTAATGTCAATAGGCTTGCA---------------------------------------------GGAGAT------------CAACAGTCGATTAATGATCGTGCGTTATCCTTGTTACAAAGAACAAGAGCAAGGAAGAACCTGTTCCCAAGAAGAGAAGAAAGAAGACGGTATGAAGGTTCA------------
CAGGATGACGTAGAATTTGAGGCA----------------------------------------------------------------GATTACGAAGACCATGCAGGAAATCTCGAAACTGAAGCAGAAAACGAAAATGAAACAGAAGTAGAAGAAGAATTATCGACGGCTAAGCAAACTCACTCGTACGCCTTAGATAGATATGTTAGACAA------------------------------------------------------AAAAGAAGACAAAAACAAAGGAAGCAGGGCTTGAAG---------------------------------------------------CGTGTGGAA---------------
----------------------------------------------------------------------------------------------------
AAGAAATATAGCCCTAGCGAATTAGCTCTTTACGAAATTCGAAAATACCAGCGTTCCACAGATTTGCTAATCTCCAAAATCCCATTTGCAAGACTAGTCAAGGAAGTCACGGATGAGTTTACAACCAAAGATCAAGACCTGCGTTGGCAGTCAATGGCGATCATGGCGCTACAGGAAGCAAGTGAAGCATATTTAGTGGGATTATTAGAGCATACCAACCTCTTAGCATTGCATGCGAAAAGGATAACCATAATGAAAAAAGACATGCAACTGGCAAGGAGAATTAGAGGACAATTCATT
>S_cas
ATGTCAAGCAGA-----------------------------------------AAATTTGTAGAACAAGGCCATGCTCAGAATTCTAGTAGTCATCTGTTCTCGAATATCCTCGATAATGAT-----------------------AGTGGATCATTGAGCAATATAAATAGATTAACACTGGACCCGGATAATACAGAAGATTTATTACAGCAAGAAGTC-----------------------------ATCAACGAGCGGGCACTTTCTCTTTTACAAAGGACTAGAGAACGTAGAAACTTATTACACCGATTTGAAGATAAAAGAAGGTAT---------
TATAACCAAGGGCAAGACGACGGAGATTTAGAG---TCTGTGGCCAGTAGTCATTATCGTTCTAATGATGTGGGTGGGAACTTTCAATTTTTTGATCAAGAAGAAGATGAGGATGAGGAAGGTAAC-------------------------------------------------------------------------------------------------------------------GCCATTGATGACGATTATGGAACCCTAGATCAATCCAACATTATCGATCGTCACCAAGAAAGAAGA---------------------------
AAAGGAAACAAACATTCTCGTCAAGAAAGACACCATCAAAGAGAACTCAAACAACGTGTTGAAAAGATTAGAACTCAGAGACAGGTCGGAAATACG--------------------------------------------------------------------------------
AAAAAATTTACTCCAAGTAGTTTAGCCCTATATGAGATAAGGAAATACCAACGATCTTCAGAACTGTTGATATCGAAGATTCCCTTTACTAAATTAGTGAAGGAAGTCACTGATGAATTTACCGTGGAAGACCAACAACTTCATTGGCAATCGATGGCCATTGTCGCGTTACAGGAAGCCAGTGAAGCTTATTTAGTGGGTTTATTGGAGCATGCCAATTTATTAGCCATACATGCCAAGAGGATCACATTAATGAAGAAGGATATACAGTTGGCCAGAAGAATTAGAGGTCAATTCATT
>S_cer
ATGTCAAGTAAACAACAATGGGTTAGTTCTGCTATTCAAAGTGATTCGAGT----------------------------------------------------------------------------------------------------GGAAGATCACTCAGTAACGTCAACAGGCTTGCA---------------------------------------------GGAGAC------------CAACAATCTATTAACGATCGTGCGTTATCGTTATTGCAGAGAACAAGAGCGACAAAGAACCTGTTTCCAAGAAGAGAGGAAAGAAGACGTTATGAAAGCTCA------------
AAAAGTGACCTAGATATCGAAACA----------------------------------------------------------------GACTACGAAGACCAAGCAGGTAATCTAGAAATCGAGACAGAAAATGAAGAAGAAGCTGAAATGGAAACTGAAGTACCTGCACCAGTGCGAACTCATTCATATGCCTTAGACAGATATGTTAGACAG------------------------------------------------------AAAAGGAGGGAAAAACAAAGAAAGCAGAGCTTAAAG---------------------------------------------------CGCGTCGAA----------------
----------------------------------------------------------------------------------------------------
AAGAAATATACTCCTAGTGAATTAGCTCTGTACGAAATTCGAAAATACCAACGTTCCACGGATTTATTAATCTCCAAAATTCCATTTGCAAGGCTAGTGAAAGAAGTTACAGACGAGTTTACAACTAAAGATCAGGATTTACGTTGGCAGTCAATGGCGATTATGGCGTTACAGGAAGCAAGCGAAGCGTATCTGGTAGGATTATTGGAACATACAAACCTCTTGGCGCTGCATGCAAAAAGAATTACTATAATGAAGAAAGACATGCAACTAGCAAGAAGAATCAGGGGACAGTTTATT
>S_klu
ATG---GATAGACAAGAATGG----------------------------------------------------------------------------------------AATGCTAGTATATCCAGCCAGACAGGCAGATCACTCAGTAACACCAATCGTCTG---------------------------------------------GGCGCA---CAAGAGCCCTCTCAACAATCTATCAACGAGAGGGCAATATCGCTTCTACAGCGTAATAGGGCCAGGAGGAAGATGTTACAAAGGCAAGAGGATAGAGAGCGATAC------------------------------------------------------------------
----------------------------------------------------------------------------------------------------------------------------------------------------------------------------------------------------------------------------------------------------------------------------------------------------------------------------------------------------------------------------------------------------
GTAAGGCGTAATGAACTACCAGAGGAACAGCCTCCAACTGCGATAGGTACCATCAGAAGCAATATAAAAAAATCCAAGAGTCAGCGTAAACGATACAAACCAAGTGATGTGGCTTTGCAAGAGATTAGAAAGTACCAACGAAGCACTGAGCTTTTGATATCGAAAATGCCATTTGCCCGTCTCGTAAAAGAGGTCACTGAACAATTTAGTACAGACGAACAGCAGCTCCGGTGGCAGTCTATGGCTATCCTAGCATTACAGGAGGCTAGTGAAGCGTACTTGGTTGGTCTACTGGAGCACACAAAC
CTGTTAGCCTTACACGCTAAAAGAATTACCATTATGAAGAAAGATATGCAGCTGGCAAGGAGAATTAGGGGCCAGTTTATT
>S_kud
ATGTCAAGCAAACAGCAATGGGCTAATTCGGCCATTCAAAGTGATTCGAGT----------------------------------------------------------------------------------------------------GGAAGGTCACTCAGCAATGTGAACAGGCTAGTA---------------------------------------------GAAGAT------------CAACAGTCTATTAATGACCGTGCACTATCTTTGTTACAAAGAACAAGAGCAAGAAAAAATCTTTTTCCAAAAAGAGAAGAAAGAAGACGATATGAAGGTCCA------------
CAAGATGACATGATTTTTGAAGAA----------------------------------------------------------------AACCATGAAGGCCAAGCGGAAAACTTGGAAACAGAGACAGAAAACGAGGATGAACGTGAAATGGAAACAGAGGTTCCAGACGCTACCCGAACACATTCATACGCTCTAGATAGATATGTTAGGCAG------------------------------------------------------AAAAGGAGACAAAAACAAATGAAGCAGGGTTTGAAA---------------------------------------------------CGCGTAGAA----------------
----------------------------------------------------------------------------------------------------
AAGAAATACAGTCCCAGTGAATTAGCTCTGTACGAAATCAGAAAATATCAACGTTCTACAGATTTGCTTATCTCTAAAATTCCATTTGCAAGACTGGTAAAAGAAGTTACAGACGAGTTTACAACTAAAGACCAAGATTTGCGCTGGCAATCGATGGCGATCATGGCGCTACAGGAGGCAAGTGAAGCATACTTGGTGGGGCTATTGGAGCATACCAACCTACTTGCGCTGCATGCGAAGAGAATAACCATAATGAAGAAGGATATGCAACTAGCAAGGAGAATTAGAGGACAGTTCATT
>S_mik
ATGTCAAGCAAACAGCAATGGGCTAATTCTGCTATTCAAAGTGATTCAAGT----------------------------------------------------------------------------------------------------GGAAGATCACTCAGCAATGTCAACAGACTTGTT---------------------------------------------GGAGAG------------CAACAATCCATTAACGATCGTGCATTATCATTATTGCAAAGGACAAGAGCAAGAAAGAATCTATTTCCAAGAAGAGAAGAAAAGAGACGATATGAGAACCCA------------
AAGGATGACGTAGATTTTGAAACT----------------------------------------------------------------GACTATGATGGCCAAGCAGATGATTTGGAGATAGAAACTGAGAACGAAGAAGAAACTGAAGTGGCTACTGAAGTATCAACGTCCGCGCGAACACATTCGTATGCCTTGGACAGATACGTTAGACAG------------------------------------------------------AAAAGAAGAGAAAAGCAAAGGAAACAGGGCTTAAAG---------------------------------------------------CGTATTGAA-----------------
----------------------------------------------------------------------------------------------------
AAGAAGTACAGTCCAAGTGAATTAGCTCTATATGAAATTCGAAAGTACCAACGTTCCACGGATTTATTGATTTCCAAAATTCCTTTTGCAAGGCTTGTAAAAGAAGTTACGGATGAGTTCACGACTAAGGATCAAGATTTACGCTGGCAGTCAATGGCGATCATGGCACTACAAGAAGCAAGTGAAGCCTATTTGGTGGGTCTATTGGAGCATACCAACCTTTTGGCATTGCACGCGAAAAGGATAACTATAATGAAGAAGGACATGCAACTAGCAAGGAGAATCAGAGGACAATTCATT
>S_par
ATGTCAAGTAAACAGCAATGGGCTAGTTCTGCTATTCAAAGTGATTCGAGT----------------------------------------------------------------------------------------------------GGAAGATCACTCAGTAATGTCAACAGGCTTGCA---------------------------------------------GGAGAC------------CAGCAGTCTATTAACGATCGTGCGTTATCGTTACTGCAAAGGACGAGAGCAAGAAAGAACTTACTACCCAGAAGAGAAGAAAGAAGGCGTTATGAGAGCTCA------------
AAAAATGACCTAGATTTTGAAACA----------------------------------------------------------------GACTACGAAGACCAAGCAGGTAATCTGGAAACTGAAACAGAGAATGAAGAAGAAGCTGGAATGAAAACCAAGGCACCAATGTCCGCCCGAACTCATTCGTATGCTTTAGACAGATACGTTAGGCAG------------------------------------------------------AAAAGAAGGGAAAAGCAAAGAAAGCAGGGCTTGAAG---------------------------------------------------CGTGTCGAA----------------
----------------------------------------------------------------------------------------------------
AAGAGATATAGTCCTAGTGAATTAGCTCTGTACGAAATTCGAAAATATCAACGTTCTACAGATTTATTAATTTCCAAAATTCCATTTGCAAGGCTAGTGAAAGAGGTTACAGATGAGTTTACAACCAAAGATCAAGATTTACGTTGGCAGTCAATGGCGATTATGGCGCTGCAGGAGGCAAGTGAAGCATACTTAGTAGGGTTACTGGAACATACCAACCTCTTGGCGTTGCATGCGAAAAGAATAACTATAATGAAGAAAGATATGCAACTAGCAAGAAGAATTAGGGGACAGTTCATT
>S_pas
ATGTCTAGCAAACAAGAATGGGCTAACTCGGCCATTCAAAGTGAATCGAGT----------------------------------------------------------------------------------------------------GGAAGATCACTCAGTAATGTCAACAGGCTTGTA---------------------------------------------GGAGAC------------CAACAATCGATTAATGATCGTGCGTTATCCTTGCTGCAAAGAACAAGAGCAAGGAAGAACCTGTTTCCAAGAAGAGAAGAAAGAAGACGATATGAAGGTCCT------------
CAGGACGACGTAGAATTTGAGGCA----------------------------------------------------------------GATTATGAGGATCATGCAGGAAATCTCGAAACGGAAACAGAAAACGGAGATGAAACTGAAGTAGAAGCAGATGTGTCAACGGTTAAGCAAACTCACTCGTACGCCTTAGATAGATATGTTAGACAA------------------------------------------------------AAACGGAGACAAAAACAAAGGCAGCAGGGCTTGAAG---------------------------------------------------CGTGTGGAA---------------
----------------------------------------------------------------------------------------------------
AAGAAATATAGTCCTAGCGAATTAGCTCTCTACGAAATTCGAAAATACCAGCGTTCTACAGATTTACTAATCTCTAAAATTCCATTTGCAAGATTAGTTAAGGAAGTCACGGATGAGTTTACAACCAAAGATCAGGACTTGCGTTGGCAGTCTATGGCGATCATGGCGCTCCAAGAAGCAAGTGAGGCATATTTGGTGGGATTATTAGAGCATACCAACCTCTTAGCTTTACATGCGAAAAGGATAACCATAATGAAAAAAGACATGCAACTAGCAAGGAGAATTAGAGGACAATTTATT
```

Trichoderma – CenH3 alignment before masking unreliable residues inferred in Bali-Phy

```
>Trichoderma_reesei
ATGCCCTCACCAACCCCAAATCGTCTTCTGCTAACCCCCAACTCTTCAGCG---------------------------------------------------------------------------
GGCGACCCACTGCCTACGAGACAACGACGACGATACCGCCCCGGCACTGTCGCATTGCGGGAAATCAGGCAATACCAGGCCAACACGAAGCTTTTGCTGCTGAAGCTGCCGTTTATGCGCCTCGTTCGAGAAATTGGATTGAATTGTCGTCCGACGGGCAAAGAGTTTAGATGGCAGAGCCAGGCCATCCAGGCGCTGCAAGAAGCCGCAGAGGCCTTCATGGTACATTTGTTTGAGGACGCGCAGCTGTGCGCTGTACACGCCAAGAGGGTCACCCTGATGCAGAAAGACATACAGCTTGCAAG
GAGAATCCGGGGCATCTGGGGCGGCCTTGGC
>Trichoderma_asperellum
ATGGCT--------------------------------------------CCTCGCCAATCAACTTCAGGAACCCAACAACGCTAT---------------
CGTGCCAGACCAAGCGACATTCAGCCGGGCGATCCACTACCTACGAGACAGAAACGTCGATATCGCCCAGGCACCGTTGCGCTACGAGAAATCAGGCAATATCAAGCCAATACAAAGCTTCTACTTCTAAAACTTCCGTTTATGCGACTCGTCCGTGAAATTGGATTAAATTGTCGCCCGAGAGGAAAAGAATTTAGATGGCAGAGCCAGGCAATCCAGGCACTGCAAGAGGCTGCAGAAGCATTTATGGTACATCTATTTGAAGATGCACAGCTATGCGCGGTCCATGCGAAAAGAGTGACACTT
ATGCAAAAAGACATTCAGCTGGCGAGGAGGATTCGAGGTATATGGGGCGGGCTTGGT
>Trichoderma_atroviride
ATGGCT--------------------------------------------CCTCGCCAATCAACGGCAGCGCCCAAACAACGATAT---------------
CGTGCCAGACCAAGCGACATACAGCCGGGCGATCCACTGCCTACGAGACAGAAGCGGCGATATCGCCCGGGCACCGTTGCGCTGCGAGAAATCAGGCAATATCAAGCCAATACAAAGCTTCTACTCCTAAAACTTCCATTTATGCGACTCGTTCGCGAAATTGGATTGAATTGTCGGCCGAGAGGGAAAGATTTTCGATGGCAGAGTCAGGCCATCCAGGCACTGCAAGAGGCCGCAGAAGCATTCATGGTACATCTTTTTGAAGATGCGCAGCTATGCGCGGTCCATGCGAAGAGAGTGACACT
TATGCAAAAAGACATCCAGCTAGCGAGGAGGATTCGAGGTATATGGGGCGGGCTTGGT
>Trichoderma_harzianum
ATGGCT--------------------------------------------CCTCGTCAAAAAACTTCGGCG---------------
GCCTCACAGAAATTGCGCTCCAGACCAAGCGATGTACAGCCGGGCGATCCATTACCTACAAGACAGAAGCGACGATATCGGCCGGGCACCGTTGCGTTGCGAGAGATCAGGCAATACCAGTCAAATACAAAGCTTTTATTGTTAAAACTACCATTCATGCGCCTTGTTCGTGAAATTGGATTGAATTGTCGGCCGAGAGGGAAAGACTTCAGATGGCAGAGTCAGGCTATTCAGGCACTACAAGAGGCCGCGGAGGCCTTTATGGTACATCTTTTTGAGGATGCGCAGCTATGCGCTGTCCACGCA
AAGAGAGTTACACTTATGCAAAAAGATATACAGCTTGCGAGGAGAATCCGAGGCATCTGGGGCGGCCTTGGT
>Trichoderma_longibrachiatum
ATGCCT--------------------------------------------CCTCGTCAGTCCACGGCGGGA---------------
ACCTCACAGAAAGTGCGCTCCCGCCCGAGCGATGTACAGCCGGGCGATCCACTGCCTGCCAGACAGCGACGACGATACCGCCCGGGCACCGTCGCATTGCGGGAAATCAGGCAATACCAGGCCAACACGAAGCTTTTGCTGCTGAAGCTGCCATTTATGCGCCTCGTTCGGGAGATTGGATTGAATTGCCGTCCAAGGGGGAAAGAGTTCAGGTGGCAGAGCCAGGCCATCCAGGCGCTGCAAGAAGCGGCAGAGGCCTTCATGGTACATCTGTTTGAGGATGCGCAGCTGTGCGCTGTGCACG
CCAAGAGGGTTACCCTCATGCAGAAAGACATACAGCTTGCGAGGAGGATTCGAGGCATCTGGGGCGGCCTCGGT
>Trichoderma_virens
ATGGCT--------------------------------------------CCTCGTCAATCAACGTCAGGG---------------
GCTTCACAAAAATTGCGTTCCAGACCAAGCGATGTACAGCCGGGCGATCCATTACCTACGAGACAGAAACGACGATATCGCCCAGGCACCGTTGCGTTGCGAGAAATCAGGCAATACCAGTCAAATACGAAGCTGTTGCTACTAAAACTGCCATTCATGCGCCTTGTTCGTGAAATTGGATTGAATTGTCGCCCGAGAGGAAAAGATTTTAGATGGCAGAGCCAGGCCATTCAGGCGCTACAAGAGGCCGCGGAGGCATTCATGGTACATCTTTTTGAGGATGCGCAGCTGTGCGCTGTCCACGCA
AAGAGAGTTACCCTTATGCAAAAAGACATACAACTGGCGAGGAGAATTCGAGGCATCTGGGGCGGCCTTGGT
```

Bony Fish – CenH3 alignment before masking unreliable residues inferred in Bali-Phy

```
>Esox_lucius
ATGCCCCGCCGCGATCTTGACACTTCCACTGCAAGTCGACGCAAGGGCAAGGCACCAAAGCGTCGGCCTCCAGTCCCAGCCCTGCCCGCTTCA------------------CCACGGCGGAGTGGACCTTTAGCCTCTGCAGGTCCTTCGCACTCTCCC------
CGTAAGAAGAAAAGGTTCCGCCCCGGCACCCGAGCTCTGATGGAGATCCGGAAGTACCAGAAGAGTACCGACTTGCTTCTGCGGAAGGGACCGTTTGCACGCCTGGTTCGGGAGGTGTGCCAGACGTTTAGCAGGGACTACATGAGATGGCAGGTGTATGCCCTACTGGCCTTGCAGGAAGCTGCGGAGGCTTTCCTTGTGTTATTGTTTTCTGATGCTTACCTCTGTACCATCCACGCCAAGCGTGTAACGCTGTTCCCACGAGACATTCAACTTGCGCGACGAATTCGAGGAGCAGATCATCTC
>Haplochromis_burtoni
ATGCGT------------CATAATTCATCTGCCAGCCGTCGGAAGGGTAAAACCCCTCAACGGCGCCCCCAAGTGCCAGCCCAAAGGACCCCCAGTGTAACATCC------CCAAGACGCAGTGGAGTTCCA---------------
GGACAGCCTCCTGTTTCCCCCAAGAAGAGGAAATTTCGACCAGGAACCAGAGCTTTGATGGAGATCCGCAAGTACCAGAAAAGCACAGATCTCCTCCTTAGAAAGGCGCCCTTTGCCCGTTTGGTGCATGAGGTGTGCCAAAGCTTTTCTAGAGAAAATCTTCGATGGCAGGTTTATGCTCTTCTGGCCCTCCAAGAGGCTGCAGAGGCTTTTCTCGTCTTGATATTTTCAGATGCCAACCTGTGTGCCATCCACGCCAAGAGAGTCACTGTATTCCCTCGTGACATTCAGCTAGCCCGGAGGATCCGT
GGGGTGGATAATCTG
>Lates_calcarifer
ATGCGT------------CATGATTCATCTGCAAGTCGACGGAAGGGCAAAACCCCTCAACGTCGCCCCCCGGTACCCGACCCAGGGCCCTCTGGTTCCACACCCAGGACCCCAAGACGAAGCAGAGTTTCA---------------
GGCGTACCTCCTGTGTCTCCCAAGAAAAGAAGGTTTCGACCAGGAACCAAGGCCTTAATGGAAATCCGCAAGTACCAGAAGAGCACCGATCTTCTGTTAAGGAAAGGACCCTTCTCTCGTCTGGTTCGTGAGGTGTGCCAAGGTTTTTCCAGAGAGGCTCTCAGGTGGCAGGTCTATGCTCTTCTTGCCCTGCAGGAGGCTGCTGAGGCCTTTCTTGTCATGTTATTCTCAGACGCCAACCTGTGTGCCATCCATGCCAAGCGGGTAACCGTGTTCCCCCGCGACATTCAATTGGCAAGGAGGATCCG
TGGGGTGGATAACATG
>Micropterus_floridanus
ATGCGT------------CATGATTCATCAGCCAGCCGCCGGAAGGGGAAAACCCCTCAACGTCGCCCCCCGCAGCCGGCCCCAGAGACC---------------CGGTCCCCAAGACGGAGCAGAGTTTCA---------------
AGGCAGCTTCCTGCGTCTCCCAAGAAGAGGAGGTTTCGACCAGGAACCAAGGCCTTAATGGAGATCCGCAAGTTCCAGAAGAGCACTGATCTTTTGCTCAGGAAAGGACCCTTCTCTCGCCTGGTTCGTGAGGTATGCCAGAGTTTTTCCAGAGAGGCTCTCCGGTGGCAGGTCTACGCACTTCTGGCCCTGCAGGAGGCTGCAGAGGCGTTTCTCGTCATGCTATTCTCAGACGCAAATCTGTGTGCCATCCACGCCAAGCGGGTCACCGTGTTCCCCCGTGATATTCAGCTGGCCAGGAGGATCC
GCGGCGTTGATAACCTG
>Misgurnus_anguillicaudatus
ATGCGG------------CACAATAAATCAGCACATAAGCGAAAGCCATCGACCCCCAGGCGCAGATCCCCT---CCTGCTACACCAGCAGCACCCTCCACATCCAGAACCCGACGCACCAGTGGACCATCC---------------GAGAAATCCCCA------
CGCAAGAAGCACAAGTTTCGACCTGGGACGCGAGCTCTCATGGAGATACGCAAATATCAGAAATCCACAGATCTGTTGTTGCGCAAGGGCCCGTTTTCACGACTGGTACGTGAGGTGTGTCAGACATTTAGCCGGGAGAACTTAATGTGGCAGGGCTATGCACTAATGGCTTTACAAGAGGCTGCAGAGGCGTTCATGGTCCGTTTGTTCTCTGATGCCAATCTCTGTGCTATTCATGCCAAGAGGGTGACATTGTTTCCACGTGATATACAGCTTGCCCGTCGAATCAGAGGTGTTGAAAACATG
>Neolamprologus_brichardi
ATGCGT------------CATAATATATCTGCCGGCCGTCGGAAGGGTAAAACCCCTCAACGGCGTCCCCAAGTGCCAGCCCAAAGCACCTCCAGTGTAACATCC------CCAAGACGCAGTGGAGTTCCA---------------
GGACTGCCTCCTGTTTCCCCCAAGAAGAGGAAATTTCGACCAGGAACCAGAGCTTTGATGGAGATCCGCAAGTACCAGAAAAGCACAGATCTCCTCCTTAGAAAGGCGCCCTTTGCCCGTTTGGTTCATGAGGTGTGCGAAAGCTTTTCTAGAGAAAGGCTCCGATGGCAGGTTTATGCTCTTCTGGCCCTCCAAGAGGCTGCAGAGGCTTTTCTCGTCTTGATATTTTCAGATGCCAACCTGTGTGCCATACACGCCAAGAGAGTCACTGTATTCCCTCGTGACATTCAGCTAGCCCGGAGGATCCGT
GGGGTGGATAATATG
>Nothobranchius_furzeri
ATGCGT------------CACAGTTCACCGCGCAGCCGGCGGAAGGCCAAAACTCCCAGTCGTCGTGACCCACCTCCATCTCCCACAGCATCATTCTCTTCA------ACACCAAGAAAAAGTGGTTTTTCT---------------
GGGATTCCTCCTGTGTCTCCCAAGAAAAGGAGGTACAGACCTGGAACCAGAGCCTTGATGGAGATTCGTAAGTACCAGAAGAGTACCGATCTTCTGCTCAGGAAAGCACCATTCGCTCGCTTGGTTCACGAGGTGTGCCAGAGTTATACCAGAGGCAATTTGCGGTGGCAGGTCTTCGCTCTTCTTGCCCTGCAGGAGGCAGCAGAGGCCTTCCTAGTCCTGCTGTTTTCAGATGCCAACCTGTGTGCCATTCATGCTAAGAGGGTGACTGTGTTTCCTCGGGACATTCAGCTGGCCAGGAGGATCC
GTGGAGTGGATCGTCTG
>Oncorhynchus_mykiss
ATGCCTCGTCACGATCCTGACAGTTCTTCTGCAAGCCGGCGCAAGGGTGCGGTACCCAAGCGTCGGCCTCCAGCCTCCACCGCGACTGCTTCAACATCAAAGGCAAAGGCACCACGGCTGAGTGGACCATCGGCCTCTGCTGTTCCAGATCCCCCCCCG------
CGAAAAAATAGAAGGTTTCGCCCTGGCACCCGAGCTCTGATGGAAATTCGCAAGTACCAGAAGAGCACTGACTTGCTTTTGCGCAAGGCACCGTTTGCACGTCTGGTTCGGGAGGTGTGCCATACTTATACCAGGGACTTCATGAGATGGCAGGTGAACGCTCTTCTGGCCTTGCAGGAGGCTGCAGAGGCTTTCCTTGTTTTACTGTTTTCCGACGCCTACCTGTGTACAATCCACGCCAAGCGTGTAACGCTGTTCCCCCGTGACATTCAGCTTGCCCGGCGAATTCGAGGAGTGGATGACCTC
>Ophthalmotilapia_ventralis
ATGCGT------------CATAATCCATCTGCCAGCCGTCGGAAGGGTAAAACCCCTCAACGGCGCCCCCAAGTGCCAGCCCAAAGGACCTCCAGTGTAACGTCC------CCAAGACGCAGTGGAGTTTCA---------------
GGACAGCCTCCTGTTTCCCCCAAGAAGAGGAAATTTCGACCGGGAACCAGAGCTTTGATGGAGATCCGCAAGTACCAGAAAAGCACAGATCTCCTCCTTAGAAAGGCGCCCTTTGCCCGTTTGGTTCATGAGGTGTGCCAAAGCTTTTCTAGAGAAAGGCTCCGATGGCAGGTTTATGCTCTTCTGGCCCTCCAAGAGGCTGCAGAGGCTTTTCTCGTCTTGATGTTTTCAGATGCCAACCTGTGTGCCATCCACGCCAAGAGAGTCACTCTATTCCCTCGTGACATTCAGCTAGCCCGGAGGATCCGT
GGGGTGGATAATCTG
>Oreochromis_niloticus
ATGCGT------------CATAATTCATCTGCCAGCCGTAGGAAGGGTAAAACCCCTCAACGGCGCCCCCAAGTGCCAGCCCAAAGGACCTCCAGTGTAACATCC------CCAAGACGCAGTGGAGTTCCA---------------
GGACAGCCTCCTGTTTCCCCCAAGAAGAGGAAATTTCGACCAGGAACCAGAGCTTTGATGGAGATCCGCAAGTACCAGAAAAGCACAGATCTCCTCCTTAGAAAGGCGCCCTTTGCCCGTTTGGTTCATGAGGTGTGCCAAAGCTTTTCTAAAGAAAGGCTCCGATGGCAGGTTTATGCTCTTCTGGCCCTCCAAGAGGCTGCAGAGGCTTTTCTCGTCTTGATATTTTCAGATGCCAACCTGTGTGCCATCCACGCCAAGAGAGTCACTGTATTCCCTCGTGACATTCAGCTAGCCCGGAGGATCCGT
GGGGTGGATAATCTG
>Salmo_salar
ATGCCCCGTCGCGATCCTGACGTTTCTTCTGCAAGCCGGCGCAAGGGTGCAGTACCCAAGCGTCGGCCTCCAGCCTCCACCGCGACTGCTTCAACATCAAAGGCAAAGGCACCACGGCTGAGCGGACCATCGGCCCCTGCAGGTCCAGCTCCCTCCCCG------
CAAAAAAAGAGAAGGTTTCGCCCTGGCAACCGAGCTCTGCTGGAAATTCGCAAGTACCAGAAAAGCACTGACTTGCTTTTGCGCAAGGGACCGTTTGCACGCCTGGTTCGGGAGGTGTGCCAGACTTATAGCAGGGACTTCATGAGATGGCAGGTGAACGCTCTTCTGGCCTTGCAGGAGGCTGCAGAGGCTTTCCTCGTTTTACTGTTTTCCGACGCCTACCTGTGTACCATCCACGCCAAGCGTGTAACGCTGTTCCCCCGTGACATTCAGCTTGCCCGGCGAATTCGAGGAGTGGATGATCTC
```

Drosophila – CenH3 alignment before masking unreliable residues inferred in Bali-Phy

>mimetica
ATGCCGAGAAAAAGTGGCGCAAGCCGCAAGATGCCCGTTCCC-----------------AAGCCAACGCTGGGGGACACGGACGCGGAG-----------------AGCGACGACGACACGGCTTTCCGATCGCCGGAACCGGAAGATGGCACCGACTACGGCCTGGAGTTCACCACAAGCCGACTGACTCTACAGGATGCC---TCCAATCGCCGTTGCTCAACGATGCGCAAGGACACCCGAAACACCCGGGCATCGCGA---------------GCGGCGGATAGAACCGCCTCAAGC---GAAGAG---GAGGAT------
CAGGAGAATCGCCTTCCTGCAGCCAGATCGCCGCAGACGCGACAGATGACCAGTCAGCAGGAGAGGCGACAGGCATCC--------------------------AGCCAGCAG--------------------------GTCAGGGTCCAAGCATCT-----------GGA------GCC---GGACAA---GCTGTCCAG------AATCAA------
ACCAGGCGACGTAAGATGGCCAATCCCATGAGCCGAGCCCAGAGAATGGATCGCGAGATCCGACATCTGCAGAATCATCCCGGCATACTCATCCCTAAGCTGCCATTCTCGCGCCTGGTTCGCGAATTTATCATGAAGTACAGCGACGGAGCTCCGTTGAAGGTCACTGAAGGCGCCCTCAACGCCATGCAGGTGTCCAGCGAGTTGTATGTGACGCAACGTCTGGCCGACTTCTACATGCTAACCAAACACCGCAATCGCGTCACGCTGGAAGTGCGCGACATGGCTTTGATGGCTCTGATCTGTG
ACCGGAGTCATAAC------------------
>takahashii
ATGCCGAGAAAAAGTGGCGCAAGCCGCAAGGTGGACGTGCCC-----------------AAGCCGACGCTGGGTGACACGGACGCGGAG-----------------AGCGACGACGACTCGGCCTTCCGATCGCCGGAACCGGAGGACGGCACCGACTACGGCTTGGAGTTCACCACCAGCCGGCTGACTCTGCAGGACGCC---TCCAATCGCCGTTGCTCGACGCTGCGCAAGGACGCCAGAAGTGGCCGGGCATTGGAA--------------GCAGCGAATAGAACAGCCTCCAGC---GAGGAG---GAGGAT------
CAGGAGAATCGACTGCCAGCAGCCAGATCGCCG------CGACGGATGACCAGTCAGCAGGAAAGGCGTCCAACTGCC--------------------------AGCCAGCAGGAGAGGCGTCCAACTGCCAAC--------------CAAGCATCT-----------GGC------TTA---GGACCAGCTGCTCCTCAG------GATCGT------
AGGCGGCCACGCAAGATGGCCAATCCCATGAGCAGAGCCCGGCGAATGGATCGCGAGATCCAACAACTGCAGAAACATCCCGGCACGCTTATACCCAAGCTGCCATTCTCGCGCTTGGTTCGCGAATGTATCATGAAGTACAGCGACGGAGCACCGCTGAAGATCACCGAGGGCGCCTTCAACGCCCTGCAGGTGTCCAGCGAGATGTACGTGACGCAGCGCCTGGCCGACTCCTACATGCTGACCAAGCACCGCAATCGCGTCACGCTGGAGGTGCGCGACATGGCTCTGATGGCTCTGATCTG
CGACCGGGGTCACAAC------------------
>lutescens
ATGCCGAGAAAAAGTGGGGCAAACCGCAAGGTGAACGTGCCC-----------------AAGCCAACGCTGGGGGACACGGACGCGGGG-----------------AGCGACGACGACACGGCCTTCCGATCGCCCGAACCGGAAGATGGCACCGACTACGGCTTGGAGTTCACCACAAGCCGGCTGACTCTGCAGGACTCC---TCCAATCGCCGTTGCTCCACGCTGCGCAAGGACACCAGAAATGACAGG-----------------------GCAGCGGATGGAACCGCCTCCAGC---GAAGAG---GAGGAT------
CAGGAGAATCGCCTGCCAGCAGCCAGATCGCCGCAGACGGGACGGATGACCAGTCAGCAGGAGAGGCGTCCTGCTCCCAGCCAGCAAGAGAGGCGTCCTGCTCCCAGCCAGCAAGAGAGGCGTCCGGCTCCCAGCCAGCCGGTTAGGACTCAAGCATCC------------GGA------ACC---GGACCAGCTGCTGGCCAG------GATCGG------
CACCGCCGACGCAAGATGGCCAACCCCATAAGCAGAGCCAAGCGAATGGATCGCGAGATCCAAAATCTGCAGAAACATCCCGGCACGCTTATACCCAAGCTGCCGTTCTCGCGTCTGGTTCGCGAATTCATCGTCAAGTACAGCGACGGAGAACCGTTTAGGGTCACCGAAGGCGCCTTCAACGCCCTGCAGGTGTCCAGCGAGATGTACGTGACGCAGCGCCTGGCCGACTCCTACATGCTGACCAAGCACCGCAATCGCGTCACGCTGGAGGTGCGCGACATGGCTCTGATGGCTTTGATCTGC
GACCGGGGTCACAAC------------------
>trilutea
--GAAGAG---GAGGAT------CAGGAGAATCGCCTGCCACCAGCCAGATCGCCGCAGACGCGACGGATGACCAGTCAGCAGGAGAGGCGACCGGCTCCC--------------------------
AGCCAGCAGGAGAGGCGTCCGGCTTCGAGCCAGCCGGTTAGCAACCAAGCATCC------------GGA------ACC---GGACCAGCTGCTGGCCAG------GATCGG------
AAACGGCCACGCAAGATGGCCAATCCCAGAAGCAGAGCCCAGCGAATGGATCGCGAGATCCAACGTCTGCAGAAACATCCCGGCACGCTTATACCCAAGTTGCCATTCTCGCGTTTGGTTCGCGAATTTATCGTCAAGTACAGCGACGGAGAACCGTTTAGGGTCACCGAAGGCGCCTTCAACGCCATGCAGGTGTCCAGCGAGATGTACGTGACTCAGCGTCTGGCCGACTCCTACATGCTGACCAAACACCGCAATCGCGTCACGCTGGAGGTGCGCGACATGGCTCTGATGGCTTTGATCTGC
GACCGGGGTCATAAC------------------
>paralutea
--TACGGCTTGGAGTTCACCACAAGCCGGCTGACTCTGCAGGACGCC---TCCAATCGCCGTTGCTCGACGCTGCGCAAGGACACCAGAAATGGCCGGGCATTGGAA--------------GCAGCGGATAGAACCGCCTCCAGC---GAAGAG---GAGGAT------
CAGGAGAATCGCCTGCCAGCAGCCAGATCGCCGCAGACGCGACGGATGACCAGCCAGCAGGAGAGGCGTCCGGCTCCC--------------------------AGCCAGCAGGAGAGGCGTCCGGCTCCCATCCAGCCGGTTAGGGTTCAAGCATCC------------GGA------ACC---GGACCAGCGGCTGGCCAG------GATCGG------
AAACGGCGACGCAAGATGGCCAATCCCATAAGCAGAGCCAAGCGAATGGATCGCGAGATCCAACGTCTGCAGAAACATCCCGGCACGCTTATACCCAAGCTGCCATTCTCGCGTCTGGTTCGCGAATTTATAGTCAAGTACAGCGATGGACCACCGTTCAGGGTCACCGAAGGCGCCTTCAACGCCATGCATGTGTCCAGCGAGATGTACGTGACGCAGCGTCTGGCCGACTCCTATATGCTGACCAAACACCGCAATCGCGTCACGCTGGAGGTGCGCGACATGGCTCTGATGGCTTTGATCTGC
GACCGGGGTCATAAC------------------
>lucipennis
--TACGGCTTGGAGTTCACCACCAGCCGCCTGACGCTGCAAGAGTCC---TCCAATCGCCGCTGCTCCACGATGCGCAAAGACCCC---GCCGGCAGAAGATCACGT--------------CCACCGGCTACGGCATCGTCGAGC---GACGAG---GAGGAT------
CAGGAAAACCACCAACCGGCAGCGAGATCGCCGCAGACCCGTCGAATGACAAGTCTTCAGGGGAGTCGCCAGGCTCCA--------------------------GCCCAGCAA-------------------------GCCAGGACCCAGGCCTCA-----------GCGTCAGGTTCC---AGATCAGCCGCTTTCCAGCAGCAGCAGCAG------CCCCGCCGCCGCAAGGCCGTCAATCCCATGAGA---------AGGATGGAGCGCGAAATCAGGCGGCTACAGGCCCATCCCGGC--
----
>prostipennis
ATGCCGAGAAAAAGTGGCGCAAGCCGCAGGGTGAACGTGCCC-----------------AAGCCAACGCTGGGGGACACGGACGCGGAG-----------------AGCGACGACGACACGGCCTTCCGATCGCCAGAACCGGAAGATGGCACCGACTACGGCTTGGAGTTCACCACAAGCCGGCTGACTCTGCAGGACGCC---TCCAATCGCCGTTGCTCGACTCTGCGCAAGGACACTAGAAATGGCCGGGCATTGGGA--------------GCAGCCGACAGAACCGCCTCCAGC---GAAGAG---GAGGAC------
CAGGAGAATCGCCTGCCAGCAGCCAGATCGCTGCAGTCGAGTCGCATAACCAGTCAGCAGGAGATGCGTCCTGCTCCC--------------------------AGCCAGCAG-------------------------GTCAGGACTCAGGCATCT-----------GGA------TCA---GGACAAGCTGCTCCTCAG------GATCGG------AACCGGCGACGCAAGATGGCCAATCCCATAAGCAGAGCCCAGCGAATGGATCGCGAGATCCAAAATCTGCAGAAACATCCCGGC--
----
>rajasekari
---GACTACGGCTTGGAGTTCACCACAAGCCGGCTCACCCTG---------AATTCCAACCGCCGATGCTCGACGCTGCACAAGGAGACCTCAGGCGGCCCTGGGTCGCGA--------------GCAGCGGGAAGAACT---TTCAGC---GATGAG---GAGGAT------CAGGAGAATCGG---
ACGCCAGCCAGCTCGCCGGGGACGCGCAGGATGAGCGGTCAGCAGGAAAGGCGCCTGGCTCCC-------------------------AGCCAGCCG-------------------------TCCAGGACTCAGGCAGCG-----------GCA------AAC---GGATCGATTGGTGCCCAG------AATCAG------ACCAGGAGACGCAAGATGGCCAAACCCTTGAGCAGAGCCAGAAGGATGGATCTAGAGATCCAGCATCTGCAGAATCATCCCGGC--
----
>mauritiana
ATGCCACGACACAGTAGAGCCAAACGC---------GCACCC-----------------AGGCCGTCGGCG-----------------AACAACTCAAAGTCGCCGAACGACGACGACACGGCCTTCCACTCGCCGGAGCCAGAAGACGGCACCGACTACGGCCTCGAGTTCACCACCAGCCAACTGACGCTTCAGGAA------AGCAATCGGCGTTCCTCGACGATGCGCAGGGACGCC--------------------GGACGGAGGCAGTCGCCAACGAATGGTAGCTCCGCCAGTGGCGAGGAG---GAGGAC------
CAGGAGAATCGCCATCCCAGAGCCAGACCGTCGCAGACGCGTCGATTGACCGTGCAGCAGGAA--AGCAAGACGCGGGCA---------GCGGGGCCAGTTGCTTGCCAA------AACCAG------
ACCAGGCGGCGCAAAGCGGCCAATCCCATGAGCAGAGCCAAGAGGATGGATCGCGAGATCCGGCGACTGCAGCACCATCCCGGCACACTGATACCCAAGCTGCCGTTCTCGCGTCTGGTGCGGGAGTTTATCATGAAGTACAGCGACGGCGAGCCGCTGAGGGTCACCGAGGGCGCCCTATTGGCCATGCAGGAGTCCTGCGAGATGTACTTGACGCAGCGGCTCGCGGACTCCTACATGCTAACCAAGCATCGCAATCGCGTCACACTGGAGGTGCGCGACACGGCATTGATGGCCTACATCT
GCGACCGGGGT------CGGCAAGCT---------
>simulans
ATGCCACGACACAGTAGAGCCAAGCGC---------GCACCC-----------------AGGCCGTCGGCG-----------------AACAACTCAAAGTCGCCGAACGACGATGACACAGCCTTCCACTCGCCGGAGCCAGAAGACGGCACCGACTACGGCCTCGAGTTCACCACCAGCCAACTGACGCTTCAGGAA------AACAATCGGCGTTCCTCGACGATGCGCAGGGACGCC--------------------GGACGGAGGCAGTCGCCAACGAATGGTAGCTCCGACAGTGGCGAGGAG---GAGGAC------
CAGGAGAATCGCCATCCCAGAGCCAGACCGTCGCAGACGCGTCGATTGACCGTGCAGCAGGAA--AGCAAGACGCGGGCA---------GCAGGGCCAGTTGCTGCGCAG------AACCAG------
ACCAGGCGGCGCAAAGCGGCCAATCCCATGAGCAGAGCCAAGAGGATGGATCGCGAGATCCGGCGACTGCAGCACCATCCCGGCACACTGATACCCAAGCTGCCGTTCTCGCGTCTGGTGCGGGAGTTTATCATGAAGTACAGCGACGGCGAGCCGCTGAGGGTCACCGAGGGCGCCCTGTTGGCCATGCAAGAGTCCTGCGAAATGTACTTGACGCAGCGGCTCGCGGACTCCTACATGCTAACCAAGCATCGCAATCGCGTCACACTGGAGGTGCGCGACATGGCATTGATGGCCTACATCT
GCGACCGGGGT------CGGCAAGCT---------
>sechellia
ATGCCACGACACAGTAGAGTCAAGCGC---------GCACCC-----------------AGGCCGTCGGCG-----------------AACAACTCAAAGTCGCCGAACGACGATGACACAGCCTTCCGCTCGCCGGAGCCAGAAGACGGCACCGACTACGGCCTCGAGTTCACCACCAGCCAACTGACGCTTCAGGAA------AACAATCGGCGTTCCTCGACGATGCGCAGGGACGCC--------------------GGACGGAGGCAGTCGCCAACGAATGGTAGCTCCGCCAGTGGCGAGGAA---GAGGAC------
CAGGAGAATCGCCATCCCAGAGCCAGACCGTCGCAGACGCGTCGATTGACCGTGCAGCAGGAA--AGCAAGACGCGGGCA---------GCAGGGCCAGTTGCTGCGCAG------AACCAG------
ACCAGGCGGCGCAAAGCGGCCAATCCCATGAGCAGAGCCAAGAGGATGGATCGCGAGATCCGGCGACTGCAGCACCATCCCGGCACACTGATACCCAAGCTGCCGTTCTCGCGTCTGGTGCGGGAGTTTATCATGAAGTACAGCGACGGCGAGCCGCTGAGGGTCACCGAGGGCGCCCTAATGGCCATGCAAGAGTCCTGCGAAATGTACTTGACGCAGCGGCTCGCGGACTCCTACATGCTAACCAAGCATCGCAATCGCGTCACACTGGAGGTGCGCGACATGGCATTGATGGCCTACATCT
GCGACCGGGGT------CGGCAAGCT---------
>melanogaster
ATGCCACGACACAGCAGAGCCAAGCGC---------GCACCC-----------------AGGCCGTCGGCG-----------------AACAACTCAAAGTCGCCGAACGACGACGACACGGCCTTCCGCTCGCCGGAGCCAGAAGACGGCACCGACTACGGCCTCGAATTCACCACCAGCCAACTGACGCTTCAGGAC------AACAATCGGCGTTCCTCGACGTTGCGCAGGGACGCC--------------------GGACGGAGGCAGCCGGCAGCGAGAGACAGCTCCACCAGTGGCGAGGAG---GAGGAC------
CAGGAGAATCGCTATCCCACAACCAGATCGCCGCAGACGCGTCGAATGACCGTGCAGCAGGAA--AGCAAAACGCGAGCA---------GCAGGGCCAGTTGCTGCGCAA------AACCAG------
ACCAGGCGGCGCAAAGCGGCCAATCCCATGAGCAGAGCCAAGAGAATGGATCGTGAGATCCGGCGACTGCAGCACCATCCCGGCACACTGATACCCAAGCTGCCGTTCTCGCGTCTAGTGCGCGAGTTCATCGTGAAGTACAGCGACGACGAGCCGCTAAGGGTCACCGAAGGCGCCCTATTGGCCATGCAGGAGTCGTGCGAGATGTACTTGACGCAGCGGCTCGCCGACTCCTACATGCTAACCAAGCATCGCAATCGCGTCACACTGGAGGTGCGCGACATGGCATTGATGGCCTACATCT
GCGACCGGGGT------CGGCAATTT---------
>yakuba
ATGCCACGACACGGGAACGCCAAGCGC---------GGCCCC-----------------AAATCGACGGTG-----------------AACAATTCAAGGCCGCAAACCGACGACGACACATGCTTCCGCTCGCCGGAACCAGAAGACGGCACCGACTACGGCCTGGAATTTACCACCAGCCAACTGACGCTACGGGAGGAC---AACAGTCGTCGTTCCTCGACGATGCGCAAGGACGCC--------------------GAACGGAGGCATCCGCCAGCGAATAGGACCTCCACCAGCGACGAGGGG---
GATGATGAGGATCAGGAAAATCGCGGTCCGGCAGCCGCGTCGTCGCAGTCGCGTCGGATGACCGTGCGCCCAGAA--ACCAGCACGCGGTCG---------CCGAGACCAGTTGCCGCACAG------
AACCAGAACCAAACCAGGCAACGCAAAATGCCCAATCCCATAAGCAGGACCAATAGGTTGAATCGCGAGATACTGCGGCTGCAGAACCATCCCGGCACACTTATACCCAAGCTGACGTTTGCGCGTCTGGTGCGCGAGTTCATCGTGAAGTACAGCGATGAAGCACCGTTGAGGGTCACCGAGGGCGCGTTAATTGCCATGCAGGAGTCCTGCGAGATGTTCTTGACGCAGCGGCTCGAGGACTCCTACATGCTAACCAAGCATCGCAATCGCGTCACACTGGAGGTGCGTGACATGGCCCTGAT
GGCCTACATCTGCGACCGAAAT------CGG------TCGTCAAAT
>teissieri
ATGCCTCGACACAGTAACGCCAAGCGC---------GGCCCC-----------------AAATCGACGGTG-----------------AACAACTCAAAACCGCCAAGCGACGACGACACAGCCTTCCGCTCGCCGGAGCCAGAAGACGGCACCGACTATGGCCTGGAGTTCACCACCAGCCAACTGACGCTACAGGAG------AACAGTCGTCGTTGCTCGACGATGCGCAAGGACGCC--------------------GGACGGCGGCATCCGGCGGCGAGTAGGATGTCCACCAGCGACGAGGAG---
GAGGATCAGGATCAGGAAAATCGCCATCCGGCAGCCAGATCACCGCAGACGCGTCGGATGACCGCGCACCAAGAA--ACCAGCACGCGGGCG---------CCGAGACCAGTTGCTGCACAG------
AACCAGAACCAAAACAGGCGGCGCAAAATGCCCAATCCCATAAGCAGAACGAAAAGAATGGATCGCGAGATACGGCGACTGCAGAACCATCCCGGCACACTAATACCCAAGCTGCCGTTCTCGCGTCTGGTGCGCGAGTTCATCGTGAAGTACAGCGATGGAGAACCGCTGAGGGTCACCGAGGGCGCCTTTATGGCCATGCAGGAGTCCTGCGAGATGTACCTGACGCAGCGGCTCGCGGACTCCTACATGCTAACCAAGCATCGCAATCGCGTTACACTGGAGGTGCGCGACATGGCCCTGA
TGGCCTACATCTGCGACCGGGGT------CGGCTAGCA---------
>orena
ATGCCTCGACACAGTGATGCCAAGCGT---------AACCCTAAATCGAACCCCAAATCGAACCCCAAAATG-----------------AACAACTCACAGCCGCCAAGCGACGACGACACAGCATTCCGCTCGCCGGCTCCAGAAGGCGACACCGACTACGGCCTGGAGTTCACCACTAGCGAACTGACGCTACAGGAGGGC---AACAATCGTCGTTGCTCCACGATGCGCAAGGACGCC--------------------GGACATAGGCATCAGCCAGCGACCCGCACCTACTCCAGCAGCGACGAG---GAGAAC------
CAGGAGAATCGCCATCCGGCAGCCACATCCAAGCAGACGCATCCGAAA--
CGGCGGCGCAAAATGAACAATCCCATAAGCAGAGTCAAGAGGGTGAACCTCGAGATACGGCGATTGCAAAGCCATTCCCGCGCACTGATACCTAAGCTGCCCTTCTCGCGTCTGGTGCGCGAATTCATCATGAAGTACAGCGATGGGGAGCCGTTGAAGATCAGCGTGGCAGCCTTAGTGGCCATACAGGAGTCCTGCGAGCTGTACGTGACACAGCGGCTGTCGGACTCCTACATGCTAACCAGGCATCGCAATCGCGTCACACTTGAGGTGCGCGACATGGCTCTGATGGCCTACATCTGCGA
GCGGGCT------CGGCTAGCC---------
>erecta
ATGCCCCGGCACAATGCTGCCAAGCGT---------AACCCC-----------------AACCCATCAATG-----------------AACAACTCAAAGCCTCCAAGCGACGACGACAAAGCATTCCGCTCGCCGGAGCCAGAAGACGACACCGACTACGGCCTGGAGTTCACCACCAGCCAACTGACGCTACAGGAGGGC---TACAATCGTCGTTGCTCCACGATGCGCAAT--------------------------------------CAGCCAGCGACCCGCACTTACACCAGCAGCGACGAGCAGGAGGAC------
CAGGAGAATCGCCATCCGGCAGCCAGATCAAAGCAGACGCGTCCGACACCCGTGCGCCAAGAA--ACTAGCTCGCGGGAA---------GCGGGACCATTTGCTGCACAG------AACCAG------
ACCAGGCGGCGCAAAATGGCCAATCCCATAAGCAGAGCCAAGCGGATGGATCTCGAGATACGGCGACTGCAGAACCATTCCGGCACACTGATACCCAAGCTGCCGTTCTCGCGTCTGGTGCGCGAGTTCATCGTGAAGTACAGCGATGGAGAGCCGTTGAGGGTCTCCGAGGGCGCCTTTATGGCCATGCAGGAGTCCTGCGAGATGTACGTGACGCAGCGGCTGTCGGACTCCTACATGCTAACCCGGCATCGCAATCGCGTGACACTGGAGGTGCGCGACATGGCTCTGATGGCCTACCTTTG
CGACCGGGGT------CGGCTGGCC---------

Primates – CenH3 alignment before masking unreliable residues inferred in Bali-Phy

```
>Human
ATGGGCCCGCGCCGCCGGAGCCGAAAGCCCGAGGCCCCGAGGAGGCGCAGCCCGAGCCCGACCCCGACCCCCGGCCCCTCCCGGCGGGGCCCCTCCTTAGGCGCTTCCTCCCATCAACACAGTCGGCGGAGACAAGGTTGGCTAAAGGAGATCCGAAAGCTTCAGAAGAGCACACACCTCTTGATAAGGAAGCTGCCCTTCAGCCGCCTGGCAAGAGAAATATGTGTTAAATTCACTCGTGGTGTGGACTTCAATTGGCAAGCCCAGGCCCTATTGGCCCTACAAGAGGCAGCAGAAGCATTTCT
AGTTCATCTCTTTGAGGACGCCTATCTCCTCACCTTACATGCAGGCCGAGTTACTCTCTTCCCAAAGGATGTGCAACTGGCCCGGAGGATCCGGGGCCTTGAGGAGGGACTCGGC
>Pan
ATGGGCCCGCGCCGCCGGAGCCGCAAGCCCGAGGCCCCGAGGAGGCGCAGCCCGAGCCCG------
ACCCCCGGCCCCTCCCGGCGGGGCCCCTCCTTAGGCGCTTCCTCCCATCAACACAGTCGGCGGAGACAAGGTTGGCTAAAGGAGATCCGAAAGCTTCAGAAGAGCACACACCTCTTGATAAGGAAGCTCCCCTTCAGCCGCCTGGCAAGAGAAATATGTGTTAAATTCACTCGTGGTGTGGACTTCAATTGGCAAGCCCAGGCCTTATTGGCCCTACAAGAGGCAGCAGAAGCATTTCTAGTTCATCTCTTTGAGGACGCCTATCTCCTCACCTTACATGCTGGCCGAGTTACTCTCTTCCCAAAGGAT
GTGCAACTGGCCCGGAGGATCCGAGGCCTTGAGGAGGGACTTGGC
>Gorilla
ATGGGCCCGCGCCGCCGGAGCCGAAAGCCCGAGGCCCCGAGGAGGCGCAGCCCGAGCCCGACCCCGACCCCCGGCCCCTCCCGGCGGGGCCCCTCCTTAGGCGCTTCCTCCCATCAACACAGTCGGCGGAGACAAGGTTGGCTAAAGGAGATCCGAAAGCTTCAGAAGAGCACACACCTCTTGATAAGGAAGCTCCCCTTCAGCCGCCTGGCAAGAGAAATATCTGTTAAATTCACTCGTGGTGTGGACTTCAATTGGCAAGCCCAGGCCCTATTGGCCCTACAAGAGGCAGCAGAAGCATTTCT
AGTTCATCTCTTTGAGGACGCCTATCTTCTCACCTTACATGCTGGCCGAGTTACTCTCTTCCCAAAGGATGTGCAACTGGCCCGGAGGATCCGGGGCCTTGAGGAGGGACTCGGC
>Pongo
ATGGGCCCGCGCCGCCGGAGCCGCAAGCCCGAGGCCCCGAGGAGGCGCAGCCCGAGCCCGACCCCGACCCCCGGCCCCTCCCGGCGGGGCCCCTCCTTAGGCGCTTCCTCCCATCAACACAGTCGGCGGAGACAAGCTTGGCTAAAGGAGATCCGAAAGCTTCAGAAGAGCACACACCTCTTGATAAGGAAGCTCCCCTTCAGCCGCCTGGCAAGAGAAATATGTGTTAAATTCACTCGTGGTGTGGACTTCAATTGGCAAGCCCAGGCCCTATTGGCCCTACAAGAGGCAGCAGAAGCATTTCT
AGTTCATCTCTTTGAGGACGCCTATCTCCTCACCTTACATGCCGGCCGAGTTACTCTCTTCCCAAAGGATGTGCAACTGGCCCGGAGGATCCGGGGCCTTGAGGAGGGACTCGGC
>Nomascus
ATGGGCCCGCGCCGCCGGATCCGCAAGCCCGAGGCCCCGAGGAGGCGCAGCCCGAGTCCG------
ACCCCCGGCCCCTCCCGGCGGGGCCCCTCCTTAGGCGCTTCCTCCCGTCAACACGGTTGGCGGAGACAAGGTTGGCTAAAGGAGATTCGAAAGCTTCAGAAGAGCACACACCTCTTGATAAGGAAGCTCCCCTTCAGCCGCCTGGCAAGAGAAATATGTGTTAAATTCACGCGTGGTGTGGACTTCAATTGGCAAGCCCAGGCCCTACTGGCCCTACAAGAGGCAGCAGAAGCATTTCTAGTTCATCTCTTTGAGGATGCCTATCTCCTCACCTTACATGCCGGCCGAGTTACTCTCTTCCCAAAGGA
TGTGCAACTGGCCCGGAGGATCCGGGGCCTTGAGGAGGGACTCGGC
>Papio
ATGGGCCCGCGCCGCCGGAGCCGCAAGCCCGAGGCCCCGAGGAGGCGCAGCCCGAGCCCG------
ACCCCCGGCCCCTCCCGGCGGGGCCCCTCCTTAGGCCCTTCCTCCCGTCAACATGGTCGGCGGAGACAAGCTTGGCTAAAGGAGATCCGAAAGCTTCAGAAGAGCACACACCTCTTGATAAGGAAGTACCCCTTCAGCCGCCTGGCAAGAGAAATATGTGTTAAATTCACTCGTGGTGTGGACTTCAATTGGCAAGCCCAGGCCCTATTGGCCCTACAAGAGGCAGCAGAAGCATTTCTAGTTCATCTCTTTGAAGATGCCTATCTCCTCACCTTACATGCCGGCCGAGTTACTCTCTTCCCAAAGGAT
GTGCAACTGGCCCGGAGGATCCGGGGCATTGAGGCGGGACTCGGC
>Macaca
ATGGGCCCGCGCCGCCGGAGCCGCAAGCCCGAGGCCCCGAGGAGGCGCAGCCCGAGCCCG------
ACCCCCGGCCCCTCCCGGCGGGGCCCCTCCTTAGGCCCTTCCTCCCGTCAACATGGTCGGCGGAGACAAGCTTGGCTAAAGGAGATCCGAAAGCTTCAGAAGAGCACACACCTCTTGATAAGGAAGTACCCCTTCAGCCGCCTGGCAAGAGAAATATGTGTTAAATTCACTCGTGGTGTGGACTTCAATTGGCAAGCCCAGGCCCTATTGGCCCTACAAGAGGCAGCGGAAGCATTTCTAGTTCATCTCTTTGAAGATGCCTATCTCCTCGCCTTACATGCCGGCCGAGTTACTCTCTTCCCAAAGGAT
GTGCAACTGGCCCGGAGGATCCGGGGCATTGAGGCGGGACTCGGC
>Chlorocebus
ATGGGCCCGCGCCGCCGGAGCCGCAAGCCCGAGGCCCCGAGGAGGCGCAGCCCGAGCCCG------
ACCCCCGGCCCCTCCCGGCGGGGCCCCTCCTTAGGCCCTTCCTCCCATCAACATGGTCGGCGGAGACAAGCTTGGCTAAAGGAGATCCGAAAGCTTCAGAAGAGCACACACCTCTTGATTAGGAAGTACCCCTTCAGCCGCTTGGCAAGAGAAATATGTGTTAAATTCACTCGTGGTGTGGACTTCAATTGGCAAGCCCAGGCCCTATTGGCCCTACAGGAGGCAGCAGAAGCATTTCTAGTTCATCTCTTTGAAGATGCCTATCTCCTCACCTTACATGCCGGCCGAGTTACTCTCTTCCCAAAGGAT
GTGCAACTGGCCCGGAGGATCCGGGGCATTGAGGCGGGACTCGGC
>Aotus
ATGGGCCCGCGCCGCCGGAGCCGCAAGCCTGAGGCCCCGAGGAGGCGCCGCGCGAGTCCG------
ACCCCCAGCCCCCCTCGGCGGGGCCCCTCCTTAGGCGCTTCCTCACGTCCACGTGGTTATCGAAGGCAAGGTTGGCTAAAGGAGATCCGAAAGCTTCAGAAGAGCACACACCTCTTGTTAAGGAAGTACCCCTTCAGCCGCCTGGCAAGAGAAATATGTGTTAAATTCACTCGTGGTGTGGACTTCAATTGGCAAGCCCAGGCCCTATTGGCCCTACAAGAGGCGGCAGAAGCATTTCTAGTTCATCTCTTTGAGGATGCCTATCTCCTCACCTTACATGCCGGCCGAGTTACTCTCTTCCCAAAGGAC
GTGCAACTGGCCCGGAGGATCCGGGGCATTGAGGAGGGACTCGGC
>Saimiri
ATGGGCCCGCGCCGCCGGAGTCGCAAGCCCGAGGCCCCGAGGAGGCGCAGCCCGAGCCCG------ACCCCCGGCCCCTCCCGGCGGGGCCCCTCA---
GGCGCTTCCTCACGTCCACGTGGTTATCGAAGGCAAGGTTGGCTAAAGGAGATCCGAAACCTTCAGAAGAGCACACACCTCTTGATAAGGAAGTACCCCTTCAGCCGCCTGGCCAGAGAAATATGTGTTAAATTCACTCGTGGTGTGGACTTCAGTTGGCAAGCCCAGGCCCTATTGGCCCTACAAGAGGCGGCAGAAGCATTTCTAGTTCATCTCTTTGAGGATGCCTATCTCCTCACCTTACATGCCGGCCGAGTTACTCTCTTCCCAAAGGATGTGCAACTGGCCCGGAGGATCCGGGGCATTGA
GGAGGGACTCGGC
>Callithrix
ATGGGCCCGCGCCGCCGGAGCCGCAAGCCCGAGGCCCCGAGGAGGCGCAGCGCGAGCCCG------
ACCCCCAGCCCCTCCCGGCGGGGCCCCTCCTTAGGTGCTTCCTCACGTCCACGTGGTTATCGAAGGCAAGGTTGGCTAAAGGAGATCCGAAAGCTTCAGAAGAGCACACACCTCTTGTTAAGGAAGTACCCCTTCAGCCGCCTGGCAAGAGAAATAAGTGTTAAATTCACTCGTGGTGTGGACTTCAATTGGCAAGCCCAGGCCCTATTGGCCCTACAAGAGGCTGCAGAAGCATTTCTAGTTCATCTCTTTGAGGATGCCTATCTCCTCACCTTACATGCCGGCCGAGTTACTCTCTTCCCAAAGGAT
GTGCAACTGGCCCGGAGGATCCGGGGCATTGAGGGAGGACTCGGC
>Colobus
ATGGGCCCGCGCCGCCGGATTCGCAAGCCCGAGGCCCCGAGGAGGCGCAGCCCGAGCCCG------
ACCCCCGGCCCCTCCCGGCGGCGCCCCGCCTTAGGCCCTTCCTCCCGTCAACATGGTCGGCGGAGACAAGCTTGGCTAAAGGAGATCCGAAAGCTTCAGAAGAGCACACACCTCTTGATAAGGAAGTACCCCTTCAGCCGCCTGGCAAGAGAAATATGTGTTCAATTCACTCGTGGTGTGGACTTCAATTGGCAAGCCCAGGCCCTATTGGCCCTACAAGAGGCAGCAGAAGCATTTCTAGTTCATCTCTTTGAAGATGCCTATCTCCTCACCTTACATGCCGGCCGAGTTACTCTCTTCCCAAAGGAT
GTGCAACTGGCCCGGAGGATCCGGGGCATTGAGGCGGGACTCGGC
>Otolemur
ATGGGCCCGCGC---CGGAGACGCAAGCCCGAAACCCCGACGAGGCGCCCCGCGAGCCCG------
AGCCCCGGCCCCTCGCGGCGGGGCCCTTCCCTAGGCACTTCTTCCCGAGGACATGGTCAGAGGAGACCTTTGTGGTTAAAGGAGATCCGAAAACTTCAGAAGAGCACAAACCTCTTGTTAAGGAAAGCCCCCTTCAGCCGCCTGACAAGAGAAATATGTGGTAAATTCACTCGTGGTGTGGACTACAATTGGCAAGCGCAGGCCCTGTTGGCCCTACAAGAGGCAGCAGAAGCATTTCTAGTTCATCTCTTTGAAGATGCTTACCTTCTCACCTTACATGCTGGCCGAGTTACTCTTTTCCCAAAGGA
TGTGCAACTGGCAAGAAGGATCCGAGGCATTCAGGAAGGGCTGGGC
>Lemur
ATGGGCCCGCGCCGCCGGAGGCGCAAGCCCGAGACCCCGACGAGGCGCGTCGAGAGCCCG------
ACCCCCGGCCCCTCGCGGCGGCGGCCCCCGTAGCCCCTACCCCCCGTCAACATCGTCGGAGGACATTTGGATGGTTACAGGAGATCCGAAAACTTCAGAAGAGCACACACCTCTTATTAAGGAAGGCCCCCTTCAGCCGCGTGGCAAGAGAAGTATGTACTAAATTCACTCGTGGTGTGGACTTCAATTGGCAAGCCCAGGCCCTGTTGGCCCTACAAGAGGCTGCAGAAGCATTTCTAGTCCATCTCTTTGAGGATGCTTATCTCCTCACCCTACATGCCGGCCGAGTTACTCTTTTCCCAAAGGAC
GTGCAGCTGGCCAGGAGGATCCGAGGCATTCAGGAAGGGCTCGGC
```

Asteraceae – CenH3 alignment before masking unreliable residues inferred in Bali-Phy

>Lactuca_sativa
ATGGCGAGAACCAAACAACCTGCTAAACGAAGTTGGGGCAAGCGACAATCT------GCTGGAGCATCAACTTCAACCTCAACAAGTACGCCACGGAAATCTCCGAGAAAGGATCCA---
GGGAGTTCAGGAACTGGACAGAGGCAGAAACAGAAACCCCATCGTTTTAAGCCTGGAACTCAAGCACTTCGTGAGATTCGTCGTCTTCAGAAGACTGTTAACCTTCTCATTCCAGCTGCTCCTTTCATTCGAACTGTAAAAGAGATAAGCAACTACATTGCCCCTGAAGTTACACGTTGGCAAGCTGAAGCTCTACAAGCCCTTCAAGAGGCTGCAGAAGATTACATAGTTCAGTTGTTTGAAGATTCAATGTTGTGCTCGATTCATGCAAAGCGTGTTACCCTGATGAAAAAGGACATGGAATTGGCT
AGAAGGCTTACGAAGAAAGGGCAACCATGG
>Taraxacum_koksaghyz
ATGGCGAGAACCAAACAACCTGCTAAACGGAGTGCGGGCAATCGAACACCT------GCTCAAGCATCAACTTCAACTTCAACCAGTACGCCGCGAAGAAGTCCGAGAAAGGTTACA---
GGGAGTTCGGGAACGGGAGAAAGGCAGAAACAGAAACCCCATCGTTTTAAGCCTGGTACTCAGGCACTTCGTGAGATTCGTCGTCTCCAGAAGACCGTTAATCTACTCATTCCAGTCGCTCCATTCGTACGAACTGTAAAGGAAATAAGCAACTACATTGCCCCTGAAGTCACACGCTGGCAAGCTGAAGCTCTACAATGCCTTCAAGAGGCAGCAGAAGATTACTTAATCCGATTGTTTGAAGATTCAATGCTATGTGCGATTCATGCAAAACGTGTTACCCTCATGCAAAAGGATTTGGCATTGGC
ACGAAGGCTTACAAAGAAAGGGCAGCCATGG
>Carthamus_tinctorius
ATGGCGAGAACCAAACAACCTGCTAAACGCAGTTCTGGCAAGCGT---GAT------GCTAGACCATCTACC------TCCACGCCTACGCCAAGGCCAAGTGCAAGGAAGAATCCC---GAGAGCTCAGGAGCTGGGGATGGTCAG------
AGGCGCCATCGTTATAGGCCTGGGACTCAGGCGCTTCGTGAGATTAGGCGCTTGCAGAAGACTGTCAATCTTCTCATTCCGGCTGCTCCTTTCATTCGAACCGTAAAGGAGATAAGCAACTACATTGCGCCAGAAGTCACTCGCTGGCAAGCTGAAGCTCTACAAGCCCTTCAAGAGGCAGCAGAAGATTACTTAATTCAGTTGTTTGAAGATTCAATGCTATGCGCGATTCATGCAAAACGCGTTACCCTCATGAAAAAGGATTGGGAGTTGGCACGGCGGCTTGGGAAGAAAGGGCAACCGTGG
>Cichorium_intybus
ATGGCGAGAACAAAGCAACCTGCTAAACGGAGTTGGGGCAATCGAAAGTCTAGTCAATCTCGAGCATCAACCTCAACTTCAACCAGTACGCCACGAAAAAGTCCGAGAAAGGATCCA---GGG------
AGAACTGGAGAAAGGCGGCAACAGAAACCCCATCGTTTCAAGCCTGGGGCTCAAGCACTTCGTGAAATTCGTCGTCTTCAGAAGACTGTTAATCTTCTCATTCCAGCTGCCCCATTCATTCGAACTGTAAAAGAAATAAGCAACTACATTGCCCCTGAAGTTACACGCTGGCAAGCTGAAGCTATACAGGCCCTTCAAGAGGCAGCAGAAGATTACTTAGTTCAGTTGTTTGAAGATTCAATGCTATGTTCGATTCATGCAAAGCGTGTTACCCTCATGAAAAAGGACTGGGAATTGGCACGGAGACT
TACAAAGAAAGGTCAACCATGG
>Helianthus_exilis_CHES4473
ATGGCGAGAACCAAACACCCTGCTAAACGCAGTTCAGGCATACCAGTAGAC------GGTAGATCATCCACT------TCAACAAACACACCAAGAAAGAGTCCGAGGAAGAATCGA---GGT------------GGAGAAAACAGG------
AAGCCGCATAGGTTTAAGCCTGGGACACAGGCGCTACGTGAGATTAGGCGTTTGCAGAAGACGGTTGAACTGATCATTCCGGCTGCTCCGTTTATTCGAACTGTAAAGGAGATAAGCAACTACATGGCCCCTGAAATCACTCGCTGGCAAGCCGAAGCTCTACAAGCCCTTCAAGAGGCAGCAGAAGATTACCTAATTCAGTTGTTTGAAGACTCAATGCTATGTGCGATTCATGCAAAGCGCGTTACCCTCATGAAAAAGGATTGGGAGTTGGCACGGCGGATTGGGAAGAAAGGGCAGCCATG
G
>Helianthus_annuus
ATGGCGAGACCCAAACCACCTGCTAAACGCAGTTCAGGCATACCAGCAGAC------GGTAGATCATCCNCT------TCACCAAACACACCAAGAAAGAGTCCGAGGAAGAATCGAGTAGGT------------GGAGAAAACAGG------
AAGCCGCATAGGTTTAAGCCTGGGACACAGGCGCTACGTGAGATTAGGCGTTTGCAGAAGACGGTTGAACTGATCATTCCGGCTGCTCCGTTTATTCGAACTGTAAAGGAGATAAGCAACTACATGGCCCCTGAAATCACTCGCTGGCAAGCCGAAGCTCTACAAGCCCTTCAAGAGGCAGCAGAAGATTACCTAATTCAGTTGTTTGAAGACTCAATGCTATGTGCGATTCATGCAAAGCGCGTTACCCTCATGAAAAAGGATTGGGAGTTGGCACGGCGGATTGGTAAGAAAGGGCAGCCATG
G
>Flaveria_vaginata
ATGGCGAGAACCAAACACCCTGCTAAACGAAGTTGGGGCACTAGAAAAGAT------GGTAGAGCATCTACT------TCAACGAGCACTCCGAGGAAAAGCCCTAGGAAGGATGCA---GGGGGTTCTGGAACTGGAGAAAAGCAG------
AGGCCCCATCGGTTTAAGCCTGGGACTCAGGCACTTCGGGAGATTAGGCGTTTGCAAAAGACGGTCAATCTTCTCATTCCTGCTGCTCCCTTCATTCGAACTGTGAGGGAGATAAGCAACTACCTGGCCCCTGAAATCACTCGCTGGCAAGCTGAAGCTTTACAAGCCCTTCAAGAGGCAGCAGAAGATTACATAATTCAGTTGTTTGAAGATTCAATGCTATGCGCAATTCATGCAAAACGTGTTACCCTTATGAAAAAGGATTGGGAGTTGGCACGGCGTCTCGGGAAGAAAGGGCAGCCATGG

Brassicaceae– CenH3 alignment before masking unreliable residues inferred in Bali-Phy

>Brassica_napus_BrCENH38
ATGGCGAGAACGAAACATTTCGCTTCCAGGGCACGAGATCGCAATCGAACT------AATGCG---ACTGCT---TCATCTTCGGCGGCGGCG---GCGGAAGGTCCGAGTGCGACCCCGACGAGAAGA---GAAGGC------AGCCAA---------------------GGA-----------------------GAA---GCTCAACAGACA---GCAACTCCTACTACGACTCCACCA------------GCCGGTAGAAAAAAAGGA---------GGGACTAAGCGAACTAAACAAGCTATGCCTAAAAGTTCCAAC------AAG---
AAGAAGACATTCCGTTACAAGCCTGGAACCGTTGCCCTCAGAGAGATTCGCCATTTCCAGAAGACCACCAAACTTCTTATCCCTGCCGCTAGTTTCATCCGAGAAGTGAGAAGTGTCACCCAGATCTTT------
GCCCCTCCCGATGTTACCCGTTGGACTGCTGAAGCTCTTATGGCTATTCAAGAGGCGGCTGAAGATTTTTTAATTGGCTTGTTCTCTGATGCTATGCTTTGCGCTATCCACGCAAGACGTGTTACTCTAATGAGAAAAGATTTTGAGCTTGCACGCCGTCTTGGAGGAAAAGGCAGACCATTG
>Brassica_carinata_BrCENH36
ATGGCGAGAACCAAACATTTCGCTTCCAGGGCACGAGATCGCAATCGAACT------AATGCG---ACTGCT---TCATCTTCGGCGGCGGCG---GCGGAAGGTCCGAGTGCGACCCCGACGAGAAGA---GAAGGC------AGCCAAGATGAA------GGTGGT-----------------------GAA---GCTCAACAGAGT---------GCAACTCCTACTACAACTCCATCA------------GCCGGTAGAAAAAAAGGA---------GGGACTAAGCGAACTAAACAAGCTATGCCTAAAAGTTCCAAC------AAG---
AAGAAGACATTCCGTTACAAGCCTGGAACCGTTGCCCTCAGAGAGATTCGCCATTTCCAGAAGACCACCAAACTCCTTATCCCTGCCGCTAGTTTCATCCGACAAGTGAGAAGTGTCACCCAGATCTTT------
GCCCCTCCCGATGTTACCCGTTGGACTGCTGAAGCTCTTATGGCTATTCAAGAGGCGGCTGAAGATTTTTTAATTGGCTTGTTCTCTGATGCTATGCTTTGCGCTATCCACGCAAGACGTGTTACTCTAATGAGAAAAGATTTTGAGCTTGCACGCCGTCTTGGAGGAAAAGGCAGACCATTG
>Brassica_oleracea_BrCENH33
ATGGCGAGAACCAAACATTTCGCTTCCAGGGCACGAGATCGCAATCGAACT------AATGCG---ACTGCT---TCATCTTCGGCGGCGGCG---GCGGAAGGTCCGAGTGCGACCCCGACGAGAAGA---GAAGGC------AGCCAAGATGAA------GGTGGT-----------------------GAA---GCTCAACAGAGT---------GCAACTCCTACTACAACTCCATCA------------GCCGGTAGAAAAAAAGGA---------GGGACTAAGCGAACTAAACAAGCTATGCCTAAAAGTTCCAAC------AAG---
AAGAAGACATTCCGTTACAAGCCTGGAACCGTTGCCCTCAGAGAGATTCGCCATTTCCAGAAGACCACCAAACTCCTTATCCCTGCCGCTAGTTTCATCCGACAAGTGAGAAGTGTCACCCAGATCTTT------
GCCCCTCCCGATGTTACCCGTTGGACTGCTGAAGCTCTTATGGCTATTCAAGAGGCGGCTGAAGATTTTTTAATTGGCTTGTTCTCTGATGCTATGCTTTGCGCTATCCATGCAAGGCGTGTTACTCTAATGAGAAAAGATTTTGAGCTTGCACGCCGTCTTGGAGGAAAAGGCAGACCATTG
>Raphanus_sativus_cenpA1
ATGGCGAGAACGAAGCATTTCGCTTCCAGAGCACGAGATCGCAATCAACCT------AATGCG--------------------GCGGCGGCG---GCGGCAGGTCCGAGTGCGACACCGACGAGAAGA---GGCAGC------AGCCAA-----------GGTGAA-----------------------GAA---GCTCAACAGACAACGCCTACCACAACT------------TCACCAGCAACAACCGCCTCCGGTAGAAAAAAA-----------GGGACTAAGCGAACTACACAAGCTATGCCTAAAAGTTCC---------AAG---
AAGAAGACTTTCCGTTACAAGCCTGGAACTGTTGCCCTCAGAGAGATTCGCCATTTCCAGAAGTCCACCAAACTTCTCATCCCTTCTGCTCCTTTCATCCGAGAAGTCAGAAGTATCACCCACAACCTT------
GCCGCTGCCTATGTTACGCGTTGGACTGCTGAAGCTCTTATTGCTCTTCAAGAGGCGGCAGAAGATTTCTTAGTTGGCTTGTTCTCTGATGCAATGCTTTGCGCTATCCATGCAAAACGTGTTACTCTAATGAGAAAAGATTTTGAGCTTGCACGCCGGCTTGGAGGAAAAGGCAGACCCTTT
>Brassica_juncea_BrCENH35
ATGGCGAGAACGAAGCATTACGCATCCAGGGCACGAGATCGCAATCGAAAT------AATGCG---ACTGCT---TCATCTACCGAGGCGGCG---GCTGCAAGTCCGAGTGCGACGCCGACGAGAAGA---GGCAGCAGCAGCAGCCAA-----------GGTGCTCGT---------------------GAA---GCTCAACAG--AGTTCCAACCAGAAAAAG---
AAGAAGTCTTTCCGTTACAAGCCTGGAACCGTTGCTCTCAGAGAGATTCGCCAGTTCCAGAAGACCACCAAACTTCTCATCCCTGCTGCTAGTTTCATCAGAGAAGTGAGAAGTATTACCCATATCCTT------
GCTCCTCCCGAAATTACGCGTTGGACTGCTGAAGCTCTTGTTGCTCTTCAAGAGGCGGCAGAAGATTACTTAGTTGGCTTGTTCTCTGATGCAATGCTCTGCGCTATCCACGCAAGACGTGTTACTCTAATGAGAAAAGATTTTGAGCTTGCACGCCGTCTTGGAGGAAAAGGCAGACCATTG
>Brassica_nigra_BrCENH32
ATGGCGAGAACGAAGCATTACGCATCCAGGGCACGAGATCGCAATCGAAAT------AATGCG---ACTGCT---TCATCTACCGAGGCGGCG---GCTGCAAGTCCGAGTGCGACGCCGACGAGAAGAGGAGGCAGCAGCAGCAGCCAA-----------GGTGCTCGT---------------------GAA---GCTCAACAG--AGTTCCAACCAGAAAAAG---
AAGAAGTCTTTCCGTTACAAGCCTGGAACCGTTGCCCTCAGAGAGATTCGCCAGTTCCAGAAGACCACCAAACTTCTCATCCCTGCTGCTAGTTTCATCAGAGAAGTGAGAAGTATTACCCATATCCTT------
GCCCCTCCCGAAATTACGCGTTGGACTGCTGAAGCTCTTGTTGCTCTTCAAGAGGCGGCAGAAGATTACTTAGTTGGCTTGTTCTCTGATGCAATGCTCTGCGCTATCCACGCAAGACGTGTTACTCTAATGAGAAAAGATTTTGAGCTTGCACGCCGTCTTGGAGGAAAAGGCAGACCATTG
>Eruca_sativa_cenpA
ATGGCGAGAACGAAGCATTTTGCATCCAGGGCACGAGATCGCAATCGAAAT------AATGCG---ACAGCT---TCATCTTCCGCGGCGGCGGCGGCGGCTGGTCCGAGTGCGACACCGACGAGAAGA---GGCAGC------AGGCAA-----------GGTGGT---GGTGGTGGTGGTGGTGTT---GAG---GCTCAACAG---GGTTCCAAC---AAGAAG---
AAGAAGTCTTTCCGTTACAAGCCTGGAACCGTTGCTCTCAGAGAGATTCGCCATTTCCAAAAGACCACCAAACTCCTTATCCCTGCTGCTACTTTCATCCGACTAGTCAGAAGCATCACCCTTGATCGT------
GCCAAGCCCCAAGTTACGCGTTGGACTGCTGAAGCTCTTGTTGCTCTTCAAGAGGCGGCAGAAGATTACTTAGTTGGCTTGTTCTCTGATTCAATGCTCTGCGCTATCCATGCTAAACGTGTTACTCTTATGAGGAAAGATTTTGAGCTTGCACGCCGCCTTGGAGGTAAAGGCAGACCATGG
>gArabidopsis_lyrata_HTR12
ATGGCGAGAACCAAGCATTTCGCTACCAAGTCACGATCTGGGAATCGAACT------GATGCC---AATGCT---TCATCTTCTCAG---------GCGGCAGGTCCGACGACGACCCCGACAACAAGA---GGC---------ACTGAA-----------GGTGGA---------------------GATAAT---ACTCAACAAACAAATCCT---ACAACT------------TCACCAGCTACT---------GGTGGAAGGAGG--------------CCTAGGAGAGCCAGACAGGCTATGCCGCGAGTTTCA---------CAG---
AATAAGCCTTATCGATACAAGCCAGGAACCGTTGCTCTGAGAGAGATTCGACATTTCCAGAAGCAGACAAACCTTCTTATTCCAGCTGCTAGCTTCATAAGACAAGTGAGAAGTATAACTCAT------
GCGTTGGCCCCTCCCCAAATCAATCGTTGGACAGCTGAAGCTCTTGTGGCTCTTCAAGAGGCTGCAGAAGATTACTTGGTTGGATTGTTCTCAGATTCAATGCTCTGTGCTATCCATGCAAGACGTGTTACTCTAATGAGAAAAGACTTTGAGCTTGCACGCCGGCTTGGAGGAAAAGGCAGACCATGG
>Arabidopsis_arenosa_HTR12
ATGGCGAGAACCAAGCATTTCGCTACCAAGTCACGAACTGGGAATCGAACT------GATAAC---GGTGCT---TCATCTTCTCAG---------GCGACAGGTCCGACTACGACCCCGACAACAAGA---GGC---------ACTGAA-----------GGTGGA---------------------GATAAT---ACTCAACAAACAAATCCT---ACAACT------------TCACCAGCTACT---------GGTGGAAGGAGG--------------CCTAGGAGAGCCAGACAGGCTATGCCGCGAGGTTCA---------CAG---
AAGAAACCTTATCGATTCAAGCCAGGAACCGTTGCTCTGAGAGAGATTCGACATTTCCAGAAGCAGACAAACCTTCTTATTCCAGCTGCTAGCTTCATAAGACAAGTGAGAAGTATAACTCAT------
GCGTTGGCCCCTCCCCAAATCAATCGTTGGACAGCTGAAGCTCTTGTGGCTCTTCAAGAGGCGGCAGAAGATTACTTGGTTGGTTTGTTCTCAGATTCAATGCTCTGTGCTATCCATGCGAGACGTGTTACTCTAATGAGAAAAGACTTTGAGCTTGCACGCCGGCTTGGAGGAAAAGGCAGACCATGG
>Arabidopsis_suecica_cenpA
ATGGCGAGAACCAAGCATTTCGTTACCAAGTCACGAACTGGGAATCGAACT------GATGCC---AATGCT---TCATCTTCTCAG---------GCGTCAGATCCGACGACGACCCCGACAACAAGA---GGC---------ACTGAA-----------GGTGGA---------------------GATAAT---ACTCAACAAACAAATCCT---ACAACT------------TCACCAGCTACT---------GGTGGAAGGAGG--------------CCTAGGAGAGCCAGACAGGCTATGCCGCGAGGTTCA---------CAG---
AAGAAGCCTTATCGATACAGGCCAGGAACCGTTGCTCTGAGAGAGATTCGACATTTCCAGAAGCAGACAAACCTTCTTATTCCAGCTGCTAGCTTCATAAGACAAGTGAGAAGTATAACCCAT------
GCGCTGGCCCCTCCCCAAATCAATCGTTGGACAGCTGAAGCTCTTGTGGCTCTTCAAGAGGCGGCAGAAGATTACTTGGTTGGTTTGTTCTCAGATTCAATGCTCTGTGCTATCCATGCAAGACGTGTTACTCTAATGAGAAAAGACTTTGAGCTTGCACGCCGGCTTGGAGGAAAAGGCAGACCATGG
>Arabidopsis_halleri_subsp._halleri_HTR12B1
ATGGCGAGAACCAAGCATTTCGCTACCAGGACACGATCTGGGAATCGAACT------GATGCC---AATGCT---TCATCTTCTCAG---------GCGGCAGGTCCGACTACGACCCCGACAACAAGA---GGC---------ACTGAA-----------GGTGGA---------------------GATAAT---ACTCAACAAACAAATCCT---ACAACT------------TCACCAGCTACT---------GGTGGAAGGAGG--------------CCTAGGAGAGCCAGACAGACTATGCCACGAGGTTCA---------CAG---
AAAAAGCCTTATCGATACAAGCTAGGAACCGTTGCTCTGAGAGAGATTCGACATTTCCAGAAGCAGACAAACCTTCTTATTCCAGCTGCTAGCTTCATAAGACAAGTGAGAAGTATAACTCAT------
GCGTTGGCCCCTCCCCAAATCAATCGTTGGACAGCTGAAGCTCTTGTGGCTCTTCAAGAGGCGGCAGAAGATTACTTGGTTGGTTTGTTCTCAGATTCAATGCTCTGTGCTATCCATGCAAGACGTGTTACTCTAATGAGAAAAGACTTTGAGCTTGCACGCCGGCTTGGAGGAAAAGGCAGACCATGG
>Arabidopsis_thaliana_HTR12
ATGGCGAGAACCAAGCATCGCGTTACCAGGTCACAACCTCGGAATCAAACT------GATGCCGCCGGTGCT---TCATCTTCTCAG---------GCGGCAGGTCCAACTACGACCCCGACAAGGAGA---GGC---------GGTGAA-----------GGTGGA---------------------GATAAT---ACTCAACAAACAAATCCT---ACAACT------------TCACCAGCTACT---------GGTACAAGGAGA------------GGGGCTAAGAGATCCAGACAGGCTATGCCACGAGGCTCA---------CAG---
AAGAAGTCTTATCGATACAGGCCAGGAACCGTTGCTCTAAAAGAGATTCGCCATTTCCAGAAGCAGACAAACCTTCTTATTCCGGCTGCCAGTTTCATAAGAGAAGTGAGAAGTATAACCCAT------
ATGTTGGCCCCTCCCCAAATCAATCGTTGGACAGCTGAAGCTCTTGTTGCTCTTCAAGAGGCGGCAGAAGATTACTTGGTTGGTTTGTTCTCAGATTCAATGCTCTGTGCTATCCATGCAAGACGTGTTACTCTAATGAGAAAAGACTTTGAACTTGCACGCCGGCTTGGAGGAAAAGGCAGACCATGG
>Crucihimalaya_himalaica_HTR12
ATGGCGAGAACCAAGCATTTCGCTACTAGGTCACGACCTCGGAATCAAACT------GATGCC---ACTGCT---TCAGCTTCTCAG---------GCGACAGGTCCGAGTACGAACCCGACAACAAGA---GGT---------AGTGAA-----------GGTGAA---------------------GATGCT---GCTCGAGGAACAAATCCT---ACAACT------------TCACCAGCTACT---------GGTAGAAAAAAA------------GGGGTTAAGAGGGCCAGACATGCTATGCCACAAGGTTCA---------CAG---
AAGAAGCCTTATCGTTACAAGGCAGGAACAGTCGCTCTGAGAGAGATTCGCCATTTTCAGAAGAACACAAACCTTCTTATTCCAGCTGCTAGTTTTATAAGACAAGTGAAAAGTATAACCTAT------
GCGGTGGCCCCTCCGCAAATCACTCGTTGGACAGCTGAAGCTCTTGTGGCTCTTCAAGAGGCGGCAGAAGATTACTTGGTTGGTTTGTTCTCAGATTCAATGCTCTGTGCTATCCATGCAAGACGAGTTACTCTAATGAGAAAAGACTTTGAGCTTGCACGCCGGCTTGGAGGAAAAGGTAGACCCTGG
>Crucihimalaya_wallichii_cenpA
ATGGCGAGAACCAAGCATTTCGCTACTAGGTCACGACCTCGGAATCAAACT------GATGCC---ACTGCT---TCAGCTTCTCAG---------GCGACAGGTCCGAGTACGAACCCGACAACAAGA---GGT---------AGTGAA-----------GGTGAA---------------------GATGCT---GCTCGAGAAACAAATCCT---ACAACT------------TCACCAGCTACT---------GGTAGAAAAAAA------------GGGGTTAAGAGGGCCAGACATGCTATGCCACAAGGTTCA---------CAG---
AAGAAGCCTTATCGTTACAAGGCAGGAACAGTCGCTCTGAGAGAGATTCGCCATTTTCAGAAGAACACAAACCTTCTTATTCCAGCTGCTAGTTTTATAAGACAAGTGAAAAGTATAACCTAT------
GCGGTGGCCCCTCCGCAAATCACTCGTTGGACAGCTGAAGCTCTTGTGGCTCTTCAAGAGGCGGCAGAAGATTACTTGGTTGGTTTGTTCTCAGATTCAATGCTCTGTGCTATCCATGCCAGACGAGTTACTCTAATGAGAAAAGACTTTGAGCTTGCACGCCGGCTTGGAGGAAAAGGTAGACCCTGG
>Turritis_glabra
ATGGCGAGAACCAAGCATTTCGCTACCAGGTCACGACCTCGGAATCAAACT------GAT--------------TCATCTTCTCAG---------GCGGCAGGTCCGAGTACGAACCCAACAACAGGA---GGT---------AGTGAA-----------GGTGGA---------------------GATGCT---GCTCAACAAACAACTCCT---ACGACT------------TCACCAGCTACT---------GGTAGAAAAAAA------------CGGGCTAAGAGAGCCAAACAGGCTATGCCACAAGGTTCA---------CAG---
AAGAAGCCTTATCGTTACAAGCCAGGAACCATTGCTCTGAGAGAGATTCGCTATTTTCAGAAGAACACAAACCTTCTTATTCCAGCTGCTAGTTTTATAAGAGAAGTGAGAAGTATAACCCAT------
GCGCTGGCCCCTCCGCAAATCTCTCGTTGGACAGCTGAAGCTCTTGTTGCTCTCCAAGAGGCGGCAGAAGATTACTTGGTTGGTTTGTTCTCAGATTCCATGCTCTGTGCAATCCATGCAAGACGTGTTACTCTGATGAGAAAAGACTTTGAGCTTGCACGCCGGATTGGAGGAAAAGGCAGACCATGG
>Olimarabidopsis_pumila_cenpA1
ATGGCGAGAACCAAGCATAACGCTATCAGGTCACGA------GATCGAACT------GGTGCA---ACTGCT---TCATCATCTCAG---------GCAGCAGGTCCGAGTACGAACCCCACAGCAGGA---GGT---------AGTGAA--------------------------------------GATGCT---GCTCAACAAACAACTCCT---ACGACT------------TCACCAGCTACT---------GGTAGTAAAAAA------------CGGGCTAAGAGAGCCAGACAGGCTATGCCACGAGGTTCA---------CAG---
AAGAAGCCTTATCGTTACAAGCCAGGAACTGTTGCTCTGAGAGAGATTCGCCATTTTCAGAAGACCACTAGCCTTCTTCTTCCAGCTGCTCCTTTTATAAGACAAGTGAGAAGTATAAGCAGT------
GCTTTGGCACCTAGGGAAATCACTCGTTGGACAGCTGAAGCTCTTGTGGCTCTCCAAGAGGCGGCAGAAGATTACTTGGTTGGTTTGTTCTCAGATTCCATGCTCTGTGCTATCCATGCAAAACGTGTTACTCTGATGAGAAAAGACTTTGAGCTTGCACGCCGGCTCGGAGGAAAAGGCAGACCATGG
>Cardamine_flexuosa_cenpA1
ATGGCGAGAACGAAGCATTTCCCTAACAGGACACGGCCTCGGACTCAAACT------GATGCC---ACTGCT---TCATCTACTCCG---------GCGGCTGGTCCGAGTACGAAGCGGCCAACAAGG---GCC---------AATCAA-----------GGTGAA-----------------------GAA---ACTCAACAAACAAATCCT---ACAACT------------TCACCAGCTACC---------AGTAAAAAGAAA------------GGGGCTAAGAGAACTAGACGGGCCATGCCACAAGGTTCA---------CAA---
AAGAAGCCTAATCGTTACAAGCCAGGAACCGTTGCTCTTAGAGAGATTCGCCATTTCCAGAAGAGCACAAATCTTCTGATTCCAGCTGCTAGTTTTATCAGACAAGTGAGAAGTATAACCCAAATGTAT------
GCCCCTCCGCAAATTAATCGTTGGACTGCTGAAGCTCTTGTGGCTCTTCAAGAGGCGGCAGAAGATTACTTGATTGGTTTGTTCTCAGATTCAATGCTCTGTGCTATCCATGCAAGACGTGTTACTCTAATGAGGAAAGACTTTGAGCTTGCACGCCGGCTTGGAGGAAAAGGCAGACCATGG
>Capsella_bursapastoris_cenpA1
ATGGCGAGAACAAAGCATTTCGCTACGAGGTCCGGACCTCGGACTCCAGCT------GTT---------GCT---TCGTCTTCTCAG---------GCGGCTGTTCCCAGTTCGAGCCCGGCAACAAGA---GGC---------AGAGTA-----------GGTGTA---------------------GACGCTGCTGCTCAACAACCAACTCCT---GCAACT------------TCACCTGCTACT---------GCTAAAAAAAAA------------GGGGCGAAGAGAGCTAGATTTGGTAGGCCACAAGGTTCA---------
CAAAAAAAGAAGCCTTACCGTTACAGGCCAGGAACCGTTGCTCTGAGAGAGATTCGCCATTATCAGAAGGGCACTAGCCTTCTTATTCCAGCTGCTGCCTTTATCAGACAAGTGAGAAGTATAACCAAT------
GCAGTGGCCCCTCGGGAAGTAAATCGTTGGACAGCTGAAGCTCTTGTGGCTCTTCAAGAGGCGGCAGAAGATTTCTTGGTTGGTTTGTTCTCTGATTCAATGCTCTGTGCTATCCATGCAAGACGTGTTACTCTAATGAGAAAAGACTTTGACCTTGCACGCCGGCTTGGAGGAAAAGGCAGACCCTGG
>Arabis_hirsuta_cenpA1
ATGGCGAGAACGAAGCATTTCGCA------AGACGAGGTCAGAATCGAACA------GATGCT---AACGTT---TCCTCTTCGCCG---------GCGGCAGGTCCGAGTACGACTCCGGCGAGAAGG---GCT---------AGTGAA-----------GATGGA---------------------GATGAT---GCTCAACAAACAACTCCT---GCAACT------------TCACCAGCTACT---------GGTAGTAAAAAT---AGTAATGGAGGAGCTAAGAGAACT---------ATGCCACAAAGTTCA---------CAG---
AAGAAGCCTCATCGATACAGGCCAGGAACCGCTGCTCTGAGAGAGATTCGCAAATTCCAGAAGTCCACAAAACTTCTAATTCCTGCTGCTAGTTTTATCAGAGAAGTGAAAGATATAACCTATACTTAT------
GCTCCTCCGCAAATCACTCGTTGGACAGCTGAAGCTCTTGTAGCTATCCAAGAGGCGGCAGAAGATTACTTAGTTGGTTTGTTCTCAGATTCAATGCTTTGTGCTGTTCATGCAAGACGTGTTACTCTAATGAGAAAAGACTTTGAGCTTGCACGCCGGCTTGGAGGAAAAGGCAGACCATGG
>Lepidium_virginicum_cenpA1
ATGACGAGAACCAAGCATTACGTTTCGAGGCCACAACCTCATCAT------CGCAAGGATGCC---ACTGTTTCTTCATCTTCTCCG---------GCGGCTGGTACAAGTACGAATCGAACAACAAGG---AGT---------TCTGAA------GGTGAAGCTGGA---------------------GATAAT---GCTCAACAAACAATTCCT---ACAACT------------TCACCGGCTACT---------AATAGCAAAAAA------------GGGGCAAAAAGAACCAAAGAAACTATGCCACAAAGTTCA---------AAT---
AAGAAAACATATCGATACAAGCCAGGCACCGTTGCTCTGAGAGAGATTCGCCATTTTCAGAAGAGCACAAATCTTCTAATTCCAGCTGCTAGTTTCATAAGAGAAGTGAGATGTATTACTCAG------
GCTGTGGCCCCTCCGCAAATCAATCGTTGGACAGCTGAAGCTCTTGTGGCTCTTCAAGAGGCGGCAGAAGACCACGTGGTTGGCTTGTTCTCTGATTCGATGCTTTGTGCTATCCATGCAAGACGAGTTACACTTATGAAAAAAGATTTTGAGCTTGCACGCCGGCTTGGAGGAAAAGGCAGACCATGG

Bryophytes – CenH3 alignment before masking unreliable residues inferred in Bali-Phy

>Ceratodon_purpureus
ATGGCGAGGCTGAAGCAGACGCCGCTGCGGAGCAACAACCGGGCGTCGACGAGC---TCTGCTGGTCGAGCTGCA------CCCTCTCCTGCC---------
GCTAGAGCGGCACAGCGCGCCGCGGCCGAGGAAGCCGCACCGGTGGAGGCCTGGGACTAAGGCGTTGATGGAGATTCGGTACTACCAGAAGACTTGCGACCTGCTCATCCCTCGCCTTCCTTTCGCTCGCTATGTGAAGGAAATTACGTCTATGTACGCAAGTGATGTTTCTCGATGGACGGCAGAAGCGTTGACAGCTCTTCAAGAGGCAACAGAAGACTACATAGTGCATTTGTTTGAGGACACAAATTTGTGTGCTATCCATGCAAAGCGAGTCACCATAATGCCGAAGGATCTGCAGTTG
GCCAGACGCCTGCGGGGTGTCATCGAGAAAGCATCA
>Pohlia_nutans
ATGGCGAGGCGGAAGTCCACCCCTCTGCACGGCAACAGACGCGCCTCCACGAGC---TCC---------------AATCCTGCTGCTGCTGCT---------GCT---------------
CAGCGGAGGCCTCGGAAACCGCACCGATGGAGACCCGGTACCAAGGCGTTGCAAGAGATTCGCCAATACCAGAAGACTGGCGACTTGCTTATCCCCCGCCTCCCGTTTGCTCGCTACGTGAAGGAAATCACGTCAATGTATGCAAGTGACGTTTCTCGATGGACGGCGGAGGCTTTGACAGCCCTTCAAGAGGCAACAGAAGACTACATGGTGCATTTGTTTGAAGACACAAATTTGTGTGCCATCCATGCGAAGCGAGTCACCATAATGCCGAAAGATCTGCAGTTGGCCAGACGACTACGAGGT
GTCATCGAAAAAGCATCA
>Physcomitrella_patens
ATGGCAAGAAGGAAAACTACCCCTGTACATGGCAACCACCGAGCTTCTACTAGC---TCTGTTGGTGGAGCTGCG------GTG--
AGGCCCAGGAAGCCGCACCGATGGAGACCCGGGACCAAGGCATTGCAAGAGATCCGCCATTATCAAAAGACGTGCGACCTTCTCATCCCTCGACTACCTTTTGCCCGCTATGTGAAGGAAATTACGATGATGTACGCTAGTGATGTTTCCCGGTGGACTGCGGAAGCTTTGACCGCTCTCCAAGAGGCCACTGAAGATTATATGTGCCATTTGTTTGAAGACACCAATTTGTGTGCTATCCATGCTAAGCGAGTAACCATAATGCCGAAGGATCTGCAGTTGGCCAGACGACTACGAGGTGCCATTG
TT---------
>Hedwigia_ciliata
ATGGCGAGGCGGAAGTCTACTCCCCTGCATGGCCGCAGACGCGCGTCCACTAGC---TCTGCAGGGGGAGCTGCA------GCAGCT---------------GCG---------------
GCGCGGAGGCCTCGGAAACCGCACAGATGGAGACCAGGCACCAAGGCATTGCAAGAGATCCGCCACTACCAGAAGACTTGCGACTTGCTTATTCCTCGACTTCCTTTTGCTCGCTATGTGAAAGAAATCACGTCTATGTATGCAAGTGACGTTTCTCGATGGACGGCGGAGGCTTTGACAGCGCTTCAAGAGGCAACAGAAGACTACATGGTGCATTTATTTGAAGACACAAATTTGTGTGCCATCCATGCGAAGCGAGTCACCATAATGCCGAAGGATCTGCAGTTGGCCAGACGACTACGAGGT
TTCATCGAAAAAGCATCA
>Anomodon_attenuatus
ATGGCGAGGCGGAAGTCGACCCCTCTGCACGGCAACAAACGCGCACCCACCCGC---CCG---------GCTGCT------GCTGCTGCTGCTGCCTCGACTTCG---------------
ACGAGGAGGCCGAGGAAAGCGCACCGATGGAGACCGGGCACCAAGGCGTTGCAGGAGATTCGGCACTACCAGAAGACGTGCGACTTGCTCATCCCCCGACTGCCGTTCGCTCGCTATGTGAAGGAAATCACGTCTATGTATGCGAGTGACGTTTCTCGATGGACGGCGGAGGCTTTGACAGCCCTTCAAGAGGCAACAGAAGACTACATGGTGCATTTGTTTGAAGACACGAATTTGTGTGCCATCCATGCGAAGCGAGTCACCATAATGCCGAAAGATCTGCAGTTAGCCAGACGACTACGAG
GTGTCATCGAAAAAACATCA
>Rhynchostegium_serrulatum
ATGGCGAGGCGGAAGTCGACTCCTCTGCACGGCAACAAACGCGCACCCACGCGCCCATCT---------GCTGCT------GGTGCTTCTTCT---------TCT---------------
ACGACGAGGCCGAGGAAAGCGCACCGATGGAGACCGGGCACCAAGGCGTTGCAGGAGATTCGGCACTACCAGAAGACGTGCGACTTGCTCATCCCACGACTGCCGTTTGCTCGCTATGTGAAGGAAATCACGTCAATGTATGCGAGTGACGTTTCTCGATGGACGGCGGAGGCTTTGACAGCCCTTCAAGAGGCAACAGAAGACTACATGGTGCATTTGTTTGAAGACACAAATTTGTGTGCCATCCATGCGAAGCGAGTCACCATAATGCCGAAGGATCTGCAGTTAGCCAGACGACTACGAG
GTGTCATCGAAAAATCATCA
>Leucodon_brachypus
ATGGCGAGGCGGAAGTCGACCCCTCTGCACGGAGAAAAACGCGCACCTACGCGC---TCG---------GCAGCT------GCTGCTGCTGCT---------TCT---------------
ACGACGAGGCCCAGGAAAGCGCACCGATGGAGACCAGGCACCAAGGCGTTGCAGGAGATTCGGCACTACCAGAAGACGTGTGACTTGCTCATCCCCCGACTGCCGTTTGCTCGCTATGTGAAGGAAATCACGTCTATGTATGCGAGTGACGTCTCTAGATGGACGGCGGAGGCTTTGACAGCCCTTCAAGAGGCAACAGAAGACTACATGGTGCACTTCTTTGAAGACACTAATTTGTGTGCCATCCATGCGAAGCGAGTCACCATAATGCCGAAGGATCTGCAGTTAGCCAGACGACTACGAGG
AGTCATCGAAAAAGCATCA
>Thuidium_delicatulum
ATGGCGAGGCGGAAGTCGACCCCTCTGCACGGCAACAAACGCACTCCCACTCGC---TCA---------GCTGCC------GCTGCTGCTGCT---------TCT---------------
ACGTCGAGGCCGAGGAAAGCGCACCGATGGAGACCGGGCACCAAGGCGTTGCAGGAGATTCGGCACTACCAGAAGACGTGCGACTTGCTCATCCCCCGACTGCCGTTTGCTCGCTATGTGAAGGAAATCACGTCTATGTACGCAAGTGACGTTTCTAGATGGACGGCGGAGGGTNTNACAGCCCTTCAAGAGGCAACAGAAGACTACATGGTGCATTTGTTTGAAGACACAAATTTGTGTGCCATCCATGCGAAGCGAGTCACCATAATGCCGAAGGATCTGCAGTTAGCCAGACGACTACGAG
GTGTCATCGAAAAAGCATCA
>Rosulabryum_capillare
ATGGCGAGGAGGAAATCGACTCCTCGGCATGGCCGCAACCGGGCGTCCACGAGC---GCTGCTGGTGGAGCTGNA------GCAGCTGCTGCT---------GCT---------------
GCGCGGCAACGAAGGAGACCGCATCGATGGAGACCAGGCACCAAGGCACTCCAAGAGATTCGGCATTACCAAAAGACGTGCGACCTCCTCATTCCTAGACTTCCGTTCGCGCGCTATGTGAAAGAAATCACGTCTATGTACGCAAGTGATGTGTCTCGGTGGACGGCGGAAGCTTTAACAGCCCTTCAAGAAGCAACTGAAGACTACATAGTNCATTTGTTTGAAGACACGAAT--
>Bryum_argenteum
ATGGCGAGGAGGAAATCGACTCCTCGGCATGGCCGGAACCGGGCGTCCACGAGC---GCTGCTGGTGGAGCTGCA------GCAGCTGCT------------GCT---------------
GCGCGGCAACGAAGGAGACCGCATCGATGGAGACCAGGCACCAAGGCACTCCAAGAGATTCGGCATTACCAAAAGACATGCGACCTCCTCATACCTAGACTTCCGTTCGCGCGCTATGTGAAAGAAATCACGTCTATGTATGCAAGTGATGTGTCTCGGTGGACGGCTGAAGCTTTAACAGCCCTTCAAGAAGCAACTGAAGATTACATAGTGCATTTGTTTGAAGACACGAATTTGTGCGCTATCCACGCTAAGCGAGTCACCATAATGCCAAAGGACTTGCAATTGGCGAGACGGCTACGCGGT
GTGATTGAGAAAACAGTA

Fabaceae – CenH3 alignment before masking unreliable residues inferred in Bali-Phy

```
>Cicer_pinnatifidum
ATGGCTAGAGTTAAGCACATTCCTCCTCCTCGTAAT------------------------CGCGCT--------------------GTA------------------------------------------------------------------------------------------------------------------------------------------------------------ --------
AGTGAGGATCAGGAACATAAGAAAAGACGCAATAAGCCTGGAACAGTGGCGCTTCGCGAGATTCGTCGTTTTCAAAAGACTTTCAATTTGCTTTTACCAGCTGCTCCATTTATAAGATGCGTCAAACAGATTACGAACCAAACATCTTCACATGTCTCACGTTGGTCGCCGGAAGCAGTAATAGCACTTCAGGAGGCGGCTGAGGATTATCTGGTACATATGTTTGAAAATGGAATGCTATGTGCACTTCATGCAAGGCGTATTACCCTTATGAAAAAGGATATTGAGTTGACCCGTAGGCTTACAGG
AATAGGAAGGCCTTGG
>Cicer_yamashitae
ATGGCTAGAGTTAAGCACATTCCTCCTCCTCGTAAC------------------------CGCGCT--------------------GTA------------------------------------------------------------------------------------------------------------------------------------------------------------ --------
AGTGAGGATCAGGAGCATAAGAAAAGACGCAATAAGCCTGGAACGGTGGCGCTTCGCGAGATTCGTCGTTTTCAAAAGACTTTCAATTTGCTTATACCAGCTGCTCCATTTATAAGATGCGTCAAACAGATTACAAACCAAACATCTTCACATGTCTCACGTTGGTCGCCGGAAGCAGTAATAGCACTTCAGGAGGCGGCTGAGGATTATCTGGTTCATATGTTTGAAAATGGAATGCTCTGTGCACTTCATGCAAGGCGTATTACCCTTATGAAAAAGGATATAGAGTTGACCCGTAGGCTTACAGG
AATAGGAAGGCCTTGG
>Cicer_judaicum
ATGGCTAGAGTTAAGCACATTCCTCCTCCTCGTAAT------------------------CGCGCT--------------------GTA------------------------------------------------------------------------------------------------------------------------------------------------------------ --------
CGTGAGGATCAGGAACATAAGAAAAGACGCAATAAGCCTGGAACAGTGGCGCTTCGTGAGATTCGTCGTTTTCAGAAGACTTTCAATTTGCTTTTACCAGCTGCTCCATTTATAAGATGCGTCAAACAGATTACGAACCAAACATCTTCACATGTCTCACGTTGGTCGCCGGAAGCAGTAATAGCACTTCAGGAGGCGGCTGAGGATTATCTGGTACATATGTTTGAAAATGGAATGCTATGTGCACTTCATGCAAAGCGTATTACCCTTATGAAAAAAGATATTGAGTTGACCCGTAGGCTTACAGG
AATAGGAAGGCCGTGG
>Cicer_reticulatum
ATGGCTAGAGTTAAGCACATTCCTCCTCCTCGTAAT------------------------CGCGCT--------------------GTA------------------------------------------------------------------------------------------------------------------------------------------------------------ --------
AGTGAGGATCAGAAACATAAGAAAAGACGCAATAAGCCTGGAACAGTAGCGCTTCGCGAGATTCGTCGTTTTCAAAAGACTTTCAATTTGGTTATACCAGCTGCTCCATTTATAAGATGCGTCAAACAGATTACGAACCAAACATCTTCACATGTCTCACGTTGGTCGCCGGAAGCAGTAATAGCACTTCAGGAGGCGGCTGAGGATTATCTGGTTCATATGTTTGAAAATGGAATGCTATGTGCCCTTCATGCAAGGCGTATTACCCTTATGAAAAAGGATATTGAGTTGACCCGTAGGCTTACAGG
AATAGGAAGGCCTTGG
>Cicer_bijugum
ATGGCTAGAGTTAAGCACATTCCTCCTCCTCGTAAT------------------------CGCGCT--------------------GTA------------------------------------------------------------------------------------------------------------------------------------------------------------ --------
AGTGAGGATCAGGAACATAAGAAAAGACGCAATAAGCCTGGAACAGTGGCGCTTCGCGAGATTCGTCGTTTTCAGAAGACTTTCAATTTGCTTTTACCAGCTGCTCCATTTATTAGATGCGTCAAACAGATTACGAACCAAACATCTTCACATGTCTCACGTTGGTCGCCGGAAGCAGTAATAGCACTTCAGGAGGCGGCTGAGGATTATCTGGTACATATGTTTGAAAATGGAATGCTATGTGCACTTCATGCAAGGCGTATTACCCTTATGAAAAAAGATATTGAGTTGACCCGTAGGCTTACAGG
AATAGGAAGGCCGTGG
>Pisum_sativumB
ATGGCGAGAGTTAAACAAACACCACGTCAC--------------------------------------------------------GCC---------------------------------------------------------------------------------------------------------------------------------------------------- --------
CGTGAGAATCAGGAAAGGAAGAAAAGACGTAATAAACCTGGAACCGTAGCGCTTCGTGAGATCAAGAAATTGCAAAAAACTTTCCAATTACTTATACCATATGCTCCGTTTGTAAGATGCGTCAGGGAAATTACAAATCAAGTATCTTCATTGGTTACACGCTGGACGCCTGAAGCGTTGCTATCACTTCAAGAGGCAGCTGAGGATTGTCTAGTTCGAATGTTTGAAGCTGGATGGCTCTGTACACTTCATGCAAAGCGTGTTACCCTTATGAAAAAGGATATTGAGTTAACGCGCAGGCTTACCGG
GATAGGAAGACCTTGG
>Astragalus_sinicus
ATGGCGAGAGTTAAAATCAAACCATCTCCTCGTAAA------------------------CGC----------------------GCA---------------------------------------------------------------------------------------------------------------------------------------------------------- --------
AGTGAGAGTCAGGGGAAGAAAAAAAGGCGCTATAAACCTGGAACAGTAGCACTTCGTGAGATTCGTCATTTCCAAAAGTCTGTCAATTTACTCATACCGGCTGCTCCTTTTATAAGATGTGTCAAACAGATTACAAATAACTTTTCTACAGAAGTCTCGCGCTGGACACCTGACGCCGTGTTAGCACTTCAAGAGGCAGCTGAGGATTATCTGGTTAAATTGTTTGAAGACGGGATGCTCTGTGCAATTCATGCAAGGCGTGTTACCCTTTTGAAAAAGGATTTTGAGTTGGCGCGGAGACTTGGAGG
AATAGGAAGGCCTTGG
>Cajanus_scarabaeoides
ATGGCGAGAGTGAAGCACACGCCAGCTTCTCGCAAGCCTGGTAGGAAGAACAAAGCGCCG-----------------------------------------------------------CCAGAATCACCG------CAA-----------GCTCAATCGCCCGCAACTAGAGAGAGGAGGAGGAGAGCTGAACAAGAGTTGCCGCAGGAGAAT---------------------GAAGCA---------GCGGCA------
AGGACTCACGGAAGAAAGAAAAGGCGCAGTAAGCCGGGAACTGCGGCGCTTCGCGAGATTCGTCATTATCAGAAGTCTTGCCAGCTTCTTATCCCAGTCGCACCCTTTATTAGATGCGTCAAAGAGATAACGCATCAATACTCTACGGAGGTGTCTCGTTGGACACCCGAAGCTGTGTTAGCACTTCAGGAGGCAGCTGAGGAATATCTGGTTCACTTGTTTGAAGACGGAATGCTCTGTGCAATTCATGCAAAGCGTGTTACTCTTATGAAAAAGGATATTGAGTTGGCTCGGAGGCTTGGAGGAA
TAGGAAGGCCT---
>Cajanus_cajanifolius
ATGGCGAGAGTGAAGCACACGCCAGCCTCTCGCAAGCCTGGTAGGAAGAACAAAGCGCCG-----------------------------------------------------------CCAGAATCACCG------CAA-----------GCTCAATCGCCCGCAACTAGAGAGAGGAGGAGGAGAGCTGAACAAGAGTTGCCGCAGGAGAAT---------------------GAAGCA---------GCGGCA------
AGGACTCACGGAAGAAAGAAAAGGCGCAGTAAGCCGGGAACTGCGGCGCTTCGCGAGATTCGTCATTATCAGAAGTCTTGCCAGCTTCTTATCCCAGTCGCACCCTTTATTAGATGCGTCAAAGAGATAACGCATCAATACTCTACGGAGGTGTCTCGTTGGACACCCGAAGCTGTGTTAGCACTTCAGGAGGCAGCTGAGGAATATCTGGTTCACTTGTTTGAAGACGGAATGCTCTGTGCAATTCATGCAAAGCGTGTTACTCTTATGAAAAAGGATATTGAGTTGGCTCGGAGGCTTGGAGGAA
TAGGAAGGCCTTGG
>Cajanus_cajan
ATGGCGAGAGTGAAGCACACGCCAGCTTCTCGCAAGCCTGGTAGGAAGAACAAAGCGCCG-----------------------------------------------------------CCAGAATCACCG------CAA-----------GCTCAATCGCCCGCAACTAGAGAGAGGAGGAGGAGAGCTGAACAAGAGTTGCCGCAGGAGAAT---------------------GAAGCA---------GCGGCA------
AGGACTCACGGAAGAAAGAAAAGGCGCAGTAAGCCGGGAACTGCGGCGCTTCGCGAGATTCGTCATTATCAGAAGTCTTGCCAGCTTCTTATCCCAGTCGCACCCTTTATTAGATGCGTCAAAGAGATAACGCATCAATACTCTACGGAGGTGTCTCGTTGGACACCCGAAGCTGTGTTAGCACTTCAGGAGGCAGCTGAGGAATATCTGGTTCACTTGTTTGAAGACGGAATGCTCTGTGCAATTCATGCAAAGCGTGTTACTCTTATGAAAAAGGATATTGAGTTGGCTCGGAGGCTTGGAGGAA
TAGGAAGGCCTTGG
>Vigna_unguiculata
ATGGCGAGAGTGAAGCACACGCCAGCTTCGCTCAAAGTTGGT---AAAAAAAAAGTCAGTCGCGCT------------------------------TCCACATCCACG-----------CCA--------------CAG--------------CAATCGCCTGCGACAAGAAGTCGT---AGAAGGGCTCAAGAAGAGGAGCCGCAGGAA------------------------GCAGCA---------
GCAGCAGCGCCACAGACTCAGGGAAGGAAGAAAAAGCGCAGTAAGCCAGGAACAGCGGCGCTTCGCGAGATTCGTCATTTTCAGAAGAGTTGCAAGCTTCTTATCCCGGCTGCCCCCTTTATCAGATGTGTCAAACAAATTACACATCAATTCTCTACGGAGGTGTCTCGCTGGACGCCTGAAGCTGTGGTAGCACTGCAGGAGGCAGCTGAAGAATGTCTAGTTCACTTGTTTGAAGATGGAATGCTTTGTGCAATTCACGCTAGGCGTATTACTCTTATGACAAAGGATATTCAGTTGGCTCGGA
GGCTTGGAGGAATAGGAAGGCCTTGG
>Glycine_max
ATGGCGAGAGTGAAGCACACGCCAGCTTCTCGCAAATCCGCT---AAAAAGCAAGCGCCACGCGCA------------------------------TCCACTTCCACGCAGCCG------CCA------CCA------CAA-----------TCCCAATCGCCTGCAACTAGAGAGAGG---AGGAGAGCTCAACAAGTGGAGCCGCAGCAG------------------------GAG---------------------
CCTGAGGCTCAGGGAAGGAAGAAGAGGCGCAATAGGTCGGGAACTGTGGCGCTTCGTGAGATTCGGCATTTTCAGAGGAGTTGTGAGCTTCTCATCCCGGCTGCTCCCTTTATCAGATGTGTCAAACAGATTACAAATCAATTCTCTACGGAGGTGTCTCGTTGGACACCTGAAGCTGTGGTAGCACTTCAGGAGGCAGCTGAGGAATATCTGGTTCACTTATTTGAAGATGGAATGCTCTGTGCAATTCATGCAAGGCGTATTACTCTTATGAAAAAGGACATTGAGTTGGCCCGGAGGCTTGGAG
GAATAGGAAGGCCTTGG
>Medicago_truncatula
ATGGCAAGAGTCAAGCACATTCCACGTCCTGGTAAA------------------------CGCACTCGTCGCAGTAGTAGTAGTAATGCA------------------------------------------------------------------------------------------------------------------------------------------------------------ --------
AATGAGTCTGAGGAAAAGAAGAAAAGAAGGAATAGACCAGGAACAGTTGCGCTTCGCGAGATTCGTAAATTTCAAAAGGCTGTTAACTTGCTTATACCTTGTGCTCCGTTTGTTAGATGCGTCAAACAGATTACAAACCAACTATCTATGGAGGTATCACGTTGGACGGCTGAAGCCTTATTAGCACTTCAGGAGGCAGCTGAGGAGCATCTGGTTCGTATGTTTGAAGGTGGGATGCTCTGTGCACTTCATGCAAAGCGTGTTACCCTTATGAAAAAGGATCTTGAGTTGACCCGTAGGCTTACAG
GAATAGGATGCGCTCGG
>Lotus_japonicus
ATGGCGAGAATCAAGCACGTTCCTGCTGCTCGTAGAACCGCT---CGAAAGAAAGCACCAACCGCA--------------------GCAGCACCATCCACATCTGCA------ACA---CCA------CAA------CAA-----------ACCCAATCACCTGGTCGTCGAGAGAGG---AGTAGAGAACAACAACGGGAACCGCAG------------------------------------------------
TCACCTGGGACTCAGGGGAAGAAGAAGAGGCGCAATAGGCCGGGAACGGTGGCGCTTCGTGAGATCAGGCGTTATCAGAAGAGTGTAGACTTGCTTATCCCTGCTGCCCCCTTTCTGAGATTGGTTAGAGAGACTACACGTCAGTTATCTTTTGAGGTTTCTCGCTGGACAGCTGAAGCTGTGGTGGCTCTGCAGGAGGCAGCGGAGGACTATCTTGTTACTATGTTTGAAGATGGAATGCTCTGCGCAATTCATGCAAAGCGCGTTACCCTTATGAAAAAGGATATTGAGTTGGCCCGTCGGCTT
CGAGGAATAGGAAGGCCTTGG
>Phaseolus_vulgaris
ATGGCGAGAGTGAAGACCACGCCAGCTTCGCGCAAAACCGGT---AAAAAAAAAGCCGCACGCGCT------------------------------TCCACGTCCACG-----------CCA---------CGGTCACAG--------------CAATCGCCT---------GGGAGG---AGAAGGGCTCAACAAGAGGAGCCGCAGGAA---GCAGAAGAAGAAGAAGAA---GAAGCA---------
GCAGCAGCGCCCGAGACTCAGGGAAGGAAGAAAAGGCGCAATAAGGCAGGAACAGTGGCGCTTCGCGAGATTCGTCAATTTCAGAGGAGTTCCAAACTTCTTATCCCGGCTGCCCCTTTTATGAGATGTGTCAGACAAATTACACAGCAATTCTCTGCGGAGGTGAGTCGCTGGACACCTGAAGCAGTGGTAGCACTCCAGGAGGCAGCTGAGGAATGTCTAGTTCACTTGTTTGAAGATGGAATGCTTTGTGCAATTCACGCAAGGCGTGTTACTCTTATGACAAAGGATATTCAGTTGGCTCGG
AGACTTGGAGGAATAGGAAGGCCTTGG
>Arachis_appressipila
ATGGCAAGAGTGAAGCATATTCCAACACCTAGTCAAAAAGGT---AAGAAAAAAGTA------------------------------------------------------------AGACCA------TCA------CAATCTCCATCGCCATCGCAAGCGTCTGGTAGCAGA---AGG---AGGGAAGATGGAGAAGAGGAG---CAGGAA---------------------CCAGAAGCAGGACGACGATCAGCAGCACCT---------------
AAGAAAAGGCGTAATAAGCCAGGAACAGTAGCTCTTCGTGAGATTCGTAAATTTCAGAAGAGTTTCAACCTACTCATCCCAGCTGCCCCCTTCATGAGATGTGTCAAACAGATTACAAACCAACTATCTACGGAGGTCAATCGCTGGACAGCTGAAGCCATGGTAGCACTTCAAGAAGCAGCTGAGGATCATCTGGTTCGTTTGTTTGAAGATGGAATGTTGTGTGCTATCCATGCAAAGCGTGTTACTCTAATGAAAAAGGACATAGAGTTGGCCCGGAGACTCGGAGTGATAGGAAGACCTTGG
>Lens_culinaris
ATGGCGAGAGTTAAACAAACACCACGTCCC--------------------------------------------------------GTA---------------------------------------------------------------------------------------------------------------------------------------------------- --------
CTTCAGAATCAGGAAAGGAAGAAAAGACGGAATAAACCTGGAACCGTAGCGCTTCGTGAGATCCGGAAACTTCAAAAAACTTTCCAATTACTTATACCATATGCTCCCTTTGTAAGATGTGTCAGGGAAATTACAAACCAAGTATCTTCTTCGGTTACGCGCTGGACGCCGGAAGCATTGATTTCACTTCAAGAGGCAGCTGAGGATTGTCTAGTTCGAATGTTTGAAGCTGGATGTCTCTGTGCACTTCATGCAAAGCGTGTTACCCTTATGAAAAAGGATATTGAATTGACGCGAAGGCTTACCGG
GGTAGCAAGACCTTGG
```

Ferns – CenH3 alignment before masking unreliable residues inferred in Bali-Phy

```
>Ceratopteris_richardii
ATGGCGAAGAAAAAGAAGACCCCGAAAAAGGCTTCCCAGGTCGCTTCTTCCTCTACCGTCCCTCAGCCTGAGATCACAGATGCAGAGAACAGCAGAAGACGACTCCATCGAAGCAGTAGT--------------------GCACCTCCAACTGCTCCA------------------------------------------------------------------------------------------GTTACT------------------------------------------------------------------------------------------------------------------------------------------------------------------------------------------
CCAACAAATGAACGAGAGCCTACAGACAATACACCTTCCAGGCGTCCACGCAAACCCCACAGATTTCGTCCAGGAACAGTAGCTCTTCGGGAAATTCGACATTATCAGAGAACTGTTAATTTTCTCATACATCCATTGCCATTTGCCAGATTGGTGAGAGAAATTGGGGTCCAATTTTCAGATACTGTCTCTCGGTGGACTGCAGAATCACTTCTAGCATTACAGGAGGCTGCTGAGGATCACATTGTACACCTATTTGAGGATACAAACCTATGTGCCATACATGCTAAGCGTGTCACCATAATGCCAA
AGGATATGCAATTAGCAAGGAGACTTAGAGCAAGTACCCTTGACAGGCCTTGG
>Ophioglossum_petiolatum
ATGGCTCGAAGGAAGCCGAGGCCGCAAAAAGCA--------------------------------------------------------------------------------------------------------------GCTCCGCCGGCCGCTACTACTTCAACAGCCGTCGCACCATCGCCACAGCCAGTAGGCGGTCGTAAAGGAAGTCGTAAAACAGCCGGCAGACGCCGTGCTACCGCGCCTACT------------------------------------------------------------------------------------------------------------------------------------------------------------------------------------
---CCC---------------------------------------------
AAAAAGCCCCACCGTTTCAAGCCCGGTACCGTTGCTTTGAGAGAGATCAAATATTACCAGAAGAAATTTCACCTCCTCATTCGTCGTCTTCCCTTTGCAAGATTAGTTAAAGAAATTACGGCTCATTTTTCACGCAATGTTACTCGCTGGACAGCGGAGGCGCTGACTGCTTTGCAAGAGGCAGCTGAAGACCAAATTGTTCATCTTTTTGAGGACACCAATTTATGTGCCATCCACGCAAAGCGCGTTACAATAATGCCAAAGGATATGCAACTTGCAAGGCGTCTACGTGGGAACACAGCTGACAGG
CCATTT
>Azolla_filiculoides
ATGGCGAGACTCAAGCAAAGACCACACAGGTCTAATCTGGCTGCATTGTCGTCCAATGTT---------GAAACTACA------GGAAGTAGCCAGCCCCATTTGAATCGCAGTAATAGT--------------------GCGCCACCAACTGCTCCC----------------------------------------------------------------------------------ACAACA--------------------------------------------------------------------------------------------------------------------------------------------------------------------------------
CCATTCAAC------------------
AATATTCCTGCTAATCGCCAACGCAAACCTCATCGATTTAGGCCTGGTACAGTGGCTTTAAGAGAAATCAGGCGCTACCAAAAGACTGTTCACTTTCTTATCCATCCCTTACCATTTGCACGACTGGTCAGGGAACTAACTGCCCAATTTTCAGACACAGTGAGCCGATGGACAGCTGAGGCATTGGTTGCTTTGCAAGAGGCTGCTGAGGATCATTTGGTGCATTTGTTTGAGGACACTAATTTATGTGCTATCCATGCAAAACCTGTCACAATAATGCCTAAAGATATGCAGCTTGCAAGACGTTTGC
GTGGCAACACCATTGACAGGCCTTGG
>Lygodium_japonicum
ATGGCCAGAAGGAAGCCTAATCCAAAGAAGGCCACGCCAGTTGCTTCTTCTTCGAACGCCACACCGCAGGAGGCGACA------GCAAGCAGCAGACCGCGCTTAGTTAGAAGCAGTAGT--------------------GCACCACCGACAACCCCA-------------------------------------------------------------------------------------------GCAACA------------------------------------------------------------------------------------------------------------------------------------------------------------------------------------
CCCTCAAAT------------------GCTCCTCCTGCT---
CGACAGCGCAAGCCTCATCGGTTTAGACCCGGAACTGTTGCTTTGCGGGAAATCAGGCATTTTCAGAAGACAGTTCACTTTCTTATTCATCCATTACCATTTGCACGGCTGGTTAGAGAAATTGCAGCTCAATGTTCGGATACAGTAACTCGATGGACTGCCGAAGCATTGGTTGCTTTGCAGGAGGCTGCAGAAGACCACCTTGTACACTTGTTTGAGGACACTAATCTGTGTGCTATTCATGCAAAACGAGTCACAATAATGACAAAAGACATGCAACTTGCACGGCGTTTACGAGGCAGCACTCTA
GATAGGCCGTGG
>Pteridium_aquilinum
------------------------------------------------------------------------------------------------------------------------------------------------------------------------------------------------------------------------------------------------------------------------------------------------------------------------------------------------------------------------------------------------------------------------------------------------------------------------------------------------------------------------------------------------------------------------------------------------------
AACCGCCAACGCAAGCCCCATCGGTTTCGCCCTGGAACTGTAGCTCTCCGAGAGATTCGACATTACCAAAAGACTGTCAATTTTCTCATTCATCCCCTGCCCTTTGCCAGATTGGTGAGAGAACTGGCCGGTCAGTTTTCAGACTCAGTATCCCGGTGGACTGCAGAGGCACTGGTTGCGCTACAGGAGGCTACTGAGGACCACCTTGTGCACCTTTTTGAGGACACTAATTTATGCGCAATTCATGCCAAACGTGTCACAATAATGACGAAAGACATGCAACTAGCAAGGCGTTTGCGAGGAACCACT
CTGGACAGGCCTTGG
>Psilotum_nudum
--------------------------------------------------------------------------------------------------------------------------------------------AGTCAAACCGCGAGACGCACCGCTCCTTCAACATCTACC------------------------------------------------------------------------------------------------------CCCGCC----------------------------------------------------------------------------------------------------------------------------------------------------------------------------------------TCT---------------------------------------------
AGAAAGCGTCATCGGTTTCGGCCTGGAACAGTGGCATTACGAGAAATTAGATTTTATCAGAAGCGCTTTCATCTTTTAATTCCACCACTGCCTTTTGCCCGACTGGTCAAGGAAATGACATTATATTTTTCTCGCGATGTGTCCCGCTGGACGGCCGAGGCATTAGTGGCGTTGCAGGAGGCTGCTGAGGATTTCATCGTTCATCTGTTTGAGGACACCAATTTATGTGCTATTCATGCAAGACGTGTCACAATAATGACCAAAGACATGCCGCTTGCCAGGCGCTTGCGTGGAGCCATTGCTGATAGG
CCCTGG
>Equisetum_diffusum
ATGGCGAAGAAGAAA------------------------------------------------------------------------------------------------------------------------------------------------------------------------------------------------------------------------------------------------------------------------------------------------
AGTGCGCCCGCGCGCTCCCAAAAGTCCCCTGCTTCCACAGTCGTCGACGGAAATGGGCAGCCTTCCAGTAAGAATGCAAAGGATCAAGGTGGGCCTTCAAGCACTCCCGATACTGCAAATGTTGGTGGCAGAGGAGTTAAAGGGGCAAAGGGAAAAGGAAGAACACAGCAGGGTGCCCAAAACCCT---------------------------------------------
CGGAAGTCACACCGTTACAAAGCCGGAACGGTTGCACTGCGGGAAATCAGATTCTACCAGAAGAATGTAGATCTACTCATTCGGCCTCTCCCCTTTGCACGCTTGGTGCGAGAAATTGCTGATCACCTCTCTCGGACTGTTACTCGCTGGACAGCTGAAGCCTTGGTGGTTATGCAAGAGGCATGCGAGGATTTTATTGTCCATCTTTTTGAGGACACCAATCTTTGTGCAATTCATGCCAAGCGCGTAACTATAATGACTAAGGACATGCAACTTGCTAGGCGGCTCCGTAATACAATGATTGATAGG
CCGTGG
>Equisetum_arvense
ATGGCGAAGAAGAAA------------------------------------------------------------------------------------------------------------------------------------------------------------------------------------------------------------------------------------------------------------------------------------------------
AGTGCTCCCGCGCGCTCCCAAAAGTCCCCTGCTTCCACAGTCGTCGACGGAAATGGGCAGCCTTCCAGTAAGAATGCAAAGGATCAAGGTGGGCCTTCAAGCACTCCCGATACTGCAAATGTTGGTGGCAGAGGAGTTAAAGGGGCAAAGGGAAAAGGAAGAACACAGCAGGGTGCCGAAAACCCT---------------------------------------------
CGGAAGTCACACCGTTACAAAGCCGGAACGGTTGCACTGCGGGAAATCAGATTCTACCAGAAGAATGTAGATCTACTCATTCGGCCTCTCCCCTTTGCACGCTTGGTGCGAGAAATTGCTGATCACCTCTCTCGGACTGTTACTCGCTGGACAGCTGAAGCCTTGGTGGTTATGCAAGAGGCATGCGAGGATTTTATTGTCCATCTTTTTGAGGACACCAATCTTTGTGCAATTCATGCCAAACGCGTAACTATAATGACTAAGGACATGCAACTTGCTAGGCGGCTCCGTAATACAATGATTGATAGG
CCGTGG
```

Lycopodiophyta – CenH3 alignment before masking unreliable residues inferred in Bali-Phy

>Isoetes_sinensis
ATG--------------------CCTAGAAAGAAAAGTACCCCAAAAACGGGCAAAGCCTCTACTCCTGCAGCTTCTTCTTCAGCTACAGGAACCAGTTCTAGAAGAGTA------------------------------GATGGTGCGAGCACTAGTAGGGCAGGAAATGCACGTAGTTCGGATAGGGTTGTAGCT--CCTGTGCAAAAAAGG------AAG---CAC---------
CGGTTTAAACCTGGAACTGTGGCGCTTAGGGAAATAAGGCATTTTCAAAAGAGCTACGGATTTCTTCTCAGGCCTTTGCCATTTGCCAGAGTGGTGAGAGAGATAACTGCCCAG------TATTCTAAAGAAGTGTCAAGATGGACTGCTGAAGCTTTAATAGCTATTCAAGAGGCTGCTGAAGACTACCTTGTACATCTTTTTGAAGACACAAATTTGTGTGCTATTCATGCCAAAAGAGTCACTATCATGCCAAAAGATTTGCAACTTGCACGTCGTTTACGAGGGGGTACGGAAAGAAAGATGTAC---
>Selaginella_kraussiana
--GGTGGCGATGGCGCCGGGACGAGCAGAGCAGGT------------------------------GCCCAGCAGAAC--AGACCA------
AGACGGCGTTTCAAGGCCGGGACTGTAGCGCTTCGCGAAATTCGGAAGTTTCAGAAGAGCTTTGAGCTCCTCCTCAGGCCTCTACCCTTCGCCAGAGTGGTTCGGGAGCTTGCATCCTTG------TGTTCAACTGAGGTTACTAGATGGACTGCTGAATCCCTCCTTGCTCTCCAAGAGGCTGCGGAGGATTACTTGGTGCATTTGTTTGAAGACACCAACTTGTGTGCGATACATGGAAAACGGGTTACGATA--
>Selaginella_stauntoniana
ATGGCCAAGAGGAGGAAATCGACGCCGAGGAAG---CGAGGGCGACCACCA---------GCGGGCGGCGATGGCGCTGGGACGAGCAGAGCCGGC------------------------------GCCCAGCAGAAC--
AAGCCGCACAGCAAGCGCAGGTTTAAGCCCGGGACTGTGGCGTTGCGGGAGATCCGACGCTTTCAAAAGAGCTATGAGCTCCTTCTCAGGCCATTGCCATTCGCAAGAGTGGTGAGAGAGATCACG------
AACGTTTTCTCGTCGGAGGTATCCAGATGGACTGCCGAAGGATTGATAGCTCTCCAAGAGGCTGCCGAGGACTACCTTGTTCATCTCTTCGAAGACACCAACTTATGTGCCATTCACGGCAAGAGAGTCACCATAATGCCCAAGGACTTGCATTTGGCCCGTCGACTAAGAGGAGCCTCCGAGCGG------TTCGTT
>Selaginella_moellendorffii
ATG---GAGTGCGCAGCGGGCGGCGATGGCGCTGGGACGAGCAGAGCCGGC------------------------------GCCCAGCAGAAC--
AAGCCACACGGCAAGCGCAGGTTTAAGCCCGGGACTGTGGCGTTGCGGGAGATCCGACGCTTTCAAAAGAGTTATGAGCTACTTCTCAGGCCATTGCCATTCGCAAGAGTGGTGAGAGAGATCACG------
AACGTTTTCTCGTCGGAGGTATCCAGATGGACTGCCGAAGGATTGATAGCTCTCCAAGAGGCTGCCGAGGACTACCTTGTTCATCTCTTCGAAGACACCAACTTATGTGCCATTCACGGCAAGAGAGTCACCATAATGCCCAAGGACTTACATTTGGCCCGTCGACTAAGAGGAGCCTCCGAACGG------TTCGTT
>Lycopodiella_caroliniana
--GCTACCCCAGTTTCTGAGTCGGCAAGCAGTAGAAGAGCTGTTGGAGAACGTCAAAGACAAGCGCCTGCT---------CAGCGAAAGCCTCAT---------
CGGTTCAAGCCTGGTACTGTGGCTCTCAGAGAGATTCGAAAGTATCAAAAAAGTTTCAATCTCTTGATAAGGCCCTTGCCATTTGCACGACTGGTTCGTGAAATCACATCTCAA------TTCTCTACTGATGTTACAAGATGGACAGCAGAAGCTCTGATTGCCATTCAAGAGGCTGCTGAGGATTACCTTGTTCATCTTTTTGAAGATACCAACTTATGTGCCATTCATGCCCGTCGGGTGACAATTATGCCAAAGGATCTGCATCTTGCGCGACGTCTTCGAGGAGCTTCTGAG---AAAATGTTCATC

Poaceae – CenH3 alignment before masking unreliable residues inferred in Bali-Phy

```
>Zea_mays
ATGGCTCGAACCAAGCACCAGGCCGTGAGGAAGACG---------GCGGAGAAGCCCAAGAAGAAGCTCCAGTTCGAGCGCTCA------------------------------------GGTGGT---GCGAGTACCTCG------GCGACGCCGGAAAGG------GCTGCTGGGACCGGGGGAAGAGCGGCGTCTGGAGGTGACTCA--------------------------
GTTAAGAAGACGAAACCACGCCACCGCTGGCGGCCAGGGACTGTAGCGCTGCGGGAGATCAGGAAGTACCAGAAGTCCACTGAACCGCTCATCCCCTTTGCGCCTTTCGTCCGTGTGGTGAGGGAGTTAACCAATTTC-------------------------------------
GTAACAAACGGGAAAGTAGAGCGCTATACCGCAGAAGCCCTCCTTGCGCTGCAAGAGGCAGCAGAATTCCACTTGATAGAACTGTTTGAAATGGCGAATCTGTGTGCCATCCATGCCAAGCGTGTCACAATCATGCAAAAGGACATACAACTTGCAAGGCGTATCGGAGGA---AGGCGTTGGGCA---
>Hordeum_vulgare
ATGGCCCGCACCAAGCACCCCGCCGTGAGGAAGTCC---------AAGGCGCCGCCCAAGAAGAAGATCGGGTCCGCTAGCTCCCCGAGC------GCG---GCGCAGCGCCGGCAGGAGACAGATGGC---GCCGGCACGTCC------GAGACTCCGAGGCGG------GCCGGGCGGGGGCCGGCCCCAGCGGCGGCTGAAGGG------GCACCTGGGGAACCG---ACGAAG------AGGAAGCCA---------
CACCGGTTCAGGCCAGGCACGGTGGCACTGCGGGAGATCAGGAAGTACCAGAAGTCGGTCAATTTTCTCATCCCGTTTGCACCGTTTGTTCGTCTGGTCAGGGAGATCACCGAATACTACTGTCCTCGA--------------------------GTCAAACGCTGG--------------ACTCCCCAGGCGCTCCTCGCAGTTCAAGAGGCTACAGAGTATCACCTCGTCGACATATTTGAAAGGGCACATCTCTGTGCCATCCATGCAAAGCGTGTTACCGTCATGCAAAAGGACATGCAACTCGCG------------------------
--------
>Hordeum_bulbosum
ATGGCCCGCACCAAGCACCCCGCCGTGAGGAAGTCC---------AAGGCGCCGCCCAGGAAGAAGGTCGGGTCCGCGCGCGCCCCGGCC------GCG---GCGCAGCGCCGGCACGAGACAGATGGC---GCGGGCACGTCC------GAGACTCCGAGG--------------CGGGGGCCGGCCCCGGCGGCGGATCAAGGG------GCACCTGGGGAACCC---AAGAAG------AGGAAGCCA---------
CACCGGTACAGGCCAGGCACGGTGGCACTGCGGGAGATCAGGAAGTACCAGAAGTCGGTCGATTTTCTCATCCCGTTTGCACCGTTTGTCCGTCTGGTCAAGGAGGTCACCGAATTCTACTGTCCTGCA-------------------------ATCAGCCGCTGG--------------ACTCCCCAAGCGCTCTTGGCAGTTCAAGAGGCTGCAGAGTATCACCTCGTCGACGTATTTGAAAGGGCACATCTCTGTGCCATCCATGCAAAGCGTGTTACCGTCATGCAAAAGGACATACAACTCGCA------------------------
--------
>Hordeum_marinum
------------------------------------------------------------------------------------------------------------------------------------------------------------------------------------------------------------------------------------------ ---------------------------GTGGCGCTGCGGGAGATCAGGAAGTACCAGAAGTCCACCGGGCTGCTCATCCCCTTCGCGCCCTTCGTCCGGCTGGTTAAGGAGATCACCAAC---------------GACTTAACGAAGGGAGAGCTGAAC---------CACTGG--------------
ACACCTCAGGCGCTCGTCTCGTTGCAAGAGGCTGCAGAGTATCACATAGTCGATCTATTCGAAAAGGCAAATCTATGTGCCATCCATGCTAAGCGTGTTACCATGATGCAAAAGGACATACAGCTGGCAAGGCGTATCGGGGGGACAAGGCTTTGG------
>Panicum_virgatum
ATGGCTCGCACCAAGCACCCGGCCGTGAGGAAATCG---------AAGGAGCAGCCCAAGAAGAAGCTCCAGTTCGGGCGCTCCCCGCACGGGAGGGCG---ACGCCG-----------ACAGGTGGA---GCGAGCACATCG------GCGACTCCGGCAAGC------GCTGCAGGGACCGGGGAGAGAGCGGCGGCTGGAGGT------ACGGCGGGGCCGCAG---CAGCAGAAGGTGAAGAAACCA---------
CACCGTTGGAAGCCAGGGACTGTAGCGCTGCGGGAGATCAGGAAGTTCCAGAAATCCACCGAGATGCTTATCCCCTTTGCACCATTTGCCCGTCTGGTGAGGGAGATCACTGAGTTCTACTCAAGGGGG-----------------------AATGTGACACGCTGG--------------
ACCCCGGAAGCCATCCTTGCAATACAAGAGGCAGCAGAATTCCACCTGATAGAACTGTTCGAAGTGGCAAATCTTTGTGCCATCCACGCCAAACGTGTTACCATCATGCAAAGGGACATACAGCTTGCAAGGCGTATCGGTGGA---AGGCGCTGG------
>Brachypodium_distachyon
ATGGCCCGCACGAAGCGCCCGGCCATCAGGAAGTCG---------AAGCCGCAGCCCAAGAAGCAACTCCAGTTCGAGCGC------------------------------------ACAGGCGGC---GCGAGCACCTCGGCGTCGGCGACCCCGGGGAGACGT---GGAGGCCGGACCCCGGCGCGAGCGGCGGGTCAAGCG------GCACCCGCACAACAG---AAGCCG------AAGAAGCCA---------
CACAGATTCCGGGCAGGCACGGTGGCGCTGCGGGAGATCAGGAAGTACCAGAAATCCCCTGAGCTGCTCATCCCATTCGCACCCTTCGTCCGTCTGATTAAGGAGATCAGTAATTTCTACTCACCTGAG--------------------------ATCTCGCGCTGG--------------ACTCCTCAAGCTCTCGTTGCTTTGCAAGAGGCTGCAGAATACCACTTGGTAAACATATTTGAAAAGGCAAATTACTGTGCCATCCATGCGAAGCGTGTTACCATGATGCAAAAGGACATACAGCTTGCGAGG-------------------
----------
>Saccharum_hybrid
ATGGCTCGAACCAAGCACCAGGCCGTGAGAAGGCCA---------ACGCAGAAGCCCAAGAAGAAGCTCCAGTTCGAGCGCGCA------------------------------------GGTGGG---GCGAGTACCTCG------GCGACCCCGGAGAGA------AATGCTGGGACCGGGGGAGGAGCGGCAGCTCGCGTT------ACACGGGGGCGT--------------GTGGAGAAGAAG---------
CTTCGCTGGCGGGCAGGGACTGTAGCGCTGCGGGAGATCAGGAAGTACCAGAAGTCCACTGAGCCGCTCATCCCCTTTGCGCCCTTCGTACGCGTGGTTAAGGAGTTAACTGGATTC-------------------------------------
ATTACAGACTGGAGGATAGGACGCTATACCCCTGAAGCCCTCCTTGCGCTGCAAGAGGCAGCAGAATTCCACTTGATAGAACTGTTTGAAGTGGCGAATCTGTGTGCCATCCATGCCAAGCGTGTCACAGTCATGCAAAAGGACATACAACTTGCAAGGCGTATCGGAGGA---AAGCGTTGGGCG---
>Sorghum_bicolor
ATGGCTCGAACCAAGCACCAGGCCGTGAGGAAGCTG---------CCGCAGAAGCCCAAGAAGAAGCTCCAGTTCGAGCGCGCA------------------------------------GGTGGG---GCGAGTACGTCG------GCGACCCCGGAGAGG---AGGAATGCTGGGACCGGGGGAGGAGCCGCGGCTCGCGTT------GCACGGGGGCGT--------------GTGGAGAAGAAG---------
CATCGCTGGCGGGCAGGGACTGTAGCGCTGCGGGAGATCAGGAAGTACCAGAAGTCCACTGAGCCGCTCATCCCCTTTGCGCCCTTCGTACGTGTGGTCAAAGAGTTAACTGCATTC-------------------------------------
ATAACAGACTGGAGGATAGGGCGCTACACCCCTGAAGCCCTCCTTGCGCTGCAAGAGGCAGCAGAATTCCACTTGATAGAACTGTTTGAAGTGGCGAATCTGTGTGCCATCCATGCCAAGCGCGTAACAGTCATGCAAAAGGACATACAACTTGCAAGGCGTATCGGAGGA---AGGCGTTGGTCG---
>Cenchrus_americanus
ATGGCTCGAACCAAGCACCAGGCCGTGAGGACGGCGGGCCGC---------CCGCCCAAGAAGAAGCTCCAGTTCGAGCGCTCCCCTCGCCAGAGGGCG---GCGCAG-----------ACAGGCGGC---GCGAGCACCTCG------GGAACCTCGGGGAGG------GGTGCGCCGGCTCGGGGTGGAGCGGCTGCCGCTGGG------GCGGCAGGGCGT--------------ATTAAGAGGCCG---------
CATCGGTGGCGACCAGGGACGGTGGCTCTGCGGGAGATCAGAAAATTCCAGAAATCTACCAATCCTCTTATCCCATTTGCTCCCTTTATTCGTCTGGTGAGGGAGATCACTAACGACTATTCGAAGGAA--------------------------GTGACACGCTGG--------------
ACTCCTGAAGCCCTCCTTGCGCTGCAAGAGGCAGCAGAGTTCCACTTAATTGAGCTGTTTGAAGTGGCAAATTTGTTGGCCATCCATGGGAAGCGTGTTACTATCATGCAAAGGGACATACAGCTTGCAAGGCGTATCGGAGGA---AGGCGTTGGTCG---
>Osativa
ATGGCTCGCACGAAGCACCCGGCGGTGAGGAAGTCG---------AAGGCGGAGCCCAAGAAGAAGCTCCAGTTCGAACGCTCCCCTCGG---CCGTCGAAGGCGCAGCGC---------GCTGGTGGCGGCACGGGTACCTCG------GCGACCACGAGGAGC-----------------GCGGCTGGAACATCGGCTTCAGGG------ACGCCTAGGCAGCAAACGAAGCAG------AGGAAGCCA---------
CACCGCTTCCGTCCAGGCACAGTGGCACTGCGGGAGATCAGGAAATTTCAGAAAACCACCGAACTGCTGATCCCGTTTGCACCATTTTCTCGGCTGGTCAGGGAGATCACTGATTTCTATTCAAAGGAT--------------------------GTGTCACGGTGG--------------
ACCCTTGAAGCTCTCCTTGCATTGCAAGAGGCAGCAGAATACCACTTAGTGGACATATTTGAAGTGTCAAATCTCTGCGCCATCCATGCTAAGCGTGTTACCATCATGCAAAAGGACATGCAACTTGCCAGGCGTATCGGTGGGCGGAGGCCATGG------
>Oaustraliensis
ATGGCTCGCACGAAGCACCCGGCGGTGAGGTCGTCG------TCAAGGACGGAGCCCAAGAAGAAGCTCCGGTTCGACCGCTCCCCTCGG---CCTTCGAAGGTGCAGAGC---------ACTGGTGGT---GCGGGTACCTCG------GCGACCACGGGGAGC---------GCGGGGACCGCGGCTGGA-----------GGG------ACGCCTGGGCAGCAGACAAGGCAG------AGGAAGCCA---------
CACCGATTCCGTCCAGGCACAGTAGCACTACGGGAGATAAGGAAATTTCAGAAAACAACTGAACTTCTTATCCCTTTTGCGCCATTTTCCCGTCTGGTCAGGGAGATCACTGACTTCTATTCGAAGGAT--------------------------GTGTCACGCTGG--------------
ACTCTTGAAGCTCTCCTTGCACTGCAAGAGGCAGCAGAATACCATTTGGTGGACTTGTTTGAAGTGTCAAACCTTTGCGCCATCCATGCTAAGCGTGTTACCATCATGCAGAAGGACATACAGCTTGCCAGGCGTATCGGTGGGCGGAGGCCATGG---GGC
>Obrachyantha
ATGGCTCGCACCAAGCACCCGGCGGTGAGGAAGTCG---------AAGCCGGAGCCCAAGAAGAAGCTCCAGTTCGAGCGCTCCCCGCGGAGGCCGTCG---GCGCAGCGC------------GGTGGC---GCGGATACCTCG------GCC---ACGAGGAGC---------GCGCGGACTGCGGCCGGAACGTCGGCCGAAGGG------ACGCCTGGACAGCAGTCGAGGCAG------AGGAAGCCA---------
CACCGATTCCGTCCAGGCACAGTAGCTCTGCGGGAGATCAGGAGATTCCAGAAATCGACCGAACTGCTTATCCCATTTGCGCCATTTTCCCGTCTGGTGAGGGAGATCACTGATTTCTATTCCAAGGAT--------------------------GTGACGCGCTGG--------------
ACTCTTGGAGCTCTCCTTGCACTGCAAGAGGCAGCAGAATACCACTTAGTGGATTTATTTGAAGTAGCAAATCTCTGCGCCATCCATGCTAAGCGTGTTACCATCATGCAAAAGGACATACAACTTGCCAGGCGTATCGGTGGACGGAGGCCATGG------
```

Tetrahymena – CenH3 alignment before masking unreliable residues inferred in Bali-Phy

>leucophrys
AGAAGAAGGAGTAATTCAAATCAAACTTAATAAAGATCATAAAGCGCT---TCCAATAACCCATAACCACAATAAAGATCAAAATCATCAGGA---AATCAAGAAAAT---AAATAAGCGAAAGCA---
AAGCAATAATAAAAGAAACCAACTACAACAAAGAAAAAGAGATAGTCTTCAGGTGATAAGTATGAATCTGCTAGGGATAAAGTGATAAGAAGATTTAGACCTGGAGATAATGCCTTAAAAGAATTAAGATAATATAACTAAACCCCATCATTATTAATCAGAAAATTACCTTTTTAGAGACTAGTGAGAGAAATATCAACAAGAATGTCAGAGCAAGAGTAATTAAGATGGACCAGTTTTGCTTTGGTTCTATTATAGACAGTTGTGGAGGACTATATGGTCTCTTTCTATGAAGATGCTAATGCCTG
T---
>silvana
AAGAGGAGGAGTAATTCAAACTAAAATCAACAAAGATCTACGAGCACA---ACTAATAATTAATCGAATTAATAGAGATCAAAATCTACTGGA---AACCAAGGTAAT---
AAATAGGTTCCTGCAACAGCTTAGAAATAAAAAAAGCCAGATACCACAAAGAAAAAGAGATAATCATCTGGTGAAAAATATGAGTCTACAAGAGATAAAGTTATTAGGAGATTTAGACCTGGTGATAATACATTAAAGGAACTTCGATAATATAATTAAGCTCCAACTCTGATGATTAGGAAACTACCTTTTTAGAAGTTAGTGAGAGAAATAGCATCTAGAATGACAGAATTAGAGCATTTGAGATGGACTAGTTAATCCTTGATGCTCTTACAATCAGTTACTGAGGATTATATGGTATCGTTTTAT
GAGGATGCTAACGCCTGT---
>limacis
AAAAAGAGGAGTAATTCTAATTAAAGCTAATAAAGATAGAGTAGTGCA---AATAATGATTAATCAACTAATCGAAGATCTAAATCTGCAGGA---AACCAAGGGAAT---AAATAAAAGGGTTTA---
CCTCAATAATAAAAAAAGGCTGTTTCTACGAAAAAAAAGAGATAATCTTCTGGTGAGAAGTATGAATCTGCTAGAGATAAAGTAATAAGAAGATTTAGACCTGGAGATAATGCACTAAAAGAACTCAGATAATATAATTAAACTCCATCATTATTAATTAGAAAATTACCTTTTTAGAGATTAGTAAGAGAAATATCAACCAGAATGACAGAAGAGGATTCGCTGAGATGGACTAGTTTTGCATTAGTATTATTGTAAACAGTTGTTGAAGATTATATGGTATCATTTTATGAAGATGCTAATGCATGT-
----
>elliotti
AAAAGAAGAAGCAATTCTAACTAAAATTAAGAGAGGTCTGAGAGTTCT---AAAAAAATTAAATAAGATAATTAAAGATCTAAATCAGCGGGAATACAGTAAGATAAT---AAAAATAAAAAGGAT---AACAAAGATTTGAAAAATAAGGCTAGCACAAAG---
AAAAGATAATCATCAGAGGAAAGATATGAATCAGCAAGAGATAAAGTTATCAGAAGATTTAGACCTGGTGATAATGCTCTTAAATAATTAAGATAATATAATCAAACTCCTTCGCTGCTTATAAGAAAATTGCCTTTTTAAAGGCTAATAAGGGAAATTTCTACAAGAATGACAGAAGAAGATCAGTTAAGATGGACAAGTTTTGCTTTAGTTTTACTTTAAACTGTTGTAGAAGATTATATGGTTTCATTTTTTGAAGATGCAAATGCTTGTGCTCTACATGCTAAAAGAGTAACTCTAATGTCTAAGG
ATCTTGCTCTTGCAGCAAGAATAAGGGGATAAAAAAATGTT
>malaccensis
AAAAGGAGGAGTAATTCAAATTAAGATTAATAGAGATCGGGTAGCTAG---AAAAAAAATAAATAGGATAATTTAAGATCTAAATCTGCTGGGATATAGTAAGAGAATGAAAAAAATAAAAAACAA---
ATCCAAGACCAAAGAAACAAAGCAAGCACAAAAAAGAAAAGAGAATCATCTGGCGAAAAATATGAATCTGCAAGAGATAAAGTTATAAGAAGATTTAGGCCAGGAGATAATGCTCTTAAATAATTAAGATAATATAATTAGACTCCATCATTGCTTATAAGAAAATTACCCTTTTAGAGGTTGATAAGAGAGATTTCTACAAGAATGACAGAAGAGGATTAGCTAAGATGGACTAGTTTTGCTCTTGTTTTACTTTAAACAGTTGTAGAAGATTATATGGTTTCCTTTTTTGAGGATGCTAATGCTTGT
GCTCTCCATGCTAAAAGAGTAACTCTAATGTAAAAAGATCTTGCCCTTGCTGCAAGAATAAGGGGATAAAAAAATGTT
>thermophila
AAAAGGAGGAGTAATTCAAATCAAAATTAATAGAGATCTGATAGCCTG---AAAAAAAATAAATAGGATAATTTGAGATCTAAATCTGCTGGGAATTAATAAGGGAATGAAAAAAACAAAAAAGAT---
ATTCAAGACCAAAGAAATAAAGCAAGTACAAAAAAGAAAAGGGAGTCATCAGGAGAAAAATATGAGTCTGCAAGAGATAAAGTTATTAGAAGATTTAGGCCAGGGGATAATGCCCTTAAGCAATTAAGATAATATAATTAGACTCCATCATTGCTAATAAGAAAATTACCCTTTTAGAGGTTAATAAGAGAGATTTCTACAAGAATGACAGAAGAAGATTCGCTGAGATGGACAAGTTTTGCTCTGGTTTTACTTTAAACAGTTGTAGAAGATTATATGGTTTCATTTTTTGAAGATGCGAATGCTTG
TGCTCTCCATGCTAAAAGAGTTACTCTCATGTCTAAAGATCTTGCTCTTGCGGCAAGAATTAGGGGATAAAAAAATGTT
>furgasoni
AAAAGGAGGAGCAATTCTAACCAAAATTAATCATCATCTAATAATGTT---GCTAATAATTAATCTGCTAATCAGAGATCAAAATCAGCAGGG---CCATAAGGGAAT---AAATAAAAAAATTAG---CCTCAATAATAAAAAAAGGCTGTTTCC------
AAAAAGAGATAATCATCTGGTGAGAAATATGAATCGGCAAGAGATAAAGTAATAAGAAGATTTAGGCCAGGGGATAATGCATTAAAAGAAATGAGATAATATAATCAAACTCCATCTCTACTAATAAGAAAATTGCCTTTTTAGAGGCTAGTAAGGGAAATATCAACAAGATTGACAGAGGAAGATAATTTGAGATGGACAAGCTTTGCCTTAGTGTTATTATAAACTGTTGTTGAAGATTACATGGTATCATTTTATGAAGATGCAAATGCATGTGCTTTACATGCTAAAAGAGTTACCTTAATGAC
AAAAGATTTAGCACTAGCTGCAAGAATTAGAGGATAAAAAAATGTT
>mobilis
AAAAGGAGAAGCAATTCTAACCAAAATTAATCAGCATCAAATAATGTT---TCAAATAATTAATCTGCAAATCAGAGATCAAAGTCAGCAGGA---CCATAAGGGAAT---AAATAAAAAAATTAG---CCTCAATAATAAAAAAAGGCTGTTTCC------
AAAAAGAGATAATCATCAGGTGAAAAATATGAATCAGCAAGAGATAAAGTAATCAGAAGATTTAGGCCAGGGGATAATGCTTTAAAAGAAATGAGATAATATAATCAAACTCCATCTCTACTAATAAGAAAATTACCTTTTTAGAGGCTAGTAAGGGAAATATCAACAAGATTGACAGAGGAAGATAACTTGAGATGGACAAGCTTTGCTTTAGTGTTATTATAAACTGTTGTTGAAGATTACATGGTATCATTTTATGAAGATGCAAATGCATGCGCTTTACATGCAAAAAGAGTTACCTTAATGAC
AAAAGATTTAGCACTAGCTGCAAGAATTAGAGGATAAAAAAATGTT
>tropicalis
AAAAGGAGAAGCAATTCTAACCAAAATTAATCAGCATCGAATAATGTT---TAAAATAATTAATCTGCAAATCAGAGATCAAAGTCAGCAGGA---CCATAAGGGAAT---AAATAAAAAAATTAG---CCTCAATAATAAAAAAAGGCTGTCACC------
AAAAAGAGATAGTCATCAGGTGAAAAATATGAATCAGCAAGAGATAAAGTAATTAGAAGATTTAGGCCAGGGGATAATGCTTTAAAAGAAATGAGATAATATAATCAAACTCCATCTCTACTAATAAGAAAATTACCTTTTTAGAGGCTAGTAAGGGAAATATCAACAAGATTGACAGAGGAAGATAACTTGAGATGGACAAGCTTTGCCTTAGTATTATTATAAACTGTTGTTGAAGATTACATGGTATCGTTTTATGAAGATGCAAATGCATGCGCTTTACATGCAAAAAGAGTTACCTTAATGAC
AAAAGATTTAGCACTAGCTGCAAGAATTAGAGGATAAAAAAATGTT
>mimbres
AGCAAAAGAAGTAATTCAAACCAGCCATAGTAAAAATCTAATAAATAAATAAGCAATATTTAAGAAAATAAAAGACGATCAAAATCTGCAGGT---GGGCCAACTAAT---AAACCAAAAAATCCA---
CCTTAGTAATAAAAAAAGGTAGCCACATCAAATAAAAAGAGATAATCATCTGGTGAAAAGTATGAATCTGCTAGAGATAAGGTAATAAGAAGATTTAGACCAGGTGATAATGCTCTCAAAGAGCTCAGATAATATAATTAGAATCCTTAGTTATTAATAAGAAAATTACCTTTTTAGAGGTTAGTAAGAGAAATAACGTAAAGGATGACAGTAGAAGAATAGTTGAGATGGACAAGTTTTGCCCTAGTTATATTATAAACAATATCTGAAGATTATATGGTCTCATTTTTTGAAGATGCAAATGCTTG
TTGCTATCATGCTAAAAGAGTTACTCTTATGACAAAAGACATGGCTCTTGCTGCTAGAATTAGAGGATAAAAAAATGTT
>rostrata
AAGAGGAGAAGTGTTTCTAATTAATCTCAATAAAGATCAAATAATGCAACAAATAATAATTAAGTAAATAATTAAAGATCGAAATCTGCAGGT---GGATAAATGAAT---AAATAAAAAAAACCCA---
TCTTTACAGTAAAATAAGCCTGTATCAACTAAGAAAAAGAGATAATCCTCTGGTGAAAAATATGAATCAGCAAGAGATAAAGTAATTAGAAGATTTAGACCAGGAGATAATGCTTTGAAAGAACTTAAATAATATAATTAAAATCCATCACTATTTATTCGAAAACTACCTTTTTAAAGGTTAGTGAGAGAAATATCAACACGATTGTCAGATTAGGATTAAATTAGATGGACAAGTATTGCTCTAATTCTATTATAAACCGTTACAGAAGATTATATGATATCATTTTATGAAGATGCGAATGCATGCG
CTTTACATGCTAAAAGAGTTACCCTTATGAGCAAAGATATGATTCTTGCTGCTAGAATTAGAGGATAAAGGAATGTT
>borealis
AAGAGGAGAAGCGTTTCTAATTAATCTCAATAAAGATCAAATAATGCTACAAATAATAATTAAGTA---AATTAAAGATCGAAATCTGCAGGT---GGATAAATGAAT---AAATAAAAAAAACCCA---TCTTAACAGTAAAATAAGCCTGTAACA------
AAAAAGAGATAATCCTCTGGTGAAAAATATGAATCTGCAAGAGATAAAGTAATTAGAGGATTCAGACCAGGAGATAATGCTTTGAAAGAACTTAAATAATATAATTAAAATCCATCACTATTTATTCGAAAACTACCTTTTTAGAGGTTAGTGAGAGAAATATCAACACGATTGTCAGATCAGGATTAAATTAGATGGACAAGTATTGCTCTAATCCTATTATAAACAGTTACAGAAGATTATATGATATCTTTTTATGAAGATGCGAATGCATGTGCTTTACACGCTAAAAGAGTTACCCTTATGAGCA
AAGATATGATTCTAGCTGCTAGAATTAGAGGATAAAGGAATGTT
>canadensis
AAGAGGAGAAGTGTTTCTAATTAATCTCAACAAAGATCAAATAATGCAACAAATAATAATTAAGTA---AATTAAAGATCGAAATCTGCAGGT---GGATAAATGAAT---AAATAAAAAAAACCCA---TCTTTACAGTAAAATAAGCCTGTAACT------
AAAAAGAGATAATCCTCTGGTGAAAAATATGAATCTGCAAGAGATAAAGTAATTAGAAGATTTAGACCAGGAGATAATGCTTTGAAAGAACTTAAATAATATAATTAAAATCCATCACTATTTATTCGAAAACTACCTTTTTAGAGGTTAGTGAGAGAAATATCAACACGATTGTCAGATTAGGATTAAATTAGATGGACAAGTATTGCTCTAATTCTATTATAAACTGTTACAGAAGATTATATGATATCATTTTATGAAGATGCGAATGCATGCGCTTTACATGCTAAAAGAGTTACCCTTATGAGCA
AAGATATGATTCTTGCTGCTAGAATTAGAGGATAAAGGAATGTT

Plasmodium – CenH3 alignment before masking unreliable residues inferred in Bali-Phy

```
>Plasmodium_falciparum
ATGGTGAGAACAAAAAAGAATATACCAAATCATAACCCCTTAAATGCATTTAATAGGGACAAGTCATTTAAA--------------------------------------------------------------------------------ACAAACAAAACATTACCAAATAGAACAGTACACCATGGAATTAGTTCCAAGACTACTAATATAAACAGACCCAGTGTAAATAGAGGAGGTATA--------------------------------------------------------------------------AATGAAGTTGCACAAAAAAACTTGCACAGG---
ACAAATATAAGGAAACCCCATAGATACAGGCCAGGTGTATTAGCATTAAAAGAAATAAGAGCATATCAAGCATCGACTCAATTATTAATACCTAAAATTCCATTTGTTCGAGTAGTAAAAGAAATTACAAGATTATTTGAATTACCAGATGAGCAATTTCGTTATACACCTGAAGCATTATTAGCTTTACAAACAGCATCAGAAGCATATTTGGTTAGTTTATTTGAGGATGCTTATTTATGTTCATTACATGCAAACAGAGTAACACTTATGCCCAAAGATATTCATTTAGCTAGAAGAATACGCGGAA
GAGAC
>Plasmodium_reichenowi
ATGGTGAGAACAAAAAAGAATATACCAAATCATAACCCCTTAAATGCATTTAATAAGGACAAGTCATTTAAA--------------------------------------------------------------------------------ACAAACAAAACATTACCAAATAGAGCAGTACACCATGGAATTAGTTCCAAGACTACTAATATAAACAGACCAAGTGTAAATAGAGGAGGTATA--------------------------------------------------------------------------AATCAAGTTGCACAAAAAAACTTGCACAGG---
ACAAATATAAGGAAACCCCATAGATACAGACCAGGTGTATTAGCATTAAAAGAAATAAGAGCATATCAAGCATCGACTCAATTATTAATACCTAAAATTCCATTTGTTCGAGTAGTAAAAGAAATTACAAGATTATTTGAATTACCAGATGAGCAATTTCGTTATACACCGGAAGCATTATTAGCTTTACAAACAGCATCAGAAGCTTATTTGGTTAGTTTATTTGAGGATGCTTATTTATGTTCATTACATGCAAACAGAGTAACACTTATGCCCAAAGATATTCATTTAGCTAGAAGAATACGCGGAA
GAGAC
>Plasmodium_yoelii
ATGGTGAGAACAAAAAAAAGCGTAACTACACATACCCCTATAAATACA------------------------CATACGCATATGTTTAATATGCTTGCAAATAATCCAATGATA---------------------------------------------------AATAAATCAGCAAATAACCAAATTGGGTCAAAATCAATA-----------------------------------AATAAAAATAATTCAGGAAAT-----------------------------------------------------ATAACACACAAAAATATAAATAAA---
AATCGTATCCGAAGACCACATAGATATCGACCTGGTGTATTAGCATTAAAAGAAATTAGAGCTTATCAAGCAACAACTCAATTATTAATACCCAAAATACCATTTGTAAGAGTAATTAAAGAAATAACAAAATTATATGAATTGCCAAATAATCAATTTCGTTATACTCCCGAAGCTTTATTAGCTCTTCAAACTGCATCAGAAGCATATTTAGTTAGCTTATTCGAAGATGCATATTTATGTTCTCTTCATGCAAATAGAGTAACACTTATGCCAAAAGATATACATTTAGCTAGACGGATAAGAGGCCGT
GAT
>Plasmodium_vinckei
ATGGCGAGGACAAAAAAGAGTGTAACTAACCATACCCCTATAAACACA------------------------CATACGCATATGTTTAATATGCTCGCAAATAATCCAATGGTA---------------------------------------------------AATAAATCAACACATAACCAAATTGGGTCTAAAGGAACAAATATAAAT---------------------------AATAAAAGTAATTCAGGGAAT-----------------------------------------------------ATGACACAAAAAGGTTTAAATAAA---
AGTCGTATTAGAAGACCACATAGATATAGACCTGGTGTATTAGCATTAAAAGAAATTCGAGCTTATCAAGCAACAACTCAATTATTAATACCTAAAATACCATTTGTAAGAGTAGTTAAAGAAATAACAAAATTATATGAATTACCAGATAGCCAATTTCGTTATACTCCTGAAGCTTTATTAGCTCTTCAAACGGCTTCGGAAGCATATTTAGTTAGCTTATTTGAAGATGCATATTTATGTTCACTACATGCAAACAGAGTGACTCTCATGCCTAAGGACATACATTTAGCTAGACGAATAAGAGGGAG
AGAT
>Plasmodium_berghei
ATGACAAGGACAAAAAAAAGTGTAACTACACATACCCCTATAAACACA------------------------CATACGCATATGTTTAATATGCTTGCAAATAATCCAATAATA---------------------------------------------------AATAAACAGGCACACAACCAACTTGGATCTAAAGTAACAAATATGAAC---------------------------AATAAAAATAATTCAGGAAAT-----------------------------------------------------ATAACACAAAAAAGTATAAATAAA---
ACTCGTATTCGAAGACCACATAGATATAGACCCGGTGTGTTAGCATTAAAAGAAATTCGAGCTTATCAATCAACAACTCAATTATTAATACCCAAAATACCATTTGTGAGGGTAGTTAAAGAAATAACAAAATTATATGAACTACCAAATAGCCAATTTCGTTATACTCCAGAAGCTTTACTAGCTCTTCAAACTGCATCAGAAGCATATTTAGTTAGTTTATTTGAAGATGCATATTTATGTTCACTTCATGCAAACAGAGTAACACTTATGCCAAAGGATATACATTTGGCTAGAAGAATAAGGGGGCG
TGAT
>Plasmodium_chabaudi
ATGGCGAGGACAAAAAAGAGTGTAACTAACCATACCCCTATCAACACA------------------------CATACGCATATGTTTAATATGCTCGCAAATAATCCAATGGTA---------------------------------------------------AATAAACCAGCACATAACCAAATTGGATCCAAACCAACAAATGTAAAT---------------------------AATAAAAGTAATTCAGGAAAT-----------------------------------------------------ATGACACAAAAAGGTTTAAATAAA---
AATCGTATTCGAAGACCACATAGATATAGACCTGGTGTATTAGCATTAAAAGAAATTCGAGCTTATCAAGCAACAACTCAATTATTAATACCTAAAATACCATTTGTAAGAGTAGTTAAAGAAATAACAAAATTATATGAATTGCCAGATAGCCAATTTCGTTATACTCCAGAAGCTTTACTAGCTCTTCAAACTGCTTCGGAAGCATATTTAGTTAGCTTATTTGAAGATGCCTATTTATGTTCGCTACATGCAAACAGAGTGACCCTCATGCCTAAGGACATACATTTAGCTAGACGAATAAGAGGCCG
AGAT
>Plasmodium_fragile
ATGGTGCGAACGAAAAAGAGCGTGCCCATGCACAACCCCTTGAGC----------------------------------------------------------------------AACCCAGACGGGGTGGGCAGCAGCAACGAACCGACGAACAAGACAGTGCCTAGCAGACCATCACACAAGCAAGTC------------------------------------------------------------------------
GCCTCGTCCAAATCAAATAACAACGCAGGAACAGGCAAAGGGACTCGAAGTCAAATTCCGCAAAAGGGTTTGAAGAAGACAACCCGAATTAGGAGGCCCCATAGGTACAGACCAGGTGTATTAGCATTAAAGGAAATAAGAGCATACCAAGCGACTACACAGTTACTCATTCCTAAAATACCTTTTGTTCGAGTCGTAAAGGAGATTACTCGGTTGTTTGAATTGCCCAATGAACAACTGCGTTACACCCCCGAGGCCCTGTTGGCACTGCAAACCGCATCGGAGGCCTACCTGGTCAGTCTCTTCG
AAGATGCCTACTTGTGTTCACTTCACGCAAACCGCGTCACCCTCATGCCTAAGGACATACACTTGGCTCGCCGTATCCGCGGCCGCGAC
```

Aspergillus – CenH3 alignment inferred in Bali-Phy. All the codons with reliability score below 80% are masked with NNN.

```
>Aspergillus_acidus
ATGCCCCCCAAGACANNNGGTCGCGGTNNNNNNCGCAAGNNNNNNACGGCGGCG----------------------CCGCGA-------------------------------------CAATCAACAGCT-------------------------------------------------GGCGACCGTGCT------------------------------NNNGCAAGCTCC------TCCACA------------------GCA---GCTACAACG-----------------------GCCTCGCCG---------------------------------------------------------GCG--------------------------------------------------
GCCAAGAAAGTG---CGCAAATCCACT---GGA---GCG-----------ACTAGG-----------------GGAGGGAAAAGGCCTGCTGGAAAGGCACCC--------------------------------------AGAGAG---TCCGATGTT-----------------
CAGCCTGGTGACCCTACACCACAAGGACGTGTACGCCGCTACAAGCCCGGAACCGTGGCTCTCAAGGAGATCCGCAAGTACCAACGCTCGTACGATCTGCTTATCCAAAAGCTTCCTTTCGCTCGGCTGGTCCGCGAGGTCGCGCTTGACCTCCTCCCTTCAGAAGTTGGCGCCGAGCTGCGGTGGCAGTCGCATGCGATCCAAGCGCTCCAGGAAGCAGCCGAAGCTTTCCTTGTACATCTCTTCGAGGACACCAACCTCTGCGCGATTCACGCCAAGCGCGTGACCATCATGCAGAAAGATATCCA
ACTTGCTCGACGCATCCGTGGTGTCTGGGGTGGTTTGGGC
>Aspergillus_aculeatus
ATGCCGCCTAAGACAGGG---------CGCGGTCGCAAGTCAATT---GCTGGG-----------------------CCACGG------------------------------------------AAANNNNNNNNNN------GCCGCCGCCGCCGCCGCCGCCACTACTGCTGCTGCTACT--------------NNNGATGGTNNN--------------GCC---------GCTGCTGCCGGCCCG------TCAACT------------------GCC---CCAGCTACGNNNNNNNNNNNN-----------------CCC---------------------------------------------------------GCC-------------------
-------------------------------GCTCGC---NNN---CGTAAACCTGCG---GGT---GTC-----------ACCAGA---------------------NNNAAGCGACCGGCTAAA---GCGCCC---------------------------------------AGGACA---TCCAATGTT-----------------
GAACCGGGCGATCCCACCCCCACAGGTCGTCGTCGCCGGTACAAACCTGGAACGGTTGCCCTGAAGGAGATCCGCCATTACCAACGCTCATTCGACCTGCTCATCCGAAAGCTGCCATTCGCGCGACTGGTCCGCGAGGTTGCCTTGGATCTTCTCCCCGCTGAAGTCGGCTCCGAGCTGCGCTGGCAATCGCATGCGATCTTGGCCCTCCAGGAAGCCGCCGAGGCGTTCCTGGTCCATCTTTTCGAGGACACCAATCTCTGTGCCATTCATGCCAAGCGCGTGACCATCATGCAAAAGGATATCCA
GCTTGCTCGACGCATCCGGGGTGTCTGGGGTGGTCTGGGT
>Aspergillus_brasiliensis
ATGCCCCCCAAGACANNNNNNN------NNNNNNCGCAAGNNNNNNGTGGCGGCG----------------------CCGCGA-------------------------------------CAATCAACAGCT-------------------------------------------------GGCGACCGTGCT------------------------------NNNGCAAGCTCC------TCGAAA------------------GCA---GCTGCAACG-----------------------ACATCGCCG---------------------------------------------------------GCG--------------------------------------------------
GCGAAGAAAGTG---CGCAAATCCACT---GGA---CCG-----------GCTAGG-----------------GGAGGGAAAAGACCTGCTGGAAAGGCGCCC--------------------------------------AGACAG---TCCGATGTT-----------------
CAGCCTGGTGACCCCACGCCACAAGGACGTGTGCGCCGCTACAAGCCCGGAACTGTGGCTCTTAAAGAGATCCGCAAGTACCAGCGCTCGTACGATCTGCTTATTCAAAAGCTTCCTTTTGCTCGGCTGGTCCGCGAGGTCGCGCTTGACCTCCTCCCTTCAGAAGTTGGCGCTGAGCTGCGGTGGCAGTCACATGCGATCCAAGCGCTTCAGGAAGCAGCCGAAGCTTTCCTCGTACATCTCTTTGAGGATACCAATCTCTGTGCTATCCACGCCAAGCGCGTGACCATCATGCAGAAAGATATCCA
ACTTGCTCGACGCATCCGTGGTGTCTGGGGTGGTTTGGGC
>Aspergillus_clavatus
ATGCCACCAAAGACAGGA---------CGGGGCCGGAAGGCAGTC---NNNNNNGGAAGA-----------------NNNNNN-----------AAA----------------------------------------------------------------------------------------NNN---------GAA------------------------------NNNGGCCCC------TCAGAC------------------GCA---CCTGCGACT-----------------------GCATCACCT---------------------------------------------------------TCG-------------------------------------------------
ACCCGAGGCCAG---CGCAAGTCAAGC---GGA---GCG-----------ACAAGG-----------------GGGGGCAAGCGACCGGCCGGC---GCACCG--------------------------------------AGAAAA---TCAGACGTT-----------------
CAACCTGGAGACCCAACCCCTCAAGGTCGCCCACGCCGATACCGACCGGGAACGGTTGCCCTGAAAGAGATCCGCAAATATCAGCGCTCGTACGATCTGCTCATCCAAAAGTTACCCTTTGCGCGACTCGTCCGCGAGGTCGCCTTGGACCTCCTCCCCACCGAGGTCGGCGCCCAATTGCGATGGCAGTCGCATGCAATCCAAGCGCTCCAGGAAGCTGCTGAAGCTTTCCTGGTCCATCTGTTCGAGGACACCAATCTCTGCGCCATCCATGCTAAGCGCGTAACAATCATGCAGAAAGATATTCA
GCTTGCTCGAAGAATTCGCGGTGCTTGGGGTGGCCTGGGT
>Aspergillus_flavus
ATGCCACCGAAGACAGGA---------GGAGGTCGCAAGGTAATC---GCCGCA-----------------------TCGCGA---------------------------------------------------GCAAAG---------------------------------------------------NNNGACGGTNNN--------------NNNAAAGAAAGCNNN------------------------------------------------NNNNNNAAA-----------------------GGGTCCCCT---------------------------------------------------------TCG--------------------------------------------------ACTCGAGGA--
----CGCAAGTCCGCT---GGA---GGT-----------GCCAGA-----------------GCGGGGAAGCGACCGGCTGGT---GCATCG--------------------------------------AGGAAA---TCAGATGTC-----------------
CAACCTGGCGATCCTACCCCTCAGGGCCGCCATCGCCGTTACAGACCGGGCACTGTTGCCCTCAAAGAAATCCGCAAATACCAACGTTCTTATGACCTCCTTATACGAAAGCTCCCATTCGCAAGACTTGTGCGCGAAGTTGCATTGGATCTACTCCCCGCAGATGTAGGATCCGAATTGCGCTGGCAGTCGCAAGCCATCATGGCGCTGCAGGAGGCCGCGGAAGCCTTTCTTGTTCATCTATTCGAGGACACGAACCTGTGTGCTCTACACGCGAAGCGAGTGACTATCATGCAGAAAGATATCCA
GCTTGCTCGGAGAATCCGTGGCGCTTGGGGTGGATTAGGC
>Aspergillus_fumigatus
ATGCCGCCGAAGACTGGA---------CGAGGACGTAAAGCAATT---GGGGCT-----------------------CCTCGAACCCAACCTCGA----------------------------------------------------------------------------------------------NNNNNNNNNNNN--------------------------------NNNGGTCCA------TCTAAC------------------GCT---GCGGCTACA-----------------------GCATCACCT---------------------------------------------------------TCT-------------------------------------------------
ACTCGGGGANNNCCGCCCAAATCTCGC---GGG---GGA-----------ACAAGG-----------------GGGGGAAAGCGACCAGCCCAC---GCCCCG--------------------------------------AGGCCG---TCAGACGTC-----------------
CAACCTGGGGACCCAACCCCTCAAGGTCGCCGACACCGGTACAAGCCGGGAACCGTCGCATTGAAAGAGATCCGCAGATACCAGCGCTCATATGACCTCCTTATTCAGAAGCTACCCTTTGCGCGACTCGTACGCGAGGTCGCATTGGAACTTCTTCCCGCCGAAGTAGGCGCGGAGCTACGGTGGCAGTCGCACGCTATTCAGGCGCTACAGGAGGCCGCCGAAGCTTTCCTCGTCCACCTTTTCGAGGATACCAATCTCTGTGCTTTGCATGCTAAGCGTGTAACGATTATGCAGAAAGATATCCA
GCTCGCCCGAAGAATACGCGGTATTTGGGGCGGTCTGGGT
>Aspergillus_glaucus
ATGCCACCAAAGACAGGA---------AAAGGCCGTAAAGTCGTC---TCAGAA-----------------------CCGCGA-----------------------------------------------------------------------------------------TCGCGCCCCAGCACCNNNNNNNNNNNN--------------------------------GCAGGCCCA------TCA---TCAAAACCGCAACAA--------------ACC-----------------------GCGTCGCCA---------------------------------------------------------NNNAACAAAAGGAAACCCAAGGCTTCC-----------------
----------NNN-----------------------TCCNNNGAANNNAGA-------------------------------------NNNAAGCGGCCATCGGGA---NNNNNNAAAACANNNNNNN-----------GCAGCAAAGACAGCANNNNNNN---TCAGACGTC-----------------
CAACCCGGCGACCCAACCCCCCAAGGCCGTTCACGCCGCTACAAACCCGGAACCGTCGCTCTAAAAGAAATCCGCAAATACCAACGCTCCTTCGACCTCCTCATCCAAAAGCTCCCCTTCGCGCGCCTCGTCCGTGAAGTCGCCCTGGAACTGCTCCCCGCAGACGTAGGCGAACAGCTCCGGTGGCAGTCGCATGCGATCCAAGCACTACAAGAAGCCGCAGAGGCATTCCTCGTCCATTTATTCGAAGATACTAATTTGTGTGCTCTGCATGCTAAGCGCGTGACTATTATGCAAAAGGATATCCAG
CTTGCACGGAGGATTCGTGGTGCTTGGGGTGGATTGGGA
>Aspergillus_kawachii
ATGCCCCCCAAGACANNNGGTCGCGGTNNNNNNCGCAAGNNNNNNACGGCGGCG----------------------CCGCGA-------------------------------------CAATCAACAGCT-------------------------------------------------GGCGACCGTGCT------------------------------NNNGCAAGCTCC------TCAACA------------------GCA---GCTATAACG-----------------------GCCTCGCCG---------------------------------------------------------GCG--------------------------------------------------
GCCAAGAAAGTG---CGCAAATCCACT---GGA---GCG-----------ACTAGG-----------------GGAGGGAAAAGGCCTGCTGGAAAGGCACCC--------------------------------------AGGGAG---TCCGATGTT-----------------
CAGCCTGGTGACCCTACACCACAAGGACGTGTACGCCGCTACAAGCCCGGAACCGTGGCTCTCAAGGAGATCCGCAAGTACCAACGCTCGTACGATCTGCTTATCCAAAAGCTTCCTTTCGCTCGGCTGGTCCGCGAGGTCGCGCTTGACCTCCTCCCTTCAGAAGTTGGCGCCGAGCTGCGGTGGCAGTCGCATGCGATCCAAGCGCTCCAGGAAGCAGCCGAAGCTTTCCTTGTACATCTCTTCGAGGACACCAACCTCTGCGCGATTCACGCCAAGCGCGTGACCATCATGCAGAAAGATATCCA
ACTTGCTCGACGCATCCGTGGTGTCTGGGGTGGTTTGGGC
>Aspergillus_nidulans
ATGCCCCCAAAA------------------NNNNNNNNN--------------------CCATCCTCG------------------------------------------------------------------------------------------------------------------------NNN---------NNNNNNGCAGGTNNNGGAAGCNNNNNN---------------GCGNNN---NNNNNNNNNACAACCTCCACCACAACCTCAGGCNNNNNNNNNNAAGGTAACTAAGAGCTCCAGAACATTAGGGTCAAAGGCATTGGCATCGGCATCGGTGNNN-----------
------------------------------------------------------AAATCT----------------------------AAG-----------------GGAAGTAAGCGACCA-----------CCG--------------------------------------ACGNNNCCATCANNNNNNN------
CAATCCAGCATCNNNCCCGGCGACCCAACCCCCAAAGGCCGTCGTCACCGGTACAAACCTGGTACCGTTGCCCTCAAAGAAATCCGCAAGTACCAACGCTCCTACGATCTGCTTCTCCGAAAATTGCCATTTGCACGTCTCGTCCGCGAAGTTGCTCTCGACATCCTCCCCGCAGACGTTGGCTCCGAGCTGCGATGGCAGTCGCATGCAATCCAGGCGCTCCAGGAAGCCGCTGAAGCCTTTTTGGTACACTTGTTCGAGGACACGAATCTTTGCGCCATCCACGCGAAACGAGTTACGATAATGCA
GAAGGATATCCAGCTTGCGCGTAGGATTCGCGGAGCTTGGGGTGGTCTTGGC
>Aspergillus_niger
ATGCCTCCCAAGACANNNNNNN------NNNGGTCGCAAGNNNNNNGTGGCGGCG----------------------CCGCGA-------------------------------------CAATCAACAGCT-------------------------------------------------GGCGACCGTGCT------------------------------NNNGCAAGCTCG------TCGAAA------------------GCA---GCTGCAACG-----------------------ACGTCGCCG---------------------------------------------------------GCA--------------------------------------------------
GCTAAAAAAGTG---CGCAAATCCACT---GGA---CCG-----------GCTAGG-----------------GGAGGGAAAAGGCCTGCTGGAAAGGCACCC--------------------------------------AGAGAG---TCCGACGTT-----------------
CAGCCTGGTGATCCCACACCACAAGGACGTGTACGCCGCTACAAGCCTGGAACCGTGGCTCTCAAAGAGATCCGCAAGTACCAGCGCTCTTATGATCTGCTTATCCAAAAGCTTCCTTTTGCTCGGCTGGTCCGCGAGGTCGCTCTTGACCTCCTCCCTTCAGAAGTTGGCGCCGAGCTGCGGTGGCAGTCGCATGCGATCCAAGCGCTCCAAGAAGCAGCCGAAGCTTTCCTTGTACATCTCTTCGAGGACACCAACCTCTGTGCTATTCACGCCAAGCGCGTGACCATCATGCAGAAAGATATCCAA
CTTGCTCGACGCATTCGTGGTGTCTGGGGTGGTTTGGGC
>Aspergillus_sojae
ATGCCACCGAAGACAGGA---------AGAGGTCGCAAGGTAATC---GCCGCA-----------------------TCGCGA---------------------------------------------------GCAAAG---------------------------------------------------NNNGACGGTNNN--------------NNNAAAGAAAGCGCAGCTGCAGGCCCA------TCCGGT------------------GCA---GCTGCAAAA-----------------------GACTCCCCT---------------------------------------------------------TCG--------------------------------------------------
ACTCGAGGA------CGCAAGTCCACT---GGA---GGT-----------GCCAGA-----------------GCGGGGAAGCGACCGGCTGGT---GCATCG--------------------------------------AGGAAA---TCAGATGTC-----------------
CAACGTGGCGATCCTACCCCTAAGGGCCGCCATCGCCGTTACAGACCGGGCACTGTTGCCCTCAAAGAAATCCGCAAATACCAACGTTCTTATGACCTCCTTTTACGGAAGCTCCCATTCGCAAGACTTGTGCGCGAAGTTGCATTGGATCTACTCCCCGCAGATGTAGGATCCGAATTGCGCTGGCAATCGCAAGCCATCATGGCGCTGCAAGAGGCCGCGGAGGCCTTCCTTGTTCATCTATTCGAGGACACGAACCTGTGTGCTCTACACGCGAAGCGAGTGACTATCATGCAGAAGGATATCCA
GCTTGCTCGGAGAATCCGTGGCGCTTGGGGTGGACTGGGC
>Aspergillus_sydowii
ATGCCTCCGAAA------------------NNNNNNNNN--------------------CCAGCCTCG--------------------------------------------------------------------------------------------------------------------------NNNACCGCCGGGCCC------TCG------------------------------------NNNNNNNNNNNNACAACAACAGGCNNNNNNCCC---------------------------------------------------------GCT-----------------------------------------------------------------
AAATCCGCC---GGA---NNN---AAAAAAGGTNNNNNNGGAGTCCAGGGAACCACGGGGGGGAAGCGACCA-----------CCA--------------------------------------AGGACA---TCANNNNNN------
CAATCAGATGTTNNNCCCGGAGACCCAACCCCCAAAGGCCGCCGTCACCGGTACAAGCCCGGCACCGTCGCCCTGAAGGAAATCCGCAAGTACCAGCGCTCATACGATCTCCTCCTCCGAAAACTTCCCTTTGCACGATTAGTGCGCGAGGTCGCATTGGACCTTCTCCCCGCAGAAGTCGGCGCGGAGCTGCGGTGGCAGTCGCATGCAATCCTTGCGCTCCAAGAGGCTGCCGAGGCCTTCCTAGTACACTTGTTCGAGGACACCAATCTATGCGCCATCCACGCGAAACGGGTTACGATCATGC
AGAAGGATATCCAGCTTGCGCGCAGAATTCGTGGGGCGTGGGGTGGTCTAGGC
>Aspergillus_terreus
ATGCCACCCAAAACAGGG---------CGAGGCCACAAGATCACC---GCCGCG-----------------------CCCCGG--------------ACAGAGGCAAGCAGCAGTCGTACCACC-----------------------------------------------------------------------------------GCC---------GCCGCCGCCGGCCCG------TCG--------------------------------NNN-----------------------GCGTCGCCG---------------------------------------------------------TCC-------------------------------------------------
GCCCACAGGGTG---CGCAAATCCACC---GGA---CCA-----------TCCAGA-----------------GGCGGGAAGCGACCGGCCGGC---GCGCCG--------------------------------------AGAGAC---TCCGACGTT-----------------
CAACCCGGCGATCCCACCCCGACCGGTCGTCGTCGCCGCTACCGACCTGGCACCGTCGCCCTCAAGGAAATCCGCAAGTACCAACGGTCATATGACCTCCTCATTCAGAAGCTCCCCTTCGCGCGACTCGTCCGTGAAGTCGCACTCGATATCCTCCCCGCCGAGCGCGGCGCCGAGCTCCGTTGGCAGTCGCATGCCATCCAGGCCCTCCAGGAGGCCGCCGAGGCGTTCCTGGTGCACCTGTTCGAGGACACGAACTTGTGCGCCCTGCACGCGAAACGAGTCACCATCATGCAGAAGGATATCC
AGCTTGCGCGACGAATTCGCGGTGTGTGGGGTGGTTTGGGC
>Aspergillus_tubingensis
ATGCCCCCCAAGACANNNGGTCGCGGTNNNNNNCGCAAGNNNNNNACGGCGGCG----------------------CCGCGA-------------------------------------CAATCAACAGCT-------------------------------------------------GGCGACCGTGCT------------------------------NNNGCAAGCTCC------TCAACA------------------GCA---GCTACAACG-----------------------GCATCGCCG---------------------------------------------------------GCG--------------------------------------------------
GCCAAGAAAGTG---CGCAAATCCACT---GGA---GCG-----------ACTAGG-----------------GGTGGGAAAAGGCCTGCTGGAAAGGCACCC--------------------------------------AGAGAG---TCCGATGTT-----------------
CAGCCTGGTGACCCCACACCACAAGGACGTGTACGCCGCTACAAGCCCGGAACCGTGGCTCTCAAGGAGATCCGCAAGTACCAGCGCTCGTACGATTTGCTTATCCAAAAGCTTCCTTTCGCTCGGCTGGTCCGCGAGGTCGCGCTTGACCTCCTCCCTTCAGAAGTTGGCGCCGAGCTGCGGTGGCAGTCGCATGCGATCCAAGCGCTCCAGGAAGCAGCCGAAGCTTTCCTTGTACATCTCTTCGAGGACACCAACCTCTGTGCGATTCACGCCAAGCGCGTGACCATCATGCAGAAAGATATCCA
ACTTGCTCGACGCATCCGTGGTGTCTGGGGTGGTTTGGGC
>Aspergillus_versicolor
ATGCCTCCGAAA------------------NNNNNNNNN--------------------CCGGCCTCG--------------------------------------------------------------------------------------------------------------------------NNNACAGCCGGACCC------TCGAAA------------------ACG---CCCGCA---------------------------NNNNNNCCC---------------------------------------------------------TCC-----------------------------------------------------------------AAATCCGCC---GGA---
NNN---AAAAAAGGTNNNNNNN-----------------GGGGGGAAGCGANNN---------------------------------------------------AGGACA---TCANNNNNNN------
CAACCAGATGTTNNNCCCGGAGACCCAACCCCCAAAGGCCGCCGCCACCGGTACAAGCCCGGCACCGTCGCCCTCAAGGAAATCCGCAAGTACCAACGCTCATATGATCTTCTCCTCCGAAAACTCCCCTTCGCACGATTAGTGCGCGAGGTCGCATTGGACCTCCTCCCCGCAGAAGTCGGCGCGGAGCTACGGTGGCAGTCGCATGCAATCCTTGCGCTCCAAGAAGCCGCCGAGGCCTTCCTGGTACATTTGTTCGAAGACACGAATTTATGCGCCATCCACGCGAAACGGGTTACGATCATGC
AGAAGGATATCCAACTTGCGCGTAGAATTCGCGGGGCGTGGGGTGGTCTAGGC
>Aspergillus_wentii
ATGCCACCAAAGACCGGT---------CGAGGCAATAAGATTATC---GCTGCT-----------------------CCCAGA---------------------------------------------------GCAAAG---------------------------------------------------NNN--------------NNNAAGGAAGAGGCGGAGGCAGGTCCA------TCAAGA------------------GCAAGCCCTGCGACC-----------------------GCGTCGCCA---------------------------------------------------------GCA-------------------------------------------------
ACCCGAGGCCGA---CGCAAGTCCGCT---GGT---GCA-----------AGCAGA-----------------GGAGGAAAGAAACCCGCTGGA---NNNNNNAAA---NNNNNNGCAAAACCATCA--------------NNNNNN---NNNGATGTC-----------------
CAGCCCGGAGACCCTACCCCTCAAGGTCGTCGTCATCGATACAAGCCAGGAACCGTTGCCCTGAAAGAGATCCGTAAATACCAGCGCTCCTATGATCTGCTGATCCAGAAGCTTCCCTTTGCGCGACTCGTGCGTGAGGTTGCGTTGGAGCTTCTCCCCGCCGACGTGGGAGCAGAGCTGCGATGGCAGTCACATGCGATCCAGGCCCTGCAGGAAGCTGCAGAGGCTTTCCTTGTCCATCTCTTCGAGGATACAAACCTGTGCGCTCTACACGCTAAGCGTGTTACCATCATGCAGAAGGATATTCA
GCTTGCCCGAAGAATCCGCGGTGCCTGGGGTGGATTGGGT
>Aspergillus_zonatus
ATGCCGCCTAAGACGGGG---------CGAGGCCGCAAGATGCTT---GCGGCG--------------CAAGCGCGCCCACGG---------------------------------------NNNNNNNNNNNN---------------------------------------------------NNNNNNNNNNNN---GACGGTGATGGGNNN---------GCTGCCGCGGGTCCG------TCG------------------------------------------------------------------------------------------------------------------------------------ ----------------------------
TCGCGCAACGATGGCGCAACGAAACCCNNNCGAGGAGGT---CGCAAGTCG------NNN---NNN-----------ACGAGG-----------------GGAGGGAAACGTCCC-----------CCT------------------------------------GGGNNN-----------
AGCGGGNNNTCGAACATCNNNCCGGGCGATCCCACCCCGAAAGGCCGTCGTCACCGATACAAGCCAGGCACCGTCGCCCTGAAGGAAATCCGAAAGTACCAGCGCTCATATGACCTACTGTTGCGCAAGCTGCCCTTTGCACGGCTTGTCCGCGAGGTCGCCCTGGAGCTATTACCGGCCAACGTGGGCGCCGAATTGCGCTGGCAGTCGCACGCGATCCAGGCTCTGCAGGAAGCCGCCGAGGCCTTCCTGGTCCATCTCTTCGAAGACACCAATCTGTGCGCCCTGCACGCCAAGCGCGTTAC
CATAATGCAAAAGGATATCCAGCTAGCGCGGAGAATACGTGGTGTATGGGGCGGCCTGGGT
>Neosartorya_fischeri
ATGCCGCCGAAGACTGGA---------CGAGGACGTAAAGCAATT---GGGGCT-----------------------CCTCGAACGCAACCTCGA----------------------------------------------------------------------------------------------NNNNNNNNNNNN--------------------------------NNNGGTCCA------TCTAAT------------------GCT---GCGGCCACT-----------------------GCATCACCT---------------------------------------------------------TCC-------------------------------------------------
ACTCGGGGANNNCCGCCCAAGTCCCGC---GGA---GGA-----------ACAAGG-----------------GGGGCCAAGCGACCAGCCCGC---GCCCCA--------------------------------------AGGCCG---TCAGACGTC-----------------
CAACCTGGGGACCCAACCCCTCAAGGTCGCCGACACCGGTACAAGCCGGGCACAGTCGCATTGAAAGAGATCCGCAAATACCAGCGCTCATATGACCTCCTTATTCAGAAGCTACCGTTTGCGCGACTCGTACGCGAGGTCGCATTGGAACTTCTCCCCGCTGAAGTAGGCGCGGAGCTACGGTGGCAGTCGCACGCTATTCAGGCGCTACAGGAGGCCGCCGAAGCTTTCCTCGTCCATCTTTTCGAGGATACCAATCTCTGTGCTTTGCATGCTAAGCGTGTAACGATTATGCAGAAAGATATCCA
GCTCGCCCGAAGAATACGCGGTGTTTGGGGCGGTCTGGGA
```

Colletotrichum – CenH3 alignment inferred in Bali-Phy. All the codons with reliability score below 80% are masked with NNN.

>Colletotrichum_eremochloae
ATGCCTCCCAAGAAGAGACAGTCAGACGCTCCGCGTCGGAAATCTCGTCAGAGCGACGTCCAACCTGGCGACCCTATCCCCAACCGAGGAAGGCGACGTTACAAGCCCGGTACAGTTGCTCTCAGGGAGATCCGAAAGTATCAAAGTGGCACTCAGCTTCTGCTGCGCCAGCTTCCATTCTCTCGCCTTGTACGCGAGATCGCCGAATCGGTGAGACCCCGAGGCGAAGCGATGCGATGGCAATCACAAGCGATACAAGCACTACAAGAAGCTGCGGAGGCTTTCTTGGTACACCTCTTCGAAGAC
ACCAACCTTTGCGCTATCCACGCCAAGAGAGTAACCATAATGCAAAAAGATATTCAGCTCGCAAGGCGCATTCGGGGCGTCTGGGGTGGCCTAGGT
>Colletotrichum_falcatum
ATGCCCTCCAAGAAGAGACAGTCAGACGCTCCGCGTCGGAAATCCCGTCAGAGCGACGTTCAACCTGGTGATCCTATTCCCAACCGAGGAAAGCGACGCTACAAGCCTGGTACAGTTGCTCTCAGGGAGATCCGAAAGTACCAAAGTGGCACTCAACTTTTGCTGCGTCAACTTCCATTCTCCCGCCTTGTACGCGAGATTGCCGAGTCGGTGAGACCCCGAGGCGAAGCAATGCGATGGCAATCACAAGCGATACAAGCACTACAAGAAGCTGCGGAGGCTTTCTTGGTACACCTCTTCGAAGAC
ACCAACCTCTGCGCTATCCACGCCAAGAGAGTAACCATAATGCAAAAAGATATTCAGCTCGCAAGGCGCATTCGGGGCGTCTGGGGTGGGCTAGGC
>Colletotrichum_higginsianum
ATGCCTCCTAGGAAGAGACAGTCGGACGCTTCACGTCGAAAATCGCGCCAGAGTGATGTACAACCTGGTGACCCGATTCCCAACCGAGGCAAGCGACGCTACAGACCCGGCACAGTTGCTCTCAGGGAAATCCGAAAATACCAGAGCGGCACCCAGCTTTTGCTGCGTCAGCTTCCCTTCTCTCGACTTGTCCGCGAAATTGCCGAGTCGGTGAGACCCCGGGGAGAGGCGATGCGGTGGCAATCACAAGCAATACAGGCACTTCAAGAAGCCGCCGAGGCTTTCTTGGTTCACCTATTTGAAGAT
ACAAACCTCTGCGCTATCCACGCCAAGAGAGTGACCATTATGCAAAAGGATATTCAGCTTGCCAGGCGCATTCGGGGCGTCTGGGGTGGATTGGGT
>Colletotrichum_somersetensis
ATGGCCCCCAAGAAGAGACTGTCAGACGCTCCGCGTCGGAAATCCCGTCAAAGCGACGTACAACCTGGCGATCCTATTCCCAACCGAGGAAAGCGACGCTACAAGCCTGGTACAGTTGCCCTCAGGGAGATCCGAAAGTATCAAAGTGGCACTCAGCTTCTGCTGCGCCAACTTCCATTCTCCCGGCTTGTACGCGAAATTGCCGAGTCGGTGAGACCCCGAGGCGAAGCAATGCGATGGCAATCACAAGCGATACAGGCGCTCCAAGAAGCTGCAGAGGCCTTCTTGGTACACCTCTTCGAAGAC
ACCAACCTCTGCGCTATCCACGCCAAGAGAGTAACCATAATGCAAAAAGATATTCAACTCGCGAGGCGCATTCGGGGCGTCTGGGGTGGGCTAGGC
>Colletotrichum_graminicola
ATGGCCCCCAAAAAGAGACATTCAGACGCTTCGCATCGGAAATCCCGTCAGAGCGACGTGCAACCCGGTGACCCTATTCCCAACCGAGGAAAGCGACGCTACAAGCCTGGTACAGTCGCTCTCAGGGAGATCCGAAAGTATCAAAGTGGCACTCAGCTTCTGCTGCGTCAACTTCCATTCTCTCGTCTTGTACGCGAAATTGCCGAGTCGGTGAGACCCCGAGGCGAAGCAATGCGATGGCAATCGCAAGCGATACAGGCACTCCAAGAAGCTGCGGAGGCCTTCTTAGTACACCTCTTCGAAGAC
ACCAACCTCTGCGCTATCCATGCCAAGAGAGTAACCATAATGCAAAAAGATATTCAGCTCGCAAGGCGCATTCGGGGCGTCTGGGGTGGGCTAGGC
>Colletotrichum_sublineola
ATGCCTCCCAAGAAGAGACAGTCAGACGCTCCGCGTCGGAAATCTCGTCAGAGCGACGTCCAACGTGGCGACCCTATCCCCAACCGAGGAAGGCGACGTTACAAGCCCGGTACAGTTGCTCTCAGGGAGATCCGAAAGTATCAAAGTGGCACTCAGCTTCTGCTGCGCCAGCTTCCATTCTCTCGCCTTGTACGCGAGATCGCCGAATCGGTGAGACCCCGAGGCGAAGCGATGCGATGGCAATCACAAGCGATACAAGCACTACAAGAAGCTGCGGAGGCTTTCTTGGTACACCTCTTCGAAGAC
ACCAACCTTTGCGCTATCCACGCCAAGAGAGTAACCATAATGCAAAAAGATATTCAGCTCGCAAGGCGCATTCGGGGCGTCTGGGGTGGCCTAGGT
>Colletotrichum_zoysiae
ATGGCCCCCAAGAAGAGACAGTCAGACGCTCCGCGTCGGAAATCCCGTCAAAGCGACGTACAACCTGGCGACCCTATTCCCAACCGAGGAAAGCGACGCTACAAGCCTGGCACAGTTGCCCTCAGGGAGATCCGAAAGTATCAAAGTGGCACTCAGCTTCTGCTGCGCCAACTTCCATTCTCCCGGCTTGTACGCGAAATTGCCGAGTCGGTGAGACCCCGAGGCGAGGCAATGCGATGGCAATCACAAGCGATACAGGCGCTCCAAGAAGCTGCAGAGGCCTTCTTGGTACACCTCTTCGAAGA
CACCAACCTCTGCGCTATCCACGCCAAGAGAGTAACCATAATGCAAAAAGATATTCAGCTCGCGAGGCGCATTCGGGGCGTCTGGGGTGGGCTAGGC

Penicillium – CenH3 alignment inferred in Bali-Phy. All the codons with reliability score below 80% are masked with NNN.

```
>Penicillium_bilaiae
ATGCCCCCC---NNNGCCCGC-----------AAA--------------------------------ACA-----------GGC-----------CCA-----------GCAAGC---------CCA-----------GCTCAA---------------------------------------------------------------------------------------------------------------------------------------------------------------------------------------------------------------GCG--------------CGCAAA---ACC----------------------------------------------------------NNNNNNNNNNNNNNNNNN--------------------------------
AAAGCAGCCTCAAAG------
NNNNNNNNNAATGTCCAACCCGGTGATCCCACACCCCAAGGTCGCCGCCGGCGCTACAAGCCCGGCACCGTCGCCTTGAAAGAAATCCGCCGTTACCAACGCTCCTACGACCTATTAATCGCCAAACTGCCCTTTGCGCGCTTAGTCCGCGAAGTCGCACTGGATCTCCTCCCCGCAGAAGTTGGCGCCGAGCTACGATGGCAATCACAAGCTATCCAGGCGCTGCAAGAAGCCGCGGAGGCCTTCATGGTGCATTTATTCGAAGATACAAATCTGTGCGCGATACATGCGAAGCGCGTGACGATT
ATGCAGAAGGATATTCAGCTTGCGCGTCGGATTCGAGGAGCGTGGGGTGGTCTAGGC
>Penicillium_brevicompactum
ATGCCACCGAAGATGGCG--------------------AAGATCTTGAAGAAGAAAACCACCGAT-----------------------------------------------AGC----------------------CCACAAAAG----------------------------ACC---AAAGGTCGCAGANNNNNNNNNNNNNNN------------------------------------------------------------------------------------------------------GATGGCGATGGCTCACCA---------AATGCCGTG------CGCAAA---TCC-----------------AAG---CCC--------------NNNAAACAA---
AAAGCCAACAAGNNN-----------------------------------------------------------------
GCAGATGTCCAACCGGGTGATCCAACCCCCACAGGCCGCCGCCGCCGCTACAAGCCTGGCACCGTGGCACTCAAAGAGATCCGCAAATACCAACGCTCCTACGACCTCCTCATAGCTAAGCTGCCGTTCGCGCGACTCGTTCGCGAAGTCGCTCTCGATCTTCTGCCCGCCGACACGGGCGCCGAACTGCGTTGGCAGTCACATGCGATCCTGGCACTGCAAGAAGCCGCCGAGGCATTCCTGGTTCATTTGTTCGAGGACACAAATCTGTGTGCGATCCACGCCAAGCGCGTCACAATTATGCAAA
AGGATATCCAGCTTGCGCGGAGAATTCGCGGGGTCTGGGCTGGTCTGGGT
>Penicillium_canescens
ATGCCACCCAAAATGGCACGA-----------AAG--------------------------------TCA-----------GCA-----------CCA-----------GGCAGC----------------------GCACAANNN------------------------------------NNNCGCAAAACAGTCGCGACA--------------------------------------------------------------------------------GCTGCCGCA--------------------NNNNNN------TCACCAATACAAGCG--------------CGCAAAATCNNN-----------------AAA--------------------NNNAAGCAANNN------
GCAAAGACAGCA-------------------------------------------------------------
GCCGATGTCCAACCGGGCGACCCAACCCCTACAGGGCGCCGCCGCCGCTACAAACCAGGCACAAAAGCCCTAAAAGAAATCCGCCAATACCAACGCGGCTACGAGCTGCTCATCGCGAAACTACCCTTTGCCCGCTTAGTCCGTGAAGTGGCGCTCGATCTTCTCCCCGCCGACGTCGGTGCCGAATTGCGCTGGCAGTCGCATGCTATTCAAGCACTGCAGGAGGCTGCCGAGGCTTTCCTTGTTCATCTATTTGAGGATACGAATCTTTGCGCATTGCATGCTAAGCGGGTTACTATTATGCAGAA
AGATATTCAGCTTGCGAGGAGAATTCGGGGTGTGTGGGGTGGTTTAGGG
>Penicillium_expansum
ATGCCACCCAAGACAGCTCGC-----------AAG--------------------------------ACA-----------GGG-----------CCG-----------GGCAGC----------------------GCGCAAAAA----------------------------GGC---AAAGTGCGCAAGACAACCGCCGCG--------------------------------------------------------------------------CCCGCCGCC--------------------NNNGGAGATGGAACACCAACAAAAGCA--------------CGCAAG---ACG-----------------AAGGGA-----------------
GGAAAGCAATCAAAAGTC---------AAC-------------------------------------------------------------
GCCAACGTCCAACCCGGAGACCCAACCCCCACAGGCCGGCGCCGGCGCTACAAGCCCGGCACCGTCGCATTGAAAGAAATCCGCCGCTACCAACGCTCCTACGACCTCCTAATCGCCAAACTCCCCTTTGCACGCTTAGTCCGCGAAGTCGCACTCGATCTCCTCCCCGCCGACGTCGGCGCAGAGTTGCGCTGGCAGTCGCATGCTATCCAAGCTCTGCAGGAGGCCGCCGAGGCTTTCCTTGTTCATCTTTTCGAGGATACAAATCTCTGCGCGCTGCACGCGAAGCGCGTTACTATTATGCAGAA
GGATATCCAGCTTGCGCGGAGAATTCGCGGTGTCTGGGCTGGGCTTGGT
>Penicillium_fellutanum
ATGGCCACCAAGACAGCANNN------------NNN-----------------------------GCGNNN-----------GCG-----------CCG-----------GCCAGC---------CCG-----------GCGCAAAAT----------------------------AGCCCA----------------------GCGCAGAAGCGTAAGCGCGGACCGACTGTATCATCGCCTTCCAAACAGAAG-----------------------------------------------------------------------------------------------NNN---NNN-----------------NNN------GGA-----------------------------
NNNAAGACANNNAAA--------------------------------AAGCCAGCG------------
AGAGCANNNAATGTCCAGCCCGGCGACCCAACGCCCTCTGGTAAACGCCGCCGCTACAAGCCCGGCACTGTTGCCCTCAAGGAAATCCGACGCTACCAACGCTCTTACGATCTCTTAATCGCGAAACTCCCCTTTGCCCGACTCGTCCGCGAGGTCGCACTCGACCTTCTCCCCGCTGAAGTCGGCTCCGAGCTGCGCTGGCAGTCGCACGCTATCCAGGCATTGCAAGAAGCCGCCGAGGCATTTCTCGTGCATCTCTTCGAAGATACCAACCTCTGTGCGCTACATGCCAAGCGCGTCACAATCAT
GCAGAAGGATATCCAGCTTGCGCGGCGGATAAGAGGCGTGTGGGGAGGTCTTGGA
>Penicillium_glabrum
ATGCCCACCAAATCANNN-----------------------------------------------------------------AAACGATTGATTCCT-----------GGGAGC---------CCT-----------GCA---NNN----------------------------NNNNNN--------------------------------------------------------------------------------------GGAAGACCTCGCAAGAGCGCANNNNNNNNNGAAACAACCGAA-----------------GGATCACCGAGCGCAGCG--------------CGCAAG---TCA-----------------ACAGCT-----------------GGCAAA------
AAAGTGACAAAANNNNNN-----------------------------------------------
GCAAAGCGAACANNNAATGTCCAACCCGGTGATCCCACACCCACAGGCCGTCGTCGCCGATACAAGCCCGGCACCGTCGCATTGAAGGAGATTCGCCGCTACCAACGCTCTTACGACCTCTTGATTGCTAAGCTTCCATTCGCCCGTCTCGTCCGTGAAGTCGCACTCGACCTCCTCCCCGCAGAGGTGGGCGGCACGTTGCGCTGGCAATCCNNNGCAATCCAGGCATTGCAGGAGGCTGCAGAAGCTTTCCTGGTGCATTTATTCGAGGATACCAACCTTTGTGCGCTGCATGCGAAGCGAGTTA
CCATTATGCAGAAGGATATTCAGCTTGCGCGCAGAATTCGGGGAGTTTGGGCAGGTCTCGGT
>Penicillium_chrysogenum
ATGCCACCCAAGCTAGCCAGC-----------AAG--------------------------------ACA-----------GCA-----------CCA-----------GGCAGC----------------------GCACAAAAA----------------------------AAC---AAAGTGCGCAAGACAGTCGCTGCG--------------------------------------------------------------------------CCGGCCGCC-----------------------NNNGATGGAACACCAACCAAAGCA--------------CAGAAA---ACG-----------------AAGGGA-----------------
GCAAAGCAATCAAAAGTCGCAAAGACCACC-------------------------------------------------------------
GCCAACGTCCAACCCGGAGACCCAACCCCCACAGGCCGCCGACGCCGCTATAAGCCCGGCACCGTCGCTTTGAAAGAAATCCGCCGCTACCAACGCTCATACGACCTACTCATCGCTAAACTTCCCTTTGCGCGCTTAGTCCGCGAAGTCGCGCTCGATCTCCTCCCCGCCGACGTCGGCGCCGAGTTGCGCTGGCAGTCGCATGCCATCCAAGCTCTGCAGGAGGCCGCTGAAGCTTTCCTCGTCCACCTCTTCGAGGATACGAATCTATGCGCGCTACATGCGAAGCGCGTAACTATTATGCAGAA
GGATATCCAGCTTGCGCGGAGAATTCGCGGTGTCTGGGCTGGGCTTGGT
>Penicillium_janthinellum
ATGCCCCCA---NNNGCAAAA-----------AAG--------------------------------ACC-----------GCC-----------GCC-----------GCCGGC---------------------------------------------------------------------------------------------------------------------------------------------------------------------------------------------GCG--------------CGCAAG---ACA-----------------ATANNN------AAGAAAGCCGTCNNNNNN------NNNNNNNNN---------------------------------------------
------------------------------
AATGTACAACCCGGCGACCCAGCCCCGACAGGCCGCAAACGCCGCTACAAGCCCGGCACCGTCGCCCTAAAAGAAATCCGCCGCTACCAACGCTCCTACGACCTTCTCATCGCCAAGCTGCCCTTCGCCCGACTGGTCCGCGAAGTCGCGCTGGACCTGCTCCCCGCCGAAGTTGGCGCCGAGCTCCGGTGGCAGTCGCATGCGATTCAGGCGCTTCAGGAAGCCGCTGAGGCGTTTATGGTGCATTTATTCGAGGACACGAATTTGTGTGCGATTCATGCGAAGCGGGTTACGATTATGCAGAAG
GATATTCAGCTTGCGAGGAGGATTCGCGGGGTTTGGGGGGGATTGGGT
>Penicillium_lanosocoeruleum
ATGCCACCCAAGTTAGCCAGC-----------AAG--------------------------------GCA-----------GTA-----------CCA-----------GGCGGC----------------------GCGCAAAAA----------------------------AGC---AAAGCGCGCAAGACAGTCGCTGCG--------------------------------------------------------------------------CCGGCTGCC-----------------------NNNGATGGAACACCAACCAAAGCA--------------CAGAAT---ATG-----------------AGGGGA-----------------
GGAAAGCAATCAAAAGTCGGAAAAATCACC-------------------------------------------------------------
GCGACCGTCCAACCCGGAGATCCAACCCCCACAGGCCGCCGACGCCGATATAAGCCCGGCACCGTCGCTTTGAAGGAAATCCGCCGCTACCAACGCTCATACGACTTACTCATCGCTAAACTTCCCTTTGCGCGCTTAGTCCGCGAAGTCGCGCTCGATCTCCTCCCCGCAGATGTCGGCGCCGAGTTGCGCTGGCAGTCGCATGCCATCCAAGCTCTGCAGGAGGCCGCTGAAGCTTTCCTCGTCCACCTCTTTGAGGATACGAACCTCTGCGCGCTACATGCGAAGCGCGTAACTATTATGCAGAA
GGATATCCAGCTTGCGCGGAGAATTCGCGGTGTCTGGGCTGGGCTTGGT
>Penicillium_marneffei
ATGCCACCAAAAACAGGCCGAGGGCGCTCTTCCNNNGCC----------------------------NNNACCACCAGCAGCNNN-----------NNNAACTCTCCCAGANNNNNNAGAGCTGGCNNNTCATCCCGTCAANNNNNNNNNNGAAACAACCACAACATCAAGTCCCATCACANNNCCA---------------------------------------------------------------------------------------------------------NNNTCACCAACTACCGCC--------------AGAAAT---
ACGAAGCAGCCAATGTCCCCANNNNNNN-----------------NNNNNN------NNNNNNGCAAAAACANNNNNNAGAGTAACCAAGCGACCAGCCGGCAAAGCCCCG--------------------
CGATCANNNAACATACAACCGGGCGACCCAACCCCCATCGGCAAACGCCGCCGCTACAAGCCCGGCACCGTCGCCCTCAAAGAAATCCGCAAATACCAACGCTCCTTCGATTTGTTGATTAGCAAACTCCCCTTTGCCCGCTTAGTTCGCGAAGTCGCACTAGACCTGCTACCAGCCGAAGTAGGCGCCGAACTACGATGGCAATCGCACGCAATTATGGCGCTGCAAGAAGCTGCAGAGGCGTTTTTGGTGCATCTGTTTGAGGATACGAATCTGTGCGCCATTCATGCGAAGAGGGTTACGATTA
TGCAGAAGGATATTCAGCTTGCCAGGCGGATTAGGGGGATGTGGGCTGGTTTGGGC
>Penicillium_raistrickii
NNN-----------GCCCGC-----------AAA--------------------------------ACC-----------GCA-----------CCT-----------GGAAGC----------------------GCTCAAAAA----------------------------GCC---AAAGCTCGCAAGACAGCCGCAACC------------------------------------------------------------------------CCCGCAGCC------------AACGACGGCGACGGCGACGGGTCCCCA-----------------GTCATCNNNAAA---ACA-----------------AAA--------------------NNNAAACAA---
AAAGTCACCAAGNNN-----------------------------------------------------------------
TCCAACGTCCAGCCGGGCGATCCAACCCCCACAGGCCGCCGTCGCCGCTACAAACCCGGTACCGTGGCACTGAAAGAAATCCGCAAATATCAACGATCCTACGACCTGCTTATCGCCAAGCTCCCATTCGCGCGACTGGTGCGCGAAGTCGCACTCGACCTCCTCCCCGCCGATACAGGGTCTGAGCTGCGCTGGCAATCGCATGCAATTCTGGCGCTGCAGGAGGCGGCCGAGGCATTCCTGGTTCATTTGTTCGAGGATACGAATCTGTGCGCTATTCACGCTAAGCGGGTTACTATTATGCAAAA
GGATATTCAGCTCGCGCGGAGAATACGCGGTGTTTGGGCTGGATTGGGC
```

Saccharomyces – CenH3 alignment inferred in Bali-Phy. All the codons with reliability score below 80% are masked with NNN.

```
>S_arb
ATGTCAAGCAAACAGCAATGGNNN---------ATCCAAAGTGATTCGAGT------------------------------------------------------------------------------------------------GGGAGATCTCTCAGTAATGTCAATAGGCTTGCA-------------------------------------------GGAGAT-----------CAACAGTCTATTAACGACCGTGCGCTATCTTTATTGCAAAGGACAAGAGCAAGGAAGAATCTTTTCCCAAGAAGGGAGGAAAAGAGACGATATGAAGGTCCA------------CAGGATGACATAGATTTTGAAGCA----
------------------------------------------------------------GAGTATGGAAACCAGGCAGGAAATTTGGAAACTGAGACAGAAAATGAGGATGAAACTGAGATGGAAACTGAAGCACCAACTGCCACGATAACTCATTCTTATGCCCTAGATAGGTACGTTAGGCAA------------------------------------------------------AAAAGAAGACAAAAGCAAAGAAGGCAAGACCTGAAG------------------------------------------------------CGCGTCGAA------------------------------------------------------------
------------------------------------------------------
AAGAAGTATAGCCCTAGTGAGTTAGCTCTGTATGAAATTCGAAAATACCAGCGTTCTACAGATTTACTTATCTCCAAAATTCCATTCGCAAGGTTAGTGAAGGAAGTTACGGATGAATTTACAACTAAAGACCAAGATTTGCGTTGGCAGTCAATGGCGATCATGGCGTTACAAGAAGCAAGTGAAGCATACTTGGTGGGGTTATTGGAGCACACCAACCTCTTGGCGTTGCATGCGAAAAGAATCACCATAATGAAGAAGGATATGCAACTCGCAAGGAGAATCAGAGGACAGTTTATT
>S_bay
ATGTCTAGCAAACAAGAATGGGCTAACTCGGCCATTCAAAGTGAATCGAGT------------------------------------------------------------------------------------------GGAAGATCACTCAGTAATGTCAATAGGCTTGCA-------------------------------------------GGAGAT-----------CAACAGTCGATTAATGATCGTGCGTTATCCTTGTTACAAAGAACAAGAGCAAGGAAGAACCTGTTCCCAAGAAGAGAAGAAAGAAGACGGTATGAAGGTTCA------------
CAGGATGACGTAGAATTTGAGGCA------------------------------------------------------------------GATTACGAAGACCATGCAGGAAATCTCGAAACTGAAGCAGAAAACGAAAATGAAACAGAAGTAGAAGAAGAATTATCGACGGCTAAGCAAACTCACTCGTACGCCTTAGATAGATATGTTAGACAA------------------------------------------------------AAAAGAAGACAAAAACAAAGGAAGCAGGGCTTGAAG------------------------------------------------------CGTGTGGAA---------------
---------------------------------------------------------------------------------------------------------
AAGAAATATAGCCCTAGCGAATTAGCTCTTTACGAAATTCGAAAATACCAGCGTTCCACAGATTTGCTAATCTCCAAAATCCCATTTGCAAGACTAGTCAAGGAAGTCACGGATGAGTTTACAACCAAAGATCAAGACCTGCGTTGGCAGTCAATGGCGATCATGGCGCTACAGGAAGCAAGTGAAGCATATTTAGTGGGATTATTAGAGCATACCAACCTCTTAGCATTGCATGCGAAAAGGATAACCATAATGAAAAAAGACATGCAACTGGCAAGGAGAATTAGAGGACAATTCATT
>S_cas
ATGTCAAGCAGA----------------------------------------AAATTTGTAGAACAAGGCCATGCTCAGAATTCTAGTAGTCATCTGTTCTCGAATATCCTCGATAATGAT-----------------------NNNGGATCATTGAGCAATATAAATAGATTANNNCTGGACCCGGATAATACAGAAGATTTATTACAGCAAGAAGTC------------------------------ATCAACGAGCGGGCACTTTCTCTTTTACAAAGGACTAGAGAACGTAGAAACTTATTACACCGATTTGAAGATAAAAGAAGGTAT---------
TATAACCAAGGGNNNNNNNNNNNNNNNNNNNNNNNN---TCTGTGGCCAGTAGTCATTATCGTTCTAATGATGTGGGTGGGAACTTTCAATTTTTTGATCAAGAANNNNNNNNNNNNNNNNNNNNNNNNNNN---------------------------------------------------------------------------------------------------GCCATTGATGACGATTATGGAACCCTAGATCAATCCAACATTATCGATCGTCACCAANNNNNNNNNN--------------------------
AAAGGAAACAAACATTCTCGTCAAGAAAGACACCATCAAAGAGAACTCAAACAACGTGTTGAAAAGATTAGAACTCAGAGACAGGTCGGAAATACG-------------------------------------------------------------------------------------
NNNAAATTTACTCCAAGTAGTTTAGCCCTATATGAGATAAGGAAATACCAACGATCTTCAGAACTGTTGATATCGAAGATTCCCTTTACTAAATTAGTGAAGGAAGTCACTGATGAATTTACCGTGGAAGACCAACAACTTCATTGGCAATCGATGGCCATTGTCGCGTTACAGGAAGCCAGTGAAGCTTATTTAGTGGGTTTATTGGAGCATGCCAATTTATTAGCCATACATGCCAAGAGGATCACATTAATGAAGAAGGATATACAGTTGGCCAGAAGAATTAGAGGTCAATTCATT
>S_cer
ATGTCAAGTAAACAACAATGGGTTAGTTCTGCTATTCAAAGTGATTCGAGT------------------------------------------------------------------------------------------GGAAGATCACTCAGTAACGTCAACAGGCTTGCA-------------------------------------------GGAGAC-----------CAACAATCTATTAACGATCGTGCGTTATCGTTATTGCAGAGAACAAGAGCGACAAAGAACCTGTTTCCAAGAAGAGAGGAAAGAAGACGTTATGAAAGCTCA------------
AAAAGTGACCTAGATATCGAAACA------------------------------------------------------------------GACTACGAAGACCAAGCAGGTAATCTAGAAATCGAGACAGAAAATGAAGAAGAAGCTGAAATGGAAACTGAAGTACCTGCACCAGTGCGAACTCATTCATATGCCTTAGACAGATATGTTAGACAG------------------------------------------------------AAAAGGAGGGAAAAACAAAGAAAGCAGAGCTTAAAG------------------------------------------------------CGCGTCGAA----------------
---------------------------------------------------------------------------------------------------------
AAGAAATATACTCCTAGTGAATTAGCTCTGTACGAAATTCGAAAATACCAACGTTCCACGGATTTATTAATCTCCAAAATTCCATTTGCAAGGCTAGTGAAAGAAGTTACAGACGAGTTTACAACTAAAGATCAGGATTTACGTTGGCAGTCAATGGCGATTATGGCGTTACAGGAAGCAAGCGAAGCGTATCTGGTAGGATTATTGGAACATACAAACCTCTTGGCGCTGCATGCAAAAAGAATTACTATAATGAAGAAAGACATGCAACTAGCAAGAAGAATCAGGGGACAGTTTATT
>S_klu
ATG---NNNNNNNNNNNNNNNN---------------------------------------------------------------------------------------------------AATGCTAGTATATCCAGCCAGACAGGCAGATCACTCAGTAACACCAATCGTCTG-------------------------------------------GGCNNN---CAAGAGCCCTCTCAACAATCTATCAACGAGAGGGCAATATCGCTTCTACAGCGTAATAGGGCCAGGAGGAAGATGTTACAAAGGCAAGAGGATAGAGAGCGATAC------------------------------------------------------------
---------------------------------------------------------------------------------------------------------------------------------------------------------------------------------------------------------------------------------------------------------------------------------------
GTAAGGCGTAATGAACTACCAGAGGAACAGCCTCCAACTGCGATAGGTACCATCAGAAGCAATATAAAAAAATCCAAGAGTCAGCGTNNNNNNTACAAACCAAGTGATGTGGCTTTGCAAGAGATTAGAAAGTACCAACGAAGCACTGAGCTTTTGATATCGAAAATGCCATTTGCCCGTCTCGTAAAAGAGGTCACTGAACAATTTAGTACAGACGAACAGCAGCTCCGGTGGCAGTCTATGGCTATCCTAGCATTACAGGAGGCTAGTGAAGCGTACTTGGTTGGTCTACTGGAGCACACAAA
CCTGTTAGCCTTACACGCTAAAAGAATTACCATTATGAAGAAAGATATGCAGCTGGCAAGGAGAATTAGGGGCCAGTTTATT
>S_kud
ATGTCAAGCAAACAGCAATGGGCTAATTCGGCCATTCAAAGTGATTCGAGT------------------------------------------------------------------------------------------GGAAGGTCACTCAGCAATGTGAACAGGCTAGTA-------------------------------------------GAAGAT-----------CAACAGTCTATTAATGACCGTGCACTATCTTTGTTACAAAGAACAAGAGCAAGAAAAAATCTTTTTCCAAAAAGAGAAGAAAGAAGACGATATGAAGGTCCA------------
CAAGATGACATGATTTTTGAAGAA------------------------------------------------------------------AACCATGAAGGCCAAGCGGAAAACTTGGAAACAGAGACAGAAAACGAGGATGAACGTGAAATGGAAACAGAGGTTCCAGACGCTACCCGAACACATTCATACGCTCTAGATAGATATGTTAGGCAG------------------------------------------------------AAAAGGAGACAAAAACAAATGAAGCAGGGTTTGAAA------------------------------------------------------CGCGTAGAA----------------
---------------------------------------------------------------------------------------------------------
AAGAAATACAGTCCCAGTGAATTAGCTCTGTACGAAATCAGAAAATATCAACGTTCTACAGATTTGCTTATCTCTAAAATTCCATTTGCAAGACTGGTAAAAGAAGTTACAGACGAGTTTACAACTAAAGACCAAGATTTGCGCTGGCAATCGATGGCGATCATGGCGCTACAGGAGGCAAGTGAAGCATACTTGGTGGGGCTATTGGAGCATACCAACCTACTTGCGCTGCATGCGAAGAGAATAACCATAATGAAGAAGGATATGCAACTAGCAAGGAGAATTAGAGGACAGTTCATT
>S_mik
ATGTCAAGCAAACAGCAATGGGCTAATTCTGCTATTCAAAGTGATTCAAGT------------------------------------------------------------------------------------------GGAAGATCACTCAGCAATGTCAACAGACTTGTT-------------------------------------------GGAGAG-----------CAACAATCCATTAACGATCGTGCATTATCATTATTGCAAAGGACAAGAGCAAGAAAGAATCTATTTCCAAGAAGAGAAGAAAAGAGACGATATGAGAACCCA------------
AAGGATGACGTAGATTTTGAAACT------------------------------------------------------------------GACTATGATGGCCAAGCAGATGATTTGGAGATAGAAACTGAGAACGAAGAAGAAACTGAAGTGGCTACTGAAGTATCAACGTCCGCGCGAACACATTCGTATGCCTTGGACAGATACGTTAGACAG------------------------------------------------------AAAAGAAGAGAAAAGCAAAGGAAACAGGGCTTAAAG------------------------------------------------------CGTATTGAA-----------------
---------------------------------------------------------------------------------------------------------
AAGAAGTACAGTCCAAGTGAATTAGCTCTATATGAAATTCGAAAGTACCAACGTTCCACGGATTTATTGATTTCCAAAATTCCTTTTGCAAGGCTTGTAAAAGAAGTTACGGATGAGTTCACGACTAAGGATCAAGATTTACGCTGGCAGTCAATGGCGATCATGGCACTACAAGAAGCAAGTGAAGCCTATTTGGTGGGTCTATTGGAGCATACCAACCTTTTGGCATTGCACGCGAAAAGGATAACTATAATGAAGAAGGACATGCAACTAGCAAGGAGAATCAGAGGACAATTCATT
>S_par
ATGTCAAGTAAACAGCAATGGGCTAGTTCTGCTATTCAAAGTGATTCGAGT------------------------------------------------------------------------------------------GGAAGATCACTCAGTAATGTCAACAGGCTTGCA-------------------------------------------GGAGAC-----------CAGCAGTCTATTAACGATCGTGCGTTATCGTTACTGCAAAGGACGAGAGCAAGAAAGAACTTACTACCCAGAAGAGAAGAAAGAAGGCGTTATGAGAGCTCA------------
AAAAATGACCTAGATTTTGAAACA------------------------------------------------------------------GACTACGAAGACCAAGCAGGTAATCTGGAAACTGAAACAGAGAATGAAGAAGAAGCTGGAATGAAAACCAAGGCACCAATGTCCGCCCGAACTCATTCGTATGCTTTAGACAGATACGTTAGGCAG------------------------------------------------------AAAAGAAGGGAAAAGCAAAGAAAGCAGGGCTTGAAG------------------------------------------------------CGTGTCGAA----------------
---------------------------------------------------------------------------------------------------------
AAGAGATATAGTCCTAGTGAATTAGCTCTGTACGAAATTCGAAAATATCAACGTTCTACAGATTTATTAATTTCCAAAATTCCATTTGCAAGGCTAGTGAAAGAGGTTACAGATGAGTTTACAACCAAAGATCAAGATTTACGTTGGCAGTCAATGGCGATTATGGCGCTGCAGGAGGCAAGTGAAGCATACTTAGTAGGGTTACTGGAACATACCAACCTCTTGGCGTTGCATGCGAAAAGAATAACTATAATGAAGAAAGATATGCAACTAGCAAGAAGAATTAGGGGACAGTTCATT
>S_pas
ATGTCTAGCAAACAAGAATGGGCTAACTCGGCCATTCAAAGTGAATCGAGT------------------------------------------------------------------------------------------GGAAGATCACTCAGTAATGTCAACAGGCTTGTA-------------------------------------------GGAGAC-----------CAACAATCGATTAATGATCGTGCGTTATCCTTGCTGCAAAGAACAAGAGCAAGGAAGAACCTGTTTCCAAGAAGAGAAGAAAGAAGACGATATGAAGGTCCT------------
CAGGACGACGTAGAATTTGAGGCA------------------------------------------------------------------GATTATGAGGATCATGCAGGAAATCTCGAAACGGAAACAGAAAACGGAGATGAAACTGAAGTAGAAGCAGATGTGTCAACGGTTAAGCAAACTCACTCGTACGCCTTAGATAGATATGTTAGACAA------------------------------------------------------AAACGGAGACAAAAACAAAGGCAGCAGGGCTTGAAG------------------------------------------------------CGTGTGGAA---------------
---------------------------------------------------------------------------------------------------------
AAGAAATATAGTCCTAGCGAATTAGCTCTCTACGAAATTCGAAAATACCAGCGTTCTACAGATTTACTAATCTCTAAAATTCCATTTGCAAGATTAGTTAAGGAAGTCACGGATGAGTTTACAACCAAAGATCAGGACTTGCGTTGGCAGTCTATGGCGATCATGGCGCTCCAAGAAGCAAGTGAGGCATATTTGGTGGGATTATTAGAGCATACCAACCTCTTAGCTTTACATGCGAAAAGGATAACCATAATGAAAAAAGACATGCAACTAGCAAGGAGAATTAGAGGACAATTTATT
```

Trichoderma – CenH3 alignment inferred in Bali-Phy. All the codons with reliability score below 80% are masked with NNN.

```
>Trichoderma_asperellum
ATGGCT--------------------------------------------CCTCGCCAATCAACTTCAGGAACCCAACAACGCTAT---------------
NNNGCCAGACCAAGCGACATTCAGCCGGGCGATCCACTACCTACGAGACAGAAACGTCGATATCGCCCAGGCACCGTTGCGCTACGAGAAATCAGGCAATATCAAGCCAATACAAAGCTTCTACTTCTAAAACTTCCGTTTATGCGACTCGTCCGTGAAATTGGATTAAATTGTCGCCCGAGAGGAAAAGAATTTAGATGGCAGAGCCAGGCAATCCAGGCACTGCAAGAGGCTGCAGAAGCATTTATGGTACATCTATTTGAAGATGCACAGCTATGCGCGGTCCATGCGAAAAGAGTGACACTT
ATGCAAAAAGACATTCAGCTGGCGAGGAGGATTCGAGGTATATGGGGCGGGCTTGGT
>Trichoderma_atroviride
ATGGCT--------------------------------------------CCTCGCCAATCAACGGCAGCGCCCAAACAACGATAT---------------
NNNGCCAGACCAAGCGACATACAGCCGGGCGATCCACTGCCTACGAGACAGAAGCGGCGATATCGCCCGGGCACCGTTGCGCTGCGAGAAATCAGGCAATATCAAGCCAATACAAAGCTTCTACTCCTAAAACTTCCATTTATGCGACTCGTTCGCGAAATTGGATTGAATTGTCGGCCGAGAGGGAAAGATTTTCGATGGCAGAGTCAGGCCATCCAGGCACTGCAAGAGGCCGCAGAAGCATTCATGGTACATCTTTTTGAAGATGCGCAGCTATGCGCGGTCCATGCGAAGAGAGTGACACT
TATGCAAAAAGACATCCAGCTAGCGAGGAGGATTCGAGGTATATGGGGCGGGCTTGGT
>Trichoderma_harzianum
ATGGCT--------------------------------------------CCTCGTCAAAAAACTTCGGCG---------------
GCCTCACAGAAATTGCGCTCCAGACCAAGCGATGTACAGCCGGGCGATCCATTACCTACAAGACAGAAGCGACGATATCGGCCGGGCACCGTTGCGTTGCGAGAGATCAGGCAATACCAGTCAAATACAAAGCTTTTATTGTTAAAACTACCATTCATGCGCCTTGTTCGTGAAATTGGATTGAATTGTCGGCCGAGAGGGAAAGACTTCAGATGGCAGAGTCAGGCTATTCAGGCACTACAAGAGGCCGCGGAGGCCTTTATGGTACATCTTTTTGAGGATGCGCAGCTATGCGCTGTCCACGCA
AAGAGAGTTACACTTATGCAAAAAGATATACAGCTTGCGAGGAGAATCCGAGGCATCTGGGGCGGCCTTGGT
>Trichoderma_longibrachiatum
ATGCCT--------------------------------------------CCTCGTCAGTCCACGGCGGGA---------------
ACCTCACAGAAAGTGCGCTCCCGCCCGAGCGATGTACAGCCGGGCGATCCACTGCCTGCCAGACAGCGACGACGATACCGCCCGGGCACCGTCGCATTGCGGGAAATCAGGCAATACCAGGCCAACACGAAGCTTTTGCTGCTGAAGCTGCCATTTATGCGCCTCGTTCGGGAGATTGGATTGAATTGCCGTCCAAGGGGGAAAGAGTTCAGGTGGCAGAGCCAGGCCATCCAGGCGCTGCAAGAAGCGGCAGAGGCCTTCATGGTACATCTGTTTGAGGATGCGCAGCTGTGCGCTGTGCACG
CCAAGAGGGTTACCCTCATGCAGAAAGACATACAGCTTGCGAGGAGGATTCGAGGCATCTGGGGCGGCCTCGGT
>Trichoderma_reesei
ATGCCCTCACCAACCCCAAATCGTCTTCTGCTAACCCCCAACTCTTCAGCG--------------------------------------------------------------------------
GGCGACCCACTGCCTACGAGACAACGACGACGATACCGCCCCGGCACTGTCGCATTGCGGGAAATCAGGCAATACCAGGCCAACACGAAGCTTTTGCTGCTGAAGCTGCCGTTTATGCGCCTCGTTCGAGAAATTGGATTGAATTGTCGTCCGACGGGCAAAGAGTTTAGATGGCAGAGCCAGGCCATCCAGGCGCTGCAAGAAGCCGCAGAGGCCTTCATGGTACATTTGTTTGAGGACGCGCAGCTGTGCGCTGTACACGCCAAGAGGGTCACCCTGATGCAGAAAGACATACAGCTTGCAAG
GAGAATCCGGGGCATCTGGGGCGGCCTTGGC
>Trichoderma_virens
ATGGCT--------------------------------------------CCTCGTCAATCAACGTCAGGG---------------
GCTTCACAAAAATTGCGTTCCAGACCAAGCGATGTACAGCCGGGCGATCCATTACCTACGAGACAGAAACGACGATATCGCCCAGGCACCGTTGCGTTGCGAGAAATCAGGCAATACCAGTCAAATACGAAGCTGTTGCTACTAAAACTGCCATTCATGCGCCTTGTTCGTGAAATTGGATTGAATTGTCGCCCGAGAGGAAAAGATTTTAGATGGCAGAGCCAGGCCATTCAGGCGCTACAAGAGGCCGCGGAGGCATTCATGGTACATCTTTTTGAGGATGCGCAGCTGTGCGCTGTCCACGCA
AAGAGAGTTACCCTTATGCAAAAAGACATACAACTGGCGAGGAGAATTCGAGGCATCTGGGGCGGCCTTGGT
```

Bony Fish – CenH3 alignment inferred in Bali-Phy. All the codons with reliability score below 80% are masked with NNN.

```
>Esox_lucius
ATGNNNCGCCGCGATCTTNNNACTTCCACTGCAAGTCGACGCAAGGGCAAGGCACCAAAGCGTCGGCCTCCAGTCCCAGCCCTGCCCGCTTCA------------------CCACGGCGGAGTGGACCTTTAGCCTCTGCAGGTCCTNNNNNNNNNNNN------
NNNAAGAAGAAAAGGTTCCGCCCCGGCACCCGAGCTCTGATGGAGATCCGGAAGTACCAGAAGAGTACCGACTTGCTTCTGCGGAAGGGACCGTTTGCACGCCTGGTTCGGGAGGTGTGCCAGACGTTTAGCAGGGACTACATGAGATGGCAGGTGTATGCCCTACTGGCCTTGCAGGAAGCTGCGGAGGCTTTCCTTGTGTTATTGTTTTCTGATGCTTACCTCTGTACCATCCACGCCAAGCGTGTAACGCTGTTCCCACGAGACATTCAACTTGCGCGACGAATTCGAGGAGCAGATCATCTC
>Haplochromis_burtoni
ATGCGT------------CATAATTCATCTGCCAGCCGTCGGAAGGGTAAAACCCCTCAACGGCGCCCCCAAGTGCCAGCCCAAAGGACCCCCAGTGTAACATCC------CCAAGACGCAGTGGAGTTCCA---------------
GGACAGCCTCCTGTTTCCCCAAGAAGAGGAAATTTCGACCAGGAACCAGAGCTTTGATGGAGATCCGCAAGTACCAGAAAAGCACAGATCTCCTCCTTAGAAAGGCGCCCTTTGCCCGTTTGGTGCATGAGGTGTGCCAAAGCTTTTCTAGAGAAAATCTTCGATGGCAGGTTTATGCTCTTCTGGCCCTCCAAGAGGCTGCAGAGGCTTTTCTCGTCTTGATATTTTCAGATGCCAACCTGTGTGCCATCCACGCCAAGAGAGTCACTGTATTCCCTCGTGACATTCAGCTAGCCCGGAGGATCCGT
GGGGTGGATAATCTG
>Lates_calcarifer
ATGCGT------------CATGATTCATCTGCAAGTCGACGGAAGGGCAAAACCCCTCAACGTCGCCCCCCGGTACCCGACCCAGGGCCCTCTGGTTCCACANNNAGGACCCCAAGACGAAGCAGAGTTTCA---------------
GGCGTACCTCCTGTGTCTCCCAAGAAAAGAAGGTTTCGACCAGGAACCAAGGCCTTAATGGAAATCCGCAAGTACCAGAAGAGCACCGATCTTCTGTTAAGGAAAGGACCCTTCTCTCGTCTGGTTCGTGAGGTGTGCCAAGGTTTTTCCAGAGAGGCTCTCAGGTGGCAGGTCTATGCTCTTCTTGCCCTGCAGGAGGCTGCTGAGGCCTTTCTTGTCATGTTATTCTCAGACGCCAACCTGTGTGCCATCCATGCCAAGCGGGTAACCGTGTTCCCCCGCGACATTCAATTGGCAAGGAGGATCCG
TGGGGTGGATAACATG
>Micropterus_floridanus
ATGCGT------------CATGATTCATCAGCCAGCCGCCGGAAGGGGAAAACCCCTCAACGTCGCCCCCCGCAGCCGGCCCCAGAGACC---------------CGGTCCCCAAGACGGAGCAGAGTTTCA---------------
AGGCAGCTTCCTGCGTCTCCCAAGAAGAGGAGGTTTCGACCAGGAACCAAGGCCTTAATGGAGATCCGCAAGTTCCAGAAGAGCACTGATCTTTTGCTCAGGAAAGGACCCTTCTCTCGCCTGGTTCGTGAGGTATGCCAGAGTTTTTCCAGAGAGGCTCTCCGGTGGCAGGTCTACGCACTTCTGGCCCTGCAGGAGGCTGCAGAGGCGTTTCTCGTCATGCTATTCTCAGACGCAAATCTGTGTGCCATCCACGCCAAGCGGGTCACCGTGTTCCCCCGTGATATTCAGCTGGCCAGGAGGATCC
GCGGCGTTGATAACCTG
>Misgurnus_anguillicaudatus
ATGCGG------------CACAATAAATCAGCANNNNNNNNNNNNNNNNNNNNNACCCCCAGGCGCAGATCCCCT---CCTGCTACACCAGCAGCACCCTCCACANNNAGAACCCGACGCACCAGTGGACCATCC---------------GAGAAANNNNNN------
NNNAAGAAGCACAAGTTTCGACCTGGGACGCGAGCTCTCATGGAGATACGCAAATATCAGAAATCCACAGATCTGTTGTTGCGCAAGGGCCCGTTTTCACGACTGGTACGTGAGGTGTGTCAGACATTTAGCCGGGAGAACTTAATGTGGCAGGGCTATGCACTAATGGCTTTACAAGAGGCTGCAGAGGCGTTCATGGTCCGTTTGTTCTCTGATGCCAATCTCTGTGCTATTCATGCCAAGAGGGTGACATTGTTTCCACGTGATATACAGCTTGCCCGTCGAATCAGAGGTGTTGAAAACATG
>Neolamprologus_brichardi
ATGCGT------------CATAATATATCTGCCGGCCGTCGGAAGGGTAAAACCCCTCAACGGCGTCCCCAAGTGCCAGCCCAAAGCACCTCCAGTGTAACATCC------CCAAGACGCAGTGGAGTTCCA---------------
GGACTGCCTCCTGTTTCCCCAAGAAGAGGAAATTTCGACCAGGAACCAGAGCTTTGATGGAGATCCGCAAGTACCAGAAAAGCACAGATCTCCTCCTTAGAAAGGCGCCCTTTGCCCGTTTGGTTCATGAGGTGTGCGAAAGCTTTTCTAGAGAAAGGCTCCGATGGCAGGTTTATGCTCTTCTGGCCCTCCAAGAGGCTGCAGAGGCTTTTCTCGTCTTGATATTTTCAGATGCCAACCTGTGTGCCATACACGCCAAGAGAGTCACTGTATTCCCTCGTGACATTCAGCTAGCCCGGAGGATCCGT
GGGGTGGATAATATG
>Nothobranchius_furzeri
ATGCGT------------CACAGTTCACCGCGCAGCCGGCGGAAGGCCAAAACTCCCAGTCGTCGTGACCCACCTCCATCTCCCACAGCATCANNNNNNNNN------NNNCCAAGAAAAAGTGGTTTTTCT---------------
GGGATTCCTCCTGTGTCTCCCAAGAAAAGGAGGTACAGACCTGGAACCAGAGCCTTGATGGAGATTCGTAAGTACCAGAAGAGTACCGATCTTCTGCTCAGGAAAGCACCATTCGCTCGCTTGGTTCACGAGGTGTGCCAGAGTTATACCAGAGGCAATTTGCGGTGGCAGGTCTTCGCTCTTCTTGCCCTGCAGGAGGCAGCAGAGGCCTTCCTAGTCCTGCTGTTTTCAGATGCCAACCTGTGTGCCATTCATGCTAAGAGGGTGACTGTGTTTCCTCGGGACATTCAGCTGGCCAGGAGGATCC
GTGGAGTGGATCGTCTG
>Oncorhynchus_mykiss
ATGNNNCGTCACGATCCTNNNAGTTCTTCTGCAAGCCGGCGCAAGGGTGCGGTACCCAAGCGTCGGCCTCCAGCCTCCACCGCGACTGCTTCAACATCAAAGNNNAAGGCACCACGGCTGAGTGGACCATCGGCCTCTGCTGTTCCANNNNNNNNNNNN------
NNNAAAAATAGAAGGTTTCGCCCTGGCACCCGAGCTCTGATGGAAATTCGCAAGTACCAGAAGAGCACTGACTTGCTTTTGCGCAAGGCACCGTTTGCACGTCTGGTTCGGGAGGTGTGCCATACTTATACCAGGGACTTCATGAGATGGCAGGTGAACGCTCTTCTGGCCTTGCAGGAGGCTGCAGAGGCTTTCCTTGTTTTACTGTTTTCCGACGCCTACCTGTGTACAATCCACGCCAAGCGTGTAACGCTGTTCCCCCGTGACATTCAGCTTGCCCGGCGAATTCGAGGAGTGGATGACCTC
>Ophthalmotilapia_ventralis
ATGCGT------------CATAATCCATCTGCCAGCCGTCGGAAGGGTAAAACCCCTCAACGGCGCCCCCAAGTGCCAGCCCAAAGGACCTCCAGTGTAACGTCC------CCAAGACGCAGTGGAGTTTCA---------------
GGACAGCCTCCTGTTTCCCCAAGAAGAGGAAATTTCGACCGGGAACCAGAGCTTTGATGGAGATCCGCAAGTACCAGAAAAGCACAGATCTCCTCCTTAGAAAGGCGCCCTTTGCCCGTTTGGTTCATGAGGTGTGCCAAAGCTTTTCTAGAGAAAGGCTCCGATGGCAGGTTTATGCTCTTCTGGCCCTCCAAGAGGCTGCAGAGGCTTTTCTCGTCTTGATGTTTTCAGATGCCAACCTGTGTGCCATCCACGCCAAGAGAGTCACTCTATTCCCTCGTGACATTCAGCTAGCCCGGAGGATCCGT
GGGGTGGATAATCTG
>Oreochromis_niloticus
ATGCGT------------CATAATTCATCTGCCAGCCGTAGGAAGGGTAAAACCCCTCAACGGCGCCCCCAAGTGCCAGCCCAAAGGACCTCCAGTGTAACATCC------CCAAGACGCAGTGGAGTTCCA---------------
GGACAGCCTCCTGTTTCCCCAAGAAGAGGAAATTTCGACCAGGAACCAGAGCTTTGATGGAGATCCGCAAGTACCAGAAAAGCACAGATCTCCTCCTTAGAAAGGCGCCCTTTGCCCGTTTGGTTCATGAGGTGTGCCAAAGCTTTTCTAAAGAAAGGCTCCGATGGCAGGTTTATGCTCTTCTGGCCCTCCAAGAGGCTGCAGAGGCTTTTCTCGTCTTGATATTTTCAGATGCCAACCTGTGTGCCATCCACGCCAAGAGAGTCACTGTATTCCCTCGTGACATTCAGCTAGCCCGGAGGATCCGT
GGGGTGGATAATCTG
>Salmo_salar
ATGNNNCGTCGCGATCCTNNNGTTTCTTCTGCAAGCCGGCGCAAGGGTGCAGTACCCAAGCGTCGGCCTCCAGCCTCCACCGCGACTGCTTCAACATCAAAGNNNAAGGCACCACGGCTGAGCGGACCATCGGCCCCTGCAGGTCCANNNNNNNNNNNN------
NNNAAAAAAGAGAAGGTTTCGCCCTGGCAACCGAGCTCTGCTGGAAATTCGCAAGTACCAGAAAAGCACTGACTTGCTTTTGCGCAAGGGACCGTTTGCACGCCTGGTTCGGGAGGTGTGCCAGACTTATAGCAGGGACTTCATGAGATGGCAGGTGAACGCTCTTCTGGCCTTGCAGGAGGCTGCAGAGGCTTTCCTCGTTTTACTGTTTTCCGACGCCTACCTGTGTACCATCCACGCCAAGCGTGTAACGCTGTTCCCCCGTGACATTCAGCTTGCCCGGCGAATTCGAGGAGTGGATGATCTC
```

Drosophila – CenH3 alignment inferred in Bali-Phy. All the codons with reliability score below 80% are masked with NNN.

>erecta
ATGCCCCGGCACAATGCTGCCAAGCGT---------AACCCC------------------AACCCATCAATG------------------AACAACTCAAAGCCTCCAAGCGACGACGACAAAGCATTCCGCTCGCCGGAGCCAGAAGACGACACCGACTACGGCCTGGAGTTCACCACCAGCCAACTGACGCTACAGGAGGGC---TACAATCGTCGTTGCTCCACGATGCGCNNN---------------------------------------CAGCCAGCGACCCGCACTTACACCNNNNNNNNNNNNNCAGNNNGAC------
CAGGAGAATCGCCATCCGGCAGCCAGATCAAAGCAGACGCGTCCGACACCCNNNNNNNNNNNNN-------…ACTAGCTCGCGGGAA---------GCGGGACCATTTGCTGCACAG------AACCAG------
ACCAGGCGGCGCAAAATGGCCAATCCCATAAGCAGAGCCAAGCGGATGGATCTCGAGATACGGCGACTGCAGAACCATTCCGGCACACTGATACCCAAGCTGCCGTTCTCGCGTCTGGTGCGCGAGTTCATCGTGAAGTACAGCGATGGAGAGCCGTTGAGGGTCTCCGAGGGCGCCTTTATGGCCATGCAGGAGTCCTGCGAGATGTACGTGACGCAGCGGCTGTCGGACTCCTACATGCTAACCCGGCATCGCAATCGCGTGACACTGGAGGTGCGCGACATGGCTCTGATGGCCTACCTTTG
CGACCGGGGT------CGGCTGGCC---------
>lucipennis
-------…TACGGCTTGGAGTTCACCACCAGCCGCCTGACGCTGCAAGAGTCC---TCCAATCGCCGCTGCTCCACGATGCGCAAAGACNNN---NNNGGCAGAAGATCACGT---------------NNNCCGGCTACGGCATCGTCGAGC---GACGAG---GAGGAT------
CAGGAAAACCACCAACCGGCAGCGAGATCGCCGCAGACCCGTCGAATGACAAGTCTTCAGGGGAGTNNNNNNNNNNNNN---------------------------NNNNNNNNN---------------------------GCCAGGACCCAGGCCNNN------------NNNTCAGGTNNN---NNNNNNNNNNNNNNNNNNNNCAGCAGNNNNNNN------CCCCGCCGCCGCAAGGCCGTCAATCCCATGNNN---------NNNATGGAGCGCGAAATCAGGCGGCTACAGGCCCATCCCGGC-------…
-------…
>lutescens
ATGCCGAGAAAAAGTGGGGCAAACCGCAAGGTGAACNNNCCC------------------AAGCCAACGNNNGGGGACACGGACGCGGGG------------------AGCGACGACGACACGGCCTTCCGATCGCCCGAACCGGAAGATGGCACCGACTACGGCTTGGAGTTCACCACAAGCCGGCTGACTCTGCAGGACTCC---TCCAATCGCCGTTGCTCCACGCTGCGCAAGGACACCAGAAATGACAGG------------------------NNNGCGGATGGAACCGCCTCCAGC---GAAGAG---GAGGAT------
CAGGAGAATCGCCTGCCAGCAGCCAGATCGCCGCAGACGGGACGGATGACCAGTCAGCAGNNNAGGNNNNNNNNNNNNNNNAGCCAGCAAGAGAGGCGTCCTGCTCCCNNNNNNNNNNNNNNNAGGCGTCCGGCTCCCAGCCAGCCGGTTAGGACTCAAGCATCC------------NNN------ACC---GGACCAGCTGCTGGCCAG------GATCGG------
CACCGCCGACGCAAGATGGCCAACCCCATAAGCAGAGCCAAGCGAATGGATCGCGAGATCCAAAATCTGCAGAAACATCCCGGCACGCTTATACCCAAGCTGCCGTTCTCGCGTCTGGTTCGCGAATTCATCGTCAAGTACAGCGACGGAGAACCGTTTAGGGTCACCGAAGGCGCCTTCAACGCCCTGCAGGTGTCCAGCGAGATGTACGTGACGCAGCGCCTGGCCGACTCCTACATGCTGACCAAGCACCGCAATCGCGTCACGCTGGAGGTGCGCGACATGGCTCTGATGGCTTTGATCTGC
GACCGGGGTCACAAC------------------
>mauritiana
ATGCCACGACACAGTAGAGCCAAACGC---------GCACCC------------------AGGCCGTCGGCG------------------AACAACTCAAAGTCGCCGAACGACGACGACACGGCCTTCCACTCGCCGGAGCCAGAAGACGGCACCGACTACGGCCTCGAGTTCACCACCAGCCAACTGACGCTTCAGNNN------AGCAATCGGCGTTCCTCGACGATGCGCAGGGACGCC---------------------GGACGGAGGCAGTCGCCAACGAATGGTAGCTCCGCCAGTGGCNNNNNN---NNNGAC------
CAGGAGAATCGCCATCCCAGAGCCAGACCGTCGCAGACGCGTCGATTGACCNNNNNNNNNNNNN-------…AGCAAGACGCGGGCA---------GCGGGGCCAGTTGCTTGCCAA------AACCAG------
ACCAGGCGGCGCAAAGCGGCCAATCCCATGAGCAGAGCCAAGAGGATGGATCGCGAGATCCGGCGACTGCAGCACCATCCCGGCACACTGATACCCAAGCTGCCGTTCTCGCGTCTGGTGCGGGAGTTTATCATGAAGTACAGCGACGGCGAGCCGCTGAGGGTCACCGAGGGCGCCCTATTGGCCATGCAGGAGTCCTGCGAGATGTACTTGACGCAGCGGCTCGCGGACTCCTACATGCTAACCAAGCATCGCAATCGCGTCACACTGGAGGTGCGCGACACGGCATTGATGGCCTACATCT
GCGACCGGGGT------CGGCAAGCT---------
>melanogaster
ATGCCACGACACAGCAGAGCCAAGCGC---------GCACCC------------------AGGCCGTCGGCG------------------AACAACTCAAAGTCGCCGAACGACGACGACACGGCCTTCCGCTCGCCGGAGCCAGAAGACGGCACCGACTACGGCCTCGAATTCACCACCAGCCAACTGACGCTTCAGNNN------AACAATCGGCGTTCCTCGACGTTGCGCAGGGACGCC---------------------GGACGGAGGCAGCCGGCAGCGAGAGACAGCTCCACCAGTGGCNNNNNN---NNNGAC------
CAGGAGAATCGCTATCCCACAACCAGATCGCCGCAGACGCGTCGAATGACCNNNNNNNNNNNNN-------…AGCAAAACGCGAGCA---------GCAGGGCCAGTTGCTGCGCAA------AACCAG------
ACCAGGCGGCGCAAAGCGGCCAATCCCATGAGCAGAGCCAAGAGAATGGATCGTGAGATCCGGCGACTGCAGCACCATCCCGGCACACTGATACCCAAGCTGCCGTTCTCGCGTCTAGTGCGCGAGTTCATCGTGAAGTACAGCGACGACGAGCCGCTAAGGGTCACCGAAGGCGCCCTATTGGCCATGCAGGAGTCGTGCGAGATGTACTTGACGCAGCGGCTCGCCGACTCCTACATGCTAACCAAGCATCGCAATCGCGTCACACTGGAGGTGCGCGACATGGCATTGATGGCCTACATCT
GCGACCGGGGT------CGGCAATTT---------
>mimetica
ATGCCGAGAAAAAGTGGCGCAAGCCGCAAGATGCCCNNNCCC------------------AAGCCAACGNNNGGGGACACGGACGCGGAG------------------AGCGACGACGACACGGCTTTCCGATCGCCGGAACCGGAAGATGGCACCGACTACGGCCTGGAGTTCACCACAAGCCGACTGACTCTACAGGATGCC---TCCAATCGCCGTTGCTCAACGATGCGCAAGGACACCCGAAACACCCGGGCATCGCGA---------------NNNGCGGATAGAACCGCCTCAAGC---GAAGAG---GAGGAT------
CAGGAGAATCGCCTTCCTGCAGCCAGATCGCCGCAGACGCGACAGATGACCAGTCAGCAGGAGAGGNNNNNNNNNNNNN---------------------------NNNNNNNNN---------------------------GTCAGGGTCCAAGCATCT------------NNN------GCC---GGACAA---NNNGTCCAG------AATCAA------
ACCAGGCGACGTAAGATGGCCAATCCCATGAGCCGAGCCCAGAGAATGGATCGCGAGATCCGACATCTGCAGAATCATCCCGGCATACTCATCCCTAAGCTGCCATTCTCGCGCCTGGTTCGCGAATTTATCATGAAGTACAGCGACGGAGCTCCGTTGAAGGTCACTGAAGGCGCCCTCAACGCCATGCAGGTGTCCAGCGAGTTGTATGTGACGCAACGTCTGGCCGACTTCTACATGCTAACCAAACACCGCAATCGCGTCACGCTGGAAGTGCGCGACATGGCTTTGATGGCTCTGATCTGTG
ACCGGAGTCATAAC------------------
>orena
ATGCCTCGACACAGTGATGCCAAGCGT---------AACCCTAAATCGAACCCCAAATCGNNNNNNNAAAATG------------------AACAACTCACAGCCGCCAAGCGACGACGACACAGCATTCCGCTCGCCGGCTCCAGAAGGCGACACCGACTACGGCCTGGAGTTCACCACTAGCGAACTGACGCTACAGGAGGGC---AACAATCGTCGTTGCTCCACGATGCGCAAGGACGCC---------------------GGACATAGGCATCAGCCAGCGACCCGCACCTACTCCNNNNNNNNNNNNN---NNNAAC------
CAGGAGAATCGCCATCGGCAGCCACATCCAAGCAGACGCATCCGAAA-------…
CGGCGGCGCAAAATGAACAATCCCATAAGCAGAGTCAAGAGGGTGAACCTCGAGATACGGCGATTGCAAAGCCATTCCCGCGCACTGATACCTAAGCTGCCCTTCTCGCGTCTGGTGCGCGAATTCATCATGAAGTACAGCGATGGGGAGCCGTTGAAGATCAGCGTGGCAGCCTTAGTGGCCATACAGGAGTCCTGCGAGCTGTACGTGACACAGCGGCTGTCGGACTCCTACATGCTAACCAGGCATCGCAATCGCGTCACACTTGAGGTGCGCGACATGGCTCTGATGGCCTACATCTGCGA
CGGGGCT------CGGCTAGCC---------
>paralutea
-------…TACGGCTTGGAGTTCACCACAAGCCGGCTGACTCTGCAGGACGCC---TCCAATCGCCGTTGCTCAACGCTGCGCAAGGACACCAGAAATGGCCGGGCATTGGAA---------------NNNGCGGATAGAACCGCCTCCAGC---GAAGAG---GAGGAT------
CAGGAGAATCGCCTGCCAGCAGCCAGATCGCCGCAGACGCGACGGATGACCAGCCAGCAGGAGAGGCGTCCGGCTCCC---------------------------AGCCAGCAGGAGAGGCGTCCGGCTCCCATCCAGCCGGTTAGGGTTCAAGCATCC------------NNN------ACC---GGACCAGCGGCTGGCCAG------GATCGG------
AAACGGCGACGCAAGATGGCCAATCCCATAAGCAGAGCCAAGCGAATGGATCGCGAGATCCAACGTCTGCAGAAACATCCCGGCACGCTTATACCCAAGCTGCCATTCTCGCGTCTGGTTCGCGAATTTATAGTCAAGTACAGCGATGGACCACCGTTCAGGGTCACCGAAGGCGCCTTCAACGCCATGCATGTGTCCAGCGAGATGTACGTGACGCAGCGTCTGGCCGACTCCTATATGCTGACCAAACACCGCAATCGCGTCACGCTGGAGGTGCGCGACATGGCTCTGATGGCTTTGATCTGC
GACCGGGGTCATAAC------------------
>prostipennis
ATGCCGAGAAAAAGTGGCGCAAGCCGCAGGGTGAACNNNCCC------------------AAGCCAACGNNNGGGGACACGGACGCGGAG------------------AGCGACGACGACACGGCCTTCCGATCGCCAGAACCGGAAGATGGCACCGACTACGGCTTGGAGTTCACCACAAGCCGGCTGACTCTGCAGGACGCC---TCCAATCGCCGTTGCTCGACTCTGCGCAAGGACACTAGAAATGGCCGGGCATTGGGA---------------NNNGCCGACAGAACCGCCTCCAGC---GAAGAG---GAGGAC------
CAGGAGAATCGCCTGCCAGCAGCCAGATCGCTGCAGTCGAGTCGCATAACCAGTCAGCAGGAGATGCGTCCTGCTCCC---------------------------AGCCAGCAG---------------------------GTCAGGACTCAGGCATCT------------NNN------TCA---GGACAAGCTGCTCCTCAG------GATCGG------AACCGGCGACGCAAGATGGCCAATCCCATAAGCAGAGCCCAGCGAATGGATCGCGAGATCCAAAATCTGCAGAAACATCCCGGC-------…
-------…
>rajasekari
-------…GACTACGGCTTGGAGTTCACCACAAGCCGGCTCACCCTG---------AATTCCAACCGCCGATGCTCGACGCTGCACAAGGAGACCTCAGGCGGCCCTGGGTCGCGA---------------NNNGCGGGAAGAACT---NNNAGC---GATGAG---GAGGAT------CAGGAGAATCGG---
NNNNNNGCCAGCTCGCCGGGGACGCGCAGGATGAGCGGTCAGCAGGAAAGGNNNNNNNNNNNNN---------------------------NNNNNNNNN---------------------------TCCAGGACTCAGGCAGCG------------NNN------AAC---GGATCGATTGGTGCCCAG------AATCAG------ACCAGGAGACGCAAGATGGCCAAACCCTTGAGCAGAGCCAGAAGGATGGATCTAGAGATCCAGCATCTGCAGAATCATCCCGGC-------…
-------…
>sechellia
ATGCCACGACACAGTAGAGTCAAGCGC---------GCACCC------------------AGGCCGTCGGCG------------------AACAACTCAAAGTCGCCGAACGACGATGACACAGCCTTCCGCTCGCCGGAGCCAGAAGACGGCACCGACTACGGCCTCGAGTTCACCACCAGCCAACTGACGCTTCAGNNN------AACAATCGGCGTTCCTCGACGATGCGCAGGGACGCC---------------------GGACGGAGGCAGTCGCCAACGAATGGTAGCTCCGCCAGTGGCNNNNNN---NNNGAC------
CAGGAGAATCGCCATCCCAGAGCCAGACCGTCGCAGACGCGTCGATTGACCNNNNNNNNNNNNN-------…AGCAAGACGCGGGCA---------GCAGGGCCAGTTGCTGCGCAG------AACCAG------
ACCAGGCGGCGCAAAGCGGCCAATCCCATGAGCAGAGCCAAGAGGATGGATCGCGAGATCCGGCGACTGCAGCACCATCCCGGCACACTGATACCCAAGCTGCCGTTCTCGCGTCTGGTGCGGGAGTTTATCATGAAGTACAGCGACGGCGAGCCGCTGAGGGTCACCGAGGGCGCCCTAATGGCCATGCAAGAGTCCTGCGAAATGTACTTGACGCAGCGGCTCGCGGACTCCTACATGCTAACCAAGCATCGCAATCGCGTCACACTGGAGGTGCGCGACATGGCATTGATGGCCTACATCT
GCGACCGGGGT------CGGCAAGCT---------
>simulans
ATGCCACGACACAGTAGAGCCAAGCGC---------GCACCC------------------AGGCCGTCGGCG------------------AACAACTCAAAGTCGCCGAACGACGATGACACAGCCTTCCACTCGCCGGAGCCAGAAGACGGCACCGACTACGGCCTCGAGTTCACCACCAGCCAACTGACGCTTCAGNNN------AACAATCGGCGTTCCTCGACGATGCGCAGGGACGCC---------------------GGACGGAGGCAGTCGCCAACGAATGGTAGCTCCGACAGTGGCNNNNNN---NNNGAC------
CAGGAGAATCGCCATCCCAGAGCCAGACCGTCGCAGACGCGTCGATTGACCNNNNNNNNNNNNN-------…AGCAAGACGCGGGCA---------GCAGGGCCAGTTGCTGCGCAG------AACCAG------
ACCAGGCGGCGCAAAGCGGCCAATCCCATGAGCAGAGCCAAGAGGATGGATCGCGAGATCCGGCGACTGCAGCACCATCCCGGCACACTGATACCCAAGCTGCCGTTCTCGCGTCTGGTGCGGGAGTTTATCATGAAGTACAGCGACGGCGAGCCGCTGAGGGTCACCGAGGGCGCCCTGTTGGCCATGCAAGAGTCCTGCGAAATGTACTTGACGCAGCGGCTCGCGGACTCCTACATGCTAACCAAGCATCGCAATCGCGTCACACTGGAGGTGCGCGACATGGCATTGATGGCCTACATCT
GCGACCGGGGT------CGGCAAGCT---------
>takahashii
ATGCCGAGAAAAAGTGGCGCAAGCCGCAAGGTGGACNNNCCC------------------AAGCCGACGNNNGGTGACACGGACGCGGAG------------------AGCGACGACGACTCGGCCTTCCGATCGCCGGAACCGGAGGACGGCACCGACTACGGCTTGGAGTTCACCACCAGCCGGCTGACTCTGCAGGACGCC---TCCAATCGCCGTTGCTCGACGCTGCGCAAGGACGCCAGAAGTGGCCGGGCATTGGAA---------------NNNGCGAATAGAACAGCCTCCAGC---GAGGAG---GAGGAT------
CAGGAGAATCGACTGCCAGCAGCCAGATCGCCG------CGACGGATGACCAGTCAGCAGGAAAGGCGTCCAACTGCC---------------------------AGCCAGCAGGAGAGGCGTCCAACTGCCAAC---------------CAAGCATCT------------NNN------TTA---GGACCAGCTGCTCCTCAG------GATCGT------
AGGCGGCCACGCAAGATGGCCAATCCCATGAGCAGAGCCCGGCGAATGGATCGCGAGATCCAACAACTGCAGAAACATCCCGGCACGCTTATACCCAAGCTGCCATTCTCGCGCTTGGTTCGCGAATGTATCATGAAGTACAGCGACGGAGCACCGCTGAAGATCACCGAGGGCGCCTTCAACGCCCTGCAGGTGTCCAGCGAGATGTACGTGACGCAGCGCCTGGCCGACTCCTACATGCTGACCAAGCACCGCAATCGCGTCACGCTGGAGGTGCGCGACATGGCTCTGATGGCTCTGATCTG
CGACCGGGGTCACAAC------------------
>teissieri
ATGCCTCGACACAGTAACGCCAAGCGC---------GGCCCC------------------AAATCGACGGTG------------------AACAACTCAAAACCGCCAAGCGACGACGACACAGCCTTCCGCTCGCCGGAGCCAGAAGACGGCACCGACTATGGCCTGGAGTTCACCACCAGCCAACTGACGCTACAGGAG------AACAGTCGTCGTTGCTCGACGATGCGCAAGGACGCC---------------------GGACGGCGGCATCCGGCGGCGAGTAGGATGTCCACCAGCGACNNNNNN---
NNNNNNCAGGATNNNGAAAATCGCCATCCGGCAGCCAGATCACCGCAGACGCGTCGGATGACCNNNNNNNNNNNNN-------…ACCAGCACGCGGGCG---------CCGAGACCAGTTGCTGCACAG------
AACNNNAACCAAAACAGGCGGCGCAAAATGCCCAATCCCATAAGCAGAACGAAAAGAATGGATCGCGAGATACGGCGACTGCAGAACCATCCCGGCACACTAATACCCAAGCTGCCGTTCTCGCGTCTGGTGCGCGAGTTCATCGTGAAGTACAGCGATGGAGAACCGCTGAGGGTCACCGAGGGCGCCTTTATGGCCATGCAGGAGTCCTGCGAGATGTACCTGACGCAGCGGCTCGCGGACTCCTACATGCTAACCAAGCATCGCAATCGCGTTACACTGGAGGTGCGCGACATGGCCCTGA
TGGCCTACATCTGCGACCGGGGT------CGGCTAGCA---------
>trilutea
-------…GAAGAG---GAGGAT------CAGGAGAATCGCCTGCCACCAGCCAGATCGCCGCAGACGCGACGGATGACCAGTCAGCAGGAGAGGCGACCGGCTCCC---------------------------
AGCCAGCAGGAGAGGCGTCCGGCTTCGAGCCAGCCGGTTAGCAACCAAGCATCC------------NNN------ACC---GGACCAGCTGCTGGCCAG------GATCGG------
AAACGGCCACGCAAGATGGCCAATCCCAGAAGCAGAGCCCAGCGAATGGATCGCGAGATCCAACGTCTGCAGAAACATCCCGGCACGCTTATACCCAAGTTGCCATTCTCGCGTTTGGTTCGCGAATTTATCGTCAAGTACAGCGACGGAGAACCGTTTAGGGTCACCGAAGGCGCCTTCAACGCCATGCAGGTGTCCAGCGAGATGTACGTGACTCAGCGTCTGGCCGACTCCTACATGCTGACCAAACACCGCAATCGCGTCACGCTGGAGGTGCGCGACATGGCTCTGATGGCTTTGATCTGC
GACCGGGGTCATAAC------------------
>yakuba
ATGCCACGACACGGAACGCCAAGCGC---------GGCCCC------------------AAATCGACGGTG------------------AACAATTCAAGGCCGCAAACCGACGACGACACATGCTTCCGCTCGCCGGAACCAGAAGACGGCACCGACTACGGCCTGGAATTTACCACCAGCCAACTGACGCTACGGGAGGAC---AACAGTCGTCGTTCCTCGACGATGCGCAAGGACGCC---------------------GAACGGAGGCATCCGCCAGCGAATAGGACCTCCACCAGCGACNNNNNN---
NNNNNNGAGGATNNNGAAAATCGCGGTCCGGCAGCCGCGTCGTCGCAGTCGCGTCGGATGACCNNNNNNNNNNNNN-------…ACCAGCACGCGGTCG---------CCGAGACCAGTTGCCGCACAG------
AACNNNAACCAAACCAGGCAACGCAAAATGCCCAATCCCATAAGCAGGACCAATAGGTTGAATCGCGAGATACTGCGGCTGCAGAACCATCCCGGCACACTTATACCCAAGCTGACGTTCGCGCGTCTGGTGCGCGAGTTCATCGTGAAGTACAGCGATGAAGCACCGTTGAGGGTCACCGAGGGCGCGTTAATTGCCATGCAGGAGTCCTGCGAGATGTTCTTGACGCAGCGGCTCGAGGACTCCTACATGCTAACCAAGCATCGCAATCGCGTCACACTGGAGGTGCGTGACATGGCCCTGAT
GGCCTACATCTGCGACCGANNN------NNN------TCGTCAAAT

Primates – CenH3 alignment inferred in Bali-Phy. All the codons with reliability score below 80% are masked with NNN.

```
>Aotus
ATGGGCCCGCGCCGCCGGAGCCGCAAGCCTGAGGCCCCGAGGAGGCGCCGCGCGAGTCCG------
ACCCCAGCCCCCCTCGGCGGGGCCCCTCCTTAGGCGCTTCCTCACGTCCACGTGGTTATCGAAGGCAAGGTTGGCTAAAGGAGATCCGAAAGCTTCAGAAGAGCACACACCTCTTGTTAAGGAAGTACCCCTTCAGCCGCCTGGCAAGAGAAATATGTGTTAAATTCACTCGTGGTGTGGACTTCAATTGGCAAGCCCAGGCCCTATTGGCCCTACAAGAGGCGGCAGAAGCATTTCTAGTTCATCTCTTTGAGGATGCCTATCTCCTCACCTTACATGCCGGCCGAGTTACTCTCTTCCCAAAGGAC
GTGCAACTGGCCCGGAGGATCCGGGGCATTGAGGAGGGACTCGGC
>Callithrix
ATGGGCCCGCGCCGCCGGAGCCGCAAGCCCGAGGCCCCGAGGAGGCGCAGCGCGAGCCCG------
ACCCCCAGCCCCTCCCGGCGGGGCCCCTCCTTAGGTGCTTCCTCACGTCCACGTGGTTATCGAAGGCAAGGTTGGCTAAAGGAGATCCGAAAGCTTCAGAAGAGCACACACCTCTTGTTAAGGAAGTACCCCTTCAGCCGCCTGGCAAGAGAAATAAGTGTTAAATTCACTCGTGGTGTGGACTTCAATTGGCAAGCCCAGGCCCTATTGGCCCTACAAGAGGCTGCAGAAGCATTTCTAGTTCATCTCTTTGAGGATGCCTATCTCCTCACCTTACATGCCGGCCGAGTTACTCTCTTCCCAAAGGAT
GTGCAACTGGCCCGGAGGATCCGGGGCATTGAGGGAGGACTCGGC
>Colobus
ATGGGCCCGCGCCGCCGGATTCGCAAGCCCGAGGCCCCGAGGAGGCGCAGCCCGAGCCCG------
ACCCCCGGCCCCTCCCGGCGGCGCCCCGCCTTAGGCCCTTCCTCCCGTCAACATGGTCGGCGGAGACAAGCTTGGCTAAAGGAGATCCGAAAGCTTCAGAAGAGCACACACCTCTTGATAAGGAAGTACCCCTTCAGCCGCCTGGCAAGAGAAATATGTGTTCAATTCACTCGTGGTGTGGACTTCAATTGGCAAGCCCAGGCCCTATTGGCCCTACAAGAGGCAGCAGAAGCATTTCTAGTTCATCTCTTTGAAGATGCCTATCTCCTCACCTTACATGCCGGCCGAGTTACTCTCTTCCCAAAGGAT
GTGCAACTGGCCCGGAGGATCCGGGGCATTGAGGCGGGACTCGGC
>Gorilla
ATGGGCCCGCGCCGCCGGAGCCGAAAGCCCGAGGCCCCGAGGAGGCGCAGCCCGAGCNNNACCCCGNNNCCCGGCCCCTCCCGGCGGGGCCCCTCCTTAGGCGCTTCCTCCCATCAACACAGTCGGCGGAGACAAGGTTGGCTAAAGGAGATCCGAAAGCTTCAGAAGAGCACACACCTCTTGATAAGGAAGCTCCCCTTCAGCCGCCTGGCAAGAGAAATATCTGTTAAATTCACTCGTGGTGTGGACTTCAATTGGCAAGCCCAGGCCCTATTGGCCCTACAAGAGGCAGCAGAAGCATTTC
TAGTTCATCTCTTTGAGGACGCCTATCTTCTCACCTTACATGCTGGCCGAGTTACTCTCTTCCCAAAGGATGTGCAACTGGCCCGGAGGATCCGGGGCCTTGAGGAGGGACTCGGC
>Human
ATGGGCCCGCGCCGCCGGAGCCGAAAGCCCGAGGCCCCGAGGAGGCGCAGCCCGAGCNNNACCCCGNNNCCCGGCCCCTCCCGGCGGGGCCCCTCCTTAGGCGCTTCCTCCCATCAACACAGTCGGCGGAGACAAGGTTGGCTAAAGGAGATCCGAAAGCTTCAGAAGAGCACACACCTCTTGATAAGGAAGCTGCCCTTCAGCCGCCTGGCAAGAGAAATATGTGTTAAATTCACTCGTGGTGTGGACTTCAATTGGCAAGCCCAGGCCCTATTGGCCCTACAAGAGGCAGCAGAAGCATTTC
TAGTTCATCTCTTTGAGGACGCCTATCTCCTCACCTTACATGCAGGCCGAGTTACTCTCTTCCCAAAGGATGTGCAACTGGCCCGGAGGATCCGGGGCCTTGAGGAGGGACTCGGC
>Chlorocebus
ATGGGCCCGCGCCGCCGGAGCCGCAAGCCCGAGGCCCCGAGGAGGCGCAGCCCGAGCCCG------
ACCCCCGGCCCCTCCCGGCGGGGCCCCTCCTTAGGCCCTTCCTCCCATCAACATGGTCGGCGGAGACAAGCTTGGCTAAAGGAGATCCGAAAGCTTCAGAAGAGCACACACCTCTTGATTAGGAAGTACCCCTTCAGCCGCTTGGCAAGAGAAATATGTGTTAAATTCACTCGTGGTGTGGACTTCAATTGGCAAGCCCAGGCCCTATTGGCCCTACAGGAGGCAGCAGAAGCATTTCTAGTTCATCTCTTTGAAGATGCCTATCTCCTCACCTTACATGCCGGCCGAGTTACTCTCTTCCCAAAGGAT
GTGCAACTGGCCCGGAGGATCCGGGGCATTGAGGCGGGACTCGGC
>Lemur
ATGGGCCCGCGCCGCCGGAGGCGCAAGCCCGAGACCCCGACGAGGCGCGTCGAGAGCCCG------
ACCCCCGGCCCCTCGCGGCGGCGGCCCCCCGTAGCCCCTACCCCCCGTCAACATCGTCGGAGGACATTTGGATGGTTACAGGAGATCCGAAAACTTCAGAAGAGCACACACCTCTTATTAAGGAAGGCCCCCTTCAGCCGCGTGGCAAGAGAAGTATGTACTAAATTCACTCGTGGTGTGGACTTCAATTGGCAAGCCCAGGCCCTGTTGGCCCTACAAGAGGCTGCAGAAGCATTTCTAGTCCATCTCTTTGAGGATGCTTATCTCCTCACCCTACATGCCGGCCGAGTTACTCTTTTCCCAAAGGAC
GTGCAGCTGGCCAGGAGGATCCGAGGCATTCAGGAAGGGCTCGGC
>Macaca
ATGGGCCCGCGCCGCCGGAGCCGCAAGCCCGAGGCCCCGAGGAGGCGCAGCCCGAGCCCG------
ACCCCCGGCCCCTCCCGGCGGGGCCCCTCCTTAGGCCCTTCCTCCCGTCAACATGGTCGGCGGAGACAAGCTTGGCTAAAGGAGATCCGAAAGCTTCAGAAGAGCACACACCTCTTGATAAGGAAGTACCCCTTCAGCCGCCTGGCAAGAGAAATATGTGTTAAATTCACTCGTGGTGTGGACTTCAATTGGCAAGCCCAGGCCCTATTGGCCCTACAAGAGGCAGCGGAAGCATTTCTAGTTCATCTCTTTGAAGATGCCTATCTCCTCGCCTTACATGCCGGCCGAGTTACTCTCTTCCCAAAGGAT
GTGCAACTGGCCCGGAGGATCCGGGGCATTGAGGCGGGACTCGGC
>Nomascus
ATGGGCCCGCGCCGCCGGATCCGCAAGCCCGAGGCCCCGAGGAGGCGCAGCCCGAGTCCG------
ACCCCCGGCCCCTCCCGGCGGGGCCCCTCCTTAGGCGCTTCCTCCCGTCAACACGGTTGGCGGAGACAAGGTTGGCTAAAGGAGATTCGAAAGCTTCAGAAGAGCACACACCTCTTGATAAGGAAGCTCCCCTTCAGCCGCCTGGCAAGAGAAATATGTGTTAAATTCACGCGTGGTGTGGACTTCAATTGGCAAGCCCAGGCCCTACTGGCCCTACAAGAGGCAGCAGAAGCATTTCTAGTTCATCTCTTTGAGGATGCCTATCTCCTCACCTTACATGCCGGCCGAGTTACTCTCTTCCCAAAGGA
TGTGCAACTGGCCCGGAGGATCCGGGGCCTTGAGGAGGGACTCGGC
>Otolemur
ATGGGCCCGNNN---CGGAGACGCAAGCCCGAAACCCCGACGAGGCGCCCCGCGAGCCCG------
AGCCCCGGCCCCTCGCGGCGGGGCCCTTCCCTAGGCACTTCTTCCCGAGGACATGGTCAGAGGAGACCTTTGTGGTTAAAGGAGATCCGAAAACTTCAGAAGAGCACAAACCTCTTGTTAAGGAAAGCCCCCTTCAGCCGCCTGACAAGAGAAATATGTGGTAAATTCACTCGTGGTGTGGACTACAATTGGCAAGCGCAGGCCCTGTTGGCCCTACAAGAGGCAGCAGAAGCATTTCTAGTTCATCTCTTTGAAGATGCTTACCTTCTCACCTTACATGCTGGCCGAGTTACTCTTTTCCCAAAGGA
TGTGCAACTGGCAAGAAGGATCCGAGGCATTCAGGAAGGGCTGGGC
>Pan
ATGGGCCCGCGCCGCCGGAGCCGCAAGCCCGAGGCCCCGAGGAGGCGCAGCCCGAGCCCG------
ACCCCCGGCCCCTCCCGGCGGGGCCCCTCCTTAGGCGCTTCCTCCCATCAACACAGTCGGCGGAGACAAGGTTGGCTAAAGGAGATCCGAAAGCTTCAGAAGAGCACACACCTCTTGATAAGGAAGCTCCCCTTCAGCCGCCTGGCAAGAGAAATATGTGTTAAATTCACTCGTGGTGTGGACTTCAATTGGCAAGCCCAGGCCTTATTGGCCCTACAAGAGGCAGCAGAAGCATTTCTAGTTCATCTCTTTGAGGACGCCTATCTCCTCACCTTACATGCTGGCCGAGTTACTCTCTTCCCAAAGGAT
GTGCAACTGGCCCGGAGGATCCGAGGCCTTGAGGAGGGACTTGGC
>Papio
ATGGGCCCGCGCCGCCGGAGCCGCAAGCCCGAGGCCCCGAGGAGGCGCAGCCCGAGCCCG------
ACCCCCGGCCCCTCCCGGCGGGGCCCCTCCTTAGGCCCTTCCTCCCGTCAACATGGTCGGCGGAGACAAGCTTGGCTAAAGGAGATCCGAAAGCTTCAGAAGAGCACACACCTCTTGATAAGGAAGTACCCCTTCAGCCGCCTGGCAAGAGAAATATGTGTTAAATTCACTCGTGGTGTGGACTTCAATTGGCAAGCCCAGGCCCTATTGGCCCTACAAGAGGCAGCAGAAGCATTTCTAGTTCATCTCTTTGAAGATGCCTATCTCCTCACCTTACATGCCGGCCGAGTTACTCTCTTCCCAAAGGAT
GTGCAACTGGCCCGGAGGATCCGGGGCATTGAGGCGGGACTCGGC
>Pongo
ATGGGCCCGCGCCGCCGGAGCCGCAAGCCCGAGGCCCCGAGGAGGCGCAGCCCGAGCNNNACCCCGNNNCCCGGCCCCTCCCGGCGGGGCCCCTCCTTAGGCGCTTCCTCCCATCAACACAGTCGGCGGAGACAAGCTTGGCTAAAGGAGATCCGAAAGCTTCAGAAGAGCACACACCTCTTGATAAGGAAGCTCCCCTTCAGCCGCCTGGCAAGAGAAATATGTGTTAAATTCACTCGTGGTGTGGACTTCAATTGGCAAGCCCAGGCCCTATTGGCCCTACAAGAGGCAGCAGAAGCATTTC
TAGTTCATCTCTTTGAGGACGCCTATCTCCTCACCTTACATGCCGGCCGAGTTACTCTCTTCCCAAAGGATGTGCAACTGGCCCGGAGGATCCGGGGCCTTGAGGAGGGACTCGGC
>Saimiri
ATGGGCCCGCGCCGCCGGAGTCGCAAGCCCGAGGCCCCGAGGAGGCGCAGCCCGAGCCCG------ACCCCCGGCCCCTCCCGGCGGGGCCCCNNN---
GGCGCTTCCTCACGTCCACGTGGTTATCGAAGGCAAGGTTGGCTAAAGGAGATCCGAAACCTTCAGAAGAGCACACACCTCTTGATAAGGAAGTACCCCTTCAGCCGCCTGGCCAGAGAAATATGTGTTAAATTCACTCGTGGTGTGGACTTCAGTTGGCAAGCCCAGGCCCTATTGGCCCTACAAGAGGCGGCAGAAGCATTTCTAGTTCATCTCTTTGAGGATGCCTATCTCCTCACCTTACATGCCGGCCGAGTTACTCTCTTCCCAAAGGATGTGCAACTGGCCCGGAGGATCCGGGGCATTGA
GGAGGGACTCGGC
```

Asteraceae – CenH3 alignment inferred in Bali-Phy. All the codons with reliability score below 80% are masked with NNN.

```
>Carthamus_tinctorius
ATGGCGAGAACCAAACAACCTGCTAAACGCAGTTCTGGCAAGCGT---GAT------GCTAGACCATCTACC------TCCACGCCTACGCCAAGGCCAAGTGCAAGGAAGAATCCC---GAGAGCTCAGGAGCTGGGGATGGTCAG------
AGGCGCCATCGTTATAGGCCTGGGACTCAGGCGCTTCGTGAGATTAGGCGCTTGCAGAAGACTGTCAATCTTCTCATTCCGGCTGCTCCTTTCATTCGAACCGTAAAGGAGATAAGCAACTACATTGCGCCAGAAGTCACTCGCTGGCAAGCTGAAGCTCTACAAGCCCTTCAAGAGGCAGCAGAAGATTACTTAATTCAGTTGTTTGAAGATTCAATGCTATGCGCGATTCATGCAAAACGCGTTACCCTCATGAAAAAGGATTGGGAGTTGGCACGGCGGCTTGGGAAGAAAGGGCAACCGTGG
>Cichorium_intybus
ATGGCGAGAACAAAGCAACCTGCTAAACGGAGTTGGGGCAATCGAAAGTCTAGTCAATCTCGAGCATCANNNTCAACTNNNNNNAGTACGCCACGAAAAAGTCCGAGAAAGGATCCA---GGG------
NNNACTGGAGAAAGGNNNCAACAGNNNCCCCATCGTTTCAAGCCTGGGGCTCAAGCACTTCGTGAAATTCGTCGTCTTCAGAAGACTGTTAATCTTCTCATTCCAGCTGCCCCATTCATTCGAACTGTAAAAGAAATAAGCAACTACATTGCCCCTGAAGTTACACGCTGGCAAGCTGAAGCTATACAGGCCCTTCAAGAGGCAGCAGAAGATTACTTAGTTCAGTTGTTTGAAGATTCAATGCTATGTTCGATTCATGCAAAGCGTGTTACCCTCATGAAAAAGGACTGGGAATTGGCACGGAGAC
TTACAAAGAAAGGTCAACCATGG
>Flaveria_vaginata
ATGGCGAGAACCAAACACCCTGCTAAACGAAGTTGGGGCACTAGAAAAGAT------GGTAGAGCATCTACT------TCAACGAGCACTCCGAGGAAAAGCCCTAGGAAGGATGCA---GGGGGTTCTGGAACTGGAGAAAAGCAG------
AGGCCCCATCGGTTTAAGCCTGGGACTCAGGCACTTCGGGAGATTAGGCGTTTGCAAAAGACGGTCAATCTTCTCATTCCTGCTGCTCCCTTCATTCGAACTGTGAGGGAGATAAGCAACTACCTGGCCCCTGAAATCACTCGCTGGCAAGCTGAAGCTTTACAAGCCCTTCAAGAGGCAGCAGAAGATTACATAATTCAGTTGTTTGAAGATTCAATGCTATGCGCAATTCATGCAAAACGTGTTACCCTTATGAAAAAGGATTGGGAGTTGGCACGGCGTCTCGGGAAGAAAGGGCAGCCATGG
>Helianthus_annuus
ATGGCGAGACCCAAACCACCTGCTAAACGCAGTTCAGGCATACCAGCAGAC------GGTAGATCATCCNCT------TCACCAAACACACCAAGAAAGAGTCCGAGGAAGAATCGAGTAGGT------------GGAGAAAACAGG------
AAGCCGCATAGGTTTAAGCCTGGGACACAGGCGCTACGTGAGATTAGGCGTTTGCAGAAGACGGTTGAACTGATCATTCCGGCTGCTCCGTTTATTCGAACTGTAAAGGAGATAAGCAACTACATGGCCCCTGAAATCACTCGCTGGCAAGCCGAAGCTCTACAAGCCCTTCAAGAGGCAGCAGAAGATTACCTAATTCAGTTGTTTGAAGACTCAATGCTATGTGCGATTCATGCAAAGCGCGTTACCCTCATGAAAAAGGATTGGGAGTTGGCACGGCGGATTGGTAAGAAAGGGCAGCCATG
G
>Helianthus_exilis_CHES4473
ATGGCGAGAACCAAACACCCTGCTAAACGCAGTTCAGGCATACCAGTAGAC------GGTAGATCATCCACT------TCAACAAACACACCAAGAAAGAGTCCGAGGAAGAATCGA---GGT------------GGAGAAAACAGG------
AAGCCGCATAGGTTTAAGCCTGGGACACAGGCGCTACGTGAGATTAGGCGTTTGCAGAAGACGGTTGAACTGATCATTCCGGCTGCTCCGTTTATTCGAACTGTAAAGGAGATAAGCAACTACATGGCCCCTGAAATCACTCGCTGGCAAGCCGAAGCTCTACAAGCCCTTCAAGAGGCAGCAGAAGATTACCTAATTCAGTTGTTTGAAGACTCAATGCTATGTGCGATTCATGCAAAGCGCGTTACCCTCATGAAAAAGGATTGGGAGTTGGCACGGCGGATTGGGAAGAAAGGGCAGCCATG
G
>Lactuca_sativa
ATGGCGAGAACCAAACAACCTGCTAAACGAAGTTGGGGCAAGCGACAATCT------GCTGGAGCATCANNNTCAACCNNNNNNAGTACGCCACGGAAATCTCCGAGAAAGGATCCA---
GGGAGTTCAGGAACTGGACAGAGGNNNAAACAGNNNCCCCATCGTTTTAAGCCTGGAACTCAAGCACTTCGTGAGATTCGTCGTCTTCAGAAGACTGTTAACCTTCTCATTCCAGCTGCTCCTTTCATTCGAACTGTAAAAGAGATAAGCAACTACATTGCCCCTGAAGTTACACGTTGGCAAGCTGAAGCTCTACAAGCCCTTCAAGAGGCTGCAGAAGATTACATAGTTCAGTTGTTTGAAGATTCAATGTTGTGCTCGATTCATGCAAAGCGTGTTACCCTGATGAAAAAGGACATGGAATTGGC
TAGAAGGCTTACGAAGAAAGGGCAACCATGG
>Taraxacum_koksaghyz
ATGGCGAGAACCAAACAACCTGCTAAACGGAGTGCGGGCAATCGAACACCT------GCTCAAGCATCANNNTCAACTNNNNNNAGTACGCCGCGAAGAAGTCCGAGAAAGGTTACA---
GGGAGTTCGGGAACGGGAGAAAGGNNNAAACAGNNNCCCCATCGTTTTAAGCCTGGTACTCAGGCACTTCGTGAGATTCGTCGTCTCCAGAAGACCGTTAATCTACTCATTCCAGTCGCTCCATTCGTACGAACTGTAAAGGAAATAAGCAACTACATTGCCCCTGAAGTCACACGCTGGCAAGCTGAAGCTCTACAATGCCTTCAAGAGGCAGCAGAAGATTACTTAATCCGATTGTTTGAAGATTCAATGCTATGTGCGATTCATGCAAAACGTGTTACCCTCATGCAAAAGGATTTGGCATTGG
CACGAAGGCTTACAAAGAAAGGGCAGCCATGG
```

Brassicaceae – CenH3 alignment inferred in Bali-Phy. All the codons with reliability score below 80% are masked with NNN.

>Arabidopsis_arenosa_HTR12
ATGGCGAGAACCAAGCATTTCGCTACCAAGTCACGAACTGGGAATCGAACT------GATNNN---NNNGCT---TCATCTTCTCAG---------GCGACAGGTCCGACTACGACCCCGACAACAAGA---GGC---------ACTGAA-----------GGTGGA--------------------GATAAT---ACTCAACAAACAAATCCT---ACAACT------------TCACCAGCTACT---------GGTGGAAGGNNN---------------CCTAGGAGAGCCAGACAGGCTATGCCGCGAGGTTCA---------CAG---
AAGAAACCTTATCGATTCAAGCCAGGAACCGTTGCTCTGAGAGAGATTCGACATTTCCAGAAGCAGACAAACCTTCTTATTCCAGCTGCTAGCTTCATAAGACAAGTGAGAAGTATAACTCAT------
GCGTTGGCCCCTCCCCAAATCAATCGTTGGACAGCTGAAGCTCTTGTGGCTCTTCAAGAGGCGGCAGAAGATTACTTGGTTGGTTTGTTCTCAGATTCAATGCTCTGTGCTATCCATGCGAGACGTGTTACTCTAATGAGAAAAGACTTTGAGCTTGCACGCCGGCTTGGAGGAAAAGGCAGACCATGG
>Arabidopsis_halleri_subsp._halleri_HTR12B1
ATGGCGAGAACCAAGCATTTCGCTACCAGGACACGATCTGGGAATCGAACT------GATGCC---AATGCT---TCATCTTCTCAG---------GCGGCAGGTCCGACTACGACCCCGACAACAAGA---GGC---------ACTGAA-----------GGTGGA--------------------GATAAT---ACTCAACAAACAAATCCT---ACAACT------------TCACCAGCTACT---------GGTGGAAGGNNN---------------CCTAGGAGAGCCAGACAGACTATGCCACGAGGTTCA---------CAG---
AAAAAGCCTTATCGATACAAGCTAGGAACCGTTGCTCTGAGAGAGATTCGACATTTCCAGAAGCAGACAAACCTTCTTATTCCAGCTGCTAGCTTCATAAGACAAGTGAGAAGTATAACTCAT------
GCGTTGGCCCCTCCCCAAATCAATCGTTGGACAGCTGAAGCTCTTGTGGCTCTTCAAGAGGCGGCAGAAGATTACTTGGTTGGTTTGTTCTCAGATTCAATGCTCTGTGCTATCCATGCAAGACGTGTTACTCTAATGAGAAAAGACTTTGAGCTTGCACGCCGGCTTGGAGGAAAAGGCAGACCATGG
>Arabidopsis_suecica_cenpA
ATGGCGAGAACCAAGCATTTCGTTACCAAGTCACGAACTGGGAATCGAACT------GATGCC---AATGCT---TCATCTTCTCAG---------GCGTCAGATCCGACGACGACCCCGACAACAAGA---GGC---------ACTGAA-----------GGTGGA--------------------GATAAT---ACTCAACAAACAAATCCT---ACAACT------------TCACCAGCTACT---------GGTGGAAGGNNN---------------CCTAGGAGAGCCAGACAGGCTATGCCGCGAGGTTCA---------CAG---
AAGAAGCCTTATCGATACAGGCCAGGAACCGTTGCTCTGAGAGAGATTCGACATTTCCAGAAGCAGACAAACCTTCTTATTCCAGCTGCTAGCTTCATAAGACAAGTGAGAAGTATAACCCAT------
GCGCTGGCCCCTCCCCAAATCAATCGTTGGACAGCTGAAGCTCTTGTGGCTCTTCAAGAGGCGGCAGAAGATTACTTGGTTGGTTTGTTCTCAGATTCAATGCTCTGTGCTATCCATGCAAGACGTGTTACTCTAATGAGAAAAGACTTTGAGCTTGCACGCCGGCTTGGAGGAAAAGGCAGACCATGG
>Arabidopsis_thaliana_HTR12
ATGGCGAGAACCAAGCATCGCGTTACCAGGTCACAACCTCGGAATCAAACT------GATNNNGCCNNNGCT---TCATCTTCTCAG---------GCGGCAGGTCCAACTACGACCCCGACAAGGAGA---GGC---------GGTGAA-----------GGTGGA--------------------GATAAT---ACTCAACAAACAAATCCT---ACAACT------------TCACCAGCTACT---------GGTACAAGGAGA------------GGGGCTAAGAGATCCAGACAGGCTATGCCACGAGGCTCA---------CAG---
AAGAAGTCTTATCGATACAGGCCAGGAACCGTTGCTCTAAAAGAGATTCGCCATTTCCAGAAGCAGACAAACCTTCTTATTCCGGCTGCCAGTTTCATAAGAGAAGTGAGAAGTATAACCCAT------
ATGTTGGCCCCTCCCCAAATCAATCGTTGGACAGCTGAAGCTCTTGTTGCTCTTCAAGAGGCGGCAGAAGATTACTTGGTTGGTTTGTTCTCAGATTCAATGCTCTGTGCTATCCATGCAAGACGTGTTACTCTAATGAGAAAAGACTTTGAACTTGCACGCCGGCTTGGAGGAAAAGGCAGACCATGG
>Arabis_hirsuta_cenpA1
ATGGCGAGAACGAAGCATTTCGCA------AGACGAGGTCAGAATCGAACA------GATGCT---AACGTT---TCCTCTTCGCCG---------GCGGCAGGTCCGAGTACGACTCCGGCGAGAAGG---GCT---------AGTGAA-----------GATGGA--------------------GATGAT---GCTCAACAAACAACTCCT---GCAACT------------TCACCAGCTACT---------GGTAGTAAAAAT---AGTAATGGAGGAGCTAAGAGAACT---------ATGCCACAAAGTTCA---------CAG---
AAGAAGCCTCATCGATACAGGCCAGGAACCGCTGCTCTGAGAGAGATTCGCAAATTCCAGAAGTCCACAAAACTTCTAATTCCTGCTGCTAGTTTTATCAGAGAAGTGAAAGATATAACCTATACTTAT------
GCTCCTCCGCAAATCACTCGTTGGACAGCTGAAGCTCTTGTAGCTATCCAAGAGGCGGCAGAAGATTACTTAGTTGGTTTGTTCTCAGATTCAATGCTTTGTGCTGTTCATGCAAGACGTGTTACTCTAATGAGAAAAGACTTTGAGCTTGCACGCCGGCTTGGAGGAAAAGGCAGACCATGG
>Brassica_carinata_BrCENH36
ATGGCGAGAACCAAACATTTCGCTTCCAGGGCACGAGATCGCAATCGAACT------AATGCG---ACTGCT---TCATCTTCGNNNGCGGCG---NNNGAAGGTCCGAGTGCGACCCCGACGAGAAGA---GAAGGC------NNNCAAGATGAA------NNNNNN------------------------NNN---GCTCAACAGAGT---------GCANNNCCTACTACAACTCCATCA------------GCCGGTAGAAAAAAAGGA---------GGGACTAAGCGAACTAAACAAGCTATGCCTAAAAGTTCCAAC------AAG---
AAGAAGACATTCCGTTACAAGCCTGGAACCGTTGCCCTCAGAGAGATTCGCCATTTCCAGAAGACCACCAAACTCCTTATCCCTGCCGCTAGTTTCATCCGACAAGTGAGAAGTGTCACCCAGATCTTT------
GCCCCTCCCGATGTTACCCGTTGGACTGCTGAAGCTCTTATGGCTATTCAAGAGGCGGCTGAAGATTTTTTAATTGGCTTGTTCTCTGATGCTATGCTTTGCGCTATCCACGCAAGACGTGTTACTCTAATGAGAAAAGATTTTGAGCTTGCACGCCGTCTTGGAGGAAAAGGCAGACCATTG
>Brassica_juncea_BrCENH35
ATGGCGAGAACGAAGCATTACGCATCCAGGGCACGAGATCGCAATCGAAAT------AATGCG---ACTGCT---TCATCTACCNNNGCGGCG---NNNGCAAGTCCGAGTGCGACGCCGACGAGAAGA---GGCNNNAGCAGCNNNCAA-----------GGTNNNCGT---------------------NNN---GCTCAACAG--AGTTCCAACCAGAAAAAG---
AAGAAGTCTTTCCGTTACAAGCCTGGAACCGTTGCTCTCAGAGAGATTCGCCAGTTCCAGAAGACCACCAAACTTCTCATCCCTGCTGCTAGTTTCATCAGAGAAGTGAGAAGTATTACCCATATCCTT------
GCTCCTCCCGAAATTACGCGTTGGACTGCTGAAGCTCTTGTTGCTCTTCAAGAGGCGGCAGAAGATTACTTAGTTGGCTTGTTCTCTGATGCAATGCTCTGCGCTATCCACGCAAGACGTGTTACTCTAATGAGAAAAGATTTTGAGCTTGCACGCCGTCTTGGAGGAAAAGGCAGACCATTG
>Brassica_napus_BrCENH38
ATGGCGAGAACGAAACATTTCGCTTCCAGGGCACGAGATCGCAATCGAACT------AATGCG---ACTGCT---TCATCTTCGNNNGCGGCG---NNNGAAGGTCCGAGTGCGACCCCGACGAGAAGA---GAAGGC------NNNCAA--------------NNN------------------------NNN---GCTCAACAGACA---------GCANNNCCTACTACGACTCCACCA------------GCCGGTAGAAAAAAAGGA---------GGGACTAAGCGAACTAAACAAGCTATGCCTAAAAGTTCCAAC------AAG---
AAGAAGACATTCCGTTACAAGCCTGGAACCGTTGCCCTCAGAGAGATTCGCCATTTCCAGAAGACCACCAAACTTCTTATCCCTGCCGCTAGTTTCATCCGAGAAGTGAGAAGTGTCACCCAGATCTTT------
GCCCCTCCCGATGTTACCCGTTGGACTGCTGAAGCTCTTATGGCTATTCAAGAGGCGGCTGAAGATTTTTTAATTGGCTTGTTCTCTGATGCTATGCTTTGCGCTATCCACGCAAGACGTGTTACTCTAATGAGAAAAGATTTTGAGCTTGCACGCCGTCTTGGAGGAAAAGGCAGACCATTG
>Brassica_nigra_BrCENH32
ATGGCGAGAACGAAGCATTACGCATCCAGGGCACGAGATCGCAATCGAAAT------AATGCG---ACTGCT---TCATCTACCNNNGCGGCG---NNNGCAAGTCCGAGTGCGACGCCGACGAGAAGAGGAGGCNNNAGCAGCNNNCAA-----------GGTNNNCGT---------------------NNN---GCTCAACAG--AGTTCCAACCAGAAAAAG---
AAGAAGTCTTTCCGTTACAAGCCTGGAACCGTTGCCCTCAGAGAGATTCGCCAGTTCCAGAAGACCACCAAACTTCTCATCCCTGCTGCTAGTTTCATCAGAGAAGTGAGAAGTATTACCCATATCCTT------
GCCCCTCCCGAAATTACGCGTTGGACTGCTGAAGCTCTTGTTGCTCTTCAAGAGGCGGCAGAAGATTACTTAGTTGGCTTGTTCTCTGATGCAATGCTCTGCGCTATCCACGCAAGACGTGTTACTCTAATGAGAAAAGATTTTGAGCTTGCACGCCGTCTTGGAGGAAAAGGCAGACCATTG
>Brassica_oleracea_BrCENH33
ATGGCGAGAACCAAACATTTCGCTTCCAGGGCACGAGATCGCAATCGAACT------AATGCG---ACTGCT---TCATCTTCGNNNGCGGCG---NNNGAAGGTCCGAGTGCGACCCCGACGAGAAGA---GAAGGC------NNNCAAGATGAA------NNNNNN------------------------NNN---GCTCAACAGAGT---------GCANNNCCTACTACAACTCCATCA------------GCCGGTAGAAAAAAAGGA---------GGGACTAAGCGAACTAAACAAGCTATGCCTAAAAGTTCCAAC------AAG---
AAGAAGACATTCCGTTACAAGCCTGGAACCGTTGCCCTCAGAGAGATTCGCCATTTCCAGAAGACCACCAAACTCCTTATCCCTGCCGCTAGTTTCATCCGACAAGTGAGAAGTGTCACCCAGATCTTT------
GCCCCTCCCGATGTTACCCGTTGGACTGCTGAAGCTCTTATGGCTATTCAAGAGGCGGCTGAAGATTTTTTAATTGGCTTGTTCTCTGATGCTATGCTTTGCGCTATCCATGCAAGGCGTGTTACTCTAATGAGAAAAGATTTTGAGCTTGCACGCCGTCTTGGAGGAAAAGGCAGACCATTG
>Capsella_bursapastoris_cenpA1
ATGGCGAGAACAAAGCATTTCGCTACGAGGTCCGGACCTCGGACTCCAGCT------GTT---------GCT---TCGTCTTCTCAG---------GCGGCTGTTCCCAGTTCGAGCCCGGCAACAAGA---GGC---------AGAGTA-----------GGTGTA--------------------GACNNNGCTNNNCAACAACCAACTCCT---GCAACT------------TCACCTGCTACT---------GCTAAAAAAAAA------------GGGGCGAAGAGAGCTAGATTTGGTAGGCCACAAGGTTCA---------
CAAAAANNNAAGCCTTACCGTTACAGGCCAGGAACCGTTGCTCTGAGAGAGATTCGCCATTATCAGAAGGGCACTAGCCTTCTTATTCCAGCTGCTGCCTTTATCAGACAAGTGAGAAGTATAACCAAT------
GCAGTGGCCCCTCGGGAAGTAAATCGTTGGACAGCTGAAGCTCTTGTGGCTCTTCAAGAGGCGGCAGAAGATTTCTTGGTTGGTTTGTTCTCTGATTCAATGCTCTGTGCTATCCATGCAAGACGTGTTACTCTAATGAGAAAAGACTTTGACCTTGCACGCCGGCTTGGAGGAAAAGGCAGACCCTGG
>Cardamine_flexuosa_cenpA1
ATGGCGAGAACGAAGCATTTCCCTAACAGGACACGGCCTCGGACTCAAACT------GATGCC---ACTGCT---TCATCTACTCCG---------GCGGCTGGTCCGAGTACGAAGCGGCCAACAAGG---GCC---------AATCAA-----------GGTNNN------------------------NNN---ACTCAACAAACAAATCCT---ACAACT------------TCACCAGCTACC---------AGTAAAAAGAAA------------GGGGCTAAGAGAACTAGACGGGCCATGCCACAAGGTTCA---------CAA---
AAGAAGCCTAATCGTTACAAGCCAGGAACCGTTGCTCTTAGAGAGATTCGCCATTTCCAGAAGAGCACAAATCTTCTGATTCCAGCTGCTAGTTTTATCAGACAAGTGAGAAGTATAACCCAAATGTAT------
GCCCCTCCGCAAATTAATCGTTGGACTGCTGAAGCTCTTGTGGCTCTTCAAGAGGCGGCAGAAGATTACTTGATTGGTTTGTTCTCAGATTCAATGCTCTGTGCTATCCATGCAAGACGTGTTACTCTAATGAGGAAAGACTTTGAGCTTGCACGCCGGCTTGGAGGAAAAGGCAGACCATGG
>Crucihimalaya_himalaica_HTR12
ATGGCGAGAACCAAGCATTTCGCTACTAGGTCACGACCTCGGAATCAAACT------GATGCC---ACTGCT---TCAGCTTCTCAG---------GCGACAGGTCCGAGTACGAACCCGACAACAAGA---GGT---------AGTGAA-----------GGTGAA--------------------GATGCT---GCTCGAGGAACAAATCCT---ACAACT------------TCACCAGCTACT---------GGTAGAAAAAAA------------GGGGTTAAGAGGGCCAGACATGCTATGCCACAAGGTTCA---------CAG---
AAGAAGCCTTATCGTTACAAGGCAGGAACAGTCGCTCTGAGAGAGATTCGCCATTTTCAGAAGAACACAAACCTTCTTATTCCAGCTGCTAGTTTTATAAGACAAGTGAAAAGTATAACCTAT------
GCGGTGGCCCCTCCGCAAATCACTCGTTGGACAGCTGAAGCTCTTGTGGCTCTTCAAGAGGCGGCAGAAGATTACTTGGTTGGTTTGTTCTCAGATTCAATGCTCTGTGCTATCCATGCAAGACGAGTTACTCTAATGAGAAAAGACTTTGAGCTTGCACGCCGGCTTGGAGGAAAAGGTAGACCCTGG
>Crucihimalaya_wallichii_cenpA
ATGGCGAGAACCAAGCATTTCGCTACTAGGTCACGACCTCGGAATCAAACT------GATGCC---ACTGCT---TCAGCTTCTCAG---------GCGACAGGTCCGAGTACGAACCCGACAACAAGA---GGT---------AGTGAA-----------GGTGAA--------------------GATGCT---GCTCGAGAAACAAATCCT---ACAACT------------TCACCAGCTACT---------GGTAGAAAAAAA------------GGGGTTAAGAGGGCCAGACATGCTATGCCACAAGGTTCA---------CAG---
AAGAAGCCTTATCGTTACAAGGCAGGAACAGTCGCTCTGAGAGAGATTCGCCATTTTCAGAAGAACACAAACCTTCTTATTCCAGCTGCTAGTTTTATAAGACAAGTGAAAAGTATAACCTAT------
GCGGTGGCCCCTCCGCAAATCACTCGTTGGACAGCTGAAGCTCTTGTGGCTCTTCAAGAGGCGGCAGAAGATTACTTGGTTGGTTTGTTCTCAGATTCAATGCTCTGTGCTATCCATGCCAGACGAGTTACTCTAATGAGAAAAGACTTTGAGCTTGCACGCCGGCTTGGAGGAAAAGGTAGACCCTGG
>Eruca_sativa_cenpA
ATGGCGAGAACGAAGCATTTTGCATCCAGGGCACGAGATCGCAATCGAAAT------AATGCG---ACAGCT---TCATCTTCCNNNNNNNNNNGCGNNNNNNGGTCCGAGTGCGACACCGACGAGAAGA---GGCAGC------NNNCAA-----------GGTNNN---GGTGGTGGTGGTGGTGTT---NNN---GCTCAACAG--GGTTCCAAC---NNNNNN---
NNNAAGTCTTTCCGTTACAAGCCTGGAACCGTTGCTCTCAGAGAGATTCGCCATTTCCAAAAGACCACCAAACTCCTTATCCCTGCTGCTACTTTCATCCGACTAGTCAGAAGCATCACCNNNNNNNNNN------
NNNNNNCCCCAAGTTACGCGTTGGACTGCTGAAGCTCTTGTTGCTCTTCAAGAGGCGGCAGAAGATTACTTAGTTGGCTTGTTCTCTGATTCAATGCTCTGCGCTATCCATGCTAAACGTGTTACTCTTATGAGGAAAGATTTTGAGCTTGCACGCCGCCTTGGAGGTAAAGGCAGACCATGG
>gArabidopsis_lyrata_HTR12
ATGGCGAGAACCAAGCATTTCGCTACCAAGTCACGATCTGGGAATCGAACT------GATGCC---AATGCT---TCATCTTCTCAG---------GCGGCAGGTCCGACGACGACCCCGACAACAAGA---GGC---------ACTGAA-----------GGTGGA--------------------GATAAT---ACTCAACAAACAAATCCT---ACAACT------------TCACCAGCTACT---------GGTGGAAGGNNN---------------CCTAGGAGAGCCAGACAGGCTATGCCGCGAGTTTCA---------CAG---
AATAAGCCTTATCGATACAAGCCAGGAACCGTTGCTCTGAGAGAGATTCGACATTTCCAGAAGCAGACAAACCTTCTTATTCCAGCTGCTAGCTTCATAAGACAAGTGAGAAGTATAACTCAT------
GCGTTGGCCCCTCCCCAAATCAATCGTTGGACAGCTGAAGCTCTTGTGGCTCTTCAAGAGGCTGCAGAAGATTACTTGGTTGGATTGTTCTCAGATTCAATGCTCTGTGCTATCCATGCAAGACGTGTTACTCTAATGAGAAAAGACTTTGAGCTTGCACGCCGGCTTGGAGGAAAAGGCAGACCATGG
>Lepidium_virginicum_cenpA1
ATGACGAGAACCAAGCATTACGTTTCGAGGCCACAACCTNNNNNNN------CGCAAGGATGCC---ACTGTTTCTTCATCTTCTCCG---------GCGGCTGGTACAAGTACGAATCGAACAACAAGG---AGT---------TCTNNN------GGTGAANNNGGA--------------------GATAAT---GCTCAACAAACAATTCCT---ACAACT------------TCACCGGCTACT---------AATAGCAAAAAA------------GGGGCAAAAAGAACCAAAGAAACTATGCCACAAAGTTCA---------AAT---
AAGAAAACATATCGATACAAGCCAGGCACCGTTGCTCTGAGAGAGATTCGCCATTTTCAGAAGAGCACAAATCTTCTAATTCCAGCTGCTAGTTTCATAAGAGAAGTGAGATGTATTACTCAG------
GCTGTGGCCCCTCCGCAAATCAATCGTTGGACAGCTGAAGCTCTTGTGGCTCTTCAAGAGGCGGCAGAAGACCACGTGGTTGGCTTGTTCTCTGATTCGATGCTTTGTGCTATCCATGCAAGACGAGTTACACTTATGAAAAAAGATTTTGAGCTTGCACGCCGGCTTGGAGGAAAAGGCAGACCATGG
>Olimarabidopsis_pumila_cenpA1
ATGGCGAGAACCAAGCATAACGCTATCAGGTCACGA------GATCGAACT------GGTGCA---ACTGCT---TCATCATCTCAG---------GCAGCAGGTCCGAGTACGAACCCCACAGCAGGA---GGT---------AGTNNN--------------------------------------GATGCT---GCTCAACAAACAACTCCT---ACGACT------------TCACCAGCTACT---------GGTAGTAAAAAA------------CGGGCTAAGAGAGCCAGACAGGCTATGCCACGAGGTTCA---------CAG---
AAGAAGCCTTATCGTTACAAGCCAGGAACTGTTGCTCTGAGAGAGATTCGCCATTTTCAGAAGACCACTAGCCTTCTTCTTCCAGCTGCTCCTTTTATAAGACAAGTGAGAAGTATAAGCAGT------
GCTTTGGCACCTAGGGAAATCACTCGTTGGACAGCTGAAGCTCTTGTGGCTCTCCAAGAGGCGGCAGAAGATTACTTGGTTGGTTTGTTCTCAGATTCCATGCTCTGTGCTATCCATGCAAAACGTGTTACTCTGATGAGAAAAGACTTTGAGCTTGCACGCCGGCTCGGAGGAAAAGGCAGACCATGG
>Raphanus_sativus_cenpA1
ATGGCGAGAACGAAGCATTTCGCTTCCAGAGCACGAGATCGCAATCAACCT------AATGCG---------------------NNNGCGGCG---NNNGCAGGTCCGAGTGCGACACCGACGAGAAGA---GGCAGC------NNNCAA-----------GGTNNN------------------------NNN---GCTCAACAGACAACGCCTACCACAACT------------TCACCAGCAACAACCGCCTCCGGTAGAAAAAAA------------GGGACTAAGCGAACTACACAAGCTATGCCTAAAAGTTCC---------AAG---
AAGAAGACTTTCCGTTACAAGCCTGGAACTGTTGCCCTCAGAGAGATTCGCCATTTCCAGAAGTCCACCAAACTTCTCATCCCTTCTGCTCCTTTCATCCGAGAAGTCAGAAGTATCACCCACAACCTT------
GCCGCTGCCTATGTTACGCGTTGGACTGCTGAAGCTCTTATTGCTCTTCAAGAGGCGGCAGAAGATTTCTTAGTTGGCTTGTTCTCTGATGCAATGCTTTGCGCTATCCATGCAAAACGTGTTACTCTAATGAGAAAAGATTTTGAGCTTGCACGCCGGCTTGGAGGAAAAGGCAGACCCTTT
>Turritis_glabra
ATGGCGAGAACCAAGCATTTCGCTACCAGGTCACGACCTCGGAATCAAACT------GAT---------------TCATCTTCTCAG---------GCGGCAGGTCCGAGTACGAACCCAACAACAGGA---GGT---------AGTGAA-----------GGTGGA--------------------GATGCT---GCTCAACAAACAACTCCT---ACGACT------------TCACCAGCTACT---------GGTAGAAAAAAA------------CGGGCTAAGAGAGCCAAACAGGCTATGCCACAAGGTTCA---------CAG---
AAGAAGCCTTATCGTTACAAGCCAGGAACCATTGCTCTGAGAGAGATTCGCTATTTTCAGAAGAACACAAACCTTCTTATTCCAGCTGCTAGTTTTATAAGAGAAGTGAGAAGTATAACCCAT------
GCGCTGGCCCCTCCGCAAATCTCTCGTTGGACAGCTGAAGCTCTTGTTGCTCTCCAAGAGGCGGCAGAAGATTACTTGGTTGGTTTGTTCTCAGATTCCATGCTCTGTGCAATCCATGCAAGACGTGTTACTCTGATGAGAAAAGACTTTGAGCTTGCACGCCGGATTGGAGGAAAAGGCAGACCATGG

Bryophyta – CenH3 alignment inferred in Bali-Phy. All the codons with reliability score below 80% are masked with NNN.

```
>Anomodon_attenuatus
ATGGCGAGGCGGAAGTCGACCCCTCTGCACGGCAACAAACGCGCACCCACCCGC---CCG---------NNNGCT------GCTGCTGCTGCTGCCTCGACTTCG--------------
ACGAGGAGGCCGAGGAAAGCGCACCGATGGAGACCGGGCACCAAGGCGTTGCAGGAGATTCGGCACTACCAGAAGACGTGCGACTTGCTCATCCCCCGACTGCCGTTCGCTCGCTATGTGAAGGAAATCACGTCTATGTATGCGAGTGACGTTTCTCGATGGACGGCGGAGGCTTTGACAGCCCTTCAAGAGGCAACAGAAGACTACATGGTGCATTTGTTTGAAGACACGAATTTGTGTGCCATCCATGCGAAGCGAGTCACCATAATGCCGAAAGATCTGCAGTTAGCCAGACGACTACGAG
GTGTCATCGAAAAAACATCA
>Bryum_argenteum
ATGGCGAGGAGGAAATCGACTCCTCGGCATGGCCGGAACCGGGCGTCCACGAGC---GCTGCTGGTGGAGCTGCA------GCANNNNNN-----------NNN--------------
GCGCGGCAACGAAGGAGACCGCATCGATGGAGACCAGGCACCAAGGCACTCCAAGAGATTCGGCATTACCAAAAGACATGCGACCTCCTCATACCTAGACTTCCGTTCGCGCGCTATGTGAAAGAAATCACGTCTATGTATGCAAGTGATGTGTCTCGGTGGACGGCTGAAGCTTTAACAGCCCTTCAAGAAGCAACTGAAGATTACATAGTGCATTTGTTTGAAGACACGAATTTGTGCGCTATCCACGCTAAGCGAGTCACCATAATGCCAAAGGACTTGCAATTGGCGAGACGGCTACGCGGT
GTGATTGAGAAAACAGTA
>Ceratodon_purpureus
ATGGCGAGGCTGAAGCAGACGCCGCTGCGGAGCAACAACCGGGCGTCGACGAGC---TCTGCTGGTCGAGCTGCA------CCCTCTCCTGCC---------
GCTAGAGCGGCACAGCGCGCGCCGCGGCCGAGGAAGCCGCACCGGTGGAGGCCTGGGACTAAGGCGTTGATGGAGATTCGGTACTACCAGAAGACTTGCGACCTGCTCATCCCTCGCCTTCCTTTCGCTCGCTATGTGAAGGAAATTACGTCTATGTACGCAAGTGATGTTTCTCGATGGACGGCAGAAGCGTTGACAGCTCTTCAAGAGGCAACAGAAGACTACATAGTGCATTTGTTTGAGGACACAAATTTGTGTGCTATCCATGCAAAGCGAGTCACCATAATGCCGAAGGATCTGCAGTTG
GCCAGACGCCTGCGGGGTGTCATCGAGAAAGCATCA
>Hedwigia_ciliata
ATGGCGAGGCGGAAGTCTACTCCCCTGCATGGCCGCAGACGCGCGTCCACTAGC---TCTGCAGGGGGAGCTGCA------NNNNNN--------------NNN--------------
GCGCGGAGGCCTCGGAAACCGCACAGATGGAGACCAGGCACCAAGGCATTGCAAGAGATCCGCCACTACCAGAAGACTTGCGACTTGCTTATTCCTCGACTTCCTTTTGCTCGCTATGTGAAAGAAATCACGTCTATGTATGCAAGTGACGTTTCTCGATGGACGGCGGAGGCTTTGACAGCGCTTCAAGAGGCAACAGAAGACTACATGGTGCATTTATTTGAAGACACAAATTTGTGTGCCATCCATGCGAAGCGAGTCACCATAATGCCGAAGGATCTGCAGTTGGCCAGACGACTACGAGGT
TTCATCGAAAAAGCATCA
>Leucodon_brachypus
ATGGCGAGGCGGAAGTCGACCCCTCTGCACGGAGAAAAACGCGCACCTACGCGC---TCG---------NNNGCT------GCTGCTGCTGCT---------TCT--------------
ACGACGAGGCCCAGGAAAGCGCACCGATGGAGACCAGGCACCAAGGCGTTGCAGGAGATTCGGCACTACCAGAAGACGTGTGACTTGCTCATCCCCCGACTGCCGTTTGCTCGCTATGTGAAGGAAATCACGTCTATGTATGCGAGTGACGTCTCTAGATGGACGGCGGAGGCTTTGACAGCCCTTCAAGAGGCAACAGAAGACTACATGGTGCACTTCTTTGAAGACACTAATTTGTGTGCCATCCATGCGAAGCGAGTCACCATAATGCCGAAGGATCTGCAGTTAGCCAGACGACTACGAGG
AGTCATCGAAAAAGCATCA
>Physcomitrella_patens
ATGGCAAGAAGGAAAACTACCCCTGTACATGGCAACCACCGAGCTTCTACTAGC---TCTGTTGGTGGAGCTGCG------NNN-----------------------------------------
AGGCCCAGGAAGCCGCACCGATGGAGACCCGGGACCAAGGCATTGCAAGAGATCCGCCATTATCAAAAGACGTGCGACCTTCTCATCCCTCGACTACCTTTTGCCCGCTATGTGAAGGAAATTACGATGATGTACGCTAGTGATGTTTCCCGGTGGACTGCGGAAGCTTTGACCGCTCTCCAAGAGGCCACTGAAGATTATATGTGCCATTTGTTTGAAGACACCAATTTGTGTGCTATCCATGCTAAGCGAGTAACCATAATGCCGAAGGATCTGCAGTTGGCCAGACGACTACGAGGTGCCATTN
NN---------
>Pohlia_nutans
ATGGCGAGGCGGAAGTCCACCCCTCTGCACGGCAACAGACGCGCCTCCACGAGC---TCC--------------AATCCTGCTGCTGCTGCT---------GCT--------------
CAGCGGAGGCCTCGGAAACCGCACCGATGGAGACCCGGTACCAAGGCGTTGCAAGAGATTCGCCAATACCAGAAGACTGGCGACTTGCTTATCCCCCGCCTCCCGTTTGCTCGCTACGTGAAGGAAATCACGTCAATGTATGCAAGTGACGTTTCTCGATGGACGGCGGAGGCTTTGACAGCCCTTCAAGAGGCAACAGAAGACTACATGGTGCATTTGTTTGAAGACACAAATTTGTGTGCCATCCATGCGAAGCGAGTCACCATAATGCCGAAAGATCTGCAGTTGGCCAGACGACTACGAGGT
GTCATCGAAAAAGCATCA
>Rhynchostegium_serrulatum
ATGGCGAGGCGGAAGTCGACTCCTCTGCACGGCAACAAACGCGCACCCACGCGCCCATCT---------NNNGCT------GGTGCTTCTTCT---------TCT--------------
ACGACGAGGCCGAGGAAAGCGCACCGATGGAGACCGGGCACCAAGGCGTTGCAGGAGATTCGGCACTACCAGAAGACGTGCGACTTGCTCATCCCACGACTGCCGTTTGCTCGCTATGTGAAGGAAATCACGTCAATGTATGCGAGTGACGTTTCTCGATGGACGGCGGAGGCTTTGACAGCCCTTCAAGAGGCAACAGAAGACTACATGGTGCATTTGTTTGAAGACACAAATTTGTGTGCCATCCATGCGAAGCGAGTCACCATAATGCCGAAGGATCTGCAGTTAGCCAGACGACTACGAG
GTGTCATCGAAAAATCATCA
>Rosulabryum_capillare
ATGGCGAGGAGGAAATCGACTCCTCGGCATGGCCGCAACCGGGCGTCCACGAGC---GCTGCTGGTGGAGCTGNA------GCAGCTGCTGCT---------GCT--------------
GCGCGGCAACGAAGGAGACCGCATCGATGGAGACCAGGCACCAAGGCACTCCAAGAGATTCGGCATTACCAAAAGACGTGCGACCTCCTCATTCCTAGACTTCCGTTCGCGCGCTATGTGAAAGAAATCACGTCTATGTACGCAAGTGATGTGTCTCGGTGGACGGCGGAAGCTTTAACAGCCCTTCAAGAAGCAACTGAAGACTACATAGTNCATTTGTTTGAAGACACGAAT-----------------------------------------------------------------------------------------
>Thuidium_delicatulum
ATGGCGAGGCGGAAGTCGACCCCTCTGCACGGCAACAAACGCACTCCCACTCGC---TCA---------NNNGCC------GCTGCTGCTGCT---------TCT--------------
ACGTCGAGGCCGAGGAAAGCGCACCGATGGAGACCGGGCACCAAGGCGTTGCAGGAGATTCGGCACTACCAGAAGACGTGCGACTTGCTCATCCCCCGACTGCCGTTTGCTCGCTATGTGAAGGAAATCACGTCTATGTACGCAAGTGACGTTTCTAGATGGACGGCGGAGGGTNTNACAGCCCTTCAAGAGGCAACAGAAGACTACATGGTGCATTTGTTTGAAGACACAAATTTGTGTGCCATCCATGCGAAGCGAGTCACCATAATGCCGAAGGATCTGCAGTTAGCCAGACGACTACGAG
GTGTCATCGAAAAAGCATCA
```

Fabaceae – CenH3 alignment inferred in Bali-Phy. All the codons with reliability score below 80% are masked with NNN.

>Arachis_appressipila
ATGGCAAGAGTGAAGCATATTCCAACACCTAGTCAAAAAGGT---AAGAAAAAAANNN--AGANNN------NNN------NNNTCTCCATCGCCANNNNNNNNNNNNGGTAGCAGA---NNN---NNNNNNNNNNNNNNNNNNNNN---NNNNNN---------------------CCANNNNNNNGGACGACGANNNNNNNGCANNN---------------
AAGAAAAGGCGTAATAAGCCAGGAACAGTAGCTCTTCGTGAGATTCGTAAATTTCAGAAGAGTTTCAACCTACTCATCCCAGCTGCCCCCTTCATGAGATGTGTCAAACAGATTACAAACCAACTATCTACGGAGGTCAATCGCTGGACAGCTGAAGCCATGGTAGCACTTCAAGAAGCAGCTGAGGATCATCTGGTTCGTTTGTTTGAAGATGGAATGTTGTGTGCTATCCATGCAAAGCGTGTTACTCTAATGAAAAAGGACATAGAGTTGGCCCGGAGACTCGGAGTGATAGGAAGACCTTGG
>Astragalus_sinicus
ATGGCGAGAGTTAAAATCAAACCATCTCCTCGTAAA------------------------CGC------------------------NNN-- --------
AGTGAGAGTCAGGGGAAGAAAAAAAGGCGCTATAAACCTGGAACAGTAGCACTTCGTGAGATTCGTCATTTCCAAAAGTCTGTCAATTTACTCATACCGGCTGCTCCTTTTATAAGATGTGTCAAACAGATTACAAATAACTTTTCTACAGAAGTCTCGCGCTGGACACCTGACGCCGTGTTAGCACTTCAAGAGGCAGCTGAGGATTATCTGGTTAAATTGTTTGAAGACGGGATGCTCTGTGCAATTCATGCAAGGCGTGTTACCCTTTTGAAAAAGGATTTTGAGTTGGCGCGGAGACTTGGAGG
AATAGGAAGGCCTTGG
>Cajanus_cajan
ATGGCGAGAGTGAAGCACACGCCAGCTTCTCGCAAGCCTGGTAGGNNNNNNAAAGCGNNN--CCAGAATCACCG------CAA------------GCTCAATCGCCCGCAACTAGAGAGNNNAGGNNNAGAGCTGAACAAGAGTTGCCGCAGGAGAAT---------------------GAAGCA---------GCGGCA------
NNNACTCACGGAAGAAAGAAAAGGCGCAGTAAGCCGGGAACTGCGGCGCTTCGCGAGATTCGTCATTATCAGAAGTCTTGCCAGCTTCTTATCCCAGTCGCACCCTTTATTAGATGCGTCAAAGAGATAACGCATCAATACTCTACGGAGGTGTCTCGTTGGACACCCGAAGCTGTGTTAGCACTTCAGGAGGCAGCTGAGGAATATCTGGTTCACTTGTTTGAAGACGGAATGCTCTGTGCAATTCATGCAAAGCGTGTTACTCTTATGAAAAAGGATATTGAGTTGGCTCGGAGGCTTGGAGGA
ATAGGAAGGCCTTGG
>Cajanus_cajanifolius
ATGGCGAGAGTGAAGCACACGCCAGCCTCTCGCAAGCCTGGTAGGNNNNNNAAAGCGNNN--CCAGAATCACCG------CAA------------GCTCAATCGCCCGCAACTAGAGAGNNNAGGNNNAGAGCTGAACAAGAGTTGCCGCAGGAGAAT---------------------GAAGCA---------GCGGCA------
NNNACTCACGGAAGAAAGAAAAGGCGCAGTAAGCCGGGAACTGCGGCGCTTCGCGAGATTCGTCATTATCAGAAGTCTTGCCAGCTTCTTATCCCAGTCGCACCCTTTATTAGATGCGTCAAAGAGATAACGCATCAATACTCTACGGAGGTGTCTCGTTGGACACCCGAAGCTGTGTTAGCACTTCAGGAGGCAGCTGAGGAATATCTGGTTCACTTGTTTGAAGACGGAATGCTCTGTGCAATTCATGCAAAGCGTGTTACTCTTATGAAAAAGGATATTGAGTTGGCTCGGAGGCTTGGAGGA
ATAGGAAGGCCTTGG
>Cajanus_scarabaeoides
ATGGCGAGAGTGAAGCACACGCCAGCTTCTCGCAAGCCTGGTAGGNNNNNNAAAGCGNNN--CCAGAATCACCG------CAA------------GCTCAATCGCCCGCAACTAGAGAGNNNAGGNNNAGAGCTGAACAAGAGTTGCCGCAGGAGAAT---------------------GAAGCA---------GCGGCA------
NNNACTCACGGAAGAAAGAAAAGGCGCAGTAAGCCGGGAACTGCGGCGCTTCGCGAGATTCGTCATTATCAGAAGTCTTGCCAGCTTCTTATCCCAGTCGCACCCTTTATTAGATGCGTCAAAGAGATAACGCATCAATACTCTACGGAGGTGTCTCGTTGGACACCCGAAGCTGTGTTAGCACTTCAGGAGGCAGCTGAGGAATATCTGGTTCACTTGTTTGAAGACGGAATGCTCTGTGCAATTCATGCAAAGCGTGTTACTCTTATGAAAAAGGATATTGAGTTGGCTCGGAGGCTTGGAGGA
ATAGGAAGGCCTNNN
>Cicer_bijugum
ATGGCTAGAGTTAAGCACATTCCTCCTCCTCGTAAT------------------------CGCGCT---------------------GTA-- --------
AGTGAGGATCAGGAACATAAGAAAAGACGCAATAAGCCTGGAACAGTGGCGCTTCGCGAGATTCGTCGTTTTCAGAAGACTTTCAATTTGCTTTTACCAGCTGCTCCATTTATTAGATGCGTCAAACAGATTACGAACCAAACATCTTCACATGTCTCACGTTGGTCGCCGGAAGCAGTAATAGCACTTCAGGAGGCGGCTGAGGATTATCTGGTACATATGTTTGAAAATGGAATGCTATGTGCACTTCATGCAAGGCGTATTACCCTTATGAAAAAAGATATTGAGTTGACCCGTAGGCTTACAGG
AATAGGAAGGCCGTGG
>Cicer_judaicum
ATGGCTAGAGTTAAGCACATTCCTCCTCCTCGTAAT------------------------CGCGCT---------------------GTA-- --------
CGTGAGGATCAGGAACATAAGAAAAGACGCAATAAGCCTGGAACAGTGGCGCTTCGTGAGATTCGTCGTTTTCAGAAGACTTTCAATTTGCTTTTACCAGCTGCTCCATTTATAAGATGCGTCAAACAGATTACGAACCAAACATCTTCACATGTCTCACGTTGGTCGCCGGAAGCAGTAATAGCACTTCAGGAGGCGGCTGAGGATTATCTGGTACATATGTTTGAAAATGGAATGCTATGTGCACTTCATGCAAAGCGTATTACCCTTATGAAAAAAGATATTGAGTTGACCCGTAGGCTTACAGG
AATAGGAAGGCCGTGG
>Cicer_pinnatifidum
ATGGCTAGAGTTAAGCACATTCCTCCTCCTCGTAAT------------------------CGCGCT---------------------GTA-- --------
AGTGAGGATCAGGAACATAAGAAAAGACGCAATAAGCCTGGAACAGTGGCGCTTCGCGAGATTCGTCGTTTTCAAAAGACTTTCAATTTGCTTTTACCAGCTGCTCCATTTATAAGATGCGTCAAACAGATTACGAACCAAACATCTTCACATGTCTCACGTTGGTCGCCGGAAGCAGTAATAGCACTTCAGGAGGCGGCTGAGGATTATCTGGTACATATGTTTGAAAATGGAATGCTATGTGCACTTCATGCAAGGCGTATTACCCTTATGAAAAAGGATATTGAGTTGACCCGTAGGCTTACAGG
AATAGGAAGGCCTTGG
>Cicer_reticulatum
ATGGCTAGAGTTAAGCACATTCCTCCTCCTCGTAAT------------------------CGCGCT---------------------GTA-- --------
AGTGAGGATCAGAAACATAAGAAAAGACGCAATAAGCCTGGAACAGTAGCGCTTCGCGAGATTCGTCGTTTTCAAAAGACTTTCAATTTGGTTATACCAGCTGCTCCATTTATAAGATGCGTCAAACAGATTACGAACCAAACATCTTCACATGTCTCACGTTGGTCGCCGGAAGCAGTAATAGCACTTCAGGAGGCGGCTGAGGATTATCTGGTTCATATGTTTGAAAATGGAATGCTATGTGCCCTTCATGCAAGGCGTATTACCCTTATGAAAAAGGATATTGAGTTGACCCGTAGGCTTACAGG
AATAGGAAGGCCTTGG
>Cicer_yamashitae
ATGGCTAGAGTTAAGCACATTCCTCCTCCTCGTAAC------------------------CGCGCT---------------------GTA-- --------
AGTGAGGATCAGGAGCATAAGAAAAGACGCAATAAGCCTGGAACGGTGGCGCTTCGCGAGATTCGTCGTTTTCAAAAGACTTTCAATTTGCTTATACCAGCTGCTCCATTTATAAGATGCGTCAAACAGATTACAAACCAAACATCTTCACATGTCTCACGTTGGTCGCCGGAAGCAGTAATAGCACTTCAGGAGGCGGCTGAGGATTATCTGGTTCATATGTTTGAAAATGGAATGCTCTGTGCACTTCATGCAAGGCGTATTACCCTTATGAAAAAGGATATAGAGTTGACCCGTAGGCTTACAGG
AATAGGAAGGCCTTGG
>Glycine_max
ATGGCGAGAGTGAAGCACACGCCAGCTTCTCGCAAATCCGCT---AAAAAGCAAGCGCCACGCNNN------------------------------TCCACTTCCACGCAGCCG------NNN------CCA------NNN------------TCCCAATCGCCTGCAACTAGAGAGAGG---AGGAGAGCTCAACAAGTGGAGCCGCAGNNN------------------------NNN---------------------
NNNGAGGCTCAGGGAAGGAAGAAGAGGCGCAATAGGTCGGGAACTGTGGCGCTTCGTGAGATTCGGCATTTTCAGAGGAGTTGTGAGCTTCTCATCCCGGCTGCTCCCTTTATCAGATGTGTCAAACAGATTACAAATCAATTCTCTACGGAGGTGTCTCGTTGGACACCTGAAGCTGTGGTAGCACTTCAGGAGGCAGCTGAGGAATATCTGGTTCACTTATTTGAAGATGGAATGCTCTGTGCAATTCATGCAAGGCGTATTACTCTTATGAAAAAGGACATTGAGTTGGCCCGGAGGCTTGGA
GGAATAGGAAGGCCTTGG
>Lens_culinaris
ATGGCGAGAGTTAAACAAACACCACGTCCC--GTA-- --------
CTTCAGAATCAGGAAAGGAAGAAAAGACGGAATAAACCTGGAACCGTAGCGCTTCGTGAGATCCGGAAACTTCAAAAAACTTTCCAATTACTTATACCATATGCTCCCTTTGTAAGATGTGTCAGGGAAATTACAAACCAAGTATCTTCTTCGGTTACGCGCTGGACGCCGGAAGCATTGATTTCACTTCAAGAGGCAGCTGAGGATTGTCTAGTTCGAATGTTTGAAGCTGGATGTCTCTGTGCACTTCATGCAAAGCGTGTTACCCTTATGAAAAAGGATATTGAATTGACGCGAAGGCTTACCGG
GGTAGCAAGACCTTGG
>Lotus_japonicus
ATGGCGAGAATCAAGCACGTTCCTGCTGCTCGTAGAACCGCT---CGAAAGAAAGCACCANNNNNNN---------------------NNNGCACCATCCACATCTNNN------ACA---NNN------CAA------NNN------------ACCCAATCACCTGGTCGTCGAGAGAGG---AGTAGAGAACAACAACGGGAACCGCAG--
TCANNNGGGACTCAGGGGAAGAAGAAGAGGCGCAATAGGCCGGGAACGGTGGCGCTTCGTGAGATCAGGCGTTATCAGAAGAGTGTAGACTTGCTTATCCCTGCTGCCCCCTTTCTGAGATTGGTTAGAGAGACTACACGTCAGTTATCTTTTGAGGTTTCTCGCTGGACAGCTGAAGCTGTGGTGGCTCTGCAGGAGGCAGCGGAGGACTATCTTGTTACTATGTTTGAAGATGGAATGCTCTGCGCAATTCATGCAAAGCGCGTTACCCTTATGAAAAAGGATATTGAGTTGGCCCGTCGGCTT
CGAGGAATAGGAAGGCCTTGG
>Medicago_truncatula
ATGGCAAGAGTCAAGCACATTCCACGTCCTGGTAAA------------------------CGCACTCGTCGCAGTAGTAGTAGTAATGCA-- --------
AATGAGTCTGAGGAAAAGAAGAAAAGAAGGAATAGACCAGGAACAGTTGCGCTTCGCGAGATTCGTAAATTTCAAAAGGCTGTTAACTTGCTTATACCTTGTGCTCCGTTTGTTAGATGCGTCAAACAGATTACAAACCAACTATCTATGGAGGTATCACGTTGGACGGCTGAAGCCTTATTAGCACTTCAGGAGGCAGCTGAGGAGCATCTGGTTCGTATGTTTGAAGGTGGGATGCTCTGTGCACTTCATGCAAAGCGTGTTACCCTTATGAAAAAGGATCTTGAGTTGACCCGTAGGCTTACAG
GAATAGGATGCGCTCGG
>Phaseolus_vulgaris
ATGGCGAGAGTGAAGACCACGCCAGCTTCGCGCAAAACCGGT---AAAAAAAAAGCCGCACGCNNN------------------------------TCCACGTCCACG------------NNN---------CGGTCANNN---------------CAATCGCCT---------GGGAGG---AGAAGGGCTCAACAAGAGGAGCCGCAGNNN---GCAGAAGAAGAAGAAGAA---NNNNNN---------
NNNNNNGCGNNNGAGACTCAGGGAAGGAAGAAAAGGCGCAATAAGGCAGGAACAGTGGCGCTTCGCGAGATTCGTCAATTTCAGAGGAGTTCCAAACTTCTTATCCCGGCTGCCCCTTTTATGAGATGTGTCAGACAAATTACACAGCAATTCTCTGCGGAGGTGAGTCGCTGGACACCTGAAGCAGTGGTAGCACTCCAGGAGGCAGCTGAGGAATGTCTAGTTCACTTGTTTGAAGATGGAATGCTTTGTGCAATTCACGCAAGGCGTGTTACTCTTATGACAAAGGATATTCAGTTGGCTCG
GAGACTTGGAGGAATAGGAAGGCCTTGG
>Pisum_sativumB
ATGGCGAGAGTTAAACAAACACCACGTCAC--GCC-- --------
CGTGAGAATCAGGAAAGGAAGAAAAGACGTAATAAACCTGGAACCGTAGCGCTTCGTGAGATCAAGAAATTGCAAAAAACTTTCCAATTACTTATACCATATGCTCCGTTTGTAAGATGCGTCAGGGAAATTACAAATCAAGTATCTTCATTGGTTACACGCTGGACGCCTGAAGCGTTGCTATCACTTCAAGAGGCAGCTGAGGATTGTCTAGTTCGAATGTTTGAAGCTGGATGGCTCTGTACACTTCATGCAAAGCGTGTTACCCTTATGAAAAAGGATATTGAGTTAACGCGCAGGCTTACCGG
GATAGGAAGACCTTGG
>Vigna_unguiculata
ATGGCGAGAGTGAAGCACACGCCAGCTTCGCTCAAAGTTGGT---AAAAAAAAAGTCAGTCGCNNN------------------------------TCCACATCCACG------------NNN---------------NNN---------------CAATCGCCTGCGACAAGAAGTCGT---AGAAGGGCTCAAGAAGAGGAGCCGCAGGAA------------------------NNNNNN---------
NNNNNNGCGNNNCAGACTCAGGGAAGGAAGAAAAAGCGCAGTAAGCCAGGAACAGCGGCGCTTCGCGAGATTCGTCATTTTCAGAAGAGTTGCAAGCTTCTTATCCCGGCTGCCCCCTTTATCAGATGTGTCAAACAAATTACACATCAATTCTCTACGGAGGTGTCTCGCTGGACGCCTGAAGCTGTGGTAGCACTGCAGGAGGCAGCTGAAGAATGTCTAGTTCACTTGTTTGAAGATGGAATGCTTTGTGCAATTCACGCTAGGCGTATTACTCTTATGACAAAGGATATTCAGTTGGCTCGG
AGGCTTGGAGGAATAGGAAGGCCTTGG

Ferns – CenH3 alignment inferred in Bali-Phy. All the codons with reliability score below 80% are masked with NNN.

```
>Azolla_filiculoides
ATGGCGAGACTCAAGCAAAGACCACACAGGTCTAATCTGGCTGCATTGTCGTCCAATGTT---------GAAACTACA------GGAAGTAGCCAGCCCCATTTGAATCGCAGTAATAGT--------------------GCGCCACCAACTGCTCCC--------------------------------------------------------------------------------------ACAACA-------------------------------------------------------------------------------------------------------------------- ---------------------------------------------------------
NNNTTCAAC------------------
AATATTCCTGCTAATCGCCAACGCAAACCTCATCGATTTAGGCCTGGTACAGTGGCTTTAAGAGAAATCAGGCGCTACCAAAAGACTGTTCACTTTCTTATCCATCCCTTACCATTTGCACGACTGGTCAGGGAACTAACTGCCCAATTTTCAGACACAGTGAGCCGATGGACAGCTGAGGCATTGGTTGCTTTGCAAGAGGCTGCTGAGGATCATTTGGTGCATTTGTTTGAGGACACTAATTTATGTGCTATCCATGCAAAACCTGTCACAATAATGCCTAAAGATATGCAGCTTGCAAGACGTTTGC
GTGGCAACACCATTGACAGGCCTTGG
>Ceratopteris_richardii
ATGGCGAAGAAAAAGAAGACCCCGAAAAAGGCTTCCCAGGTCGCTTCTTCCTCTACCGTCCCTCAGCCTGAGATCACAGATGCANNNAACAGCAGAAGACGACTCCATCGAAGCAGTAGT--------------------GCACCTCCAACTGCTCCA--------------------------------------------------------------------------------------GTTACT-------------------------------------------------------------------------------------------------------------------- --------------------------------------------------------
-
NNNACAAATGAACGAGAGCCTACAGACAATACACCTTCCAGGCGTCCACGCAAACCCCACAGATTTCGTCCAGGAACAGTAGCTCTTCGGGAAATTCGACATTATCAGAGAACTGTTAATTTTCTCATACATCCATTGCCATTTGCCAGATTGGTGAGAGAAATTGGGGTCCAATTTTCAGATACTGTCTCTCGGTGGACTGCAGAATCACTTCTAGCATTACAGGAGGCTGCTGAGGATCACATTGTACACCTATTTGAGGATACAAACCTATGTGCCATACATGCTAAGCGTGTCACCATAATGCCA
AAGGATATGCAATTAGCAAGGAGACTTAGAGCAAGTACCCTTGACAGGCCTTGG
>Equisetum_arvense
ATGGCGAAGAAGAAA---------------------------------------------------------------------------------------------------------------------- ----------------------------------------------------------------------------------------------------------------------
AGTGCTCCCGCGCGCTCCCAAAAGTCCCCTGCTTCCACAGTCGTCGACGGAAATGGGCAGCCTTCCAGTAAGAATGCAAAGGATCAAGGTGGGCCTTCAAGCACTCCCGATACTGCAAATGTTGGTGGCAGAGGAGTTAAAGGGGCAAAGGGAAAAGGAAGAACACAGCAGGGTGCCGAAAACCCT---------------------------------------------
CGGAAGTCACACCGTTACAAAGCCGGAACGGTTGCACTGCGGGAAATCAGATTCTACCAGAAGAATGTAGATCTACTCATTCGGCCTCTCCCCTTTGCACGCTTGGTGCGAGAAATTGCTGATCACCTCTCTCGGACTGTTACTCGCTGGACAGCTGAAGCCTTGGTGGTTATGCAAGAGGCATGCGAGGATTTTATTGTCCATCTTTTTGAGGACACCAATCTTTGTGCAATTCATGCCAAACGCGTAACTATAATGACTAAGGACATGCAACTTGCTAGGCGGCTCCGTAATACAATGATTGATAGG
CCGTGG
>Equisetum_diffusum
ATGGCGAAGAAGAAA---------------------------------------------------------------------------------------------------------------------- ----------------------------------------------------------------------------------------------------------------------
AGTGCGCCCGCGCGCTCCCAAAAGTCCCCTGCTTCCACAGTCGTCGACGGAAATGGGCAGCCTTCCAGTAAGAATGCAAAGGATCAAGGTGGGCCTTCAAGCACTCCCGATACTGCAAATGTTGGTGGCAGAGGAGTTAAAGGGGCAAAGGGAAAAGGAAGAACACAGCAGGGTGCCCAAAACCCT---------------------------------------------
CGGAAGTCACACCGTTACAAAGCCGGAACGGTTGCACTGCGGGAAATCAGATTCTACCAGAAGAATGTAGATCTACTCATTCGGCCTCTCCCCTTTGCACGCTTGGTGCGAGAAATTGCTGATCACCTCTCTCGGACTGTTACTCGCTGGACAGCTGAAGCCTTGGTGGTTATGCAAGAGGCATGCGAGGATTTTATTGTCCATCTTTTTGAGGACACCAATCTTTGTGCAATTCATGCCAAGCGCGTAACTATAATGACTAAGGACATGCAACTTGCTAGGCGGCTCCGTAATACAATGATTGATAGG
CCGTGG
>Lygodium_japonicum
ATGGCCAGAAGGAAGCCTAATCCAAAGAAGGCCACGCCAGTTGCTTCTTCTTCGAACGCCACACCGCAGGAGGCGACA------GCAAGCAGCAGACCGCGCTTAGTTAGAAGCAGTAGT--------------------GCACCACCGACAACCCCA--------------------------------------------------------------------------------------GCAACA------------------------------------------------------------------------------------------------------------------- ---------------------------------------------------------
NNNTCAAAT------------------NNNNNNNNNNNN---
CGACAGCGCAAGCCTCATCGGTTTAGACCCGGAACTGTTGCTTTGCGGGAAATCAGGCATTTTCAGAAGACAGTTCACTTTCTTATTCATCCATTACCATTTGCACGGCTGGTTAGAGAAATTGCAGCTCAATGTTCGGATACAGTAACTCGATGGACTGCCGAAGCATTGGTTGCTTTGCAGGAGGCTGCAGAAGACCACCTTGTACACTTGTTTGAGGACACTAATCTGTGTGCTATTCATGCAAAACGAGTCACAATAATGACAAAAGACATGCAACTTGCACGGCGTTTACGAGGCAGCACTCTA
GATAGGCCGTGG
>Ophioglossum_petiolatum
ATGGCTCGAAGGAAGCCGAGGCCGCAAAAAGCA---------------------------------------------------------------------------------------------------- -------------NNNNNNNNNNNNNNNNNNNACTTCAACAGCCGTCGCACCATCGCCACAGCCAGTAGGCGGTCGTAAAGGAAGTCGTAAAACAGCCGGCAGACGCCGTGCTACCGCGNNNNNN-------------------------------------------------------------------------------------------------------------------------------- --------------------------------------------
----------CCC---------------------------------------------
AAAAAGCCCCACCGTTTCAAGCCCGGTACCGTTGCTTTGAGAGAGATCAAATATTACCAGAAGAAATTTCACCTCCTCATTCGTCGTCTTCCCTTTGCAAGATTAGTTAAAGAAATTACGGCTCATTTTTCACGCAATGTTACTCGCTGGACAGCGGAGGCGCTGACTGCTTTGCAAGAGGCAGCTGAAGACCAAATTGTTCATCTTTTTGAGGACACCAATTTATGTGCCATCCACGCAAAGCGCGTTACAATAATGCCAAAGGATATGCAACTTGCAAGGCGTCTACGTGGGAACACAGCTGACAGG
CCATTT
>Psilotum_nudum
-----------------------------------------------------------------------------------------------------------------------AGTCAAACCGCGAGACGCACCNNNNNNNNNNNNNNNNNN---------------------------------------------------------------------------------------NNNNNN---------------------------------------------------------------------------------------------------------------------------- ------------------------------------------------------TCT---------------------------------------------
AGAAAGCGTCATCGGTTTCGGCCTGGAACAGTGGCATTACGAGAAATTAGATTTTATCAGAAGCGCTTTCATCTTTTAATTCCACCACTGCCTTTTGCCCGACTGGTCAAGGAAATGACATTATATTTTTCTCGCGATGTGTCCCGCTGGACGGCCGAGGCATTAGTGGCGTTGCAGGAGGCTGCTGAGGATTTCATCGTTCATCTGTTTGAGGACACCAATTTATGTGCTATTCATGCAAGACGTGTCACAATAATGACCAAAGACATGCCGCTTGCCAGGCGCTTGCGTGGAGCCATTGCTGATAGG
CCCTGG
>Pteridium_aquilinum
----------------------------------------------------------------------------------------------------------------------------------------------------------------------------------------------------------------------------------------------------------------------------------------------------- ------------------------------------------------------------------------------------------------------------------------------------------------------------ ------------------------------------------------------------------------------------------
AACCGCCAACGCAAGCCCCATCGGTTTCGCCCTGGAACTGTAGCTCTCCGAGAGATTCGACATTACCAAAAGACTGTCAATTTTCTCATTCATCCCCTGCCCTTTGCCAGATTGGTGAGAGAACTGGCCGGTCAGTTTTCAGACTCAGTATCCCGGTGGACTGCAGAGGCACTGGTTGCGCTACAGGAGGCTACTGAGGACCACCTTGTGCACCTTTTTGAGGACACTAATTTATGCGCAATTCATGCCAAACGTGTCACAATAATGACGAAAGACATGCAACTAGCAAGGCGTTTGCGAGGAACCACT
CTGGACAGGCCTTGG
```

Lycopodiophyta – CenH3 alignment inferred in Bali-Phy. All the codons with reliability score below 80% are masked with NNN.

```
>Isoetes_sinensis
ATG--------------------NNNNNNNNNAAAAGTACCCCAAAAACGGGCAAAGCCTCTACTCCTGCAGCTTCTTCTTCAGCTACAGGAACCAGTTCTAGAAGAGTA---------------------------------GATGGTGCGAGCACTAGTAGGGCAGGAAATGCACGTAGTTCGGATAGGGTTGTAGCT---------------------------------------------------------------------------NNNNNNCAAAAAAGG------NNN---NNN---------
CGGTTTAAACCTGGAACTGTGGCGCTTAGGGAAATAAGGCATTTTCAAAAGAGCTACGGATTTCTTCTCAGGCCTTTGCCATTTGCCAGAGTGGTGAGAGAGATAACTGCCCAG------TATTCTAAAGAAGTGTCAAGATGGACTGCTGAAGCTTTAATAGCTATTCAAGAGGCTGCTGAAGACTACCTTGTACATCTTTTTGAAGACACAAATTTGTGTGCTATTCATGCCAAAAGAGTCACTATCATGCCAAAAGATTTGCAACTTGCACGTCGTTTACGAGGGGGTACGGAAAGAAAGATGNNN--
-
>Lycopodiella_caroliniana
---------------------------------------------------------------------------------------------------------------------------------------------------------------------------------------------------------------------GCTACCCCAGTTTCTGAGTCGGCAAGCAGTAGAAGAGCTGTTGGAGAACGTCAAAGACAAGCGNNNNNN---------CAGCGANNNCCTNNN---------
CGGTTCAAGCCTGGTACTGTGGCTCTCAGAGAGATTCGAAAGTATCAAAAAAGTTTCAATCTCTTGATAAGGCCCTTGCCATTTGCACGACTGGTTCGTGAAATCACATCTCAA------TTCTCTACTGATGTTACAAGATGGACAGCAGAAGCTCTGATTGCCATTCAAGAGGCTGCTGAGGATTACCTTGTTCATCTTTTTGAAGATACCAACTTATGTGCCATTCATGCCCGTCGGGTGACAATTATGCCAAAGGATCTGCATCTTGCGCGACGTCTTCGAGGAGCTTCTGAG---AAAATGTTCATC
>Selaginella_kraussiana
------------------------------------------------------------------------------------------------------------------------------------------GGTGGCGATGGCGCCGGGACGAGCAGAGCAGGT------------------------------GCCCAGCAGAAC------------------------------------------------------------------------------------AGACCA------
NNNCGGCGTTTCAAGGCCGGGACTGTAGCGCTTCGCGAAATTCGGAAGTTTCAGAAGAGCTTTGAGCTCCTCCTCAGGCCTCTACCCTTCGCCAGAGTGGTTCGGGAGCTTGCATCCTTG------TGTTCAACTGAGGTTACTAGATGGACTGCTGAATCCCTCCTTGCTCTCCAAGAGGCTGCGGAGGATTACTTGGTGCATTTGTTTGAAGACACCAACTTGTGTGCGATACATGGAAAACGGGTTACGATA---------------------------------------------------------------
>Selaginella_moellendorffii
ATG---------------------------------------------------------------------------------------------------------------------------GAGTGCGCAGCGGGCGGCGATGGCGCTGGGACGAGCAGAGCCGGC------------------------------GCCCAGCAGAAC------------------------------------------------------------------------------------
AAGCCACACGGCAAGCGCAGGTTTAAGCCCGGGACTGTGGCGTTGCGGGAGATCCGACGCTTTCAAAAGAGTTATGAGCTACTTCTCAGGCCATTGCCATTCGCAAGAGTGGTGAGAGAGATCACG------
AACGTTTTCTCGTCGGAGGTATCCAGATGGACTGCCGAAGGATTGATAGCTCTCCAAGAGGCTGCCGAGGACTACCTTGTTCATCTCTTCGAAGACACCAACTTATGTGCCATTCACGGCAAGAGAGTCACCATAATGCCCAAGGACTTACATTTGGCCCGTCGACTAAGAGGAGCCTCCGAACGG------TTCGTT
>Selaginella_stauntoniana
ATGGCCAAGAGGAGGAAATCGACGNNNNNNNNN------------------------------------------------------------------------------CGAGGGCGACCACCA---------GCGGGCGGCGATGGCGCTGGGACGAGCAGAGCCGGC------------------------------GCCCAGCAGAAC---------------------------------------------------------------------------------------
AAGCCGCACAGCAAGCGCAGGTTTAAGCCCGGGACTGTGGCGTTGCGGGAGATCCGACGCTTTCAAAAGAGCTATGAGCTCCTTCTCAGGCCATTGCCATTCGCAAGAGTGGTGAGAGAGATCACG------
AACGTTTTCTCGTCGGAGGTATCCAGATGGACTGCCGAAGGATTGATAGCTCTCCAAGAGGCTGCCGAGGACTACCTTGTTCATCTCTTCGAAGACACCAACTTATGTGCCATTCACGGCAAGAGAGTCACCATAATGCCCAAGGACTTGCATTTGGCCCGTCGACTAAGAGGAGCCTCCGAGCGG------TTCGTT
```

Poaceae – CenH3 alignment inferred in Bali-Phy. All the codons with reliability score below 80% are masked with NNN.

```
>Brachypodium_distachyon
ATGGCCCGCACGAAGCGCCCGGCCATCAGGAAGTCG---------AAGCCGCAGCCCAAGAAGCAACTCCAGTTCGAGNNN-------------------------------------ACAGGCGGC---GCGAGCACCNNNGCGTCGNNNACCCCGNNNNNNNCGT---NNNNNNCGGACCCCGGCGCGAGCGGCGGGTCAAGCG------GCACCCGCACAACAG---NNNCCG------AAGAAGCCA---------
CACAGATTCCGGGCAGGCACGGTGGCGCTGCGGGAGATCAGGAAGTACCAGAAATCCCCTGAGCTGCTCATCCCATTCGCACCCTTCGTCCGTCTGATTAAGGAGATCAGTAATTTCTACTCACCTGAG--------------------------ATCTCGCGCTGG---------------ACTCCTCAAGCTCTCGTTGCTTTGCAAGAGGCTGCAGAATACCACTTGGTAAACATATTTGAAAAGGCAAATTACTGTGCCATCCATGCGAAGCGTGTTACCATGATGCAAAAGGACATACAGCTTGCGAGG-------------------
----------
>Cenchrus_americanus
ATGGCTCGAACCAAGCACCAGGCCGTGAGGNNNNNNGGCCGC---------NNNCCCAAGAAGAAGCTCCAGTTCGAGCGCTCCCCTCGCCAGNNNNNN---GCGCAG------------ACAGGCGGC---GCGAGCACCTCG------GGAACCTCGGGGAGG------GGTNNNCCGGCTCGGGGTGGAGCGGCTGCCGCTGGG------GCGGCAGGGNNN---------------ATTNNNAGGCCG---------
CATCGGTGGCGACCAGGGACGGTGGCTCTGCGGGAGATCAGAAAATTCCAGAAATCTACCAATCCTCTTATCCCATTTGCTCCCTTTATTCGTCTGGTGAGGGAGATCACTAACGACTATTCGAAGGAA--------------------------GTGACACGCTGG---------------
ACTCCTGAAGCCCTCCTTGCGCTGCAAGAGGCAGCAGAGTTCCACTTAATTGAGCTGTTTGAAGTGGCAAATTTGTTGGCCATCCATGGGAAGCGTGTTACTATCATGCAAAGGGACATACAGCTTGCAAGGCGTATCGGAGGA---NNNCGTTGGTCG---
>Hordeum_bulbosum
ATGGCCCGCACCAAGCACCCCGCCGTGAGGAAGTCC---------AAGGCGCCGCCCAGGAAGAAGGTCGGGTCCGCGCGCGCCCCGNNN------NNN---GCGCAGCGCCGGCACGAGACAGATGGC---GCGGGCACGTCC------GAGACTCCGAGG---------------NNNGGGCCGGCCCCGGCGGCGGATCAAGGG------GCACCTGGGGAACCC---NNNAAG------AGGAAGCCA---------
CACCGGTACAGGCCAGGCACGGTGGCACTGCGGGAGATCAGGAAGTACCAGAAGTCGGTCGATTTTCTCATCCCGTTTGCACCGTTTGTCCGTCTGGTCAAGGAGGTCACCGAATTCTACTGTCCTGCA--------------------------ATCAGCCGCTGG---------------ACTCCCCAAGCGCTCTTGGCAGTTCAAGAGGCTGCAGAGTATCACCTCGTCGACGTATTTGAAAGGGCACATCTCTGTGCCATCCATGCAAAGCGTGTTACCGTCATGCAAAAGGACATACAACTCGCA------------------------
--------
>Hordeum_marinum
------------------------------------------------------------------------------------------------------------------------------------------------------------------------------------------------------------------------ ------------------------------GTGGCGCTGCGGGAGATCAGGAAGTACCAGAAGTCCACCGGGCTGCTCATCCCCTTCGCGCCCTTCGTCCGGCTGGTTAAGGAGATCACCNNN---------------GACTTAACGAAGGGAGAGCTGAAC---------NNNTGG---------------
ACACCTCAGGCGCTCGTCTCGTTGCAAGAGGCTGCAGAGTATCACATAGTCGATCTATTCGAAAAGGCAAATCTATGTGCCATCCATGCTAAGCGTGTTACCATGATGCAAAAGGACATACAGCTGGCAAGGCGTATCGGGGGGACAAGGCTTTGG------
>Hordeum_vulgare
ATGGCCCGCACCAAGCACCCCGCCGTGAGGAAGTCC---------AAGGCGCCGCCCAAGAAGAAGATCGGGTCCGCTAGCTCCCCGNNN------NNN---GCGCAGCGCCGGCAGGAGACAGATGGC---GCCGGCACGTCC------GAGACTCCGAGGCGG------GCCNNNCGGGGGCCGGCCCCAGCGGCGGCTGAAGGG------GCACCTGGGGAACCG---NNNAAG------AGGAAGCCA---------
CACCGGTTCAGGCCAGGCACGGTGGCACTGCGGGAGATCAGGAAGTACCAGAAGTCGGTCAATTTTCTCATCCCGTTTGCACCGTTTGTTCGTCTGGTCAGGGAGATCACCGAATACTACTGTCCTCGA--------------------------GTCAAACGCTGG---------------ACTCCCCAGGCGCTCCTCGCAGTTCAAGAGGCTACAGAGTATCACCTCGTCGACATATTTGAAAGGGCACATCTCTGTGCCATCCATGCAAAGCGTGTTACCGTCATGCAAAAGGACATGCAACTCGCG------------------------
--------
>Oaustraliensis
ATGGCTCGCACGAAGCACCCGGCGGTGAGGTCGNNN------TCAAGGACGGAGCCCAAGAAGAAGCTCCGGTTCGACCGCTCCCCTCGG---CCTTCGAAGGTGCAGAGC---------ACTGGTGGT---GCGGGTACCTCG------GCGACCACGGGGAGC---------GCGGGGACCGCGGCTNNN------------GGG------ACGCCTGGGCAGCAGACAAGGCAG------AGGAAGCCA---------
CACCGATTCCGTCCAGGCACAGTAGCACTACGGGAGATAAGGAAATTTCAGAAAACAACTGAACTTCTTATCCCTTTTGCGCCATTTTCCCGTCTGGTCAGGGAGATCACTGACTTCTATTCGAAGGAT--------------------------GTGTCACGCTGG---------------
ACTCTTGAAGCTCTCCTTGCACTGCAAGAGGCAGCAGAATACCATTTGGTGGACTTGTTTGAAGTGTCAAACCTTTGCGCCATCCATGCTAAGCGTGTTACCATCATGCAGAAGGACATACAGCTTGCCAGGCGTATCGGTGGGCGGAGGCCATGG---GGC
>Obrachyantha
ATGGCTCGCACCAAGCACCCGGCGGTGAGGAAGTCG---------AAGCCGGAGCCCAAGAAGAAGCTCCAGTTCGAGCGCTCCCCGCGGAGGNNNNNN---GCGCAGCGC-----------GGTGGC---GCGGATACCTCG------NNN---ACGAGGAGC---------GCGCGGACTGCGGCCGGAACGTCGGCCGAAGGG------ACGCCTGGACAGCAGTCGAGGCAG------AGGAAGCCA---------
CACCGATTCCGTCCAGGCACAGTAGCTCTGCGGGAGATCAGGAGATTCCAGAAATCGACCGAACTGCTTATCCCATTTGCGCCATTTTCCCGTCTGGTGAGGGAGATCACTGATTTCTATTCCAAGGAT--------------------------GTGACGCGCTGG---------------
ACTCTTGGAGCTCTCCTTGCACTGCAAGAGGCAGCAGAATACCACTTAGTGGATTTATTTGAAGTAGCAAATCTCTGCGCCATCCATGCTAAGCGTGTTACCATCATGCAAAAGGACATACAACTTGCCAGGCGTATCGGTGGACGGAGGCCATGG------
>Osativa
ATGGCTCGCACGAAGCACCCGGCGGTGAGGAAGTCG---------AAGGCGGAGCCCAAGAAGAAGCTCCAGTTCGAACGCTCCCCTCGG---CCGTCGAAGGCGCAGCGC---------GCTGGTNNNGGCACGGGTACCTCG------GCGACCACGAGGAGC------------------NNNGCTGGAACATCGGCTTCAGGG------ACGCCTAGGCAGCAAACGAAGCAG------AGGAAGCCA---------
CACCGCTTCCGTCCAGGCACAGTGGCACTGCGGGAGATCAGGAAATTTCAGAAAACCACCGAACTGCTGATCCCGTTTGCACCATTTTCTCGGCTGGTCAGGGAGATCACTGATTTCTATTCAAAGGAT--------------------------GTGTCACGGTGG---------------
ACCCTTGAAGCTCTCCTTGCATTGCAAGAGGCAGCAGAATACCACTTAGTGGACATATTTGAAGTGTCAAATCTCTGCGCCATCCATGCTAAGCGTGTTACCATCATGCAAAAGGACATGCAACTTGCCAGGCGTATCGGTGGGCGGAGGCCATGG------
>Panicum_virgatum
ATGGCTCGCACCAAGCACCCGGCCGTGAGGAAATCG---------AAGGAGCAGCCCAAGAAGAAGCTCCAGTTCGGGCGCTCCCCGCACGGGNNNNNN---ACGCCG------------ACAGGTGGA---GCGAGCACATCG------GCGACTCCGGCAAGC------GCTNNNGGGACCGGGGAGAGAGCGGCGGCTGGAGGT------ACGGCGGGGCCGCAG---NNNCAGAAGGTGNNNAAACCA---------
CACCGTTGGAAGCCAGGGACTGTAGCGCTGCGGGAGATCAGGAAGTTCCAGAAATCCACCGAGATGCTTATCCCCTTTGCACCATTTGCCCGTCTGGTGAGGGAGATCACTGAGTTCTACTCAAGGNNN-----------------------AATGTGACACGCTGG---------------
ACCCCGGAAGCCATCCTTGCAATACAAGAGGCAGCAGAATTCCACCTGATAGAACTGTTCGAAGTGGCAAATCTTTGTGCCATCCACGCCAAACGTGTTACCATCATGCAAAGGGACATACAGCTTGCAAGGCGTATCGGTGGA---NNNCGCTGG------
>Saccharum_hybrid
ATGGCTCGAACCAAGCACCAGGCCGTGAGAAGGCCA---------ACGCAGAAGCCCAAGAAGAAGCTCCAGTTCGAGCGCNNN---------------------------------------GGTGGG---GCGAGTACCTCG------GCGACCCCGGAGAGA------AATNNNGGGACCGGGGGAGGAGCGGCAGCTCGCGTT------ACACGGGGGNNN---------------GTGNNNAAGAAG---------
CTTCGCTGGCGGGCAGGGACTGTAGCGCTGCGGGAGATCAGGAAGTACCAGAAGTCCACTGAGCCGCTCATCCCCTTTGCGCCCTTCGTACGCGTGGTTAAGGAGTTAACTGGATTC---------------------------------------
NNNNNNNNNNNNAGGATAGGACGCTATACCCCTGAAGCCCTCCTTGCGCTGCAAGAGGCAGCAGAATTCCACTTGATAGAACTGTTTGAAGTGGCGAATCTGTGTGCCATCCATGCCAAGCGTGTCACAGTCATGCAAAAGGACATACAACTTGCAAGGCGTATCGGAGGA---NNNCGTTGGGCG---
>Sorghum_bicolor
ATGGCTCGAACCAAGCACCAGGCCGTGAGGAAGCTG---------CCGCAGAAGCCCAAGAAGAAGCTCCAGTTCGAGCGCNNN---------------------------------------GGTGGG---GCGAGTACGTCG------GCGACCCCGGAGNNN---AGGAATNNNGGGACCGGGGGAGGAGCCGCGGCTCGCGTT------GCACGGGGGNNN---------------GTGNNNAAGAAG---------
CATCGCTGGCGGGCAGGGACTGTAGCGCTGCGGGAGATCAGGAAGTACCAGAAGTCCACTGAGCCGCTCATCCCCTTTGCGCCCTTCGTACGTGTGGTCAAAGAGTTAACTGCATTC---------------------------------------
NNNNNNNNNNNNAGGATAGGGCGCTACACCCCTGAAGCCCTCCTTGCGCTGCAAGAGGCAGCAGAATTCCACTTGATAGAACTGTTTGAAGTGGCGAATCTGTGTGCCATCCATGCCAAGCGCGTAACAGTCATGCAAAAGGACATACAACTTGCAAGGCGTATCGGAGGA---NNNCGTTGGTCG---
>Zea_mays
ATGGCTCGAACCAAGCACCAGGCCGTGAGGAAGACG---------GCGGAGAAGCCCAAGAAGAAGCTCCAGTTCGAGCGCNNN---------------------------------------GGTGGT---GCGAGTACCTCG------GCGACGCCGGAAAGG------GCTNNNGGGACCGGGGGAAGAGCGGCGTCTGGAGGTGACTCA---------------------------
NNNNNNAAGACGAAACCACGCCACCGCTGGCGGCCAGGGACTGTAGCGCTGCGGGAGATCAGGAAGTACCAGAAGTCCACTGAACCGCTCATCCCCTTTGCGCCTTTCGTCCGTGTGGTGAGGGAGTTAACCAATTTC---------------------------------------
NNNNNNNNNNNNAAAGTAGAGCGCTATACCGCAGAAGCCCTCCTTGCGCTGCAAGAGGCAGCAGAATTCCACTTGATAGAACTGTTTGAAATGGCGAATCTGTGTGCCATCCATGCCAAGCGTGTCACAATCATGCAAAAGGACATACAACTTGCAAGGCGTATCGGAGGA---NNNCGTTGGGCA---
```

Tetrahymena – CenH3 alignment inferred in Bali-Phy. All the codons with reliability score below 80% are masked with NNN.

>borealis
AAGAGGAGAAGCGTTTCTAATTAATCTCAATAAAGATCAAATAATGCTACANNNAATAATTAAGTA---AATTAAAGATCGAAATCTGCAGGT---GGATAAATGAAT---AAATAAAAAAACCCA---TCTTAACAGTAAAATAAGCCTGTAACA------
AAAAAGAGATAATCCTCTGGTGAAAAATATGAATCTGCAAGAGATAAAGTAATTAGAGGATTCAGACCAGGAGATAATGCTTTGAAAGAACTTAAATAATATAATTAAAATCCATCACTATTTATTCGAAAACTACCTTTTTAGAGGTTAGTGAGAGAAATATCAACACGATTGTCAGATCAGGATTAAATTAGATGGACAAGTATTGCTCTAATCCTATTATAAACAGTTACAGAAGATTATATGATATCTTTTTATGAAGATGCGAATGCATGTGCTTTACACGCTAAAAGAGTTACCCTTATGAGCA
AAGATATGATTCTAGCTGCTAGAATTAGAGGATAAAGGAATGTT
>canadensis
AAGAGGAGAAGTGTTTCTAATTAATCTCAACAAAGATCAAATAATGCAACANNNAATAATTAAGTA---AATTAAAGATCGAAATCTGCAGGT---GGATAAATGAAT---AAATAAAAAAACCCA---TCTTTACAGTAAAATAAGCCTGTAACT------
AAAAAGAGATAATCCTCTGGTGAAAAATATGAATCTGCAAGAGATAAAGTAATTAGAAGATTTAGACCAGGAGATAATGCTTTGAAAGAACTTAAATAATATAATTAAAATCCATCACTATTTATTCGAAAACTACCTTTTTAGAGGTTAGTGAGAGAAATATCAACACGATTGTCAGATTAGGATTAAATTAGATGGACAAGTATTGCTCTAATTCTATTATAAACTGTTACAGAAGATTATATGATATCATTTTATGAAGATGCGAATGCATGCGCTTTACATGCTAAAAGAGTTACCCTTATGAGCA
AAGATATGATTCTTGCTGCTAGAATTAGAGGATAAAGGAATGTT
>elliotti
AAAAGAAGAAGCAATTCTAACTAAAATTAAGAGAGGTCTGAGAGTTCT---AAAAAAATTAAATAAGATAATTAAAGATCTAAATCAGCGGGAATANNNNNNGATAAT---AAANNNNNNNNNNNN---NNNNNNNNNNNNNNNNNNNNNNNNAGCACANNN---
AAAAGATAATCATCAGAGGAAAGATATGAATCAGCAAGAGATAAAGTTATCAGAAGATTTAGACCTGGTGATAATGCTCTTAAATAATTAAGATAATATAATCAAACTCCTTCGCTGCTTATAAGAAAATTGCCTTTTTAAAGGCTAATAAGGGAAATTTCTACAAGAATGACAGAAGAAGATCAGTTAAGATGGACAAGTTTTGCTTTAGTTTTACTTTAAACTGTTGTAGAAGATTATATGGTTTCATTTTTTGAAGATGCAAATGCTTGTGCTCTACATGCTAAAAGAGTAACTCTAATGTCTAAGG
ATCTTGCTCTTGCAGCAAGAATAAGGGGATAAAAAAATGTT
>furgasoni
AAAAGGAGGAGCAATTCTAACCAAAATTAATCATCATCTAATAATGTT---GCTAATAATTAATCTGCTAATCAGAGATCAAAATCAGCAGGG---CCATAAGGGAAT---AAATAAAAAAATTAG---CCTCAATAATAAAAAAAGGCTGTTTCC------
AAAAAGAGATAATCATCTGGTGAGAAATATGAATCGGCAAGAGATAAAGTAATAAGAAGATTTAGGCCAGGGGATAATGCATTAAAAGAAATGAGATAATATAATCAAACTCCATCTCTACTAATAAGAAAATTGCCTTTTTAGAGGCTAGTAAGGGAAATATCAACAAGATTGACAGAGGAAGATAATTTGAGATGGACAAGCTTTGCCTTAGTGTTATTATAAACTGTTGTTGAAGATTACATGGTATCATTTTATGAAGATGCAAATGCATGTGCTTTACATGCTAAAAGAGTTACCTTAATGAC
AAAAGATTTAGCACTAGCTGCAAGAATTAGAGGATAAAAAAATGTT
>leucophrys
AGAAGAAGGAGTAATTCAAATCAAACTTAATAAAGATCATAAAGCGCT---TCCAATAACCCATAACCACAATAAAGATCAAAATCATCAGGA---AATCAAGAAAAT---AAATAAGCGAAAGCA---
AAGCAATAATAAAAGAAACCAACTACAACAAAGAAAAAGAGATAGTCTTCAGGTGATAAGTATGAATCTGCTAGGGATAAAGTGATAAGAAGATTTAGACCTGGAGATAATGCCTTAAAAGAATTAAGATAATATAACTAAACCCCATCATTATTAATCAGAAAATTACCTTTTTAGAGACTAGTGAGAGAAATATCAACAAGAATGTCAGAGCAAGAGTAATTAAGATGGACCAGTTTTGCTTTGGTTCTATTATAGACAGTTGTGGAGGACTATATGGTCTCTTTCTATGAAGATGCTAATGCCTG
T??
>limacis
AAAAAGAGGAGTAATTCTAATTAAAGCTAATAAAGATAGAGTAGTGCA---AATAATGATTAATCAACTAATCGAAGATCTAAATCTGCAGGA---AACCAAGGGAAT---AAATAAAAGGGTTTA---
CCTCAATAATAAAAAAAGGCTGTTTCTACGAAAAAAAAGAGATAATCTTCTGGTGAGAAGTATGAATCTGCTAGAGATAAAGTAATAAGAAGATTTAGACCTGGAGATAATGCACTAAAAGAACTCAGATAATATAATTAAACTCCATCATTATTAATTAGAAAATTACCTTTTTAGAGATTAGTAAGAGAAATATCAACCAGAATGACAGAAGAGGATTCGCTGAGATGGACTAGTTTTGCATTAGTATTATTGTAAACAGTTGTTGAAGATTATATGGTATCATTTTATGAAGATGCTAATGCATGT
??
>malaccensis
AAAAGGAGGAGTAATTCAAATTAAGATTAATAGAGATCGGGTAGCTAG---AAAAAAAATAAATAGGATAATTTAAGATCTAAATCTGCTGGGATANNNNNNGAGAATGAAAAANNNNNNNNNNNN---
NNNNNNNNNNNNNNNNNNNNNNNNAGCACAAAAAAGAAAAGAGAATCATCTGGCGAAAAATATGAATCTGCAAGAGATAAAGTTATAAGAAGATTTAGGCCAGGAGATAATGCTCTTAAATAATTAAGATAATATAATTAGACTCCATCATTGCTTATAAGAAAATTACCCTTTTAGAGGTTGATAAGAGAGATTTCTACAAGAATGACAGAAGAGGATTAGCTAAGATGGACTAGTTTTGCTCTTGTTTTACTTTAAACAGTTGTAGAAGATTATATGGTTTCCTTTTTTGAGGATGCTAATGC
TTGTGCTCTCCATGCTAAAAGAGTAACTCTAATGTAAAAAGATCTTGCCCTTGCTGCAAGAATAAGGGGATAAAAAAATGTT
>mimbres
AGCAAAAGAAGTAATTCAAACCAGCCATAGTAAAAATCTAATAAATAAATAAGCAATATTTAAGAAAATAAAAGACGATCAAAATCTGCAGGT---GGGCCAACTAAT---AAACCAAAAAATCCA---
CCTTAGTAATAAAAAAAGGTAGCCACATCAAATAAAAAGAGATAATCATCTGGTGAAAAGTATGAATCTGCTAGAGATAAGGTAATAAGAAGATTTAGACCAGGTGATAATGCTCTCAAAGAGCTCAGATAATATAATTAGAATCCTTAGTTATTAATAAGAAAATTACCTTTTTAGAGGTTAGTAAGAGAAATAACGTAAAGGATGACAGTAGAAGAATAGTTGAGATGGACAAGTTTTGCCCTAGTTATATTATAAACAATATCTGAAGATTATATGGTCTCATTTTTTGAAGATGCAAATGCTTG
TTGCTATCATGCTAAAAGAGTTACTCTTATGACAAAAGACATGGCTCTTGCTGCTAGAATTAGAGGATAAAAAAATGTT
>mobilis
AAAAGGAGAAGCAATTCTAACCAAAATTAATCAGCATCAAATAATGTT---TCAAATAATTAATCTGCAAATCAGAGATCAAAGTCAGCAGGA---CCATAAGGGAAT---AAATAAAAAAATTAG---CCTCAATAATAAAAAAAGGCTGTTTCC------
AAAAAGAGATAATCATCAGGTGAAAAATATGAATCAGCAAGAGATAAAGTAATCAGAAGATTTAGGCCAGGGGATAATGCTTTAAAAGAAATGAGATAATATAATCAAACTCCATCTCTACTAATAAGAAAATTACCTTTTTAGAGGCTAGTAAGGGAAATATCAACAAGATTGACAGAGGAAGATAACTTGAGATGGACAAGCTTTGCTTTAGTGTTATTATAAACTGTTGTTGAAGATTACATGGTATCATTTTATGAAGATGCAAATGCATGCGCTTTACATGCAAAAAGAGTTACCTTAATGAC
AAAAGATTTAGCACTAGCTGCAAGAATTAGAGGATAAAAAAATGTT
>rostrata
AAGAGGAGAAGTGTTTCTAATTAATCTCAATAAAGATCAAATAATGCAACANNNAATAATTAAGTAAATAATTAAAGATCGAAATCTGCAGGT---GGATAAATGAAT---AAATAAAAAAACCCA---
TCTTTACAGTAAAATAAGCCTGTATCAACTAAGAAAAAGAGATAATCCTCTGGTGAAAAATATGAATCAGCAAGAGATAAAGTAATTAGAAGATTTAGACCAGGAGATAATGCTTTGAAAGAACTTAAATAATATAATTAAAATCCATCACTATTTATTCGAAAACTACCTTTTTAAAGGTTAGTGAGAGAAATATCAACACGATTGTCAGATTAGGATTAAATTAGATGGACAAGTATTGCTCTAATTCTATTATAAACCGTTACAGAAGATTATATGATATCATTTTATGAAGATGCGAATGCATGCG
CTTTACATGCTAAAAGAGTTACCCTTATGAGCAAAGATATGATTCTTGCTGCTAGAATTAGAGGATAAAGGAATGTT
>silvana
AAGAGGAGGAGTAATTCAAACTAAAATCAACAAAGATCTACGAGCACA---ACTAATAATTAATCGAATTAATAGAGATCAAAATCTACTGGA---AACCAAGGTAAT---
AAATAGGTTCCTGCAACAGCTTAGAAATAAAAAAAGCCAGATACCACAAAGAAAAAGAGATAATCATCTGGTGAAAAATATGAGTCTACAAGAGATAAAGTTATTAGGAGATTTAGACCTGGTGATAATACATTAAAGGAACTTCGATAATATAATTAAGCTCCAACTCTGATGATTAGGAAACTACCTTTTTAGAAGTTAGTGAGAGAAATAGCATCTAGAATGACAGAATTAGAGCATTTGAGATGGACTAGTTAATCCTTGATGCTCTTACAATCAGTTACTGAGGATTATATGGTATCGTTTTAT
GAGGATGCTAACGCCTGT??
>thermophila
AAAAGGAGGAGTAATTCAAATCAAAATTAATAGAGATCTGATAGCCTG---AAAAAAAATAAATAGGATAATTTGAGATCTAAATCTGCTGGGAATNNNNNNGGGAATGAAAAANNNNNNNNNNNN---
NNNNNNNNNNNNNNNNNNNNNNNNAGTACAAAAAAGAAAAGGGAGTCATCAGGAGAAAAATATGAGTCTGCAAGAGATAAAGTTATTAGAAGATTTAGGCCAGGGGATAATGCCCTTAAGCAATTAAGATAATATAATTAGACTCCATCATTGCTAATAAGAAAATTACCCTTTTAGAGGTTAATAAGAGAGATTTCTACAAGAATGACAGAAGAAGATTCGCTGAGATGGACAAGTTTTGCTCTGGTTTTACTTTAAACAGTTGTAGAAGATTATATGGTTTCATTTTTTGAAGATGCGAATG
CTTGTGCTCTCCATGCTAAAAGAGTTACTCTCATGTCTAAAGATCTTGCTCTTGCGGCAAGAATTAGGGGATAAAAAAATGTT
>tropicalis
AAAAGGAGAAGCAATTCTAACCAAAATTAATCAGCATCGAATAATGTT---TAAAATAATTAATCTGCAAATCAGAGATCAAAGTCAGCAGGA---CCATAAGGGAAT---AAATAAAAAAATTAG---CCTCAATAATAAAAAAAGGCTGTCACC------
AAAAAGAGATAGTCATCAGGTGAAAAATATGAATCAGCAAGAGATAAAGTAATTAGAAGATTTAGGCCAGGGGATAATGCTTTAAAAGAAATGAGATAATATAATCAAACTCCATCTCTACTAATAAGAAAATTACCTTTTTAGAGGCTAGTAAGGGAAATATCAACAAGATTGACAGAGGAAGATAACTTGAGATGGACAAGCTTTGCCTTAGTATTATTATAAACTGTTGTTGAAGATTACATGGTATCGTTTTATGAAGATGCAAATGCATGCGCTTTACATGCAAAAAGAGTTACCTTAATGAC
AAAAGATTTAGCACTAGCTGCAAGAATTAGAGGATAAAAAAATGTT

Plasmodium – CenH3 alignment inferred in Bali-Phy. All the codons with reliability score below 80% are masked with NNN.

>Plasmodium_berghei
ATGACAAGGACAAAAAAAAGTGTAACTACACATACCCCTATAAACACA------------------------CATACGCATATGTTTAATATGCTTGCAAATAATCCAATAATA--AATAAACAGGCACACAACCAACTTGGATCTAAAGTAACAAATATGAAC--------------------------AATAAAAATAATTCAGGAAAT--ATAACACAAAAAAGTATAAATAAA---
ACTCGTATTCGAAGACCACATAGATATAGACCCGGTGTGTTAGCATTAAAAGAAATTCGAGCTTATCAATCAACAACTCAATTATTAATACCCAAAATACCATTTGTGAGGGTAGTTAAAGAAATAACAAAATTATATGAACTACCAAATAGCCAATTTCGTTATACTCCAGAAGCTTTACTAGCTCTTCAAACTGCATCAGAAGCATATTTAGTTAGTTTATTTGAAGATGCATATTTATGTTCACTTCATGCAAACAGAGTAACACTTATGCCAAAGGATATACATTTGGCTAGAAGAATAAGGGGGCG
TGAT
>Plasmodium_falciparum
ATGGTGAGAACAAAAAAGAATATACCAAATCATAACCCCTTAAATNNNTTTAATAGGGACAAGTCATTTAAA---ACAAACAAAACATTACCANNNNNNACAGTACACCATGGAATTAGTTCCAAGACTACTAATATAAACAGACCCAGTGTAAATAGAGGAGGTATA--AATGAANNNNNNCAAAAAAACTTGCACAGG---
NNNNNNATAAGGAAACCCCATAGATACAGGCCAGGTGTATTAGCATTAAAAGAAATAAGAGCATATCAAGCATCGACTCAATTATTAATACCTAAAATTCCATTTGTTCGAGTAGTAAAAGAAATTACAAGATTATTTGAATTACCAGATGAGCAATTTCGTTATACACCTGAAGCATTATTAGCTTTACAAACAGCATCAGAAGCATATTTGGTTAGTTTATTTGAGGATGCTTATTTATGTTCATTACATGCAAACAGAGTAACACTTATGCCCAAAGATATTCATTTAGCTAGAAGAATACGCGGAA
GAGAC
>Plasmodium_fragile
ATGGTGCGAACGAAAAAGAGCGTGCCCATGCACAACCCCTTGNNN--AACCCAGACGGGGTGGGCAGCAGCAACGAACCGACGAACAAGACAGTGCCTNNNNNNCCATCACACAAGNNNNNN--
GCCTCGTCCAAATCAAATAACAACGCAGGAACAGGCAAAGGGACTCGANNNNNNNNNNNNNCAAAAGGGTTTGAAGAAGACANNNCGAATTAGGAGGCCCCATAGGTACAGACCAGGTGTATTAGCATTAAAGGAAATAAGAGCATACCAAGCGACTACACAGTTACTCATTCCTAAAATACCTTTTGTTCGAGTCGTAAAGGAGATTACTCGGTTGTTTGAATTGCCCAATGAACAACTGCGTTACACCCCCGAGGCCCTGTTGGCACTGCAAACCGCATCGGAGGCCTACCTGGTCAGTCTCTT
CGAAGATGCCTACTTGTGTTCACTTCACGCAAACCGCGTCACCCTCATGCCTAAGGACATACACTTGGCTCGCCGTATCCGCGGCCGCGAC
>Plasmodium_chabaudi
ATGGCGAGGACAAAAAAGAGTGTAACTAACCATACCCCTATCAACACA------------------------CATACGCATATGTTTAATATGCTCGCAAATAATCCAATGGTA--AATAAACCAGCACATAACCAAATTGGATCCAAACCAACAAATGTAAAT--------------------------AATAAAAGTAATTCAGGAAAT--ATGACACAAAAAGGTTTAAATAAA---
AATCGTATTCGAAGACCACATAGATATAGACCTGGTGTATTAGCATTAAAAGAAATTCGAGCTTATCAAGCAACAACTCAATTATTAATACCTAAAATACCATTTGTAAGAGTAGTTAAAGAAATAACAAAATTATATGAATTGCCAGATAGCCAATTTCGTTATACTCCAGAAGCTTTACTAGCTCTTCAAACTGCTTCGGAAGCATATTTAGTTAGCTTATTTGAAGATGCCTATTTATGTTCGCTACATGCAAACAGAGTGACCCTCATGCCTAAGGACATACATTTAGCTAGACGAATAAGAGGCCG
AGAT
>Plasmodium_reichenowi
ATGGTGAGAACAAAAAAGAATATACCAAATCATAACCCCTTAAATNNNTTTAATAAGGACAAGTCATTTAAA---ACAAACAAAACATTACCANNNNNNGCAGTACACCATGGAATTAGTTCCAAGACTACTAATATAAACAGACCAAGTGTAAATAGAGGAGGTATA--AATCAANNNNNNCAAAAAAACTTGCACAGG---
NNNNNNATAAGGAAACCCCATAGATACAGACCAGGTGTATTAGCATTAAAAGAAATAAGAGCATATCAAGCATCGACTCAATTATTAATACCTAAAATTCCATTTGTTCGAGTAGTAAAAGAAATTACAAGATTATTTGAATTACCAGATGAGCAATTTCGTTATACACCGGAAGCATTATTAGCTTTACAAACAGCATCAGAAGCTTATTTGGTTAGTTTATTTGAGGATGCTTATTTATGTTCATTACATGCAAACAGAGTAACACTTATGCCCAAAGATATTCATTTAGCTAGAAGAATACGCGGAA
GAGAC
>Plasmodium_vinckei
ATGGCGAGGACAAAAAAGAGTGTAACTAACCATACCCCTATAAACACA------------------------CATACGCATATGTTTAATATGCTCGCAAATAATCCAATGGTA--AATAAATCAACACATAACCAAATTGGGTCTAAAGGAACAAATATAAAT--------------------------AATAAAAGTAATTCAGGGAAT--ATGACACAAAAAGGTTTAAATAAA---
AGTCGTATTAGAAGACCACATAGATATAGACCTGGTGTATTAGCATTAAAAGAAATTCGAGCTTATCAAGCAACAACTCAATTATTAATACCTAAAATACCATTTGTAAGAGTAGTTAAAGAAATAACAAAATTATATGAATTACCAGATAGCCAATTTCGTTATACTCCTGAAGCTTTATTAGCTCTTCAAACGGCTTCGGAAGCATATTTAGTTAGCTTATTTGAAGATGCATATTTATGTTCACTACATGCAAACAGAGTGACTCTCATGCCTAAGGACATACATTTAGCTAGACGAATAAGAGGGAG
AGAT
>Plasmodium_yoelii
ATGGTGAGAACAAAAAAAAGCGTAACTACACATACCCCTATAAATACA------------------------CATACGCATATGTTTAATATGCTTGCAAATAATCCAATGATA--AATAAATCAGCAAATAACCAAATTGGGTCAAAATCAATA-------------------------------------NNNAAAAATAATTCAGGAAAT--ATAACACACAAAAATATAAATAAA---
AATCGTATCCGAAGACCACATAGATATCGACCTGGTGTATTAGCATTAAAAGAAATTAGAGCTTATCAAGCAACAACTCAATTATTAATACCCAAAATACCATTTGTAAGAGTAATTAAAGAAATAACAAAATTATATGAATTGCCAAATAATCAATTTCGTTATACTCCCGAAGCTTTATTAGCTCTTCAAACTGCATCAGAAGCATATTTAGTTAGCTTATTCGAAGATGCATATTTATGTTCTCTTCATGCAAATAGAGTAACACTTATGCCAAAAGATATACATTTAGCTAGACGGATAAGAGGCCGT
GAT
