# Supplementary material for: CenH3 evolution reflects meiotic symmetry as predicted by the centromere drive model
Source: Sci Rep. 2016 Sep 15;6:33308. doi: 10.1038/srep33308 (PMC5024113; doi:10.1038/srep33308)
Supplement: Supplementary File S3 [file srep33308-s3.pdf]

CenH3 evolution reflects meiotic symmetry as predicted by the centromere drive model

František Zedek, Petr Bureš

Supplementary File S3 – The alignments and phylogenetic trees that were used for selection analyses in nexus format

Aspergillus

```
#NEXUS
[ Title ]
begin taxa;
     dimensions ntax=18;
     taxlabels
          Aspergillus_acidus
          Aspergillus_aculeatus
          Aspergillus_brasiliensis
          Aspergillus_clavatus
          Aspergillus_flavus
          Aspergillus_fumigatus
          Aspergillus_glaucus
          Aspergillus_kawachii
          Aspergillus_nidulans
          Aspergillus_niger
          Aspergillus_sojae
          Aspergillus_sydowii
          Aspergillus_terreus
          Aspergillus_tubingensis
          Aspergillus_versicolor
          Aspergillus_wentii
          Aspergillus_zonatus
          Neosartorya_fischeri
;
end;
begin characters;
     dimensions nchar=969;
     format missing=? gap=- matchchar=. datatype=nucleotide;
     matrix

Aspergillus_acidus
ATGCCCCCCAAGACA???GGTCGCGGT??????CGCAAG??????ACGGCGGCG------
------------------CCGCGA-----------------------------------
------CAATCAACAGCT-----------------------------------------
------------------GGCGACCGTGCT-----------------------------
???GCAAGCTCC------TCCACA------------------GCA---GCTACAACG---
---------------------GCCTCGCCG-----------------------------
---------------------------GCG-----------------------------
---------------------GCCAAGAAAGTG---CGCAAATCCACT---GGA---GCG
------------ACTAGG------------------GGAGGGAAAAGGCCTGCTGGAAAG
GCACCC---------------------------------------AGAGAG---TCCGAT
GTT------------------CAGCCTGGTGACCCTACACCACAAGGACGTGTACGCCGC
TACAAGCCCGGAACCGTGGCTCTCAAGGAGATCCGCAAGTACCAACGCTCGTACGATCTG
CTTATCCAAAAGCTTCCTTTCGCTCGGCTGGTCCGCGAGGTCGCGCTTGACCTCCTCCCT
TCAGAAGTTGGCGCCGAGCTGCGGTGGCAGTCGCATGCGATCCAAGCGCTCCAGGAAGCA
GCCGAAGCTTTCCTTGTACATCTCTTCGAGGACACCAACCTCTGCGCGATTCACGCCAAG
CGCGTGACCATCATGCAGAAAGATATCCAACTTGCTCGACGCATCCGTGGTGTCTGGGGT
GGTTTGGGC

Aspergillus_aculeatus
ATGCCGCCTAAGACAGGG---------CGCGGTCGCAAGTCAATT---GCTGGG------
------------------CCACGG-----------------------------------
------AAA?????????------GCCGCCGCCGCCGCCGCCGCCACTACTGCTGCTGCT
ACT---------------???GATGGT???---------------GCC---------GCT
GCTGCCGGCCCG------TCAACT------------------GCC---CCAGCTACG???
?????????------------------CCC-----------------------------
---------------------------GCC-----------------------------
---------------------GCTCGC---???---CGTAAACCTGCG---GGT---GTC
------------ACCAGA---------------------???AAGCGACCGGCTAAA---
GCGCCC---------------------------------------AGGACA---TCCAAT
GTT------------------GAACCGGGCGATCCCACCCCCACAGGTCGTCGTCGCCGG
TACAAACCTGGAACGGTTGCCCTGAAGGAGATCCGCCATTACCAACGCTCATTCGACCTG
CTCATCCGAAAGCTGCCATTCGCGCGACTGGTCCGCGAGGTTGCCTTGGATCTTCTCCCC
GCTGAAGTCGGCTCCGAGCTGCGCTGGCAATCGCATGCGATCTTGGCCCTCCAGGAAGCC
GCCGAGGCGTTCCTGGTCCATCTTTTCGAGGACACCAATCTCTGTGCCATTCATGCCAAG
CGCGTGACCATCATGCAAAAGGATATCCAGCTTGCTCGACGCATCCGGGGTGTCTGGGGT
GGTCTGGGT

Aspergillus_brasiliensis
ATGCCCCCCAAGACA??????------??????CGCAAG??????GTGGCGGCG------
------------------CCGCGA-----------------------------------
------CAATCAACAGCT-----------------------------------------
------------------GGCGACCGTGCT-----------------------------
???GCAAGCTCC------TCGAAA------------------GCA---GCTGCAACG---
---------------------ACATCGCCG-----------------------------
---------------------------GCG-----------------------------
---------------------GCGAAGAAAGTG---CGCAAATCCACT---GGA---CCG
------------GCTAGG------------------GGAGGGAAAAGACCTGCTGGAAAG
GCGCCC---------------------------------------AGACAG---TCCGAT
GTT------------------CAGCCTGGTGACCCCACGCCACAAGGACGTGTGCGCCGC
TACAAGCCCGGAACTGTGGCTCTTAAAGAGATCCGCAAGTACCAGCGCTCGTACGATCTG
CTTATTCAAAAGCTTCCTTTTGCTCGGCTGGTCCGCGAGGTCGCGCTTGACCTCCTCCCT
TCAGAAGTTGGCGCTGAGCTGCGGTGGCAGTCACATGCGATCCAAGCGCTTCAGGAAGCA
GCCGAAGCTTTCCTCGTACATCTCTTTGAGGATACCAATCTCTGTGCTATCCACGCCAAG
CGCGTGACCATCATGCAGAAAGATATCCAACTTGCTCGACGCATCCGTGGTGTCTGGGGT
GGTTTGGGC

Aspergillus_clavatus
ATGCCACCAAAGACAGGA---------CGGGGCCGGAAGGCAGTC---??????GGAAGA
------------------??????------------AAA--------------------
-----------------------------------------------------------
------------------???---------GAA--------------------------
---???GGCCCC------TCAGAC------------------GCA---CCTGCGACT---
---------------------GCATCACCT-----------------------------
---------------------------TCG-----------------------------
---------------------ACCCGAGGCCAG---CGCAAGTCAAGC---GGA---GCG
------------ACAAGG------------------GGGGGCAAGCGACCGGCCGGC---
GCACCG---------------------------------------AGAAAA---TCAGAC
GTT------------------CAACCTGGAGACCCAACCCCTCAAGGTCGCCCACGCCGA
TACCGACCGGGAACGGTTGCCCTGAAAGAGATCCGCAAATATCAGCGCTCGTACGATCTG
CTCATCCAAAAGTTACCCTTTGCGCGACTCGTCCGCGAGGTCGCCTTGGACCTCCTCCCC
ACCGAGGTCGGCGCCCAATTGCGATGGCAGTCGCATGCAATCCAAGCGCTCCAGGAAGCT
GCTGAAGCTTTCCTGGTCCATCTGTTCGAGGACACCAATCTCTGCGCCATCCATGCTAAG
CGCGTAACAATCATGCAGAAAGATATTCAGCTTGCTCGAAGAATTCGCGGTGCTTGGGGT
GGCCTGGGT

Aspergillus_flavus
ATGCCACCGAAGACAGGA---------GGAGGTCGCAAGGTAATC---GCCGCA------
------------------TCGCGA-----------------------------------
------------------GCAAAG-----------------------------------
------------------???GACGGT???---------------???AAAGAAAGC???
---------------------------------------------??????AAA---
---------------------GGGTCCCCT-----------------------------
---------------------------TCG-----------------------------
---------------------ACTCGAGGA------CGCAAGTCCGCT---GGA---GGT
------------GCCAGA------------------GCGGGGAAGCGACCGGCTGGT---
GCATCG---------------------------------------AGGAAA---TCAGAT
GTC------------------CAACCTGGCGATCCTACCCCTCAGGGCCGCCATCGCCGT
TACAGACCGGGCACTGTTGCCCTCAAAGAAATCCGCAAATACCAACGTTCTTATGACCTC
CTTATACGAAAGCTCCCATTCGCAAGACTTGTGCGCGAAGTTGCATTGGATCTACTCCCC
GCAGATGTAGGATCCGAATTGCGCTGGCAGTCGCAAGCCATCATGGCGCTGCAGGAGGCC
GCGGAAGCCTTTCTTGTTCATCTATTCGAGGACACGAACCTGTGTGCTCTACACGCGAAG
CGAGTGACTATCATGCAGAAAGATATCCAGCTTGCTCGGAGAATCCGTGGCGCTTGGGGT
GGATTAGGC

Aspergillus_fumigatus
ATGCCGCCGAAGACTGGA---------CGAGGACGTAAAGCAATT---GGGGCT------
------------------CCTCGAACCCAACCTCGA-----------------------
-----------------------------------------------------------
------------------????????????-----------------------------
---???GGTCCA------TCTAAC------------------GCT---GCGGCTACA---
---------------------GCATCACCT-----------------------------
---------------------------TCT-----------------------------
---------------------ACTCGGGGA???CCGCCCAAATCTCGC---GGG---GGA
------------ACAAGG------------------GGGGGAAAGCGACCAGCCCAC---
GCCCCG---------------------------------------AGGCCG---TCAGAC
GTC------------------CAACCTGGGGACCCAACCCCTCAAGGTCGCCGACACCGG
TACAAGCCGGGAACCGTCGCATTGAAAGAGATCCGCAGATACCAGCGCTCATATGACCTC
CTTATTCAGAAGCTACCCTTTGCGCGACTCGTACGCGAGGTCGCATTGGAACTTCTTCCC
GCCGAAGTAGGCGCGGAGCTACGGTGGCAGTCGCACGCTATTCAGGCGCTACAGGAGGCC
GCCGAAGCTTTCCTCGTCCACCTTTTCGAGGATACCAATCTCTGTGCTTTGCATGCTAAG
CGTGTAACGATTATGCAGAAAGATATCCAGCTCGCCCGAAGAATACGCGGTATTTGGGGC
GGTCTGGGT

Aspergillus_glaucus
ATGCCACCAAAGACAGGA---------AAAGGCCGTAAAGTCGTC---TCAGAA------
------------------CCGCGA-----------------------------------
-----------------------------------------------------------
---TCGCGCCCCAGCACC????????????-----------------------------
---GCAGGCCCA------TCA---TCAAAACCGCAACAA---------------ACC---
---------------------GCGTCGCCA-----------------------------
---------------------------???AACAAAAGGAAACCCAAGGCTTCC------
---------------------???------------------------TCC???GAA???
AGA------------------------------------???AAGCGGCCATCGGGA---
??????AAAACA??????------------GCAGCAAAGACAGCA??????---TCAGAC
GTC------------------CAACCCGGCGACCCAACCCCCCAAGGCCGTTCACGCCGC
TACAAACCCGGAACCGTCGCTCTAAAAGAAATCCGCAAATACCAACGCTCCTTCGACCTC
CTCATCCAAAAGCTCCCCTTCGCGCGCCTCGTCCGTGAAGTCGCCCTGGAACTGCTCCCC
GCAGACGTAGGCGAACAGCTCCGGTGGCAGTCGCATGCGATCCAAGCACTACAAGAAGCC
GCAGAGGCATTCCTCGTCCATTTATTCGAAGATACTAATTTGTGTGCTCTGCATGCTAAG
CGCGTGACTATTATGCAAAAGGATATCCAGCTTGCACGGAGGATTCGTGGTGCTTGGGGT
GGATTGGGA

Aspergillus_kawachii
ATGCCCCCCAAGACA???GGTCGCGGT??????CGCAAG??????ACGGCGGCG------
------------------CCGCGA-----------------------------------
------CAATCAACAGCT-----------------------------------------
------------------GGCGACCGTGCT-----------------------------
???GCAAGCTCC------TCAACA------------------GCA---GCTATAACG---
---------------------GCCTCGCCG-----------------------------
---------------------------GCG-----------------------------
---------------------GCCAAGAAAGTG---CGCAAATCCACT---GGA---GCG
------------ACTAGG------------------GGAGGGAAAAGGCCTGCTGGAAAG
GCACCC---------------------------------------AGGGAG---TCCGAT
GTT------------------CAGCCTGGTGACCCTACACCACAAGGACGTGTACGCCGC
TACAAGCCCGGAACCGTGGCTCTCAAGGAGATCCGCAAGTACCAACGCTCGTACGATCTG
CTTATCCAAAAGCTTCCTTTCGCTCGGCTGGTCCGCGAGGTCGCGCTTGACCTCCTCCCT
TCAGAAGTTGGCGCCGAGCTGCGGTGGCAGTCGCATGCGATCCAAGCGCTCCAGGAAGCA
GCCGAAGCTTTCCTTGTACATCTCTTCGAGGACACCAACCTCTGCGCGATTCACGCCAAG
CGCGTGACCATCATGCAGAAAGATATCCAACTTGCTCGACGCATCCGTGGTGTCTGGGGT
GGTTTGGGC

Aspergillus_nidulans
ATGCCCCCAAAA------------------?????????--------------------
CCATCCTCG-------------------------------------------------
-----------------------------------------------------------
--------------------------------------------???---------???
???GCAGGT???GGAAGC??????---------------GCG???---?????????ACA
ACCTCCACCACAACCTCAGGC?????????AAGGTAACTAAGAGCTCCAGAACATTAGGG
TCAAAGGCATTGGCATCGGCATCGGTG???-----------------------------
---------------------------------------AAATCT---------------
---------------AAG------------------GGAAGTAAGCGACCA---------
---CCG---------------------------------------ACG???CCATCA???
???------CAATCCAGCATC???CCCGGCGACCCAACCCCCAAAGGCCGTCGTCACCGG
TACAAACCTGGTACCGTTGCCCTCAAAGAAATCCGCAAGTACCAACGCTCCTACGATCTG
CTTCTCCGAAAATTGCCATTTGCACGTCTCGTCCGCGAAGTTGCTCTCGACATCCTCCCC
GCAGACGTTGGCTCCGAGCTGCGATGGCAGTCGCATGCAATCCAGGCGCTCCAGGAAGCC
GCTGAAGCCTTTTTGGTACACTTGTTCGAGGACACGAATCTTTGCGCCATCCACGCGAAA
CGAGTTACGATAATGCAGAAGGATATCCAGCTTGCGCGTAGGATTCGCGGAGCTTGGGGT
GGTCTTGGC

Aspergillus_niger
ATGCCTCCCAAGACA??????------???GGTCGCAAG??????GTGGCGGCG------
------------------CCGCGA-----------------------------------
------CAATCAACAGCT-----------------------------------------
------------------GGCGACCGTGCT-----------------------------
???GCAAGCTCG------TCGAAA------------------GCA---GCTGCAACG---
---------------------ACGTCGCCG-----------------------------
---------------------------GCA-----------------------------
---------------------GCTAAAAAAGTG---CGCAAATCCACT---GGA---CCG
------------GCTAGG------------------GGAGGGAAAAGGCCTGCTGGAAAG
GCACCC---------------------------------------AGAGAG---TCCGAC
GTT------------------CAGCCTGGTGATCCCACACCACAAGGACGTGTACGCCGC
TACAAGCCTGGAACCGTGGCTCTCAAAGAGATCCGCAAGTACCAGCGCTCTTATGATCTG
CTTATCCAAAAGCTTCCTTTTGCTCGGCTGGTCCGCGAGGTCGCTCTTGACCTCCTCCCT
TCAGAAGTTGGCGCCGAGCTGCGGTGGCAGTCGCATGCGATCCAAGCGCTCCAAGAAGCA
GCCGAAGCTTTCCTTGTACATCTCTTCGAGGACACCAACCTCTGTGCTATTCACGCCAAG
CGCGTGACCATCATGCAGAAAGATATCCAACTTGCTCGACGCATTCGTGGTGTCTGGGGT
GGTTTGGGC

Aspergillus_sojae
ATGCCACCGAAGACAGGA---------AGAGGTCGCAAGGTAATC---GCCGCA------
------------------TCGCGA-----------------------------------
------------------GCAAAG-----------------------------------
------------------???GACGGT???---------------???AAAGAAAGCGCA
GCTGCAGGCCCA------TCCGGT------------------GCA---GCTGCAAAA---
---------------------GACTCCCCT-----------------------------
---------------------------TCG-----------------------------
---------------------ACTCGAGGA------CGCAAGTCCACT---GGA---GGT
------------GCCAGA------------------GCGGGGAAGCGACCGGCTGGT---
GCATCG---------------------------------------AGGAAA---TCAGAT
GTC------------------CAACGTGGCGATCCTACCCCTAAGGGCCGCCATCGCCGT
TACAGACCGGGCACTGTTGCCCTCAAAGAAATCCGCAAATACCAACGTTCTTATGACCTC
CTTTTACGGAAGCTCCCATTCGCAAGACTTGTGCGCGAAGTTGCATTGGATCTACTCCCC
GCAGATGTAGGATCCGAATTGCGCTGGCAATCGCAAGCCATCATGGCGCTGCAAGAGGCC
GCGGAGGCCTTCCTTGTTCATCTATTCGAGGACACGAACCTGTGTGCTCTACACGCGAAG
CGAGTGACTATCATGCAGAAGGATATCCAGCTTGCTCGGAGAATCCGTGGCGCTTGGGGT
GGACTGGGC

Aspergillus_sydowii
ATGCCTCCGAAA------------------?????????--------------------
CCAGCCTCG-------------------------------------------------
-----------------------------------------------------------
-------------------------------------------------------???
ACCGCCGGGCCC------TCG-----------------------------------???
?????????ACAACAACAGGC??????CCC-----------------------------
---------------------------GCT-----------------------------
---------------------------------------AAATCCGCC---GGA---???
---AAAAAAGGT??????GGAGTCCAGGGAACCACGGGGGGGAAGCGACCA---------
---CCA---------------------------------------AGGACA---TCA???
???------CAATCAGATGTT???CCCGGAGACCCAACCCCCAAAGGCCGCCGTCACCGG
TACAAGCCCGGCACCGTCGCCCTGAAGGAAATCCGCAAGTACCAGCGCTCATACGATCTC
CTCCTCCGAAAACTTCCCTTTGCACGATTAGTGCGCGAGGTCGCATTGGACCTTCTCCCC
GCAGAAGTCGGCGCGGAGCTGCGGTGGCAGTCGCATGCAATCCTTGCGCTCCAAGAGGCT
GCCGAGGCCTTCCTAGTACACTTGTTCGAGGACACCAATCTATGCGCCATCCACGCGAAA
CGGGTTACGATCATGCAGAAGGATATCCAGCTTGCGCGCAGAATTCGTGGGGCGTGGGGT
GGTCTAGGC

Aspergillus_terreus
ATGCCACCCAAAACAGGG---------CGAGGCCACAAGATCACC---GCCGCG------
------------------CCCCGG---------------ACAGAGGCAAGCAGCAGTCGT
ACCACC-----------------------------------------------------
--------------------------------------------GCC---------GCC
GCCGCCGGCCCG------TCG--------------------------------???---
---------------------GCGTCGCCG-----------------------------
---------------------------TCC-----------------------------
---------------------GCCCACAGGGTG---CGCAAATCCACC---GGA---CCA
------------TCCAGA------------------GGCGGGAAGCGACCGGCCGGC---
GCGCCG---------------------------------------AGAGAC---TCCGAC
GTT------------------CAACCCGGCGATCCCACCCCGACCGGTCGTCGTCGCCGC
TACCGACCTGGCACCGTCGCCCTCAAGGAAATCCGCAAGTACCAACGGTCATATGACCTC
CTCATTCAGAAGCTCCCCTTCGCGCGACTCGTCCGTGAAGTCGCACTCGATATCCTCCCC
GCCGAGCGCGGCGCCGAGCTCCGTTGGCAGTCGCATGCCATCCAGGCCCTCCAGGAGGCC
GCCGAGGCGTTCCTGGTGCACCTGTTCGAGGACACGAACTTGTGCGCCCTGCACGCGAAA
CGAGTCACCATCATGCAGAAGGATATCCAGCTTGCGCGACGAATTCGCGGTGTGTGGGGT
GGTTTGGGC

Aspergillus_tubingensis
ATGCCCCCCAAGACA???GGTCGCGGT??????CGCAAG??????ACGGCGGCG------
------------------CCGCGA-----------------------------------
------CAATCAACAGCT-----------------------------------------
------------------GGCGACCGTGCT-----------------------------
???GCAAGCTCC------TCAACA------------------GCA---GCTACAACG---
---------------------GCATCGCCG-----------------------------
---------------------------GCG-----------------------------
---------------------GCCAAGAAAGTG---CGCAAATCCACT---GGA---GCG
------------ACTAGG------------------GGTGGGAAAAGGCCTGCTGGAAAG
GCACCC---------------------------------------AGAGAG---TCCGAT
GTT------------------CAGCCTGGTGACCCCACACCACAAGGACGTGTACGCCGC
TACAAGCCCGGAACCGTGGCTCTCAAGGAGATCCGCAAGTACCAGCGCTCGTACGATTTG
CTTATCCAAAAGCTTCCTTTCGCTCGGCTGGTCCGCGAGGTCGCGCTTGACCTCCTCCCT
TCAGAAGTTGGCGCCGAGCTGCGGTGGCAGTCGCATGCGATCCAAGCGCTCCAGGAAGCA
GCCGAAGCTTTCCTTGTACATCTCTTCGAGGACACCAACCTCTGTGCGATTCACGCCAAG
CGCGTGACCATCATGCAGAAAGATATCCAACTTGCTCGACGCATCCGTGGTGTCTGGGGT
GGTTTGGGC

Aspergillus_versicolor
ATGCCTCCGAAA------------------?????????--------------------
CCGGCCTCG-------------------------------------------------
-----------------------------------------------------------
-------------------------------------------------------???
ACAGCCGGACCC------TCGAAA------------------ACG---CCCGCA------
---------------------??????CCC-----------------------------
---------------------------TCC-----------------------------
---------------------------------------AAATCCGCC---GGA---???
---AAAAAAGGT??????------------------GGGGGGAAGCGA???---------
---------------------------------------------AGGACA---TCA???
???------CAACCAGATGTT???CCCGGAGACCCAACCCCCAAAGGCCGCCGCCACCGG
TACAAGCCCGGCACCGTCGCCCTCAAGGAAATCCGCAAGTACCAACGCTCATATGATCTT
CTCCTCCGAAAACTCCCCTTCGCACGATTAGTGCGCGAGGTCGCATTGGACCTCCTCCCC
GCAGAAGTCGGCGCGGAGCTACGGTGGCAGTCGCATGCAATCCTTGCGCTCCAAGAAGCC
GCCGAGGCCTTCCTGGTACATTTGTTCGAAGACACGAATTTATGCGCCATCCACGCGAAA
CGGGTTACGATCATGCAGAAGGATATCCAACTTGCGCGTAGAATTCGCGGGGCGTGGGGT
GGTCTAGGC

Aspergillus_wentii
ATGCCACCAAAGACCGGT---------CGAGGCAATAAGATTATC---GCTGCT------
------------------CCCAGA-----------------------------------
------------------GCAAAG-----------------------------------
---------------------------???---------------???AAGGAAGAGGCG
GAGGCAGGTCCA------TCAAGA------------------GCAAGCCCTGCGACC---
---------------------GCGTCGCCA-----------------------------
---------------------------GCA-----------------------------
---------------------ACCCGAGGCCGA---CGCAAGTCCGCT---GGT---GCA
------------AGCAGA------------------GGAGGAAAGAAACCCGCTGGA---
??????AAA---??????GCAAAACCATCA---------------??????---???GAT
GTC------------------CAGCCCGGAGACCCTACCCCTCAAGGTCGTCGTCATCGA
TACAAGCCAGGAACCGTTGCCCTGAAAGAGATCCGTAAATACCAGCGCTCCTATGATCTG
CTGATCCAGAAGCTTCCCTTTGCGCGACTCGTGCGTGAGGTTGCGTTGGAGCTTCTCCCC
GCCGACGTGGGAGCAGAGCTGCGATGGCAGTCACATGCGATCCAGGCCCTGCAGGAAGCT
GCAGAGGCTTTCCTTGTCCATCTCTTCGAGGATACAAACCTGTGCGCTCTACACGCTAAG
CGTGTTACCATCATGCAGAAGGATATTCAGCTTGCCCGAAGAATCCGCGGTGCCTGGGGT
GGATTGGGT

Aspergillus_zonatus
ATGCCGCCTAAGACGGGG---------CGAGGCCGCAAGATGCTT---GCGGCG------
---------CAAGCGCGCCCACGG-----------------------------------
------????????????-----------------------------------------
------------------????????????---GACGGTGATGGG???---------GCT
GCCGCGGGTCCG------TCG--------------------------------------
-----------------------------------------------------------
---------------------------------------------------TCGCGC
AACGATGGCGCAACGAAACCC???CGAGGAGGT---CGCAAGTCG------???---???
------------ACGAGG------------------GGAGGGAAACGTCCC---------
---CCT---------------------------------------GGG???---------
---AGCGGG???TCGAACATC???CCGGGCGATCCCACCCCGAAAGGCCGTCGTCACCGA
TACAAGCCAGGCACCGTCGCCCTGAAGGAAATCCGAAAGTACCAGCGCTCATATGACCTA
CTGTTGCGCAAGCTGCCCTTTGCACGGCTTGTCCGCGAGGTCGCCCTGGAGCTATTACCG
GCCAACGTGGGCGCCGAATTGCGCTGGCAGTCGCACGCGATCCAGGCTCTGCAGGAAGCC
GCCGAGGCCTTCCTGGTCCATCTCTTCGAAGACACCAATCTGTGCGCCCTGCACGCCAAG
CGCGTTACCATAATGCAAAAGGATATCCAGCTAGCGCGGAGAATACGTGGTGTATGGGGC
GGCCTGGGT

Neosartorya_fischeri
ATGCCGCCGAAGACTGGA---------CGAGGACGTAAAGCAATT---GGGGCT------
------------------CCTCGAACGCAACCTCGA-----------------------
-----------------------------------------------------------
------------------????????????-----------------------------
---???GGTCCA------TCTAAT------------------GCT---GCGGCCACT---
---------------------GCATCACCT-----------------------------
---------------------------TCC-----------------------------
---------------------ACTCGGGGA???CCGCCCAAGTCCCGC---GGA---GGA
------------ACAAGG------------------GGGGCCAAGCGACCAGCCCGC---
GCCCCA---------------------------------------AGGCCG---TCAGAC
GTC------------------CAACCTGGGGACCCAACCCCTCAAGGTCGCCGACACCGG
TACAAGCCGGGCACAGTCGCATTGAAAGAGATCCGCAAATACCAGCGCTCATATGACCTC
CTTATTCAGAAGCTACCGTTTGCGCGACTCGTACGCGAGGTCGCATTGGAACTTCTCCCC
GCTGAAGTAGGCGCGGAGCTACGGTGGCAGTCGCACGCTATTCAGGCGCTACAGGAGGCC
GCCGAAGCTTTCCTCGTCCATCTTTTCGAGGATACCAATCTCTGTGCTTTGCATGCTAAG
CGTGTAACGATTATGCAGAAAGATATCCAGCTCGCCCGAAGAATACGCGGTGTTTGGGGC
GGTCTGGGA

;
end;

BEGIN TREES;
          TREE tree = (Aspergillus_acidus,Aspergillus_kawachii,(Aspergillus_tubingensis,(Aspergillus_brasiliensis,(Aspergillus_niger,(Aspergillus_terreus,(((Aspergillus_clavatus,(Aspergillus_fumigatus,Neosartorya_fischeri)),((Aspergillus_glaucus,Aspergillus_wentii),(Aspergillus_flavus,Aspergillus_sojae))),(Aspergillus_aculeatus,(Aspergillus_zonatus,(Aspergillus_nidulans,(Aspergillus_sydowii,Aspergillus_versicolor))))))))));
END;
```

Asteraceae

```
#NEXUS
[ Title ]
begin taxa;
     dimensions ntax=7;
     taxlabels
          Carthamus_tinctorius
          Cichorium_intybus
          Flaveria_vaginata
          Helianthus_annuus
          Helianthus_exilis_CHES4473
          Lactuca_sativa
          Taraxacum_koksaghyz
;
end;
begin characters;
     dimensions nchar=459;
     format missing=? gap=- matchchar=. datatype=nucleotide;
     matrix

Carthamus_tinctorius
ATGGCGAGAACCAAACAACCTGCTAAACGCAGTTCTGGCAAGCGT---GAT------GCT
AGACCATCTACC------TCCACGCCTACGCCAAGGCCAAGTGCAAGGAAGAATCCC---
GAGAGCTCAGGAGCTGGGGATGGTCAG------AGGCGCCATCGTTATAGGCCTGGGACT
CAGGCGCTTCGTGAGATTAGGCGCTTGCAGAAGACTGTCAATCTTCTCATTCCGGCTGCT
CCTTTCATTCGAACCGTAAAGGAGATAAGCAACTACATTGCGCCAGAAGTCACTCGCTGG
CAAGCTGAAGCTCTACAAGCCCTTCAAGAGGCAGCAGAAGATTACTTAATTCAGTTGTTT
GAAGATTCAATGCTATGCGCGATTCATGCAAAACGCGTTACCCTCATGAAAAAGGATTGG
GAGTTGGCACGGCGGCTTGGGAAGAAAGGGCAACCGTGG

Cichorium_intybus
ATGGCGAGAACAAAGCAACCTGCTAAACGGAGTTGGGGCAATCGAAAGTCTAGTCAATCT
CGAGCATCA???TCAACT??????AGTACGCCACGAAAAAGTCCGAGAAAGGATCCA---
GGG------???ACTGGAGAAAGG???CAACAG???CCCCATCGTTTCAAGCCTGGGGCT
CAAGCACTTCGTGAAATTCGTCGTCTTCAGAAGACTGTTAATCTTCTCATTCCAGCTGCC
CCATTCATTCGAACTGTAAAAGAAATAAGCAACTACATTGCCCCTGAAGTTACACGCTGG
CAAGCTGAAGCTATACAGGCCCTTCAAGAGGCAGCAGAAGATTACTTAGTTCAGTTGTTT
GAAGATTCAATGCTATGTTCGATTCATGCAAAGCGTGTTACCCTCATGAAAAAGGACTGG
GAATTGGCACGGAGACTTACAAAGAAAGGTCAACCATGG

Flaveria_vaginata
ATGGCGAGAACCAAACACCCTGCTAAACGAAGTTGGGGCACTAGAAAAGAT------GGT
AGAGCATCTACT------TCAACGAGCACTCCGAGGAAAAGCCCTAGGAAGGATGCA---
GGGGGTTCTGGAACTGGAGAAAAGCAG------AGGCCCCATCGGTTTAAGCCTGGGACT
CAGGCACTTCGGGAGATTAGGCGTTTGCAAAAGACGGTCAATCTTCTCATTCCTGCTGCT
CCCTTCATTCGAACTGTGAGGGAGATAAGCAACTACCTGGCCCCTGAAATCACTCGCTGG
CAAGCTGAAGCTTTACAAGCCCTTCAAGAGGCAGCAGAAGATTACATAATTCAGTTGTTT
GAAGATTCAATGCTATGCGCAATTCATGCAAAACGTGTTACCCTTATGAAAAAGGATTGG
GAGTTGGCACGGCGTCTCGGGAAGAAAGGGCAGCCATGG

Helianthus_annuus
ATGGCGAGACCCAAACCACCTGCTAAACGCAGTTCAGGCATACCAGCAGAC------GGT
AGATCATCC?CT------TCACCAAACACACCAAGAAAGAGTCCGAGGAAGAATCGAGTA
GGT------------GGAGAAAACAGG------AAGCCGCATAGGTTTAAGCCTGGGACA
CAGGCGCTACGTGAGATTAGGCGTTTGCAGAAGACGGTTGAACTGATCATTCCGGCTGCT
CCGTTTATTCGAACTGTAAAGGAGATAAGCAACTACATGGCCCCTGAAATCACTCGCTGG
CAAGCCGAAGCTCTACAAGCCCTTCAAGAGGCAGCAGAAGATTACCTAATTCAGTTGTTT
GAAGACTCAATGCTATGTGCGATTCATGCAAAGCGCGTTACCCTCATGAAAAAGGATTGG
GAGTTGGCACGGCGGATTGGTAAGAAAGGGCAGCCATGG

Helianthus_exilis_CHES4473
ATGGCGAGAACCAAACACCCTGCTAAACGCAGTTCAGGCATACCAGTAGAC------GGT
AGATCATCCACT------TCAACAAACACACCAAGAAAGAGTCCGAGGAAGAATCGA---
GGT------------GGAGAAAACAGG------AAGCCGCATAGGTTTAAGCCTGGGACA
CAGGCGCTACGTGAGATTAGGCGTTTGCAGAAGACGGTTGAACTGATCATTCCGGCTGCT
CCGTTTATTCGAACTGTAAAGGAGATAAGCAACTACATGGCCCCTGAAATCACTCGCTGG
CAAGCCGAAGCTCTACAAGCCCTTCAAGAGGCAGCAGAAGATTACCTAATTCAGTTGTTT
GAAGACTCAATGCTATGTGCGATTCATGCAAAGCGCGTTACCCTCATGAAAAAGGATTGG
GAGTTGGCACGGCGGATTGGGAAGAAAGGGCAGCCATGG

Lactuca_sativa
ATGGCGAGAACCAAACAACCTGCTAAACGAAGTTGGGGCAAGCGACAATCT------GCT
GGAGCATCA???TCAACC??????AGTACGCCACGGAAATCTCCGAGAAAGGATCCA---
GGGAGTTCAGGAACTGGACAGAGG???AAACAG???CCCCATCGTTTTAAGCCTGGAACT
CAAGCACTTCGTGAGATTCGTCGTCTTCAGAAGACTGTTAACCTTCTCATTCCAGCTGCT
CCTTTCATTCGAACTGTAAAAGAGATAAGCAACTACATTGCCCCTGAAGTTACACGTTGG
CAAGCTGAAGCTCTACAAGCCCTTCAAGAGGCTGCAGAAGATTACATAGTTCAGTTGTTT
GAAGATTCAATGTTGTGCTCGATTCATGCAAAGCGTGTTACCCTGATGAAAAAGGACATG
GAATTGGCTAGAAGGCTTACGAAGAAAGGGCAACCATGG

Taraxacum_koksaghyz
ATGGCGAGAACCAAACAACCTGCTAAACGGAGTGCGGGCAATCGAACACCT------GCT
CAAGCATCA???TCAACT??????AGTACGCCGCGAAGAAGTCCGAGAAAGGTTACA---
GGGAGTTCGGGAACGGGAGAAAGG???AAACAG???CCCCATCGTTTTAAGCCTGGTACT
CAGGCACTTCGTGAGATTCGTCGTCTCCAGAAGACCGTTAATCTACTCATTCCAGTCGCT
CCATTCGTACGAACTGTAAAGGAAATAAGCAACTACATTGCCCCTGAAGTCACACGCTGG
CAAGCTGAAGCTCTACAATGCCTTCAAGAGGCAGCAGAAGATTACTTAATCCGATTGTTT
GAAGATTCAATGCTATGTGCGATTCATGCAAAACGTGTTACCCTCATGCAAAAGGATTTG
GCATTGGCACGAAGGCTTACAAAGAAAGGGCAGCCATGG

;
end;

BEGIN TREES;
                    TREE tree = (Carthamus_tinctorius,(Taraxacum_koksaghyz,(Cichorium_intybus,Lactuca_sativa)),(Flaveria_vaginata,(Helianthus_annuus,Helianthus_exilis_CHES4473)));
END
```

Bony Fish

```
#NEXUS
[ Title ]
begin taxa;
    dimensions ntax=11;
    taxlabels
        Esox_lucius
        Haplochromis_burtoni
        Lates_calcarifer
        Micropterus_floridanus
        Misgurnus_anguillicaudatus
        Neolamprologus_brichardi
        Nothobranchius_furzeri
        Oncorhynchus_mykiss
        Ophthalmotilapia_ventralis
        Oreochromis_niloticus
        Salmo_salar
;
end;
begin characters;
    dimensions nchar=471;
    format missing=? gap=- matchchar=. datatype=nucleotide;
    matrix

Esox_lucius
ATG???CGCCGCGATCTT???ACTTCCACTGCAAGTCGACGCAAGGGCAAGGCACCAAAG
CGTCGGCCTCCAGTCCCAGCCCTGCCCGCTTCA------------------CCACGGCGG
AGTGGACCTTTAGCCTCTGCAGGTCCT????????????------???AAGAAGAAAAGG
TTCCGCCCCGGCACCCGAGCTCTGATGGAGATCCGGAAGTACCAGAAGAGTACCGACTTG
CTTCTGCGGAAGGGACCGTTTGCACGCCTGGTTCGGGAGGTGTGCCAGACGTTTAGCAGG
GACTACATGAGATGGCAGGTGTATGCCCTACTGGCCTTGCAGGAAGCTGCGGAGGCTTTC
CTTGTGTTATTGTTTTCTGATGCTTACCTCTGTACCATCCACGCCAAGCGTGTAACGCTG
TTCCCACGAGACATTCAACTTGCGCGACGAATTCGAGGAGCAGATCATCTC

Haplochromis_burtoni
ATGCGT------------CATAATTCATCTGCCAGCCGTCGGAAGGGTAAAACCCCTCAA
CGGCGCCCCCAAGTGCCAGCCCAAAGGACCCCCAGTGTAACATCC------CCAAGACGC
AGTGGAGTTCCA---------------GGACAGCCTCCTGTTTCCCCCAAGAAGAGGAAA
TTTCGACCAGGAACCAGAGCTTTGATGGAGATCCGCAAGTACCAGAAAAGCACAGATCTC
CTCCTTAGAAAGGCGCCCTTTGCCCGTTTGGTGCATGAGGTGTGCCAAAGCTTTTCTAGA
GAAAATCTTCGATGGCAGGTTTATGCTCTTCTGGCCCTCCAAGAGGCTGCAGAGGCTTTT
CTCGTCTTGATATTTTCAGATGCCAACCTGTGTGCCATCCACGCCAAGAGAGTCACTGTA
TTCCCTCGTGACATTCAGCTAGCCCGGAGGATCCGTGGGGTGGATAATCTG

Lates_calcarifer
ATGCGT------------CATGATTCATCTGCAAGTCGACGGAAGGGCAAAACCCCTCAA
CGTCGCCCCCCGGTACCCGACCCAGGGCCCTCTGGTTCCACA???AGGACCCCAAGACGA
AGCAGAGTTTCA---------------GGCGTACCTCCTGTGTCTCCCAAGAAAAGAAGG
TTTCGACCAGGAACCAAGGCCTTAATGGAAATCCGCAAGTACCAGAAGAGCACCGATCTT
CTGTTAAGGAAAGGACCCTTCTCTCGTCTGGTTCGTGAGGTGTGCCAAGGTTTTTCCAGA
GAGGCTCTCAGGTGGCAGGTCTATGCTCTTCTTGCCCTGCAGGAGGCTGCTGAGGCCTTT
CTTGTCATGTTATTCTCAGACGCCAACCTGTGTGCCATCCATGCCAAGCGGGTAACCGTG
TTCCCCCGCGACATTCAATTGGCAAGGAGGATCCGTGGGGTGGATAACATG

Micropterus_floridanus
ATGCGT------------CATGATTCATCAGCCAGCCGCCGGAAGGGGAAAACCCCTCAA
CGTCGCCCCCCGCAGCCGGCCCCAGAGACC---------------CGGTCCCCAAGACGG
AGCAGAGTTTCA---------------AGGCAGCTTCCTGCGTCTCCCAAGAAGAGGAGG
TTTCGACCAGGAACCAAGGCCTTAATGGAGATCCGCAAGTTCCAGAAGAGCACTGATCTT
TTGCTCAGGAAAGGACCCTTCTCTCGCCTGGTTCGTGAGGTATGCCAGAGTTTTTCCAGA
GAGGCTCTCCGGTGGCAGGTCTACGCACTTCTGGCCCTGCAGGAGGCTGCAGAGGCGTTT
CTCGTCATGCTATTCTCAGACGCAAATCTGTGTGCCATCCACGCCAAGCGGGTCACCGTG
TTCCCCCGTGATATTCAGCTGGCCAGGAGGATCCGCGGCGTTGATAACCTG

Misgurnus_anguillicaudatus
ATGCGG------------CACAATAAATCAGCA?????????????????ACCCCCAGG
CGCAGATCCCCT---CCTGCTACACCAGCAGCACCCTCCACA???AGAACCCGACGCACC
AGTGGACCATCC---------------GAGAAA??????------???AAGAAGCACAAG
TTTCGACCTGGGACGCGAGCTCTCATGGAGATACGCAAATATCAGAAATCCACAGATCTG
TTGTTGCGCAAGGGCCCGTTTTCACGACTGGTACGTGAGGTGTGTCAGACATTTAGCCGG
GAGAACTTAATGTGGCAGGGCTATGCACTAATGGCTTTACAAGAGGCTGCAGAGGCGTTC
ATGGTCCGTTTGTTCTCTGATGCCAATCTCTGTGCTATTCATGCCAAGAGGGTGACATTG
TTTCCACGTGATATACAGCTTGCCCGTCGAATCAGAGGTGTTGAAAACATG

Neolamprologus_brichardi
ATGCGT------------CATAATATATCTGCCGGCCGTCGGAAGGGTAAAACCCCTCAA
CGGCGTCCCCAAGTGCCAGCCCAAAGCACCTCCAGTGTAACATCC------CCAAGACGC
AGTGGAGTTCCA---------------GGACTGCCTCCTGTTTCCCCCAAGAAGAGGAAA
TTTCGACCAGGAACCAGAGCTTTGATGGAGATCCGCAAGTACCAGAAAAGCACAGATCTC
CTCCTTAGAAAGGCGCCCTTTGCCCGTTTGGTTCATGAGGTGTGCGAAAGCTTTTCTAGA
GAAAGGCTCCGATGGCAGGTTTATGCTCTTCTGGCCCTCCAAGAGGCTGCAGAGGCTTTT
CTCGTCTTGATATTTTCAGATGCCAACCTGTGTGCCATACACGCCAAGAGAGTCACTGTA
TTCCCTCGTGACATTCAGCTAGCCCGGAGGATCCGTGGGGTGGATAATATG

Nothobranchius_furzeri
ATGCGT------------CACAGTTCACCGCGCAGCCGGCGGAAGGCCAAAACTCCCAGT
CGTCGTGACCCACCTCCATCTCCCACAGCATCA?????????------???CCAAGAAAA
AGTGGTTTTTCT---------------GGGATTCCTCCTGTGTCTCCCAAGAAAAGGAGG
TACAGACCTGGAACCAGAGCCTTGATGGAGATTCGTAAGTACCAGAAGAGTACCGATCTT
CTGCTCAGGAAAGCACCATTCGCTCGCTTGGTTCACGAGGTGTGCCAGAGTTATACCAGA
GGCAATTTGCGGTGGCAGGTCTTCGCTCTTCTTGCCCTGCAGGAGGCAGCAGAGGCCTTC
CTAGTCCTGCTGTTTTCAGATGCCAACCTGTGTGCCATTCATGCTAAGAGGGTGACTGTG
TTTCCTCGGGACATTCAGCTGGCCAGGAGGATCCGTGGAGTGGATCGTCTG

Oncorhynchus_mykiss
ATG???CGTCACGATCCT???AGTTCTTCTGCAAGCCGGCGCAAGGGTGCGGTACCCAAG
CGTCGGCCTCCAGCCTCCACCGCGACTGCTTCAACATCAAAG???AAGGCACCACGGCTG
AGTGGACCATCGGCCTCTGCTGTTCCA????????????------???AAAAATAGAAGG
TTTCGCCCTGGCACCCGAGCTCTGATGGAAATTCGCAAGTACCAGAAGAGCACTGACTTG
CTTTTGCGCAAGGCACCGTTTGCACGTCTGGTTCGGGAGGTGTGCCATACTTATACCAGG
GACTTCATGAGATGGCAGGTGAACGCTCTTCTGGCCTTGCAGGAGGCTGCAGAGGCTTTC
CTTGTTTTACTGTTTTCCGACGCCTACCTGTGTACAATCCACGCCAAGCGTGTAACGCTG
TTCCCCCGTGACATTCAGCTTGCCCGGCGAATTCGAGGAGTGGATGACCTC

Ophthalmotilapia_ventralis
ATGCGT------------CATAATCCATCTGCCAGCCGTCGGAAGGGTAAAACCCCTCAA
CGGCGCCCCCAAGTGCCAGCCCAAAGGACCTCCAGTGTAACGTCC------CCAAGACGC
AGTGGAGTTTCA---------------GGACAGCCTCCTGTTTCCCCCAAGAAGAGGAAA
TTTCGACCGGGAACCAGAGCTTTGATGGAGATCCGCAAGTACCAGAAAAGCACAGATCTC
CTCCTTAGAAAGGCGCCCTTTGCCCGTTTGGTTCATGAGGTGTGCCAAAGCTTTTCTAGA
GAAAGGCTCCGATGGCAGGTTTATGCTCTTCTGGCCCTCCAAGAGGCTGCAGAGGCTTTT
CTCGTCTTGATGTTTTCAGATGCCAACCTGTGTGCCATCCACGCCAAGAGAGTCACTCTA
TTCCCTCGTGACATTCAGCTAGCCCGGAGGATCCGTGGGGTGGATAATCTG

Oreochromis_niloticus
ATGCGT------------CATAATTCATCTGCCAGCCGTAGGAAGGGTAAAACCCCTCAA
CGGCGCCCCCAAGTGCCAGCCCAAAGGACCTCCAGTGTAACATCC------CCAAGACGC
AGTGGAGTTCCA---------------GGACAGCCTCCTGTTTCCCCCAAGAAGAGGAAA
TTTCGACCAGGAACCAGAGCTTTGATGGAGATCCGCAAGTACCAGAAAAGCACAGATCTC
CTCCTTAGAAAGGCGCCCTTTGCCCGTTTGGTTCATGAGGTGTGCCAAAGCTTTTCTAAA
GAAAGGCTCCGATGGCAGGTTTATGCTCTTCTGGCCCTCCAAGAGGCTGCAGAGGCTTTT
CTCGTCTTGATATTTTCAGATGCCAACCTGTGTGCCATCCACGCCAAGAGAGTCACTGTA
TTCCCTCGTGACATTCAGCTAGCCCGGAGGATCCGTGGGGTGGATAATCTG

Salmo_salar
ATG???CGTCGCGATCCT???GTTTCTTCTGCAAGCCGGCGCAAGGGTGCAGTACCCAAG
CGTCGGCCTCCAGCCTCCACCGCGACTGCTTCAACATCAAAG???AAGGCACCACGGCTG
AGCGGACCATCGGCCCCTGCAGGTCCA????????????------???AAAAAGAGAAGG
TTTCGCCCTGGCAACCGAGCTCTGCTGGAAATTCGCAAGTACCAGAAAAGCACTGACTTG
CTTTTGCGCAAGGGACCGTTTGCACGCCTGGTTCGGGAGGTGTGCCAGACTTATAGCAGG
GACTTCATGAGATGGCAGGTGAACGCTCTTCTGGCCTTGCAGGAGGCTGCAGAGGCTTTC
CTCGTTTTACTGTTTTCCGACGCCTACCTGTGTACCATCCACGCCAAGCGTGTAACGCTG
TTCCCCCGTGACATTCAGCTTGCCCGGCGAATTCGAGGAGTGGATGATCTC

;
end;

BEGIN TREES;
        TREE tree = (Esox_lucius,(Oncorhynchus_mykiss,Salmo_salar),(Misgurnus_anguillicaudatus,(Nothobranchius_furzeri,((Haplochromis_burtoni,(Oreochromis_niloticus,(Neolamprologus_brichardi,Ophthalmotilapia_ventralis))),(Lates_calcarifer,Micropterus_floridanus)))));
END
```

Brassicaceae

```
#NEXUS
[ Title ]
begin taxa;
    dimensions ntax=20;
    taxlabels
        Arabidopsis_arenosa_HTR12
        Arabidopsis_halleri_subsp._halleri_HTR12B1
        Arabidopsis_suecica_cenpA
        Arabidopsis_thaliana_HTR12
        Arabis_hirsuta_cenpA1
        Brassica_carinata_BrCENH36
        Brassica_juncea_BrCENH35
        Brassica_napus_BrCENH38
        Brassica_nigra_BrCENH32
        Brassica_oleracea_BrCENH33
        Capsella_bursapastoris_cenpA1
        Cardamine_flexuosa_cenpA1
        Crucihimalaya_himalaica_HTR12
        Crucihimalaya_wallichii_cenpA
        Eruca_sativa_cenpA
        gArabidopsis_lyrata_HTR12
        Lepidium_virginicum_cenpA1
        Olimarabidopsis_pumila_cenpA1
        Raphanus_sativus_cenpA1
        Turritis_glabra
;
end;
begin characters;
    dimensions nchar=654;
    format missing=? gap=- matchchar=. datatype=nucleotide;
    matrix

Arabidopsis_arenosa_HTR12
ATGGCGAGAACCAAGCATTTCGCTACCAAGTCACGAACTGGGAATCGAACT------GAT
???---???GCT---TCATCTTCTCAG---------GCGACAGGTCCGACTACGACCCCG
ACAACAAGA---GGC---------ACTGAA------------GGTGGA------------
---------GATAAT---ACTCAACAAACAAATCCT---ACAACT------------TCA
CCAGCTACT---------GGTGGAAGG???---------------CCTAGGAGAGCCAGA
CAGGCTATGCCGCGAGGTTCA---------CAG---AAGAAACCTTATCGATTCAAGCCA
GGAACCGTTGCTCTGAGAGAGATTCGACATTTCCAGAAGCAGACAAACCTTCTTATTCCA
GCTGCTAGCTTCATAAGACAAGTGAGAAGTATAACTCAT------GCGTTGGCCCCTCCC
CAAATCAATCGTTGGACAGCTGAAGCTCTTGTGGCTCTTCAAGAGGCGGCAGAAGATTAC
TTGGTTGGTTTGTTCTCAGATTCAATGCTCTGTGCTATCCATGCGAGACGTGTTACTCTA
ATGAGAAAAGACTTTGAGCTTGCACGCCGGCTTGGAGGAAAAGGCAGACCATGG

Arabidopsis_halleri_subsp._halleri_HTR12B1
ATGGCGAGAACCAAGCATTTCGCTACCAGGACACGATCTGGGAATCGAACT------GAT
GCC---AATGCT---TCATCTTCTCAG---------GCGGCAGGTCCGACTACGACCCCG
ACAACAAGA---GGC---------ACTGAA------------GGTGGA------------
---------GATAAT---ACTCAACAAACAAATCCT---ACAACT------------TCA
CCAGCTACT---------GGTGGAAGG???---------------CCTAGGAGAGCCAGA
CAGACTATGCCACGAGGTTCA---------CAG---AAAAAGCCTTATCGATACAAGCTA
GGAACCGTTGCTCTGAGAGAGATTCGACATTTCCAGAAGCAGACAAACCTTCTTATTCCA
GCTGCTAGCTTCATAAGACAAGTGAGAAGTATAACTCAT------GCGTTGGCCCCTCCC
CAAATCAATCGTTGGACAGCTGAAGCTCTTGTGGCTCTTCAAGAGGCGGCAGAAGATTAC
TTGGTTGGTTTGTTCTCAGATTCAATGCTCTGTGCTATCCATGCAAGACGTGTTACTCTA
ATGAGAAAAGACTTTGAGCTTGCACGCCGGCTTGGAGGAAAAGGCAGACCATGG

Arabidopsis_suecica_cenpA
ATGGCGAGAACCAAGCATTTCGTTACCAAGTCACGAACTGGGAATCGAACT------GAT
GCC---AATGCT---TCATCTTCTCAG---------GCGTCAGATCCGACGACGACCCCG
ACAACAAGA---GGC---------ACTGAA------------GGTGGA------------
---------GATAAT---ACTCAACAAACAAATCCT---ACAACT------------TCA
CCAGCTACT---------GGTGGAAGG???---------------CCTAGGAGAGCCAGA
CAGGCTATGCCGCGAGGTTCA---------CAG---AAGAAGCCTTATCGATACAGGCCA
GGAACCGTTGCTCTGAGAGAGATTCGACATTTCCAGAAGCAGACAAACCTTCTTATTCCA
GCTGCTAGCTTCATAAGACAAGTGAGAAGTATAACCCAT------GCGCTGGCCCCTCCC
CAAATCAATCGTTGGACAGCTGAAGCTCTTGTGGCTCTTCAAGAGGCGGCAGAAGATTAC
TTGGTTGGTTTGTTCTCAGATTCAATGCTCTGTGCTATCCATGCAAGACGTGTTACTCTA
ATGAGAAAAGACTTTGAGCTTGCACGCCGGCTTGGAGGAAAAGGCAGACCATGG

Arabidopsis_thaliana_HTR12
ATGGCGAGAACCAAGCATCGCGTTACCAGGTCACAACCTCGGAATCAAACT------GAT
???GCC???GCT---TCATCTTCTCAG---------GCGGCAGGTCCAACTACGACCCCG
ACAAGGAGA---GGC---------GGTGAA------------GGTGGA------------
---------GATAAT---ACTCAACAAACAAATCCT---ACAACT------------TCA
CCAGCTACT---------GGTACAAGGAGA------------GGGGCTAAGAGATCCAGA
CAGGCTATGCCACGAGGCTCA---------CAG---AAGAAGTCTTATCGATACAGGCCA
GGAACCGTTGCTCTAAAAGAGATTCGCCATTTCCAGAAGCAGACAAACCTTCTTATTCCG
GCTGCCAGTTTCATAAGAGAAGTGAGAAGTATAACCCAT------ATGTTGGCCCCTCCC
CAAATCAATCGTTGGACAGCTGAAGCTCTTGTTGCTCTTCAAGAGGCGGCAGAAGATTAC
TTGGTTGGTTTGTTCTCAGATTCAATGCTCTGTGCTATCCATGCAAGACGTGTTACTCTA
ATGAGAAAAGACTTTGAACTTGCACGCCGGCTTGGAGGAAAAGGCAGACCATGG

Arabis_hirsuta_cenpA1
ATGGCGAGAACGAAGCATTTCGCA------AGACGAGGTCAGAATCGAACA------GAT
GCT---AACGTT---TCCTCTTCGCCG---------GCGGCAGGTCCGAGTACGACTCCG
GCGAGAAGG---GCT---------AGTGAA------------GATGGA------------
---------GATGAT---GCTCAACAAACAACTCCT---GCAACT------------TCA
CCAGCTACT---------GGTAGTAAAAAT---AGTAATGGAGGAGCTAAGAGAACT---
------ATGCCACAAAGTTCA---------CAG---AAGAAGCCTCATCGATACAGGCCA
GGAACCGCTGCTCTGAGAGAGATTCGCAAATTCCAGAAGTCCACAAAACTTCTAATTCCT
GCTGCTAGTTTTATCAGAGAAGTGAAAGATATAACCTATACTTAT------GCTCCTCCG
CAAATCACTCGTTGGACAGCTGAAGCTCTTGTAGCTATCCAAGAGGCGGCAGAAGATTAC
TTAGTTGGTTTGTTCTCAGATTCAATGCTTTGTGCTGTTCATGCAAGACGTGTTACTCTA
ATGAGAAAAGACTTTGAGCTTGCACGCCGGCTTGGAGGAAAAGGCAGACCATGG

Brassica_carinata_BrCENH36
ATGGCGAGAACCAAACATTTCGCTTCCAGGGCACGAGATCGCAATCGAACT------AAT
GCG---ACTGCT---TCATCTTCG???GCGGCG---???GAAGGTCCGAGTGCGACCCCG
ACGAGAAGA---GAAGGC------???CAAGATGAA------??????------------
------------???---GCTCAACAGAGT---------GCA???CCTACTACAACTCCA
TCA------------GCCGGTAGAAAAAAAGGA---------GGGACTAAGCGAACTAAA
CAAGCTATGCCTAAAAGTTCCAAC------AAG---AAGAAGACATTCCGTTACAAGCCT
GGAACCGTTGCCCTCAGAGAGATTCGCCATTTCCAGAAGACCACCAAACTCCTTATCCCT
GCCGCTAGTTTCATCCGACAAGTGAGAAGTGTCACCCAGATCTTT------GCCCCTCCC
GATGTTACCCGTTGGACTGCTGAAGCTCTTATGGCTATTCAAGAGGCGGCTGAAGATTTT
TTAATTGGCTTGTTCTCTGATGCTATGCTTTGCGCTATCCACGCAAGACGTGTTACTCTA
ATGAGAAAAGATTTTGAGCTTGCACGCCGTCTTGGAGGAAAAGGCAGACCATTG

Brassica_juncea_BrCENH35
ATGGCGAGAACGAAGCATTACGCATCCAGGGCACGAGATCGCAATCGAAAT------AAT
GCG---ACTGCT---TCATCTACC???GCGGCG---???GCAAGTCCGAGTGCGACGCCG
ACGAGAAGA---GGC???AGCAGC???CAA------------GGT???CGT---------
------------???---GCTCAACAG--------------------------------
-----------------------------------------------------------
---------------AGTTCCAACCAGAAAAAG---AAGAAGTCTTTCCGTTACAAGCCT
GGAACCGTTGCTCTCAGAGAGATTCGCCAGTTCCAGAAGACCACCAAACTTCTCATCCCT
GCTGCTAGTTTCATCAGAGAAGTGAGAAGTATTACCCATATCCTT------GCTCCTCCC
GAAATTACGCGTTGGACTGCTGAAGCTCTTGTTGCTCTTCAAGAGGCGGCAGAAGATTAC
TTAGTTGGCTTGTTCTCTGATGCAATGCTCTGCGCTATCCACGCAAGACGTGTTACTCTA
ATGAGAAAAGATTTTGAGCTTGCACGCCGTCTTGGAGGAAAAGGCAGACCATTG

Brassica_napus_BrCENH38
ATGGCGAGAACGAAACATTTCGCTTCCAGGGCACGAGATCGCAATCGAACT------AAT
GCG---ACTGCT---TCATCTTCG???GCGGCG---???GAAGGTCCGAGTGCGACCCCG
ACGAGAAGA---GAAGGC------???CAA---------------???------------
------------???---GCTCAACAGACA---------GCA???CCTACTACGACTCCA
CCA------------GCCGGTAGAAAAAAAGGA---------GGGACTAAGCGAACTAAA
CAAGCTATGCCTAAAAGTTCCAAC------AAG---AAGAAGACATTCCGTTACAAGCCT
GGAACCGTTGCCCTCAGAGAGATTCGCCATTTCCAGAAGACCACCAAACTTCTTATCCCT
GCCGCTAGTTTCATCCGAGAAGTGAGAAGTGTCACCCAGATCTTT------GCCCCTCCC
GATGTTACCCGTTGGACTGCTGAAGCTCTTATGGCTATTCAAGAGGCGGCTGAAGATTTT
TTAATTGGCTTGTTCTCTGATGCTATGCTTTGCGCTATCCACGCAAGACGTGTTACTCTA
ATGAGAAAAGATTTTGAGCTTGCACGCCGTCTTGGAGGAAAAGGCAGACCATTG

Brassica_nigra_BrCENH32
ATGGCGAGAACGAAGCATTACGCATCCAGGGCACGAGATCGCAATCGAAAT------AAT
GCG---ACTGCT---TCATCTACC???GCGGCG---???GCAAGTCCGAGTGCGACGCCG
ACGAGAAGAGGAGGC???AGCAGC???CAA------------GGT???CGT---------
------------???---GCTCAACAG--------------------------------
-----------------------------------------------------------
---------------AGTTCCAACCAGAAAAAG---AAGAAGTCTTTCCGTTACAAGCCT
GGAACCGTTGCCCTCAGAGAGATTCGCCAGTTCCAGAAGACCACCAAACTTCTCATCCCT
GCTGCTAGTTTCATCAGAGAAGTGAGAAGTATTACCCATATCCTT------GCCCCTCCC
GAAATTACGCGTTGGACTGCTGAAGCTCTTGTTGCTCTTCAAGAGGCGGCAGAAGATTAC
TTAGTTGGCTTGTTCTCTGATGCAATGCTCTGCGCTATCCACGCAAGACGTGTTACTCTA
ATGAGAAAAGATTTTGAGCTTGCACGCCGTCTTGGAGGAAAAGGCAGACCATTG

Brassica_oleracea_BrCENH33
ATGGCGAGAACCAAACATTTCGCTTCCAGGGCACGAGATCGCAATCGAACT------AAT
GCG---ACTGCT---TCATCTTCG???GCGGCG---???GAAGGTCCGAGTGCGACCCCG
ACGAGAAGA---GAAGGC------???CAAGATGAA------??????------------
------------???---GCTCAACAGAGT---------GCA???CCTACTACAACTCCA
TCA------------GCCGGTAGAAAAAAAGGA---------GGGACTAAGCGAACTAAA
CAAGCTATGCCTAAAAGTTCCAAC------AAG---AAGAAGACATTCCGTTACAAGCCT
GGAACCGTTGCCCTCAGAGAGATTCGCCATTTCCAGAAGACCACCAAACTCCTTATCCCT
GCCGCTAGTTTCATCCGACAAGTGAGAAGTGTCACCCAGATCTTT------GCCCCTCCC
GATGTTACCCGTTGGACTGCTGAAGCTCTTATGGCTATTCAAGAGGCGGCTGAAGATTTT
TTAATTGGCTTGTTCTCTGATGCTATGCTTTGCGCTATCCATGCAAGGCGTGTTACTCTA
ATGAGAAAAGATTTTGAGCTTGCACGCCGTCTTGGAGGAAAAGGCAGACCATTG

Capsella_bursapastoris_cenpA1
ATGGCGAGAACAAAGCATTTCGCTACGAGGTCCGGACCTCGGACTCCAGCT------GTT
---------GCT---TCGTCTTCTCAG---------GCGGCTGTTCCCAGTTCGAGCCCG
GCAACAAGA---GGC---------AGAGTA------------GGTGTA------------
---------GAC???GCT???CAACAACCAACTCCT---GCAACT------------TCA
CCTGCTACT---------GCTAAAAAAAAA------------GGGGCGAAGAGAGCTAGA
TTTGGTAGGCCACAAGGTTCA---------CAAAAA???AAGCCTTACCGTTACAGGCCA
GGAACCGTTGCTCTGAGAGAGATTCGCCATTATCAGAAGGGCACTAGCCTTCTTATTCCA
GCTGCTGCCTTTATCAGACAAGTGAGAAGTATAACCAAT------GCAGTGGCCCCTCGG
GAAGTAAATCGTTGGACAGCTGAAGCTCTTGTGGCTCTTCAAGAGGCGGCAGAAGATTTC
TTGGTTGGTTTGTTCTCTGATTCAATGCTCTGTGCTATCCATGCAAGACGTGTTACTCTA
ATGAGAAAAGACTTTGACCTTGCACGCCGGCTTGGAGGAAAAGGCAGACCCTGG

Cardamine_flexuosa_cenpA1
ATGGCGAGAACGAAGCATTTCCCTAACAGGACACGGCCTCGGACTCAAACT------GAT
GCC---ACTGCT---TCATCTACTCCG---------GCGGCTGGTCCGAGTACGAAGCGG
CCAACAAGG---GCC---------AATCAA------------GGT???------------
------------???---ACTCAACAAACAAATCCT---ACAACT------------TCA
CCAGCTACC---------AGTAAAAAGAAA------------GGGGCTAAGAGAACTAGA
CGGGCCATGCCACAAGGTTCA---------CAA---AAGAAGCCTAATCGTTACAAGCCA
GGAACCGTTGCTCTTAGAGAGATTCGCCATTTCCAGAAGAGCACAAATCTTCTGATTCCA
GCTGCTAGTTTTATCAGACAAGTGAGAAGTATAACCCAAATGTAT------GCCCCTCCG
CAAATTAATCGTTGGACTGCTGAAGCTCTTGTGGCTCTTCAAGAGGCGGCAGAAGATTAC
TTGATTGGTTTGTTCTCAGATTCAATGCTCTGTGCTATCCATGCAAGACGTGTTACTCTA
ATGAGGAAAGACTTTGAGCTTGCACGCCGGCTTGGAGGAAAAGGCAGACCATGG

Crucihimalaya_himalaica_HTR12
ATGGCGAGAACCAAGCATTTCGCTACTAGGTCACGACCTCGGAATCAAACT------GAT
GCC---ACTGCT---TCAGCTTCTCAG---------GCGACAGGTCCGAGTACGAACCCG
ACAACAAGA---GGT---------AGTGAA------------GGTGAA------------
---------GATGCT---GCTCGAGGAACAAATCCT---ACAACT------------TCA
CCAGCTACT---------GGTAGAAAAAAA------------GGGGTTAAGAGGGCCAGA
CATGCTATGCCACAAGGTTCA---------CAG---AAGAAGCCTTATCGTTACAAGGCA
GGAACAGTCGCTCTGAGAGAGATTCGCCATTTTCAGAAGAACACAAACCTTCTTATTCCA
GCTGCTAGTTTTATAAGACAAGTGAAAAGTATAACCTAT------GCGGTGGCCCCTCCG
CAAATCACTCGTTGGACAGCTGAAGCTCTTGTGGCTCTTCAAGAGGCGGCAGAAGATTAC
TTGGTTGGTTTGTTCTCAGATTCAATGCTCTGTGCTATCCATGCAAGACGAGTTACTCTA
ATGAGAAAAGACTTTGAGCTTGCACGCCGGCTTGGAGGAAAAGGTAGACCCTGG

Crucihimalaya_wallichii_cenpA
ATGGCGAGAACCAAGCATTTCGCTACTAGGTCACGACCTCGGAATCAAACT------GAT
GCC---ACTGCT---TCAGCTTCTCAG---------GCGACAGGTCCGAGTACGAACCCG
ACAACAAGA---GGT---------AGTGAA------------GGTGAA------------
---------GATGCT---GCTCGAGAAACAAATCCT---ACAACT------------TCA
CCAGCTACT---------GGTAGAAAAAAA------------GGGGTTAAGAGGGCCAGA
CATGCTATGCCACAAGGTTCA---------CAG---AAGAAGCCTTATCGTTACAAGGCA
GGAACAGTCGCTCTGAGAGAGATTCGCCATTTTCAGAAGAACACAAACCTTCTTATTCCA
GCTGCTAGTTTTATAAGACAAGTGAAAAGTATAACCTAT------GCGGTGGCCCCTCCG
CAAATCACTCGTTGGACAGCTGAAGCTCTTGTGGCTCTTCAAGAGGCGGCAGAAGATTAC
TTGGTTGGTTTGTTCTCAGATTCAATGCTCTGTGCTATCCATGCCAGACGAGTTACTCTA
ATGAGAAAAGACTTTGAGCTTGCACGCCGGCTTGGAGGAAAAGGTAGACCCTGG

Eruca_sativa_cenpA
ATGGCGAGAACGAAGCATTTTGCATCCAGGGCACGAGATCGCAATCGAAAT------AAT
GCG---ACAGCT---TCATCTTCC?????????GCG??????GGTCCGAGTGCGACACCG
ACGAGAAGA---GGCAGC------???CAA------------GGT???---GGTGGTGGT
GGTGGTGTT---???---GCTCAACAG--------------------------------
-----------------------------------------------------------
---------------GGTTCCAAC---??????---???AAGTCTTTCCGTTACAAGCCT
GGAACCGTTGCTCTCAGAGAGATTCGCCATTTCCAAAAGACCACCAAACTCCTTATCCCT
GCTGCTACTTTCATCCGACTAGTCAGAAGCATCACC?????????------??????CCC
CAAGTTACGCGTTGGACTGCTGAAGCTCTTGTTGCTCTTCAAGAGGCGGCAGAAGATTAC
TTAGTTGGCTTGTTCTCTGATTCAATGCTCTGCGCTATCCATGCTAAACGTGTTACTCTT
ATGAGGAAAGATTTTGAGCTTGCACGCCGCCTTGGAGGTAAAGGCAGACCATGG

gArabidopsis_lyrata_HTR12
ATGGCGAGAACCAAGCATTTCGCTACCAAGTCACGATCTGGGAATCGAACT------GAT
GCC---AATGCT---TCATCTTCTCAG---------GCGGCAGGTCCGACGACGACCCCG
ACAACAAGA---GGC---------ACTGAA------------GGTGGA------------
---------GATAAT---ACTCAACAAACAAATCCT---ACAACT------------TCA
CCAGCTACT---------GGTGGAAGG???---------------CCTAGGAGAGCCAGA
CAGGCTATGCCGCGAGTTTCA---------CAG---AATAAGCCTTATCGATACAAGCCA
GGAACCGTTGCTCTGAGAGAGATTCGACATTTCCAGAAGCAGACAAACCTTCTTATTCCA
GCTGCTAGCTTCATAAGACAAGTGAGAAGTATAACTCAT------GCGTTGGCCCCTCCC
CAAATCAATCGTTGGACAGCTGAAGCTCTTGTGGCTCTTCAAGAGGCTGCAGAAGATTAC
TTGGTTGGATTGTTCTCAGATTCAATGCTCTGTGCTATCCATGCAAGACGTGTTACTCTA
ATGAGAAAAGACTTTGAGCTTGCACGCCGGCTTGGAGGAAAAGGCAGACCATGG

Lepidium_virginicum_cenpA1
ATGACGAGAACCAAGCATTACGTTTCGAGGCCACAACCT??????------CGCAAGGAT
GCC---ACTGTTTCTTCATCTTCTCCG---------GCGGCTGGTACAAGTACGAATCGA
ACAACAAGG---AGT---------TCT???------GGTGAA???GGA------------
---------GATAAT---GCTCAACAAACAATTCCT---ACAACT------------TCA
CCGGCTACT---------AATAGCAAAAAA------------GGGGCAAAAAGAACCAAA
GAAACTATGCCACAAAGTTCA---------AAT---AAGAAAACATATCGATACAAGCCA
GGCACCGTTGCTCTGAGAGAGATTCGCCATTTTCAGAAGAGCACAAATCTTCTAATTCCA
GCTGCTAGTTTCATAAGAGAAGTGAGATGTATTACTCAG------GCTGTGGCCCCTCCG
CAAATCAATCGTTGGACAGCTGAAGCTCTTGTGGCTCTTCAAGAGGCGGCAGAAGACCAC
GTGGTTGGCTTGTTCTCTGATTCGATGCTTTGTGCTATCCATGCAAGACGAGTTACACTT
ATGAAAAAAGATTTTGAGCTTGCACGCCGGCTTGGAGGAAAAGGCAGACCATGG

Olimarabidopsis_pumila_cenpA1
ATGGCGAGAACCAAGCATAACGCTATCAGGTCACGA------GATCGAACT------GGT
GCA---ACTGCT---TCATCATCTCAG---------GCAGCAGGTCCGAGTACGAACCCC
ACAGCAGGA---GGT---------AGT???-----------------------------
---------GATGCT---GCTCAACAAACAACTCCT---ACGACT------------TCA
CCAGCTACT---------GGTAGTAAAAAA------------CGGGCTAAGAGAGCCAGA
CAGGCTATGCCACGAGGTTCA---------CAG---AAGAAGCCTTATCGTTACAAGCCA
GGAACTGTTGCTCTGAGAGAGATTCGCCATTTTCAGAAGACCACTAGCCTTCTTCTTCCA
GCTGCTCCTTTTATAAGACAAGTGAGAAGTATAAGCAGT------GCTTTGGCACCTAGG
GAAATCACTCGTTGGACAGCTGAAGCTCTTGTGGCTCTCCAAGAGGCGGCAGAAGATTAC
TTGGTTGGTTTGTTCTCAGATTCCATGCTCTGTGCTATCCATGCAAAACGTGTTACTCTG
ATGAGAAAAGACTTTGAGCTTGCACGCCGGCTCGGAGGAAAAGGCAGACCATGG

Raphanus_sativus_cenpA1
ATGGCGAGAACGAAGCATTTCGCTTCCAGAGCACGAGATCGCAATCAACCT------AAT
GCG---------------------???GCGGCG---???GCAGGTCCGAGTGCGACACCG
ACGAGAAGA---GGCAGC------???CAA------------GGT???------------
------------???---GCTCAACAGACAACGCCTACCACAACT------------TCA
CCAGCAACAACCGCCTCCGGTAGAAAAAAA------------GGGACTAAGCGAACTACA
CAAGCTATGCCTAAAAGTTCC---------AAG---AAGAAGACTTTCCGTTACAAGCCT
GGAACTGTTGCCCTCAGAGAGATTCGCCATTTCCAGAAGTCCACCAAACTTCTCATCCCT
TCTGCTCCTTTCATCCGAGAAGTCAGAAGTATCACCCACAACCTT------GCCGCTGCC
TATGTTACGCGTTGGACTGCTGAAGCTCTTATTGCTCTTCAAGAGGCGGCAGAAGATTTC
TTAGTTGGCTTGTTCTCTGATGCAATGCTTTGCGCTATCCATGCAAAACGTGTTACTCTA
ATGAGAAAAGATTTTGAGCTTGCACGCCGGCTTGGAGGAAAAGGCAGACCCTTT

Turritis_glabra
ATGGCGAGAACCAAGCATTTCGCTACCAGGTCACGACCTCGGAATCAAACT------GAT
---------------TCATCTTCTCAG---------GCGGCAGGTCCGAGTACGAACCCA
ACAACAGGA---GGT---------AGTGAA------------GGTGGA------------
---------GATGCT---GCTCAACAAACAACTCCT---ACGACT------------TCA
CCAGCTACT---------GGTAGAAAAAAA------------CGGGCTAAGAGAGCCAAA
CAGGCTATGCCACAAGGTTCA---------CAG---AAGAAGCCTTATCGTTACAAGCCA
GGAACCATTGCTCTGAGAGAGATTCGCTATTTTCAGAAGAACACAAACCTTCTTATTCCA
GCTGCTAGTTTTATAAGAGAAGTGAGAAGTATAACCCAT------GCGCTGGCCCCTCCG
CAAATCTCTCGTTGGACAGCTGAAGCTCTTGTTGCTCTCCAAGAGGCGGCAGAAGATTAC
TTGGTTGGTTTGTTCTCAGATTCCATGCTCTGTGCAATCCATGCAAGACGTGTTACTCTG
ATGAGAAAAGACTTTGAGCTTGCACGCCGGATTGGAGGAAAAGGCAGACCATGG

;
end;

BEGIN TREES;
        TREE tree =
(((((Arabidopsis_arenosa_HTR12,(Arabidopsis_suecica_cenpA,gArabidopsis_lyrata_HTR12)),Arabidopsis_halleri_subsp._halleri_HTR12B1),Arabidopsis_thaliana_HTR12),((((Raphanus_sativus_cenpA1,((Eruca_sativa_cenpA,(Brassica_juncea_BrCENH35,Brassica_nigra_BrCENH32)),(Brassica_napus_BrCENH38,(Brassica_carinata_BrCENH36,Brassica_oleracea_BrCENH33)))),Arabis_hirsuta_cenpA1),(Cardamine_flexuosa_cenpA1)),Lepidium_virginicum_cenpA1)),(Turritis_glabra,Olimarabidopsis_pumila_cenpA1),((Crucihimalaya_himalaica_HTR12,Crucihimalaya_wall
ichii_cenpA),Capsella_bursapastoris_cenpA1));
END;
```

Bryophyta

```
#NEXUS
[ Title ]
begin taxa;
     dimensions ntax=10;
     taxlabels
          Anomodon_attenuatus
          Bryum_argenteum
          Ceratodon_purpureus
          Hedwigia_ciliata
          Leucodon_brachypus
          Physcomitrella_patens
          Pohlia_nutans
          Rhynchostegium_serrulatum
          Rosulabryum_capillare
          Thuidium_delicatulum
;
end;
begin characters;
     dimensions nchar=444;
     format missing=? gap=- matchchar=. datatype=nucleotide;
     matrix

Anomodon_attenuatus
ATGGCGAGGCGGAAGTCGACCCCTCTGCACGGCAACAAACGCGCACCCACCCGC---CCG
---------???GCT------GCTGCTGCTGCTGCCTCGACTTCG---------------
ACGAGGAGGCCGAGGAAAGCGCACCGATGGAGACCGGGCACCAAGGCGTTGCAGGAGATT
CGGCACTACCAGAAGACGTGCGACTTGCTCATCCCCCGACTGCCGTTCGCTCGCTATGTG
AAGGAAATCACGTCTATGTATGCGAGTGACGTTTCTCGATGGACGGCGGAGGCTTTGACA
GCCCTTCAAGAGGCAACAGAAGACTACATGGTGCATTTGTTTGAAGACACGAATTTGTGT
GCCATCCATGCGAAGCGAGTCACCATAATGCCGAAAGATCTGCAGTTAGCCAGACGACTA
CGAGGTGTCATCGAAAAAACATCA

Bryum_argenteum
ATGGCGAGGAGGAAATCGACTCCTCGGCATGGCCGGAACCGGGCGTCCACGAGC---GCT
GCTGGTGGAGCTGCA------GCA??????------------???---------------
GCGCGGCAACGAAGGAGACCGCATCGATGGAGACCAGGCACCAAGGCACTCCAAGAGATT
CGGCATTACCAAAAGACATGCGACCTCCTCATACCTAGACTTCCGTTCGCGCGCTATGTG
AAAGAAATCACGTCTATGTATGCAAGTGATGTGTCTCGGTGGACGGCTGAAGCTTTAACA
GCCCTTCAAGAAGCAACTGAAGATTACATAGTGCATTTGTTTGAAGACACGAATTTGTGC
GCTATCCACGCTAAGCGAGTCACCATAATGCCAAAGGACTTGCAATTGGCGAGACGGCTA
CGCGGTGTGATTGAGAAAACAGTA

Ceratodon_purpureus
ATGGCGAGGCTGAAGCAGACGCCGCTGCGGAGCAACAACCGGGCGTCGACGAGC---TCT
GCTGGTCGAGCTGCA------CCCTCTCCTGCC---------GCTAGAGCGGCACAGCGC
GCGCCGCGGCCGAGGAAGCCGCACCGGTGGAGGCCTGGGACTAAGGCGTTGATGGAGATT
CGGTACTACCAGAAGACTTGCGACCTGCTCATCCCTCGCCTTCCTTTCGCTCGCTATGTG
AAGGAAATTACGTCTATGTACGCAAGTGATGTTTCTCGATGGACGGCAGAAGCGTTGACA
GCTCTTCAAGAGGCAACAGAAGACTACATAGTGCATTTGTTTGAGGACACAAATTTGTGT
GCTATCCATGCAAAGCGAGTCACCATAATGCCGAAGGATCTGCAGTTGGCCAGACGCCTG
CGGGGTGTCATCGAGAAAGCATCA

Hedwigia_ciliata
ATGGCGAGGCGGAAGTCTACTCCCCTGCATGGCCGCAGACGCGCGTCCACTAGC---TCT
GCAGGGGGAGCTGCA------??????---------------???---------------
GCGCGGAGGCCTCGGAAACCGCACAGATGGAGACCAGGCACCAAGGCATTGCAAGAGATC
CGCCACTACCAGAAGACTTGCGACTTGCTTATTCCTCGACTTCCTTTTGCTCGCTATGTG
AAAGAAATCACGTCTATGTATGCAAGTGACGTTTCTCGATGGACGGCGGAGGCTTTGACA
GCGCTTCAAGAGGCAACAGAAGACTACATGGTGCATTTATTTGAAGACACAAATTTGTGT
GCCATCCATGCGAAGCGAGTCACCATAATGCCGAAGGATCTGCAGTTGGCCAGACGACTA
CGAGGTTTCATCGAAAAAGCATCA

Leucodon_brachypus
ATGGCGAGGCGGAAGTCGACCCCTCTGCACGGAGAAAAACGCGCACCTACGCGC---TCG
---------???GCT------GCTGCTGCTGCT---------TCT---------------
ACGACGAGGCCCAGGAAAGCGCACCGATGGAGACCAGGCACCAAGGCGTTGCAGGAGATT
CGGCACTACCAGAAGACGTGTGACTTGCTCATCCCCCGACTGCCGTTTGCTCGCTATGTG
AAGGAAATCACGTCTATGTATGCGAGTGACGTCTCTAGATGGACGGCGGAGGCTTTGACA
GCCCTTCAAGAGGCAACAGAAGACTACATGGTGCACTTCTTTGAAGACACTAATTTGTGT
GCCATCCATGCGAAGCGAGTCACCATAATGCCGAAGGATCTGCAGTTAGCCAGACGACTA
CGAGGAGTCATCGAAAAAGCATCA

Physcomitrella_patens
ATGGCAAGAAGGAAAACTACCCCTGTACATGGCAACCACCGAGCTTCTACTAGC---TCT
GTTGGTGGAGCTGCG------???-----------------------------------
------AGGCCCAGGAAGCCGCACCGATGGAGACCCGGGACCAAGGCATTGCAAGAGATC
CGCCATTATCAAAAGACGTGCGACCTTCTCATCCCTCGACTACCTTTTGCCCGCTATGTG
AAGGAAATTACGATGATGTACGCTAGTGATGTTTCCCGGTGGACTGCGGAAGCTTTGACC
GCTCTCCAAGAGGCCACTGAAGATTATATGTGCCATTTGTTTGAAGACACCAATTTGTGT
GCTATCCATGCTAAGCGAGTAACCATAATGCCGAAGGATCTGCAGTTGGCCAGACGACTA
CGAGGTGCCATT????????????

Pohlia_nutans
ATGGCGAGGCGGAAGTCCACCCCTCTGCACGGCAACAGACGCGCCTCCACGAGC---TCC
---------------AATCCTGCTGCTGCTGCT---------GCT---------------
CAGCGGAGGCCTCGGAAACCGCACCGATGGAGACCCGGTACCAAGGCGTTGCAAGAGATT
CGCCAATACCAGAAGACTGGCGACTTGCTTATCCCCCGCCTCCCGTTTGCTCGCTACGTG
AAGGAAATCACGTCAATGTATGCAAGTGACGTTTCTCGATGGACGGCGGAGGCTTTGACA
GCCCTTCAAGAGGCAACAGAAGACTACATGGTGCATTTGTTTGAAGACACAAATTTGTGT
GCCATCCATGCGAAGCGAGTCACCATAATGCCGAAAGATCTGCAGTTGGCCAGACGACTA
CGAGGTGTCATCGAAAAAGCATCA

Rhynchostegium_serrulatum
ATGGCGAGGCGGAAGTCGACTCCTCTGCACGGCAACAAACGCGCACCCACGCGCCCATCT
---------???GCT------GGTGCTTCTTCT---------TCT---------------
ACGACGAGGCCGAGGAAAGCGCACCGATGGAGACCGGGCACCAAGGCGTTGCAGGAGATT
CGGCACTACCAGAAGACGTGCGACTTGCTCATCCCACGACTGCCGTTTGCTCGCTATGTG
AAGGAAATCACGTCAATGTATGCGAGTGACGTTTCTCGATGGACGGCGGAGGCTTTGACA
GCCCTTCAAGAGGCAACAGAAGACTACATGGTGCATTTGTTTGAAGACACAAATTTGTGT
GCCATCCATGCGAAGCGAGTCACCATAATGCCGAAGGATCTGCAGTTAGCCAGACGACTA
CGAGGTGTCATCGAAAAATCATCA

Rosulabryum_capillare
ATGGCGAGGAGGAAATCGACTCCTCGGCATGGCCGCAACCGGGCGTCCACGAGC---GCT
GCTGGTGGAGCTG?A------GCAGCTGCTGCT---------GCT---------------
GCGCGGCAACGAAGGAGACCGCATCGATGGAGACCAGGCACCAAGGCACTCCAAGAGATT
CGGCATTACCAAAAGACGTGCGACCTCCTCATTCCTAGACTTCCGTTCGCGCGCTATGTG
AAAGAAATCACGTCTATGTACGCAAGTGATGTGTCTCGGTGGACGGCGGAAGCTTTAACA
GCCCTTCAAGAAGCAACTGAAGACTACATAGT?CATTTGTTTGAAGACACGAAT??????
????????????????????????????????????????????????????????????
????????????????????????

Thuidium_delicatulum
ATGGCGAGGCGGAAGTCGACCCCTCTGCACGGCAACAAACGCACTCCCACTCGC---TCA
---------???GCC------GCTGCTGCTGCT---------TCT---------------
ACGTCGAGGCCGAGGAAAGCGCACCGATGGAGACCGGGCACCAAGGCGTTGCAGGAGATT
CGGCACTACCAGAAGACGTGCGACTTGCTCATCCCCCGACTGCCGTTTGCTCGCTATGTG
AAGGAAATCACGTCTATGTACGCAAGTGACGTTTCTAGATGGACGGCGGAGGGT?T?ACA
GCCCTTCAAGAGGCAACAGAAGACTACATGGTGCATTTGTTTGAAGACACAAATTTGTGT
GCCATCCATGCGAAGCGAGTCACCATAATGCCGAAGGATCTGCAGTTAGCCAGACGACTA
CGAGGTGTCATCGAAAAAGCATCA

;
end;

BEGIN TREES;
          TREE tree = (Anomodon_attenuatus,(Rhynchostegium_serrulatum,(Leucodon_brachypus,Thuidium_delicatulum)),((Ceratodon_purpureus,(Physcomitrella_patens,(Bryum_argenteum,Rosulabryum_capillare))),(Hedwigia_ciliata,Pohlia_nutans)));
END
```

Colletotrichum

```
#NEXUS
[ Title ]
begin taxa;
    dimensions ntax=7;
    taxlabels
        Colletotrichum_eremochloae
        Colletotrichum_falcatum
        Colletotrichum_higginsianum
        Colletotrichum_somersetensis
        Colletotrichum_graminicola
        Colletotrichum_sublineola
        Colletotrichum_zoysiae
;
end;
begin characters;
    dimensions nchar=402;
    format missing=? gap=- matchchar=. datatype=nucleotide;
    matrix

Colletotrichum_eremochloae
ATGCCTCCCAAGAAGAGACAGTCAGACGCTCCGCGTCGGAAATCTCGTCAGAGCGACGTC
CAACCTGGCGACCCTATCCCCAACCGAGGAAGGCGACGTTACAAGCCCGGTACAGTTGCT
CTCAGGGAGATCCGAAAGTATCAAAGTGGCACTCAGCTTCTGCTGCGCCAGCTTCCATTC
TCTCGCCTTGTACGCGAGATCGCCGAATCGGTGAGACCCCGAGGCGAAGCGATGCGATGG
CAATCACAAGCGATACAAGCACTACAAGAAGCTGCGGAGGCTTTCTTGGTACACCTCTTC
GAAGACACCAACCTTTGCGCTATCCACGCCAAGAGAGTAACCATAATGCAAAAAGATATT
CAGCTCGCAAGGCGCATTCGGGGCGTCTGGGGTGGCCTAGGT

Colletotrichum_falcatum
ATGCCCTCCAAGAAGAGACAGTCAGACGCTCCGCGTCGGAAATCCCGTCAGAGCGACGTT
CAACCTGGTGATCCTATTCCCAACCGAGGAAAGCGACGCTACAAGCCTGGTACAGTTGCT
CTCAGGGAGATCCGAAAGTACCAAAGTGGCACTCAACTTTTGCTGCGTCAACTTCCATTC
TCCCGCCTTGTACGCGAGATTGCCGAGTCGGTGAGACCCCGAGGCGAAGCAATGCGATGG
CAATCACAAGCGATACAAGCACTACAAGAAGCTGCGGAGGCTTTCTTGGTACACCTCTTC
GAAGACACCAACCTCTGCGCTATCCACGCCAAGAGAGTAACCATAATGCAAAAAGATATT
CAGCTCGCAAGGCGCATTCGGGGCGTCTGGGGTGGGCTAGGC

Colletotrichum_higginsianum
ATGCCTCCTAGGAAGAGACAGTCGGACGCTTCACGTCGAAAATCGCGCCAGAGTGATGTA
CAACCTGGTGACCCGATTCCCAACCGAGGCAAGCGACGCTACAGACCCGGCACAGTTGCT
CTCAGGGAAATCCGAAAATACCAGAGCGGCACCCAGCTTTTGCTGCGTCAGCTTCCCTTC
TCTCGACTTGTCCGCGAAATTGCCGAGTCGGTGAGACCCCGGGGAGAGGCGATGCGGTGG
CAATCACAAGCAATACAGGCACTTCAAGAAGCCGCCGAGGCTTTCTTGGTTCACCTATTT
GAAGATACAAACCTCTGCGCTATCCACGCCAAGAGAGTGACCATTATGCAAAAGGATATT
CAGCTTGCCAGGCGCATTCGGGGCGTCTGGGGTGGATTGGGT

Colletotrichum_somersetensis
ATGGCCCCCAAGAAGAGACTGTCAGACGCTCCGCGTCGGAAATCCCGTCAAAGCGACGTA
CAACCTGGCGATCCTATTCCCAACCGAGGAAAGCGACGCTACAAGCCTGGTACAGTTGCC
CTCAGGGAGATCCGAAAGTATCAAAGTGGCACTCAGCTTCTGCTGCGCCAACTTCCATTC
TCCCGGCTTGTACGCGAAATTGCCGAGTCGGTGAGACCCCGAGGCGAAGCAATGCGATGG
CAATCACAAGCGATACAGGCGCTCCAAGAAGCTGCAGAGGCCTTCTTGGTACACCTCTTC
GAAGACACCAACCTCTGCGCTATCCACGCCAAGAGAGTAACCATAATGCAAAAAGATATT
CAACTCGCGAGGCGCATTCGGGGCGTCTGGGGTGGGCTAGGC

Colletotrichum_graminicola
ATGGCCCCCAAAAAGAGACATTCAGACGCTTCGCATCGGAAATCCCGTCAGAGCGACGTG
CAACCCGGTGACCCTATTCCCAACCGAGGAAAGCGACGCTACAAGCCTGGTACAGTCGCT
CTCAGGGAGATCCGAAAGTATCAAAGTGGCACTCAGCTTCTGCTGCGTCAACTTCCATTC
TCTCGTCTTGTACGCGAAATTGCCGAGTCGGTGAGACCCCGAGGCGAAGCAATGCGATGG
CAATCGCAAGCGATACAGGCACTCCAAGAAGCTGCGGAGGCCTTCTTAGTACACCTCTTC
GAAGACACCAACCTCTGCGCTATCCATGCCAAGAGAGTAACCATAATGCAAAAAGATATT
CAGCTCGCAAGGCGCATTCGGGGCGTCTGGGGTGGGCTAGGC

Colletotrichum_sublineola
ATGCCTCCCAAGAAGAGACAGTCAGACGCTCCGCGTCGGAAATCTCGTCAGAGCGACGTC
CAACGTGGCGACCCTATCCCCAACCGAGGAAGGCGACGTTACAAGCCCGGTACAGTTGCT
CTCAGGGAGATCCGAAAGTATCAAAGTGGCACTCAGCTTCTGCTGCGCCAGCTTCCATTC
TCTCGCCTTGTACGCGAGATCGCCGAATCGGTGAGACCCCGAGGCGAAGCGATGCGATGG
CAATCACAAGCGATACAAGCACTACAAGAAGCTGCGGAGGCTTTCTTGGTACACCTCTTC
GAAGACACCAACCTTTGCGCTATCCACGCCAAGAGAGTAACCATAATGCAAAAAGATATT
CAGCTCGCAAGGCGCATTCGGGGCGTCTGGGGTGGCCTAGGT

Colletotrichum_zoysiae
ATGGCCCCCAAGAAGAGACAGTCAGACGCTCCGCGTCGGAAATCCCGTCAAAGCGACGTA
CAACCTGGCGACCCTATTCCCAACCGAGGAAAGCGACGCTACAAGCCTGGCACAGTTGCC
CTCAGGGAGATCCGAAAGTATCAAAGTGGCACTCAGCTTCTGCTGCGCCAACTTCCATTC
TCCCGGCTTGTACGCGAAATTGCCGAGTCGGTGAGACCCCGAGGCGAGGCAATGCGATGG
CAATCACAAGCGATACAGGCGCTCCAAGAAGCTGCAGAGGCCTTCTTGGTACACCTCTTC
GAAGACACCAACCTCTGCGCTATCCACGCCAAGAGAGTAACCATAATGCAAAAAGATATT
CAGCTCGCGAGGCGCATTCGGGGCGTCTGGGGTGGGCTAGGC

;
end;

BEGIN TREES;
            TREE tree = (Colletotrichum_graminicola,(Colletotrichum_somersetensis,Colletotrichum_zoysiae),(Colletotrichum_higginsianum,(Colletotrichum_falcatum,(Colletotrichum_eremochloae,Colletotrichum_sublineola))));
END
```

Drosophila

```
#NEXUS
[ Title ]
begin taxa;
     dimensions ntax=16;
     taxlabels
          erecta
          lucipennis
          lutescens
          mauritiana
          melanogaster
          mimetica
          orena
          paralutea
          prostipennis
          rajasekari
          sechellia
          simulans
          takahashii
          teissieri
          trilutea
          yakuba
;
end;
begin characters;
     dimensions nchar=888;
     format missing=? gap=- matchchar=. datatype=nucleotide;
     matrix

erecta
ATGCCCCGGCACAATGCTGCCAAGCGT---------AACCCC------------------
AACCCATCAATG------------------AACAACTCAAAGCCTCCAAGCGACGACGAC
AAAGCATTCCGCTCGCCGGAGCCAGAAGACGACACCGACTACGGCCTGGAGTTCACCACC
AGCCAACTGACGCTACAGGAGGGC---TACAATCGTCGTTGCTCCACGATGCGC???---
------------------------------------CAGCCAGCGACCCGCACTTACACC
????????????CAG???GAC------CAGGAGAATCGCCATCCGGCAGCCAGATCAAAG
CAGACGCGTCCGACACCC????????????------------------------------
------------------------------------------------------------
------ACTAGCTCGCGGGAA---------GCGGGACCATTTGCTGCACAG------AAC
CAG------ACCAGGCGGCGCAAAATGGCCAATCCCATAAGCAGAGCCAAGCGGATGGAT
CTCGAGATACGGCGACTGCAGAACCATTCCGGCACACTGATACCCAAGCTGCCGTTCTCG
CGTCTGGTGCGCGAGTTCATCGTGAAGTACAGCGATGGAGAGCCGTTGAGGGTCTCCGAG
GGCGCCTTTATGGCCATGCAGGAGTCCTGCGAGATGTACGTGACGCAGCGGCTGTCGGAC
TCCTACATGCTAACCCGGCATCGCAATCGCGTGACACTGGAGGTGCGCGACATGGCTCTG
ATGGCCTACCTTTGCGACCGGGGT------CGGCTGGCC---------

lucipennis
????????????????????????????????????????????????????????????
????????????????????????????????????????????????????????????
????????????????????????????????????????TACGGCTTGGAGTTCACCACC
AGCCGCCTGACGCTGCAAGAGTCC---TCCAATCGCCGCTGCTCCACGATGCGCAAAGAC
???---???GGCAGAAGATCACGT---------------???CCGGCTACGGCATCGTCG
AGC---GACGAG---GAGGAT------CAGGAAAACCACCAACCGGCAGCGAGATCGCCG
CAGACCCGTCGAATGACAAGTCTTCAGGGGAGT????????????---------------
------------?????????--------------------------GCCAGGACCCAG
GCC???------------???TCAGGT???---??????????????????CAGCAG???
???------CCCCGCCGCCGCAAGGCCGTCAATCCCATG???---------???ATGGAG
CGCGAAATCAGGCGGCTACAGGCCCATCCCGGC???????????????????????????
????????????????????????????????????????????????????????????
????????????????????????????????????????????????????????????
????????????????????????????????????????????????????????????
????????????????????????????????????????????????

lutescens
ATGCCGAGAAAAAGTGGGGCAAACCGCAAGGTGAAC???CCC------------------
AAGCCAACG???GGGGACACGGACGCGGGG------------------AGCGACGACGAC
ACGGCCTTCCGATCGCCCGAACCGGAAGATGGCACCGACTACGGCTTGGAGTTCACCACA
AGCCGGCTGACTCTGCAGGACTCC---TCCAATCGCCGTTGCTCCACGCTGCGCAAGGAC
ACCAGAAATGACAGG------------------------???GCGGATGGAACCGCCTCC
AGC---GAAGAG---GAGGAT------CAGGAGAATCGCCTGCCAGCAGCCAGATCGCCG
CAGACGGGACGGATGACCAGTCAGCAG???AGG????????????AGCCAGCAAGAGAGG
CGTCCTGCTCCC????????????AGGCGTCCGGCTCCCAGCCAGCCGGTTAGGACTCAA
GCATCC------------???------ACC---GGACCAGCTGCTGGCCAG------GAT
CGG------CACCGCCGACGCAAGATGGCCAACCCCATAAGCAGAGCCAAGCGAATGGAT
CGCGAGATCCAAAATCTGCAGAAACATCCCGGCACGCTTATACCCAAGCTGCCGTTCTCG
CGTCTGGTTCGCGAATTCATCGTCAAGTACAGCGACGGAGAACCGTTTAGGGTCACCGAA
GGCGCCTTCAACGCCCTGCAGGTGTCCAGCGAGATGTACGTGACGCAGCGCCTGGCCGAC
TCCTACATGCTGACCAAGCACCGCAATCGCGTCACGCTGGAGGTGCGCGACATGGCTCTG
ATGGCTTTGATCTGCGACCGGGGTCACAAC------------------

mauritiana
ATGCCACGACACAGTAGAGCCAAACGC---------GCACCC------------------
AGGCCGTCGGCG------------------AACAACTCAAAGTCGCCGAACGACGACGAC
ACGGCCTTCCACTCGCCGGAGCCAGAAGACGGCACCGACTACGGCCTCGAGTTCACCACC
AGCCAACTGACGCTTCAG???------AGCAATCGGCGTTCCTCGACGATGCGCAGGGAC
GCC---------------------GGACGGAGGCAGTCGCCAACGAATGGTAGCTCCGCC
AGTGGC??????---???GAC------CAGGAGAATCGCCATCCCAGAGCCAGACCGTCG
CAGACGCGTCGATTGACC????????????------------------------------
------------------------------------------------------------
------AGCAAGACGCGGGCA---------GCGGGGCCAGTTGCTTGCCAA------AAC
CAG------ACCAGGCGGCGCAAAGCGGCCAATCCCATGAGCAGAGCCAAGAGGATGGAT
CGCGAGATCCGGCGACTGCAGCACCATCCCGGCACACTGATACCCAAGCTGCCGTTCTCG
CGTCTGGTGCGGGAGTTTATCATGAAGTACAGCGACGGCGAGCCGCTGAGGGTCACCGAG
GGCGCCCTATTGGCCATGCAGGAGTCCTGCGAGATGTACTTGACGCAGCGGCTCGCGGAC
TCCTACATGCTAACCAAGCATCGCAATCGCGTCACACTGGAGGTGCGCGACACGGCATTG
ATGGCCTACATCTGCGACCGGGGT------CGGCAAGCT---------

melanogaster
ATGCCACGACACAGCAGAGCCAAGCGC---------GCACCC------------------
AGGCCGTCGGCG------------------AACAACTCAAAGTCGCCGAACGACGACGAC
ACGGCCTTCCGCTCGCCGGAGCCAGAAGACGGCACCGACTACGGCCTCGAATTCACCACC
AGCCAACTGACGCTTCAG???------AACAATCGGCGTTCCTCGACGTTGCGCAGGGAC
GCC---------------------GGACGGAGGCAGCCGGCAGCGAGAGACAGCTCCACC
AGTGGC??????---???GAC------CAGGAGAATCGCTATCCCACAACCAGATCGCCG
CAGACGCGTCGAATGACC????????????------------------------------
------------------------------------------------------------
------AGCAAAACGCGAGCA---------GCAGGGCCAGTTGCTGCGCAA------AAC
CAG------ACCAGGCGGCGCAAAGCGGCCAATCCCATGAGCAGAGCCAAGAGAATGGAT
CGTGAGATCCGGCGACTGCAGCACCATCCCGGCACACTGATACCCAAGCTGCCGTTCTCG
CGTCTAGTGCGCGAGTTCATCGTGAAGTACAGCGACGACGAGCCGCTAAGGGTCACCGAA
GGCGCCCTATTGGCCATGCAGGAGTCGTGCGAGATGTACTTGACGCAGCGGCTCGCCGAC
TCCTACATGCTAACCAAGCATCGCAATCGCGTCACACTGGAGGTGCGCGACATGGCATTG
ATGGCCTACATCTGCGACCGGGGT------CGGCAATTT---------

mimetica
ATGCCGAGAAAAAGTGGCGCAAGCCGCAAGATGCCC???CCC------------------
AAGCCAACG???GGGGACACGGACGCGGAG------------------AGCGACGACGAC
ACGGCTTTCCGATCGCCGGAACCGGAAGATGGCACCGACTACGGCCTGGAGTTCACCACA
AGCCGACTGACTCTACAGGATGCC---TCCAATCGCCGTTGCTCAACGATGCGCAAGGAC
ACCCGAAACACCCGGGCATCGCGA---------------???GCGGATAGAACCGCCTCA
AGC---GAAGAG---GAGGAT------CAGGAGAATCGCCTTCCTGCAGCCAGATCGCCG
CAGACGCGACAGATGACCAGTCAGCAGGAGAGG????????????---------------
------------?????????--------------------------GTCAGGGTCCAA
GCATCT------------???------GCC---GGACAA---???GTCCAG------AAT
CAA------ACCAGGCGACGTAAGATGGCCAATCCCATGAGCCGAGCCCAGAGAATGGAT
CGCGAGATCCGACATCTGCAGAATCATCCCGGCATACTCATCCCTAAGCTGCCATTCTCG
CGCCTGGTTCGCGAATTTATCATGAAGTACAGCGACGGAGCTCCGTTGAAGGTCACTGAA
GGCGCCCTCAACGCCATGCAGGTGTCCAGCGAGTTGTATGTGACGCAACGTCTGGCCGAC
TTCTACATGCTAACCAAACACCGCAATCGCGTCACGCTGGAAGTGCGCGACATGGCTTTG
ATGGCTCTGATCTGTGACCGGAGTCATAAC------------------

orena
ATGCCTCGACACAGTGATGCCAAGCGT---------AACCCTAAATCGAACCCCAAATCG
??????AAAATG------------------AACAACTCACAGCCGCCAAGCGACGACGAC
ACAGCATTCCGCTCGCCGGCTCCAGAAGGCGACACCGACTACGGCCTGGAGTTCACCACT
AGCGAACTGACGCTACAGGAGGGC---AACAATCGTCGTTGCTCCACGATGCGCAAGGAC
GCC---------------------GGACATAGGCATCAGCCAGCGACCCGCACCTACTCC
????????????---???AAC------CAGGAGAATCGCCATCCGGCAGCCACATCCAAG
CAGACGCATCCGAAA---------------------------------------------
------------------------------------------------------------
------------------------------------------------------------
------------CGGCGGCGCAAAATGAACAATCCCATAAGCAGAGTCAAGAGGGTGAAC
CTCGAGATACGGCGATTGCAAAGCCATTCCCGCGCACTGATACCTAAGCTGCCCTTCTCG
CGTCTGGTGCGCGAATTCATCATGAAGTACAGCGATGGGGAGCCGTTGAAGATCAGCGTG
GCAGCCTTAGTGGCCATACAGGAGTCCTGCGAGCTGTACGTGACACAGCGGCTGTCGGAC
TCCTACATGCTAACCAGGCATCGCAATCGCGTCACACTTGAGGTGCGCGACATGGCTCTG
ATGGCCTACATCTGCGAGCGGGCT------CGGCTAGCC---------

paralutea
????????????????????????????????????????????????????????????
????????????????????????????????????????????????????????????
????????????????????????????????????????TACGGCTTGGAGTTCACCACA
AGCCGGCTGACTCTGCAGGACGCC---TCCAATCGCCGTTGCTCGACGCTGCGCAAGGAC
ACCAGAAATGGCCGGGCATTGGAA---------------???GCGGATAGAACCGCCTCC
AGC---GAAGAG---GAGGAT------CAGGAGAATCGCCTGCCAGCAGCCAGATCGCCG
CAGACGCGACGGATGACCAGCCAGCAGGAGAGGCGTCCGGCTCCC---------------
------------AGCCAGCAGGAGAGGCGTCCGGCTCCCATCCAGCCGGTTAGGGTTCAA
GCATCC------------???------ACC---GGACCAGCGGCTGGCCAG------GAT
CGG------AAACGGCGACGCAAGATGGCCAATCCCATAAGCAGAGCCAAGCGAATGGAT
CGCGAGATCCAACGTCTGCAGAAACATCCCGGCACGCTTATACCCAAGCTGCCATTCTCG
CGTCTGGTTCGCGAATTTATAGTCAAGTACAGCGATGGACCACCGTTCAGGGTCACCGAA
GGCGCCTTCAACGCCATGCATGTGTCCAGCGAGATGTACGTGACGCAGCGTCTGGCCGAC
TCCTATATGCTGACCAAACACCGCAATCGCGTCACGCTGGAGGTGCGCGACATGGCTCTG
ATGGCTTTGATCTGCGACCGGGGTCATAAC------------------

prostipennis
ATGCCGAGAAAAAGTGGCGCAAGCCGCAGGGTGAAC???CCC------------------
AAGCCAACG???GGGGACACGGACGCGGAG------------------AGCGACGACGAC
ACGGCCTTCCGATCGCCAGAACCGGAAGATGGCACCGACTACGGCTTGGAGTTCACCACA
AGCCGGCTGACTCTGCAGGACGCC---TCCAATCGCCGTTGCTCGACTCTGCGCAAGGAC
ACTAGAAATGGCCGGGCATTGGGA---------------???GCCGACAGAACCGCCTCC
AGC---GAAGAG---GAGGAC------CAGGAGAATCGCCTGCCAGCAGCCAGATCGCTG
CAGTCGAGTCGCATAACCAGTCAGCAGGAGATGCGTCCTGCTCCC---------------
------------AGCCAGCAG--------------------------GTCAGGACTCAG
GCATCT------------???------TCA---GGACAAGCTGCTCCTCAG------GAT
CGG------AACCGGCGACGCAAGATGGCCAATCCCATAAGCAGAGCCCAGCGAATGGAT
CGCGAGATCCAAAATCTGCAGAAACATCCCGGC???????????????????????????
????????????????????????????????????????????????????????????
????????????????????????????????????????????????????????????
????????????????????????????????????????????????????????????
????????????????????????????????????????????????

rajasekari
????????????????????????????????????????????????????????????
????????????????????????????????????????????????????????????
????????????????????????????????????GACTACGGCTTGGAGTTCACCACA
AGCCGGCTCACCCTG---------AATTCCAACCGCCGATGCTCGACGCTGCACAAGGAG
ACCTCAGGCGGCCCTGGGTCGCGA---------------???GCGGGAAGAACT---???
AGC---GATGAG---GAGGAT------CAGGAGAATCGG---??????GCCAGCTCGCCG
GGGACGCGCAGGATGAGCGGTCAGCAGGAAAGG????????????---------------
------------?????????--------------------------TCCAGGACTCAG
GCAGCG------------???------AAC---GGATCGATTGGTGCCCAG------AAT
CAG------ACCAGGAGACGCAAGATGGCCAAACCCTTGAGCAGAGCCAGAAGGATGGAT
CTAGAGATCCAGCATCTGCAGAATCATCCCGGC???????????????????????????
????????????????????????????????????????????????????????????
????????????????????????????????????????????????????????????
????????????????????????????????????????????????????????????
????????????????????????????????????????????????

sechellia
ATGCCACGACACAGTAGAGTCAAGCGC---------GCACCC------------------
AGGCCGTCGGCG------------------AACAACTCAAAGTCGCCGAACGACGATGAC
ACAGCCTTCCGCTCGCCGGAGCCAGAAGACGGCACCGACTACGGCCTCGAGTTCACCACC
AGCCAACTGACGCTTCAG???------AACAATCGGCGTTCCTCGACGATGCGCAGGGAC
GCC---------------------GGACGGAGGCAGTCGCCAACGAATGGTAGCTCCGCC
AGTGGC??????---???GAC------CAGGAGAATCGCCATCCCAGAGCCAGACCGTCG
CAGACGCGTCGATTGACC????????????------------------------------
------------------------------------------------------------
------AGCAAGACGCGGGCA---------GCAGGGCCAGTTGCTGCGCAG------AAC
CAG------ACCAGGCGGCGCAAAGCGGCCAATCCCATGAGCAGAGCCAAGAGGATGGAT
CGCGAGATCCGGCGACTGCAGCACCATCCCGGCACACTGATACCCAAGCTGCCGTTCTCG
CGTCTGGTGCGGGAGTTTATCATGAAGTACAGCGACGGCGAGCCGCTGAGGGTCACCGAG
GGCGCCCTAATGGCCATGCAAGAGTCCTGCGAAATGTACTTGACGCAGCGGCTCGCGGAC
TCCTACATGCTAACCAAGCATCGCAATCGCGTCACACTGGAGGTGCGCGACATGGCATTG
ATGGCCTACATCTGCGACCGGGGT------CGGCAAGCT---------

simulans
ATGCCACGACACAGTAGAGCCAAGCGC---------GCACCC------------------
AGGCCGTCGGCG------------------AACAACTCAAAGTCGCCGAACGACGATGAC
ACAGCCTTCCACTCGCCGGAGCCAGAAGACGGCACCGACTACGGCCTCGAGTTCACCACC
AGCCAACTGACGCTTCAG???------AACAATCGGCGTTCCTCGACGATGCGCAGGGAC
GCC---------------------GGACGGAGGCAGTCGCCAACGAATGGTAGCTCCGAC
AGTGGC??????---???GAC------CAGGAGAATCGCCATCCCAGAGCCAGACCGTCG
CAGACGCGTCGATTGACC????????????------------------------------
------------------------------------------------------------
------AGCAAGACGCGGGCA---------GCAGGGCCAGTTGCTGCGCAG------AAC
CAG------ACCAGGCGGCGCAAAGCGGCCAATCCCATGAGCAGAGCCAAGAGGATGGAT
CGCGAGATCCGGCGACTGCAGCACCATCCCGGCACACTGATACCCAAGCTGCCGTTCTCG
CGTCTGGTGCGGGAGTTTATCATGAAGTACAGCGACGGCGAGCCGCTGAGGGTCACCGAG
GGCGCCCTGTTGGCCATGCAAGAGTCCTGCGAAATGTACTTGACGCAGCGGCTCGCGGAC
TCCTACATGCTAACCAAGCATCGCAATCGCGTCACACTGGAGGTGCGCGACATGGCATTG
ATGGCCTACATCTGCGACCGGGGT------CGGCAAGCT---------

takahashii
ATGCCGAGAAAAAGTGGCGCAAGCCGCAAGGTGGAC???CCC------------------
AAGCCGACG???GGTGACACGGACGCGGAG------------------AGCGACGACGAC
TCGGCCTTCCGATCGCCGGAACCGGAGGACGGCACCGACTACGGCTTGGAGTTCACCACC
AGCCGGCTGACTCTGCAGGACGCC---TCCAATCGCCGTTGCTCGACGCTGCGCAAGGAC
GCCAGAAGTGGCCGGGCATTGGAA---------------???GCGAATAGAACAGCCTCC
AGC---GAGGAG---GAGGAT------CAGGAGAATCGACTGCCAGCAGCCAGATCGCCG
------CGACGGATGACCAGTCAGCAGGAAAGGCGTCCAACTGCC---------------
------------AGCCAGCAGGAGAGGCGTCCAACTGCCAAC---------------CAA
GCATCT------------???------TTA---GGACCAGCTGCTCCTCAG------GAT
CGT------AGGCGGCCACGCAAGATGGCCAATCCCATGAGCAGAGCCCGGCGAATGGAT
CGCGAGATCCAACAACTGCAGAAACATCCCGGCACGCTTATACCCAAGCTGCCATTCTCG
CGCTTGGTTCGCGAATGTATCATGAAGTACAGCGACGGAGCACCGCTGAAGATCACCGAG
GGCGCCTTCAACGCCCTGCAGGTGTCCAGCGAGATGTACGTGACGCAGCGCCTGGCCGAC
TCCTACATGCTGACCAAGCACCGCAATCGCGTCACGCTGGAGGTGCGCGACATGGCTCTG
ATGGCTCTGATCTGCGACCGGGGTCACAAC------------------

teissieri
ATGCCTCGACACAGTAACGCCAAGCGC---------GGCCCC------------------
AAATCGACGGTG------------------AACAACTCAAAACCGCCAAGCGACGACGAC
ACAGCCTTCCGCTCGCCGGAGCCAGAAGACGGCACCGACTATGGCCTGGAGTTCACCACC
AGCCAACTGACGCTACAGGAG------AACAGTCGTCGTTGCTCGACGATGCGCAAGGAC
GCC---------------------GGACGGCGGCATCCGGCGGCGAGTAGGATGTCCACC
AGCGAC??????---??????CAGGAT???GAAAATCGCCATCCGGCAGCCAGATCACCG
CAGACGCGTCGGATGACC????????????------------------------------
------------------------------------------------------------
------ACCAGCACGCGGGCG---------CCGAGACCAGTTGCTGCACAG------AAC
???AACCAAAACAGGCGGCGCAAAATGCCCAATCCCATAAGCAGAACGAAAAGAATGGAT
CGCGAGATACGGCGACTGCAGAACCATCCCGGCACACTAATACCCAAGCTGCCGTTCTCG
CGTCTGGTGCGCGAGTTCATCGTGAAGTACAGCGATGGAGAACCGCTGAGGGTCACCGAG
GGCGCCTTTATGGCCATGCAGGAGTCCTGCGAGATGTACCTGACGCAGCGGCTCGCGGAC
TCCTACATGCTAACCAAGCATCGCAATCGCGTTACACTGGAGGTGCGCGACATGGCCCTG
ATGGCCTACATCTGCGACCGGGGT------CGGCTAGCA---------

trilutea
????????????????????????????????????????????????????????????
????????????????????????????????????????????????????????????
????????????????????????????????????????????????????????????
????????????????????????????????????????????????????????????
????????????????????????????????????????????????????????????
??????GAAGAG---GAGGAT------CAGGAGAATCGCCTGCCACCAGCCAGATCGCCG
CAGACGCGACGGATGACCAGTCAGCAGGAGAGGCGACCGGCTCCC---------------
------------AGCCAGCAGGAGAGGCGTCCGGCTTCGAGCCAGCCGGTTAGCAACCAA
GCATCC------------???------ACC---GGACCAGCTGCTGGCCAG------GAT
CGG------AAACGGCCACGCAAGATGGCCAATCCCAGAAGCAGAGCCCAGCGAATGGAT
CGCGAGATCCAACGTCTGCAGAAACATCCCGGCACGCTTATACCCAAGTTGCCATTCTCG
CGTTTGGTTCGCGAATTTATCGTCAAGTACAGCGACGGAGAACCGTTTAGGGTCACCGAA
GGCGCCTTCAACGCCATGCAGGTGTCCAGCGAGATGTACGTGACTCAGCGTCTGGCCGAC
TCCTACATGCTGACCAAACACCGCAATCGCGTCACGCTGGAGGTGCGCGACATGGCTCTG
ATGGCTTTGATCTGCGACCGGGGTCATAAC------------------

yakuba
ATGCCACGACACGGGAACGCCAAGCGC---------GGCCCC------------------
AAATCGACGGTG------------------AACAATTCAAGGCCGCAAACCGACGACGAC
ACATGCTTCCGCTCGCCGGAACCAGAAGACGGCACCGACTACGGCCTGGAATTTACCACC
AGCCAACTGACGCTACGGGAGGAC---AACAGTCGTCGTTCCTCGACGATGCGCAAGGAC
GCC---------------------GAACGGAGGCATCCGCCAGCGAATAGGACCTCCACC
AGCGAC??????---??????GAGGAT???GAAAATCGCGGTCCGGCAGCCGCGTCGTCG
CAGTCGCGTCGGATGACC????????????------------------------------
------------------------------------------------------------
------ACCAGCACGCGGTCG---------CCGAGACCAGTTGCCGCACAG------AAC
???AACCAAACCAGGCAACGCAAAATGCCCAATCCCATAAGCAGGACCAATAGGTTGAAT
CGCGAGATACTGCGGCTGCAGAACCATCCCGGCACACTTATACCCAAGCTGACGTTTGCG
CGTCTGGTGCGCGAGTTCATCGTGAAGTACAGCGATGAAGCACCGTTGAGGGTCACCGAG
GGCGCGTTAATTGCCATGCAGGAGTCCTGCGAGATGTTCTTGACGCAGCGGCTCGAGGAC
TCCTACATGCTAACCAAGCATCGCAATCGCGTCACACTGGAGGTGCGTGACATGGCCCTG
ATGGCCTACATCTGCGACCGA???------???------TCGTCAAAT

;
end;

BEGIN TREES;
                    TREE tree = (erecta,orena,((teissieri,yakuba),(((mimetica,(prostipennis,(takahashii,(lutescens,(paralutea,trilutea))))),(lucipennis,rajasekari)),(melanogaster,(mauritiana,(sechellia,simulans))))));
END;
```

Fabaceae

```
#NEXUS
[ Title ]
begin taxa;
	dimensions ntax=17;
	taxlabels
		Arachis_appressipila
		Astragalus_sinicus
		Cajanus_cajan
		Cajanus_cajanifolius
		Cajanus_scarabaeoides
		Cicer_bijugum
		Cicer_judaicum
		Cicer_pinnatifidum
		Cicer_reticulatum
		Cicer_yamashitae
		Glycine_max
		Lens_culinaris
		Lotus_japonicus
		Medicago_truncatula
		Phaseolus_vulgaris
		Pisum_sativumB
		Vigna_unguiculata
;
end;
begin characters;
	dimensions nchar=585;
	format missing=? gap=- matchchar=. datatype=nucleotide;
	matrix

Arachis_appressipila
ATGGCAAGAGTGAAGCATATTCCAACACCTAGTCAAAAAGGT---AAGAAAAAA???---
------------------------------------------------------AGA
???------???------???TCTCCATCGCCA????????????GGTAGCAGA---???
---?????????????????????---??????---------------------CCA???
???GGACGACGA??????GCA???---------------AAGAAAAGGCGTAATAAGCCA
GGAACAGTAGCTCTTCGTGAGATTCGTAAATTTCAGAAGAGTTTCAACCTACTCATCCCA
GCTGCCCCCTTCATGAGATGTGTCAAACAGATTACAAACCAACTATCTACGGAGGTCAAT
CGCTGGACAGCTGAAGCCATGGTAGCACTTCAAGAAGCAGCTGAGGATCATCTGGTTCGT
TTGTTTGAAGATGGAATGTTGTGTGCTATCCATGCAAAGCGTGTTACTCTAATGAAAAAG
GACATAGAGTTGGCCCGGAGACTCGGAGTGATAGGAAGACCTTGG

Astragalus_sinicus
ATGGCGAGAGTTAAAATCAAACCATCTCCTCGTAAA------------------------
CGC------------------------???------------------------------
----------------------------------------------------------
----------------------------------------------------------
---------------------AGTGAGAGTCAGGGGAAGAAAAAAAGGCGCTATAAACCT
GGAACAGTAGCACTTCGTGAGATTCGTCATTTCCAAAAGTCTGTCAATTTACTCATACCG
GCTGCTCCTTTTATAAGATGTGTCAAACAGATTACAAATAACTTTTCTACAGAAGTCTCG
CGCTGGACACCTGACGCCGTGTTAGCACTTCAAGAGGCAGCTGAGGATTATCTGGTTAAA
TTGTTTGAAGACGGGATGCTCTGTGCAATTCATGCAAGGCGTGTTACCCTTTTGAAAAAG
GATTTTGAGTTGGCGCGGAGACTTGGAGGAATAGGAAGGCCTTGG

Cajanus_cajan
ATGGCGAGAGTGAAGCACACGCCAGCTTCTCGCAAGCCTGGTAGG??????AAAGCG???
----------------------------------------------------------
CCAGAATCACCG------CAA------------GCTCAATCGCCCGCAACTAGAGAG???
AGG???AGAGCTGAACAAGAGTTGCCGCAGGAGAAT---------------------GAA
GCA---------GCGGCA------???ACTCACGGAAGAAAGAAAAGGCGCAGTAAGCCG
GGAACTGCGGCGCTTCGCGAGATTCGTCATTATCAGAAGTCTTGCCAGCTTCTTATCCCA
GTCGCACCCTTTATTAGATGCGTCAAAGAGATAACGCATCAATACTCTACGGAGGTGTCT
CGTTGGACACCCGAAGCTGTGTTAGCACTTCAGGAGGCAGCTGAGGAATATCTGGTTCAC
TTGTTTGAAGACGGAATGCTCTGTGCAATTCATGCAAAGCGTGTTACTCTTATGAAAAAG
GATATTGAGTTGGCTCGGAGGCTTGGAGGAATAGGAAGGCCTTGG

Cajanus_cajanifolius
ATGGCGAGAGTGAAGCACACGCCAGCCTCTCGCAAGCCTGGTAGG??????AAAGCG???
----------------------------------------------------------
CCAGAATCACCG------CAA------------GCTCAATCGCCCGCAACTAGAGAG???
AGG???AGAGCTGAACAAGAGTTGCCGCAGGAGAAT---------------------GAA
GCA---------GCGGCA------???ACTCACGGAAGAAAGAAAAGGCGCAGTAAGCCG
GGAACTGCGGCGCTTCGCGAGATTCGTCATTATCAGAAGTCTTGCCAGCTTCTTATCCCA
GTCGCACCCTTTATTAGATGCGTCAAAGAGATAACGCATCAATACTCTACGGAGGTGTCT
CGTTGGACACCCGAAGCTGTGTTAGCACTTCAGGAGGCAGCTGAGGAATATCTGGTTCAC
TTGTTTGAAGACGGAATGCTCTGTGCAATTCATGCAAAGCGTGTTACTCTTATGAAAAAG
GATATTGAGTTGGCTCGGAGGCTTGGAGGAATAGGAAGGCCTTGG

Cajanus_scarabaeoides
ATGGCGAGAGTGAAGCACACGCCAGCTTCTCGCAAGCCTGGTAGG??????AAAGCG???
----------------------------------------------------------
CCAGAATCACCG------CAA------------GCTCAATCGCCCGCAACTAGAGAG???
AGG???AGAGCTGAACAAGAGTTGCCGCAGGAGAAT---------------------GAA
GCA---------GCGGCA------???ACTCACGGAAGAAAGAAAAGGCGCAGTAAGCCG
GGAACTGCGGCGCTTCGCGAGATTCGTCATTATCAGAAGTCTTGCCAGCTTCTTATCCCA
GTCGCACCCTTTATTAGATGCGTCAAAGAGATAACGCATCAATACTCTACGGAGGTGTCT
CGTTGGACACCCGAAGCTGTGTTAGCACTTCAGGAGGCAGCTGAGGAATATCTGGTTCAC
TTGTTTGAAGACGGAATGCTCTGTGCAATTCATGCAAAGCGTGTTACTCTTATGAAAAAG
GATATTGAGTTGGCTCGGAGGCTTGGAGGAATAGGAAGGCCT???

Cicer_bijugum
ATGGCTAGAGTTAAGCACATTCCTCCTCCTCGTAAT------------------------
CGCGCT---------------------GTA------------------------------
----------------------------------------------------------
----------------------------------------------------------
---------------------AGTGAGGATCAGGAACATAAGAAAAGACGCAATAAGCCT
GGAACAGTGGCGCTTCGCGAGATTCGTCGTTTTCAGAAGACTTTCAATTTGCTTTTACCA
GCTGCTCCATTTATTAGATGCGTCAAACAGATTACGAACCAAACATCTTCACATGTCTCA
CGTTGGTCGCCGGAAGCAGTAATAGCACTTCAGGAGGCGGCTGAGGATTATCTGGTACAT
ATGTTTGAAAATGGAATGCTATGTGCACTTCATGCAAGGCGTATTACCCTTATGAAAAAA
GATATTGAGTTGACCCGTAGGCTTACAGGAATAGGAAGGCCGTGG

Cicer_judaicum
ATGGCTAGAGTTAAGCACATTCCTCCTCCTCGTAAT------------------------
CGCGCT---------------------GTA------------------------------
----------------------------------------------------------
----------------------------------------------------------
---------------------CGTGAGGATCAGGAACATAAGAAAAGACGCAATAAGCCT
GGAACAGTGGCGCTTCGTGAGATTCGTCGTTTTCAGAAGACTTTCAATTTGCTTTTACCA
GCTGCTCCATTTATAAGATGCGTCAAACAGATTACGAACCAAACATCTTCACATGTCTCA
CGTTGGTCGCCGGAAGCAGTAATAGCACTTCAGGAGGCGGCTGAGGATTATCTGGTACAT
ATGTTTGAAAATGGAATGCTATGTGCACTTCATGCAAAGCGTATTACCCTTATGAAAAAA
GATATTGAGTTGACCCGTAGGCTTACAGGAATAGGAAGGCCGTGG

Cicer_pinnatifidum
ATGGCTAGAGTTAAGCACATTCCTCCTCCTCGTAAT------------------------
CGCGCT---------------------GTA------------------------------
----------------------------------------------------------
----------------------------------------------------------
---------------------AGTGAGGATCAGGAACATAAGAAAAGACGCAATAAGCCT
GGAACAGTGGCGCTTCGCGAGATTCGTCGTTTTCAAAAGACTTTCAATTTGCTTTTACCA
GCTGCTCCATTTATAAGATGCGTCAAACAGATTACGAACCAAACATCTTCACATGTCTCA
CGTTGGTCGCCGGAAGCAGTAATAGCACTTCAGGAGGCGGCTGAGGATTATCTGGTACAT
ATGTTTGAAAATGGAATGCTATGTGCACTTCATGCAAGGCGTATTACCCTTATGAAAAAG
GATATTGAGTTGACCCGTAGGCTTACAGGAATAGGAAGGCCTTGG

Cicer_reticulatum
ATGGCTAGAGTTAAGCACATTCCTCCTCCTCGTAAT------------------------
CGCGCT---------------------GTA------------------------------
----------------------------------------------------------
----------------------------------------------------------
---------------------AGTGAGGATCAGAAACATAAGAAAAGACGCAATAAGCCT
GGAACAGTAGCGCTTCGCGAGATTCGTCGTTTTCAAAAGACTTTCAATTTGGTTATACCA
GCTGCTCCATTTATAAGATGCGTCAAACAGATTACGAACCAAACATCTTCACATGTCTCA
CGTTGGTCGCCGGAAGCAGTAATAGCACTTCAGGAGGCGGCTGAGGATTATCTGGTTCAT
ATGTTTGAAAATGGAATGCTATGTGCCCTTCATGCAAGGCGTATTACCCTTATGAAAAAG
GATATTGAGTTGACCCGTAGGCTTACAGGAATAGGAAGGCCTTGG

Cicer_yamashitae
ATGGCTAGAGTTAAGCACATTCCTCCTCCTCGTAAC------------------------
CGCGCT---------------------GTA------------------------------
----------------------------------------------------------
----------------------------------------------------------
---------------------AGTGAGGATCAGGAGCATAAGAAAAGACGCAATAAGCCT
GGAACGGTGGCGCTTCGCGAGATTCGTCGTTTTCAAAAGACTTTCAATTTGCTTATACCA
GCTGCTCCATTTATAAGATGCGTCAAACAGATTACAAACCAAACATCTTCACATGTCTCA
CGTTGGTCGCCGGAAGCAGTAATAGCACTTCAGGAGGCGGCTGAGGATTATCTGGTTCAT
ATGTTTGAAAATGGAATGCTCTGTGCACTTCATGCAAGGCGTATTACCCTTATGAAAAAG
GATATAGAGTTGACCCGTAGGCTTACAGGAATAGGAAGGCCTTGG

Glycine_max
ATGGCGAGAGTGAAGCACACGCCAGCTTCTCGCAAATCCGCT---AAAAAGCAAGCGCCA
CGC???------------------------------TCCACTTCCACGCAGCCG------
???------CCA------???------------TCCCAATCGCCTGCAACTAGAGAGAGG
---AGGAGAGCTCAACAAGTGGAGCCGCAG???------------------------???
---------------------???GAGGCTCAGGGAAGGAAGAAGAGGCGCAATAGGTCG
GGAACTGTGGCGCTTCGTGAGATTCGGCATTTTCAGAGGAGTTGTGAGCTTCTCATCCCG
GCTGCTCCCTTTATCAGATGTGTCAAACAGATTACAAATCAATTCTCTACGGAGGTGTCT
CGTTGGACACCTGAAGCTGTGGTAGCACTTCAGGAGGCAGCTGAGGAATATCTGGTTCAC
TTATTTGAAGATGGAATGCTCTGTGCAATTCATGCAAGGCGTATTACTCTTATGAAAAAG
GACATTGAGTTGGCCCGGAGGCTTGGAGGAATAGGAAGGCCTTGG

Lens_culinaris
ATGGCGAGAGTTAAACAAACACCACGTCCC------------------------------
---------------------------GTA------------------------------
----------------------------------------------------------
----------------------------------------------------------
---------------------CTTCAGAATCAGGAAAGGAAGAAAAGACGGAATAAACCT
GGAACCGTAGCGCTTCGTGAGATCCGGAAACTTCAAAAAACTTTCCAATTACTTATACCA
TATGCTCCCTTTGTAAGATGTGTCAGGGAAATTACAAACCAAGTATCTTCTTCGGTTACG
CGCTGGACGCCGGAAGCATTGATTTCACTTCAAGAGGCAGCTGAGGATTGTCTAGTTCGA
ATGTTTGAAGCTGGATGTCTCTGTGCACTTCATGCAAAGCGTGTTACCCTTATGAAAAAG
GATATTGAATTGACGCGAAGGCTTACCGGGGTAGCAAGACCTTGG

Lotus_japonicus
ATGGCGAGAATCAAGCACGTTCCTGCTGCTCGTAGAACCGCT---CGAAAGAAAGCACCA
??????---------------------???GCACCATCCACATCT???------ACA---
???------CAA------???------------ACCCAATCACCTGGTCGTCGAGAGAGG
---AGTAGAGAACAACAACGGGAACCGCAG------------------------------
------------------TCA???GGGACTCAGGGGAAGAAGAAGAGGCGCAATAGGCCG
GGAACGGTGGCGCTTCGTGAGATCAGGCGTTATCAGAAGAGTGTAGACTTGCTTATCCCT
GCTGCCCCCTTTCTGAGATTGGTTAGAGAGACTACACGTCAGTTATCTTTTGAGGTTTCT
CGCTGGACAGCTGAAGCTGTGGTGGCTCTGCAGGAGGCAGCGGAGGACTATCTTGTTACT
ATGTTTGAAGATGGAATGCTCTGCGCAATTCATGCAAAGCGCGTTACCCTTATGAAAAAG
GATATTGAGTTGGCCCGTCGGCTTCGAGGAATAGGAAGGCCTTGG

Medicago_truncatula
ATGGCAAGAGTCAAGCACATTCCACGTCCTGGTAAA------------------------
CGCACTCGTCGCAGTAGTAGTAGTAATGCA------------------------------
----------------------------------------------------------
----------------------------------------------------------
---------------------AATGAGTCTGAGGAAAAGAAGAAAAGAAGGAATAGACCA
GGAACAGTTGCGCTTCGCGAGATTCGTAAATTTCAAAAGGCTGTTAACTTGCTTATACCT
TGTGCTCCGTTTGTTAGATGCGTCAAACAGATTACAAACCAACTATCTATGGAGGTATCA
CGTTGGACGGCTGAAGCCTTATTAGCACTTCAGGAGGCAGCTGAGGAGCATCTGGTTCGT
ATGTTTGAAGGTGGGATGCTCTGTGCACTTCATGCAAAGCGTGTTACCCTTATGAAAAAG
GATCTTGAGTTGACCCGTAGGCTTACAGGAATAGGATGCGCTCGG

Phaseolus_vulgaris
ATGGCGAGAGTGAAGACCACGCCAGCTTCGCGCAAAACCGGT---AAAAAAAAAGCCGCA
CGC???------------------------------TCCACGTCCACG------------
???---------CGGTCA???---------------CAATCGCCT---------GGGAGG
---AGAAGGGCTCAACAAGAGGAGCCGCAG???---GCAGAAGAAGAAGAAGAA---???
???---------??????GCG???GAGACTCAGGGAAGGAAGAAAAGGCGCAATAAGGCA
GGAACAGTGGCGCTTCGCGAGATTCGTCAATTTCAGAGGAGTTCCAAACTTCTTATCCCG
GCTGCCCCTTTTATGAGATGTGTCAGACAAATTACACAGCAATTCTCTGCGGAGGTGAGT
CGCTGGACACCTGAAGCAGTGGTAGCACTCCAGGAGGCAGCTGAGGAATGTCTAGTTCAC
TTGTTTGAAGATGGAATGCTTTGTGCAATTCACGCAAGGCGTGTTACTCTTATGACAAAG
GATATTCAGTTGGCTCGGAGACTTGGAGGAATAGGAAGGCCTTGG

Pisum_sativumB
ATGGCGAGAGTTAAACAAACACCACGTCAC------------------------------
---------------------------GCC------------------------------
----------------------------------------------------------
----------------------------------------------------------
---------------------CGTGAGAATCAGGAAAGGAAGAAAAGACGTAATAAACCT
GGAACCGTAGCGCTTCGTGAGATCAAGAAATTGCAAAAAACTTTCCAATTACTTATACCA
TATGCTCCGTTTGTAAGATGCGTCAGGGAAATTACAAATCAAGTATCTTCATTGGTTACA
CGCTGGACGCCTGAAGCGTTGCTATCACTTCAAGAGGCAGCTGAGGATTGTCTAGTTCGA
ATGTTTGAAGCTGGATGGCTCTGTACACTTCATGCAAAGCGTGTTACCCTTATGAAAAAG
GATATTGAGTTAACGCGCAGGCTTACCGGGATAGGAAGACCTTGG

Vigna_unguiculata
ATGGCGAGAGTGAAGCACACGCCAGCTTCGCTCAAAGTTGGT---AAAAAAAAAGTCAGT
CGC???------------------------------TCCACATCCACG------------
???---------------???---------------CAATCGCCTGCGACAAGAAGTCGT
---AGAAGGGCTCAAGAAGAGGAGCCGCAGGAA------------------------???
???---------??????GCG???CAGACTCAGGGAAGGAAGAAAAAGCGCAGTAAGCCA
GGAACAGCGGCGCTTCGCGAGATTCGTCATTTTCAGAAGAGTTGCAAGCTTCTTATCCCG
GCTGCCCCCTTTATCAGATGTGTCAAACAAATTACACATCAATTCTCTACGGAGGTGTCT
CGCTGGACGCCTGAAGCTGTGGTAGCACTGCAGGAGGCAGCTGAAGAATGTCTAGTTCAC
TTGTTTGAAGATGGAATGCTTTGTGCAATTCACGCTAGGCGTATTACTCTTATGACAAAG
GATATTCAGTTGGCTCGGAGGCTTGGAGGAATAGGAAGGCCTTGG

;
end;

BEGIN TREES;
		TREE tree = (Arachis_appressipila,(Glycine_max,((Phaseolus_vulgaris,Vigna_unguiculata),(Cajanus_cajanifolius,(Cajanus_cajan,Cajanus_scarabaeoides)))),(Lotus_japonicus,(Astragalus_sinicus,((Medicago_truncatula,(Lens_culinaris,Pisum_sativumB)),(Cicer_yamashitae,(Cicer_reticulatum,(Cicer_pinnatifidum,(Cicer_bijugum,Cicer_judaicum))))))));
END
```

Ferns

```
#NEXUS
[ Title ]
begin taxa;
	dimensions ntax=8;
	taxlabels
		Azolla_filiculoides
		Ceratopteris_richardii
		Equisetum_arvense
		Equisetum_diffusum
		Lygodium_japonicum
		Ophioglossum_petiolatum
		Psilotum_nudum
		Pteridium_aquilinum
;
end;
begin characters;
	dimensions nchar=798;
	format missing=? gap=- matchchar=. datatype=nucleotide;
	matrix

Azolla_filiculoides
ATGGCGAGACTCAAGCAAAGACCACACAGGTCTAATCTGGCTGCATTGTCGTCCAATGTT
---------GAAACTACA------GGAAGTAGCCAGCCCCATTTGAATCGCAGTAATAGT
---------------------GCGCCACCAACTGCTCCC---------------------
------------------------------------------------------------
------ACAACA------------------------------------------------
------------------------------------------------------------
------------------------------------------------------------
---------------???TTCAAC------------------AATATTCCTGCTAATCGC
CAACGCAAACCTCATCGATTTAGGCCTGGTACAGTGGCTTTAAGAGAAATCAGGCGCTAC
CAAAAGACTGTTCACTTTCTTATCCATCCCTTACCATTTGCACGACTGGTCAGGGAACTA
ACTGCCCAATTTTCAGACACAGTGAGCCGATGGACAGCTGAGGCATTGGTTGCTTTGCAA
GAGGCTGCTGAGGATCATTTGGTGCATTTGTTTGAGGACACTAATTTATGTGCTATCCAT
GCAAAACCTGTCACAATAATGCCTAAAGATATGCAGCTTGCAAGACGTTTGCGTGGCAAC
ACCATTGACAGGCCTTGG

Ceratopteris_richardii
ATGGCGAAGAAAAAGAAGACCCCGAAAAAGGCTTCCCAGGTCGCTTCTTCCTCTACCGTC
CCTCAGCCTGAGATCACAGATGCA???AACAGCAGAAGACGACTCCATCGAAGCAGTAGT
---------------------GCACCTCCAACTGCTCCA---------------------
------------------------------------------------------------
------GTTACT------------------------------------------------
------------------------------------------------------------
------------------------------------------------------------
---------------???ACAAATGAACGAGAGCCTACAGACAATACACCTTCCAGGCGT
CCACGCAAACCCCACAGATTTCGTCCAGGAACAGTAGCTCTTCGGGAAATTCGACATTAT
CAGAGAACTGTTAATTTTCTCATACATCCATTGCCATTTGCCAGATTGGTGAGAGAAATT
GGGGTCCAATTTTCAGATACTGTCTCTCGGTGGACTGCAGAATCACTTCTAGCATTACAG
GAGGCTGCTGAGGATCACATTGTACACCTATTTGAGGATACAAACCTATGTGCCATACAT
GCTAAGCGTGTCACCATAATGCCAAAGGATATGCAATTAGCAAGGAGACTTAGAGCAAGT
ACCCTTGACAGGCCTTGG

Equisetum_arvense
ATGGCGAAGAAGAAA---------------------------------------------
------------------------------------------------------------
------------------------------------------------------------
------------------------------------------------------------
------------AGTGCTCCCGCGCGCTCCCAAAAGTCCCCTGCTTCCACAGTCGTCGAC
GGAAATGGGCAGCCTTCCAGTAAGAATGCAAAGGATCAAGGTGGGCCTTCAAGCACTCCC
GATACTGCAAATGTTGGTGGCAGAGGAGTTAAAGGGGCAAAGGGAAAAGGAAGAACACAG
CAGGGTGCCGAAAACCCT------------------------------------------
---CGGAAGTCACACCGTTACAAAGCCGGAACGGTTGCACTGCGGGAAATCAGATTCTAC
CAGAAGAATGTAGATCTACTCATTCGGCCTCTCCCCTTTGCACGCTTGGTGCGAGAAATT
GCTGATCACCTCTCTCGGACTGTTACTCGCTGGACAGCTGAAGCCTTGGTGGTTATGCAA
GAGGCATGCGAGGATTTTATTGTCCATCTTTTTGAGGACACCAATCTTTGTGCAATTCAT
GCCAAACGCGTAACTATAATGACTAAGGACATGCAACTTGCTAGGCGGCTCCGTAATACA
ATGATTGATAGGCCGTGG

Equisetum_diffusum
ATGGCGAAGAAGAAA---------------------------------------------
------------------------------------------------------------
------------------------------------------------------------
------------------------------------------------------------
------------AGTGCGCCCGCGCGCTCCCAAAAGTCCCCTGCTTCCACAGTCGTCGAC
GGAAATGGGCAGCCTTCCAGTAAGAATGCAAAGGATCAAGGTGGGCCTTCAAGCACTCCC
GATACTGCAAATGTTGGTGGCAGAGGAGTTAAAGGGGCAAAGGGAAAAGGAAGAACACAG
CAGGGTGCCCAAAACCCT------------------------------------------
---CGGAAGTCACACCGTTACAAAGCCGGAACGGTTGCACTGCGGGAAATCAGATTCTAC
CAGAAGAATGTAGATCTACTCATTCGGCCTCTCCCCTTTGCACGCTTGGTGCGAGAAATT
GCTGATCACCTCTCTCGGACTGTTACTCGCTGGACAGCTGAAGCCTTGGTGGTTATGCAA
GAGGCATGCGAGGATTTTATTGTCCATCTTTTTGAGGACACCAATCTTTGTGCAATTCAT
GCCAAGCGCGTAACTATAATGACTAAGGACATGCAACTTGCTAGGCGGCTCCGTAATACA
ATGATTGATAGGCCGTGG

Lygodium_japonicum
ATGGCCAGAAGGAAGCCTAATCCAAAGAAGGCCACGCCAGTTGCTTCTTCTTCGAACGCC
ACACCGCAGGAGGCGACA------GCAAGCAGCAGACCGCGCTTAGTTAGAAGCAGTAGT
---------------------GCACCACCGACAACCCCA---------------------
------------------------------------------------------------
------GCAACA------------------------------------------------
------------------------------------------------------------
------------------------------------------------------------
---------------???TCAAT-------------------????????????---CGA
CAGCGCAAGCCTCATCGGTTTAGACCCGGAACTGTTGCTTTGCGGGAAATCAGGCATTTT
CAGAAGACAGTTCACTTTCTTATTCATCCATTACCATTTGCACGGCTGGTTAGAGAAATT
GCAGCTCAATGTTCGGATACAGTAACTCGATGGACTGCCGAAGCATTGGTTGCTTTGCAG
GAGGCTGCAGAAGACCACCTTGTACACTTGTTTGAGGACACTAATCTGTGTGCTATTCAT
GCAAAACGAGTCACAATAATGACAAAAGACATGCAACTTGCACGGCGTTTACGAGGCAGC
ACTCTAGATAGGCCGTGG

Ophioglossum_petiolatum
ATGGCTCGAAGGAAGCCGAGGCCGCAAAAAGCA---------------------------
------------------------------------------------------------
---------------------??????????????????ACTTCAACAGCCGTCGCACCA
TCGCCACAGCCAGTAGGCGGTCGTAAAGGAAGTCGTAAAACAGCCGGCAGACGCCGTGCT
ACCGCG??????------------------------------------------------
------------------------------------------------------------
------------------------------------------------------------
---------------CCC------------------------------------------
---AAAAAGCCCCACCGTTTCAAGCCCGGTACCGTTGCTTTGAGAGAGATCAAATATTAC
CAGAAGAAATTTCACCTCCTCATTCGTCGTCTTCCCTTTGCAAGATTAGTTAAAGAAATT
ACGGCTCATTTTTCACGCAATGTTACTCGCTGGACAGCGGAGGCGCTGACTGCTTTGCAA
GAGGCAGCTGAAGACCAAATTGTTCATCTTTTTGAGGACACCAATTTATGTGCCATCCAC
GCAAAGCGCGTTACAATAATGCCAAAGGATATGCAACTTGCAAGGCGTCTACGTGGGAAC
ACAGCTGACAGGCCATTT

Psilotum_nudum
????????????????????????????????????????????????????????????
????????????????????????????????????????????????????????????
AGTCAAACCGCGAGACGCACC??????????????????---------------------
------------------------------------------------------------
------??????------------------------------------------------
------------------------------------------------------------
------------------------------------------------------------
---------------TCT------------------------------------------
---AGAAAGCGTCATCGGTTTCGGCCTGGAACAGTGGCATTACGAGAAATTAGATTTTAT
CAGAAGCGCTTTCATCTTTTAATTCCACCACTGCCTTTTGCCCGACTGGTCAAGGAAATG
ACATTATATTTTTCTCGCGATGTGTCCCGCTGGACGGCCGAGGCATTAGTGGCGTTGCAG
GAGGCTGCTGAGGATTTCATCGTTCATCTGTTTGAGGACACCAATTTATGTGCTATTCAT
GCAAGACGTGTCACAATAATGACCAAAGACATGCCGCTTGCCAGGCGCTTGCGTGGAGCC
ATTGCTGATAGGCCCTGG

Pteridium_aquilinum
????????????????????????????????????????????????????????????
????????????????????????????????????????????????????????????
????????????????????????????????????????????????????????????
????????????????????????????????????????????????????????????
????????????????????????????????????????????????????????????
????????????????????????????????????????????????????????????
????????????????????????????????????????????????????????????
??????????????????????????????????????????????????????AACCGC
CAACGCAAGCCCCATCGGTTTCGCCCTGGAACTGTAGCTCTCCGAGAGATTCGACATTAC
CAAAAGACTGTCAATTTTCTCATTCATCCCCTGCCCTTTGCCAGATTGGTGAGAGAACTG
GCCGGTCAGTTTTCAGACTCAGTATCCCGGTGGACTGCAGAGGCACTGGTTGCGCTACAG
GAGGCTACTGAGGACCACCTTGTGCACCTTTTTGAGGACACTAATTTATGCGCAATTCAT
GCCAAACGTGTCACAATAATGACGAAAGACATGCAACTAGCAAGGCGTTTGCGAGGAACC
ACTCTGGACAGGCCTTGG

;
end;

BEGIN TREES;
			TREE tree = (Azolla_filiculoides,(Lygodium_japonicum,(Ceratopteris_richardii,Pteridium_aquilinum)),(Ophioglossum_petiolatum,(Psilotum_nudum,(Equisetum_arvense,Equisetum_diffusum))));
END;
```

Lycopodiophyta

```
#NEXUS
[ Title ]
begin taxa;
      dimensions ntax=5;
      taxlabels
            Isoetes_sinensis
            Lycopodiella_caroliniana
            Selaginella_kraussiana
            Selaginella_moellendorffii
            Selaginella_stauntoniana
;
end;
begin characters;
      dimensions nchar=627;
      format missing=? gap=- matchchar=. datatype=nucleotide;
      matrix

Isoetes_sinensis
ATG---------------------?????????AAAAGTACCCCAAAAACGGGCAAAGCC
TCTACTCCTGCAGCTTCTTCTTCAGCTACAGGAACCAGTTCTAGAAGAGTA---------
------------------------GATGGTGCGAGCACTAGTAGGGCAGGAAATGCACGT
AGTTCGGATAGGGTTGTAGCT------------------------------------
-----------------------------------??????CAAAAAAGG------???
---???---------CGGTTTAAACCTGGAACTGTGGCGCTTAGGGAAATAAGGCATTTT
CAAAAGAGCTACGGATTTCTTCTCAGGCCTTTGCCATTTGCCAGAGTGGTGAGAGAGATA
ACTGCCCAG------TATTCTAAAGAAGTGTCAAGATGGACTGCTGAAGCTTTAATAGCT
ATTCAAGAGGCTGCTGAAGACTACCTTGTACATCTTTTTGAAGACACAAATTTGTGTGCT
ATTCATGCCAAAAGAGTCACTATCATGCCAAAAGATTTGCAACTTGCACGTCGTTTACGA
GGGGGTACGGAAAGAAAGATG???---

Lycopodiella_caroliniana
????????????????????????????????????????????????????????????
????????????????????????????????????????????????????????????
????????????????????????????????????????????????????????????
??????????????????????????????GCTACCCCAGTTTCTGAGTCGGCAAGC
AGTAGAAGAGCTGTTGGAGAACGTCAAAGACAAGCG??????---------CAGCGA???
CCT???---------CGGTTCAAGCCTGGTACTGTGGCTCTCAGAGAGATTCGAAAGTAT
CAAAAAAGTTTCAATCTCTTGATAAGGCCCTTGCCATTTGCACGACTGGTTCGTGAAATC
ACATCTCAA------TTCTCTACTGATGTTACAAGATGGACAGCAGAAGCTCTGATTGCC
ATTCAAGAGGCTGCTGAGGATTACCTTGTTCATCTTTTTGAAGATACCAACTTATGTGCC
ATTCATGCCCGTCGGGTGACAATTATGCCAAAGGATCTGCATCTTGCGCGACGTCTTCGA
GGAGCTTCTGAG---AAAATGTTCATC

Selaginella_kraussiana
????????????????????????????????????????????????????????????
????????????????????????????????????????????????????????????
?????????????????GGTGGCGATGGCGCCGGGACGAGCAGAGCAGGT---------
---------------------GCCCAGCAGAAC---------------------------
------------------------------------------------------AGA
CCA------???CGGCGTTTCAAGGCCGGGACTGTAGCGCTTCGCGAAATTCGGAAGTTT
CAGAAGAGCTTTGAGCTCCTCCTCAGGCCTCTACCCTTCGCCAGAGTGGTTCGGGAGCTT
GCATCCTTG------TGTTCAACTGAGGTTACTAGATGGACTGCTGAATCCCTCCTTGCT
CTCCAAGAGGCTGCGGAGGATTACTTGGTGCATTTGTTTGAAGACACCAACTTGTGTGCG
ATACATGGAAAACGGGTTACGATA------------------------------------
---------------------------

Selaginella_moellendorffii
ATG------------------------------------------------------
---------------------------------------------------------
------GAGTGCGCAGCGGGCGGCGATGGCGCTGGGACGAGCAGAGCCGGC---------
---------------------GCCCAGCAGAAC---------------------------
------------------------------------------------------AAG
CCACACGGCAAGCGCAGGTTTAAGCCCGGGACTGTGGCGTTGCGGGAGATCCGACGCTTT
CAAAAGAGTTATGAGCTACTTCTCAGGCCATTGCCATTCGCAAGAGTGGTGAGAGAGATC
ACG------AACGTTTTCTCGTCGGAGGTATCCAGATGGACTGCCGAAGGATTGATAGCT
CTCCAAGAGGCTGCCGAGGACTACCTTGTTCATCTCTTCGAAGACACCAACTTATGTGCC
ATTCACGGCAAGAGAGTCACCATAATGCCCAAGGACTTACATTTGGCCCGTCGACTAAGA
GGAGCCTCCGAACGG------TTCGTT

Selaginella_stauntoniana
ATGGCCAAGAGGAGGAAATCGACG?????????---------------------------
-------------------------------------------------CGAGGGCGA
CCACCA---------GCGGGCGGCGATGGCGCTGGGACGAGCAGAGCCGGC---------
---------------------GCCCAGCAGAAC---------------------------
------------------------------------------------------AAG
CCGCACAGCAAGCGCAGGTTTAAGCCCGGGACTGTGGCGTTGCGGGAGATCCGACGCTTT
CAAAAGAGCTATGAGCTCCTTCTCAGGCCATTGCCATTCGCAAGAGTGGTGAGAGAGATC
ACG------AACGTTTTCTCGTCGGAGGTATCCAGATGGACTGCCGAAGGATTGATAGCT
CTCCAAGAGGCTGCCGAGGACTACCTTGTTCATCTCTTCGAAGACACCAACTTATGTGCC
ATTCACGGCAAGAGAGTCACCATAATGCCCAAGGACTTGCATTTGGCCCGTCGACTAAGA
GGAGCCTCCGAGCGG------TTCGTT

;
end;

BEGIN TREES;
                                    TREE tree = (Isoetes_sinensis,Lycopodiella_caroliniana,(Selaginella_kraussiana,(Selaginella_moellendorffii,Selaginella_stauntoniana)));
END
```

Penicillium

```
#NEXUS
[ Title ]
begin taxa;
    dimensions ntax=11;
    taxlabels
        Penicillium_bilaiae
        Penicillium_brevicompactum
        Penicillium_canescens
        Penicillium_expansum
        Penicillium_fellutanum
        Penicillium_glabrum
        Penicillium_chrysogenum
        Penicillium_janthinellum
        Penicillium_lanosocoeruleum
        Penicillium_marneffei
        Penicillium_raistrickii
;
end;
begin characters;
    dimensions nchar=861;
    format missing=? gap=- matchchar=. datatype=nucleotide;
    matrix

Penicillium_bilaiae
ATGCCCCCC---???GCCCGC------------AAA------------------------
---------ACA------------GGC------------CCA------------GCAAGC
---------CCA------------GCTCAA------------------------------
------------------------------------------------------------
------------------------------------------------------------
---------------------------------------GCG---------------CGC
AAA---ACC---------------------------------------------------
------??????????????????---------------------------------AAA
GCAGCCTCAAAG------?????????AATGTCCAACCCGGTGATCCCACACCCCAAGGT
CGCCGCCGGCGCTACAAGCCCGGCACCGTCGCCTTGAAAGAAATCCGCCGTTACCAACGC
TCCTACGACCTATTAATCGCCAAACTGCCCTTTGCGCGCTTAGTCCGCGAAGTCGCACTG
GATCTCCTCCCCGCAGAAGTTGGCGCCGAGCTACGATGGCAATCACAAGCTATCCAGGCG
CTGCAAGAAGCCGCGGAGGCCTTCATGGTGCATTTATTCGAAGATACAAATCTGTGCGCG
ATACATGCGAAGCGCGTGACGATTATGCAGAAGGATATTCAGCTTGCGCGTCGGATTCGA
GGAGCGTGGGGTGGTCTAGGC

Penicillium_brevicompactum
ATGCCACCGAAGATGGCG---------------------AAGATCTTGAAGAAGAAAACC
ACCGAT---------------------------------------------------AGC
------------------------CCACAAAAG---------------------------
---ACC---AAAGGTCGCAGA????????????---------------------------
------------------------------------------------------------
---------------GATGGCGATGGCTCACCA---------AATGCCGTG------CGC
AAA---TCC------------------AAG---CCC---------------???AAACAA
---AAAGCCAACAAG???------------------------------------------
------------------------GCAGATGTCCAACCGGGTGATCCAACCCCCACAGGC
CGCCGCCGCCGCTACAAGCCTGGCACCGTGGCACTCAAAGAGATCCGCAAATACCAACGC
TCCTACGACCTCCTCATAGCTAAGCTGCCGTTCGCGCGACTCGTTCGCGAAGTCGCTCTC
GATCTTCTGCCCGCCGACACGGGCGCCGAACTGCGTTGGCAGTCACATGCGATCCTGGCA
CTGCAAGAAGCCGCCGAGGCATTCCTGGTTCATTTGTTCGAGGACACAAATCTGTGTGCG
ATCCACGCCAAGCGCGTCACAATTATGCAAAAGGATATCCAGCTTGCGCGGAGAATTCGC
GGGGTCTGGGCTGGTCTGGGT

Penicillium_canescens
ATGCCACCCAAAATGGCACGA------------AAG------------------------
---------TCA------------GCA------------CCA------------GGCAGC
------------------------GCACAA???---------------------------
------------???CGCAAAACAGTCGCGACA---------------------------
------------------------------------------GCTGCCGCA------
---------------??????------TCACCAATACAAGCG---------------CGC
AAAATC???------------------AAA---------------------???AAGCAA
???------GCAAAGACAGCA---------------------------------------
------------------------GCCGATGTCCAACCGGGCGACCCAACCCCTACAGGG
CGCCGCCGCCGCTACAAACCAGGCACAAAAGCCCTAAAAGAAATCCGCCAATACCAACGC
GGCTACGAGCTGCTCATCGCGAAACTACCCTTTGCCCGCTTAGTCCGTGAAGTGGCGCTC
GATCTTCTCCCCGCCGACGTCGGTGCCGAATTGCGCTGGCAGTCGCATGCTATTCAAGCA
CTGCAGGAGGCTGCCGAGGCTTTCCTTGTTCATCTATTTGAGGATACGAATCTTTGCGCA
TTGCATGCTAAGCGGGTTACTATTATGCAGAAAGATATTCAGCTTGCGAGGAGAATTCGG
GGTGTGTGGGGTGGTTTAGGG

Penicillium_expansum
ATGCCACCCAAGACAGCTCGC------------AAG------------------------
---------ACA------------GGG------------CCG------------GGCAGC
------------------------GCGCAAAAA---------------------------
---GGC---AAAGTGCGCAAGACAACCGCCGCG---------------------------
------------------------------------------CCCGCCGCC------
---------------???GGAGATGGAACACCAACAAAAGCA---------------CGC
AAG---ACG------------------AAGGGA------------------GGAAAGCAA
TCAAAAGTC---------AAC---------------------------------------
------------------------GCCAACGTCCAACCCGGAGACCCAACCCCCACAGGC
CGGCGCCGGCGCTACAAGCCCGGCACCGTCGCATTGAAAGAAATCCGCCGCTACCAACGC
TCCTACGACCTCCTAATCGCCAAACTCCCCTTTGCACGCTTAGTCCGCGAAGTCGCACTC
GATCTCCTCCCCGCCGACGTCGGCGCAGAGTTGCGCTGGCAGTCGCATGCTATCCAAGCT
CTGCAGGAGGCCGCCGAGGCTTTCCTTGTTCATCTTTTCGAGGATACAAATCTCTGCGCG
CTGCACGCGAAGCGCGTTACTATTATGCAGAAGGATATCCAGCTTGCGCGGAGAATTCGC
GGTGTCTGGGCTGGGCTTGGT

Penicillium_fellutanum
ATGGCCACCAAGACAGCA???------------???------------------------
------GCG???------------GCG------------CCG------------GCCAGC
---------CCG------------GCGCAAAAT---------------------------
---AGCCCA------------------------GCGCAGAAGCGTAAGCGCGGACCGACT
GTATCATCGCCTTCCAAACAGAAG------------------------------------
------------------------------------------------------------
???---???------------------???------GGA---------------------
---------???AAGACA???AAA---------------------------------AAG
CCAGCG------------AGAGCA???AATGTCCAGCCCGGCGACCCAACGCCCTCTGGT
AAACGCCGCCGCTACAAGCCCGGCACTGTTGCCCTCAAGGAAATCCGACGCTACCAACGC
TCTTACGATCTCTTAATCGCGAAACTCCCCTTTGCCCGACTCGTCCGCGAGGTCGCACTC
GACCTTCTCCCCGCTGAAGTCGGCTCCGAGCTGCGCTGGCAGTCGCACGCTATCCAGGCA
TTGCAAGAAGCCGCCGAGGCATTTCTCGTGCATCTCTTCGAAGATACCAACCTCTGTGCG
CTACATGCCAAGCGCGTCACAATCATGCAGAAGGATATCCAGCTTGCGCGGCGGATAAGA
GGCGTGTGGGGAGGTCTTGGA

Penicillium_glabrum
ATGCCCACCAAATCA???------------------------------------------
---------------------------AAACGATTGATTCCT------------GGGAGC
---------CCT------------GCA---???---------------------------
---??????---------------------------------------------------
------------------------GGAAGACCTCGCAAGAGCGCA?????????GAAACA
ACCGAA------------------GGATCACCGAGCGCAGCG---------------CGC
AAG---TCA------------------ACAGCT------------------GGCAAA---
---AAAGTGACAAAA??????---------------------------------------
------------GCAAAGCGAACA???AATGTCCAACCCGGTGATCCCACACCCACAGGC
CGTCGTCGCCGATACAAGCCCGGCACCGTCGCATTGAAGGAGATTCGCCGCTACCAACGC
TCTTACGACCTCTTGATTGCTAAGCTTCCATTCGCCCGTCTCGTCCGTGAAGTCGCACTC
GACCTCCTCCCCGCAGAGGTGGGCGGCACGTTGCGCTGGCAATCC???GCAATCCAGGCA
TTGCAGGAGGCTGCAGAAGCTTTCCTGGTGCATTTATTCGAGGATACCAACCTTTGTGCG
CTGCATGCGAAGCGAGTTACCATTATGCAGAAGGATATTCAGCTTGCGCGCAGAATTCGG
GGAGTTTGGGCAGGTCTCGGT

Penicillium_chrysogenum
ATGCCACCCAAGCTAGCCAGC------------AAG------------------------
---------ACA------------GCA------------CCA------------GGCAGC
------------------------GCACAAAAA---------------------------
---AAC---AAAGTGCGCAAGACAGTCGCTGCG---------------------------
------------------------------------------CCGGCCGCC------
------------------???GATGGAACACCAACCAAAGCA---------------CAG
AAA---ACG------------------AAGGGA------------------GCAAAGCAA
TCAAAAGTCGCAAAGACCACC---------------------------------------
------------------------GCCAACGTCCAACCCGGAGACCCAACCCCCACAGGC
CGCCGACGCCGCTATAAGCCCGGCACCGTCGCTTTGAAAGAAATCCGCCGCTACCAACGC
TCATACGACCTACTCATCGCTAAACTTCCCTTTGCGCGCTTAGTCCGCGAAGTCGCGCTC
GATCTCCTCCCCGCCGACGTCGGCGCCGAGTTGCGCTGGCAGTCGCATGCCATCCAAGCT
CTGCAGGAGGCCGCTGAAGCTTTCCTCGTCCACCTCTTCGAGGATACGAATCTATGCGCG
CTACATGCGAAGCGCGTAACTATTATGCAGAAGGATATCCAGCTTGCGCGGAGAATTCGC
GGTGTCTGGGCTGGGCTTGGT

Penicillium_janthinellum
ATGCCCCCA---???GCAAAA------------AAG------------------------
---------ACC------------GCC------------GCC------------GCCGGC
------------------------------------------------------------
------------------------------------------------------------
------------------------------------------------------------
---------------------------------------GCG---------------CGC
AAG---ACA------------------ATA???------AAGAAAGCCGTC??????---
---?????????------------------------------------------------
---------------------------AATGTACAACCCGGCGACCCAGCCCCGACAGGC
CGCAAACGCCGCTACAAGCCCGGCACCGTCGCCCTAAAAGAAATCCGCCGCTACCAACGC
TCCTACGACCTTCTCATCGCCAAGCTGCCCTTCGCCCGACTGGTCCGCGAAGTCGCGCTG
GACCTGCTCCCCGCCGAAGTTGGCGCCGAGCTCCGGTGGCAGTCGCATGCGATTCAGGCG
CTTCAGGAAGCCGCTGAGGCGTTTATGGTGCATTTATTCGAGGACACGAATTTGTGTGCG
ATTCATGCGAAGCGGGTTACGATTATGCAGAAGGATATTCAGCTTGCGAGGAGGATTCGC
GGGGTTTGGGGGGGATTGGGT

Penicillium_lanosocoeruleum
ATGCCACCCAAGTTAGCCAGC------------AAG------------------------
---------GCA------------GTA------------CCA------------GGCGGC
------------------------GCGCAAAAA---------------------------
---AGC---AAAGCGCGCAAGACAGTCGCTGCG---------------------------
------------------------------------------CCGGCTGCC------
------------------???GATGGAACACCAACCAAAGCA---------------CAG
AAT---ATG------------------AGGGGA------------------GGAAAGCAA
TCAAAAGTCGGAAAAAATCACC--------------------------------------
------------------------GCGACCGTCCAACCCGGAGATCCAACCCCCACAGGC
CGCCGACGCCGATATAAGCCCGGCACCGTCGCTTTGAAGGAAATCCGCCGCTACCAACGC
TCATACGACTTACTCATCGCTAAACTTCCCTTTGCGCGCTTAGTCCGCGAAGTCGCGCTC
GATCTCCTCCCCGCAGATGTCGGCGCCGAGTTGCGCTGGCAGTCGCATGCCATCCAAGCT
CTGCAGGAGGCCGCTGAAGCTTTCCTCGTCCACCTCTTTGAGGATACGAACCTCTGCGCG
CTACATGCGAAGCGCGTAACTATTATGCAGAAGGATATCCAGCTTGCGCGGAGAATTCGC
GGTGTCTGGGCTGGGCTTGGT

Penicillium_marneffei
ATGCCACCAAAAACAGGCCGAGGGCGCTCTTCC???GCC---------------------
---------???ACCACCAGCAGC???------------???AACTCTCCCAGA??????
AGAGCTGGC???TCATCCCGTCAA?????????GAAACAACCACAACATCAAGTCCCATC
ACA???CCA---------------------------------------------------
------------------------------------------------------------
------------------------???TCACCAACTACCGCC---------------AGA
AAT---ACGAAGCAGCCAATGTCCCCA??????------------------??????---
---??????GCAAAAACA??????AGAGTAACCAAGCGACCAGCCGGCAAAGCCCCG---
------------------CGATCA???AACATACAACCGGGCGACCCAACCCCCATCGGC
AAACGCCGCCGCTACAAGCCCGGCACCGTCGCCCTCAAAGAAATCCGCAAATACCAACGC
TCCTTCGATTTGTTGATTAGCAAACTCCCCTTTGCCCGCTTAGTTCGCGAAGTCGCACTA
GACCTGCTACCAGCCGAAGTAGGCGCCGAACTACGATGGCAATCGCACGCAATTATGGCG
CTGCAAGAAGCTGCAGAGGCGTTTTTGGTGCATCTGTTTGAGGATACGAATCTGTGCGCC
ATTCATGCGAAGAGGGTTACGATTATGCAGAAGGATATTCAGCTTGCCAGGCGGATTAGG
GGGATGTGGGCTGGTTTGGGC

Penicillium_raistrickii
???------------GCCCGC------------AAA------------------------
---------ACC------------GCA------------CCT------------GGAAGC
------------------------GCTCAAAAA---------------------------
---GCC---AAAGCTCGCAAGACAGCCGCAACC---------------------------
------------------------------------------CCCGCAGCC------
------AACGACGGCGACGGCGACGGGTCCCCA------------------GTCATC???
AAA---ACA------------------AAA---------------------???AAACAA
---AAAGTCACCAAG???------------------------------------------
------------------------TCCAACGTCCAGCCGGGCGATCCAACCCCCACAGGC
CGCCGTCGCCGCTACAAACCCGGTACCGTGGCACTGAAAGAAATCCGCAAATATCAACGA
TCCTACGACCTGCTTATCGCCAAGCTCCCATTCGCGCGACTGGTGCGCGAAGTCGCACTC
GACCTCCTCCCCGCCGATACAGGGTCTGAGCTGCGCTGGCAATCGCATGCAATTCTGGCG
CTGCAGGAGGCGGCCGAGGCATTCCTGGTTCATTTGTTCGAGGATACGAATCTGTGCGCT
ATTCACGCTAAGCGGGTTACTATTATGCAAAAGGATATTCAGCTCGCGCGGAGAATACGC
GGTGTTTGGGCTGGATTGGGC

;
end;

BEGIN TREES;
        TREE tree = (Penicillium_bilaiae,Penicillium_janthinellum,((Penicillium_glabrum,((Penicillium_canescens,(Penicillium_brevicompactum,Penicillium_raistrickii)),(Penicillium_expansum,(Penicillium_chrysogenum,Penicillium_lanosocoeruleum)))),(Penicillium_fellutanum,Penicillium_marneffei)));
END;
```

Plasmodium

```
#NEXUS
[ Title ]
begin taxa;
    dimensions ntax=7;
    taxlabels
        Plasmodium_berghei
        Plasmodium_falciparum
        Plasmodium_fragile
        Plasmodium_chabaudi
        Plasmodium_reichenowi
        Plasmodium_vinckei
        Plasmodium_yoelii
;
end;
begin characters;
    dimensions nchar=657;
    format missing=? gap=- matchchar=. datatype=nucleotide;
    matrix

Plasmodium_berghei
ATGACAAGGACAAAAAAAAGTGTAACTACACATACCCCTATAAACACA------------
------------CATACGCATATGTTTAATATGCTTGCAAATAATCCAATAATA------
------------------------------------------AATAAACAGGCACAC
AACCAACTTGGATCTAAAGTAACAAATATGAAC---------------------------
AATAAAAATAATTCAGGAAAT--------------------------------------
---------------ATAACACAAAAAAGTATAAATAAA---ACTCGTATTCGAAGACCA
CATAGATATAGACCCGGTGTGTTAGCATTAAAAGAAATTCGAGCTTATCAATCAACAACT
CAATTATTAATACCCAAAATACCATTTGTGAGGGTAGTTAAAGAAATAACAAAATTATAT
GAACTACCAAATAGCCAATTTCGTTATACTCCAGAAGCTTTACTAGCTCTTCAAACTGCA
TCAGAAGCATATTTAGTTAGTTTATTTGAAGATGCATATTTATGTTCACTTCATGCAAAC
AGAGTAACACTTATGCCAAAGGATATACATTTGGCTAGAAGAATAAGGGGGCGTGAT

Plasmodium_falciparum
ATGGTGAGAACAAAAAAGAATATACCAAATCATAACCCCTTAAAT???TTTAATAGGGAC
AAGTCATTTAAA-----------------------------------------------
--------------------------ACAAACAAAACATTACCA??????ACAGTACAC
CATGGAATTAGTTCCAAGACTACTAATATAAACAGACCCAGTGTAAATAGAGGAGGTATA
----------------------------------------------------------
---------AATGAA??????CAAAAAAACTTGCACAGG---??????ATAAGGAAACCC
CATAGATACAGGCCAGGTGTATTAGCATTAAAAGAAATAAGAGCATATCAAGCATCGACT
CAATTATTAATACCTAAAATTCCATTTGTTCGAGTAGTAAAAGAAATTACAAGATTATTT
GAATTACCAGATGAGCAATTTCGTTATACACCTGAAGCATTATTAGCTTTACAAACAGCA
TCAGAAGCATATTTGGTTAGTTTATTTGAGGATGCTTATTTATGTTCATTACATGCAAAC
AGAGTAACACTTATGCCCAAAGATATTCATTTAGCTAGAAGAATACGCGGAAGAGAC

Plasmodium_fragile
ATGGTGCGAACGAAAAAGAGCGTGCCCATGCACAACCCCTTG???---------------
--------------------------------------------------AACCCA
GACGGGGTGGGCAGCAGCAACGAACCGACGAACAAGACAGTGCCT??????CCATCACAC
AAG??????-------------------------------------------------
---------------------GCCTCGTCCAAATCAAATAACAACGCAGGAACAGGCAAA
GGGACTCGA????????????CAAAAGGGTTTGAAGAAGACA???CGAATTAGGAGGCCC
CATAGGTACAGACCAGGTGTATTAGCATTAAAGGAAATAAGAGCATACCAAGCGACTACA
CAGTTACTCATTCCTAAAATACCTTTTGTTCGAGTCGTAAAGGAGATTACTCGGTTGTTT
GAATTGCCCAATGAACAACTGCGTTACACCCCCGAGGCCCTGTTGGCACTGCAAACCGCA
TCGGAGGCCTACCTGGTCAGTCTCTTCGAAGATGCCTACTTGTGTTCACTTCACGCAAAC
CGCGTCACCCTCATGCCTAAGGACATACACTTGGCTCGCCGTATCCGCGGCCGCGAC

Plasmodium_chabaudi
ATGGCGAGGACAAAAAAGAGTGTAACTAACCATACCCCTATCAACACA------------
------------CATACGCATATGTTTAATATGCTCGCAAATAATCCAATGGTA------
------------------------------------------AATAAACCAGCACAT
AACCAAATTGGATCCAAACCAACAAATGTAAAT---------------------------
AATAAAAGTAATTCAGGAAAT--------------------------------------
---------------ATGACACAAAAAGGTTTAAATAAA---AATCGTATTCGAAGACCA
CATAGATATAGACCTGGTGTATTAGCATTAAAAGAAATTCGAGCTTATCAAGCAACAACT
CAATTATTAATACCTAAAATACCATTTGTAAGAGTAGTTAAAGAAATAACAAAATTATAT
GAATTGCCAGATAGCCAATTTCGTTATACTCCAGAAGCTTTACTAGCTCTTCAAACTGCT
TCGGAAGCATATTTAGTTAGCTTATTTGAAGATGCCTATTTATGTTCGCTACATGCAAAC
AGAGTGACCCTCATGCCTAAGGACATACATTTAGCTAGACGAATAAGAGGCCGAGAT

Plasmodium_reichenowi
ATGGTGAGAACAAAAAAGAATATACCAAATCATAACCCCTTAAAT???TTTAATAAGGAC
AAGTCATTTAAA-----------------------------------------------
--------------------------ACAAACAAAACATTACCA??????GCAGTACAC
CATGGAATTAGTTCCAAGACTACTAATATAAACAGACCAAGTGTAAATAGAGGAGGTATA
----------------------------------------------------------
---------AATCAA??????CAAAAAAACTTGCACAGG---??????ATAAGGAAACCC
CATAGATACAGACCAGGTGTATTAGCATTAAAAGAAATAAGAGCATATCAAGCATCGACT
CAATTATTAATACCTAAAATTCCATTTGTTCGAGTAGTAAAAGAAATTACAAGATTATTT
GAATTACCAGATGAGCAATTTCGTTATACACCGGAAGCATTATTAGCTTTACAAACAGCA
TCAGAAGCTTATTTGGTTAGTTTATTTGAGGATGCTTATTTATGTTCATTACATGCAAAC
AGAGTAACACTTATGCCCAAAGATATTCATTTAGCTAGAAGAATACGCGGAAGAGAC

Plasmodium_vinckei
ATGGCGAGGACAAAAAAGAGTGTAACTAACCATACCCCTATAAACACA------------
------------CATACGCATATGTTTAATATGCTCGCAAATAATCCAATGGTA------
------------------------------------------AATAAATCAACACAT
AACCAAATTGGGTCTAAAGGAACAAATATAAAT---------------------------
AATAAAAGTAATTCAGGGAAT--------------------------------------
---------------ATGACACAAAAAGGTTTAAATAAA---AGTCGTATTAGAAGACCA
CATAGATATAGACCTGGTGTATTAGCATTAAAAGAAATTCGAGCTTATCAAGCAACAACT
CAATTATTAATACCTAAAATACCATTTGTAAGAGTAGTTAAAGAAATAACAAAATTATAT
GAATTACCAGATAGCCAATTTCGTTATACTCCTGAAGCTTTATTAGCTCTTCAAACGGCT
TCGGAAGCATATTTAGTTAGCTTATTTGAAGATGCATATTTATGTTCACTACATGCAAAC
AGAGTGACTCTCATGCCTAAGGACATACATTTAGCTAGACGAATAAGAGGGAGAGAT

Plasmodium_yoelii
ATGGTGAGAACAAAAAAAAGCGTAACTACACATACCCCTATAAATACA------------
------------CATACGCATATGTTTAATATGCTTGCAAATAATCCAATGATA------
------------------------------------------AATAAATCAGCAAAT
AACCAAATTGGGTCAAAATCAATA------------------------------------
???AAAAATAATTCAGGAAAT--------------------------------------
---------------ATAACACACAAAAATATAAATAAA---AATCGTATCCGAAGACCA
CATAGATATCGACCTGGTGTATTAGCATTAAAAGAAATTAGAGCTTATCAAGCAACAACT
CAATTATTAATACCCAAAATACCATTTGTAAGAGTAATTAAAGAAATAACAAAATTATAT
GAATTGCCAAATAATCAATTTCGTTATACTCCCGAAGCTTTATTAGCTCTTCAAACTGCA
TCAGAAGCATATTTAGTTAGCTTATTCGAAGATGCATATTTATGTTCTCTTCATGCAAAT
AGAGTAACACTTATGCCAAAAGATATACATTTAGCTAGACGGATAAGAGGCCGTGAT

;
end;

BEGIN TREES;
        TREE tree = (Plasmodium_berghei,Plasmodium_yoelii,((Plasmodium_chabaudi,Plasmodium_vinckei),(Plasmodium_fragile,(Plasmodium_falciparum,Plasmodium_reichenowi))));
END;
```

Poaceae

```
#NEXUS
[ Title ]
begin taxa;
	dimensions ntax=12;
	taxlabels
		Brachypodium_distachyon
		Cenchrus_americanus
		Hordeum_bulbosum
		Hordeum_marinum
		Hordeum_vulgare
		Oaustraliensis
		Obrachyantha
		Osativa
		Panicum_virgatum
		Saccharum_hybrid
		Sorghum_bicolor
		Zea_mays
;
end;
begin characters;
	dimensions nchar=606;
	format missing=? gap=- matchchar=. datatype=nucleotide;
	matrix

Brachypodium_distachyon
ATGGCCCGCACGAAGCGCCCGGCCATCAGGAAGTCG---------AAGCCGCAGCCCAAG
AAGCAACTCCAGTTCGAG???---------------------------------------
ACAGGCGGC---GCGAGCACC???GCGTCG???ACCCCG??????CGT---??????CGG
ACCCCGGCGCGAGCGGCGGGTCAAGCG------GCACCCGCACAACAG---???CCG---
---AAGAAGCCA---------CACAGATTCCGGGCAGGCACGGTGGCGCTGCGGGAGATC
AGGAAGTACCAGAAATCCCCTGAGCTGCTCATCCCATTCGCACCCTTCGTCCGTCTGATT
AAGGAGATCAGTAATTTCTACTCACCTGAG---------------------------ATC
TCGCGCTGG---------------ACTCCTCAAGCTCTCGTTGCTTTGCAAGAGGCTGCA
GAATACCACTTGGTAAACATATTTGAAAAGGCAAATTACTGTGCCATCCATGCGAAGCGT
GTTACCATGATGCAAAAGGACATACAGCTTGCGAGG????????????????????????
??????

Cenchrus_americanus
ATGGCTCGAACCAAGCACCAGGCCGTGAGG??????GGCCGC---------???CCCAAG
AAGAAGCTCCAGTTCGAGCGCTCCCCTCGCCAG??????---GCGCAG------------
ACAGGCGGC---GCGAGCACCTCG------GGAACCTCGGGGAGG------GGT???CCG
GCTCGGGGTGGAGCGGCTGCCGCTGGG------GCGGCAGGG???---------------
ATT???AGGCCG---------CATCGGTGGCGACCAGGGACGGTGGCTCTGCGGGAGATC
AGAAAATTCCAGAAATCTACCAATCCTCTTATCCCATTTGCTCCCTTTATTCGTCTGGTG
AGGGAGATCACTAACGACTATTCGAAGGAA---------------------------GTG
ACACGCTGG---------------ACTCCTGAAGCCCTCCTTGCGCTGCAAGAGGCAGCA
GAGTTCCACTTAATTGAGCTGTTTGAAGTGGCAAATTTGTTGGCCATCCATGGGAAGCGT
GTTACTATCATGCAAAGGGACATACAGCTTGCAAGGCGTATCGGAGGA---???CGTTGG
TCG---

Hordeum_bulbosum
ATGGCCCGCACCAAGCACCCCGCCGTGAGGAAGTCC---------AAGGCGCCGCCCAGG
AAGAAGGTCGGGTCCGCGCGCGCCCCG???------???---GCGCAGCGCCGGCACGAG
ACAGATGGC---GCGGGCACGTCC------GAGACTCCGAGG---------------???
GGGCCGGCCCCGGCGGCGGATCAAGGG------GCACCTGGGGAACCC---???AAG---
---AGGAAGCCA---------CACCGGTACAGGCCAGGCACGGTGGCACTGCGGGAGATC
AGGAAGTACCAGAAGTCGGTCGATTTTCTCATCCCGTTTGCACCGTTTGTCCGTCTGGTC
AAGGAGGTCACCGAATTCTACTGTCCTGCA---------------------------ATC
AGCCGCTGG---------------ACTCCCCAAGCGCTCTTGGCAGTTCAAGAGGCTGCA
GAGTATCACCTCGTCGACGTATTTGAAAGGGCACATCTCTGTGCCATCCATGCAAAGCGT
GTTACCGTCATGCAAAAGGACATACAACTCGCA???????????????????????????
??????

Hordeum_marinum
????????????????????????????????????????????????????????????
????????????????????????????????????????????????????????????
????????????????????????????????????????????????????????????
????????????????????????????????????????????????????????????
??????????????????????????????????????????GTGGCGCTGCGGGAGATC
AGGAAGTACCAGAAGTCCACCGGGCTGCTCATCCCCTTCGCGCCCTTCGTCCGGCTGGTT
AAGGAGATCACC???---------------GACTTAACGAAGGGAGAGCTGAAC------
---???TGG---------------ACACCTCAGGCGCTCGTCTCGTTGCAAGAGGCTGCA
GAGTATCACATAGTCGATCTATTCGAAAAGGCAAATCTATGTGCCATCCATGCTAAGCGT
GTTACCATGATGCAAAAGGACATACAGCTGGCAAGGCGTATCGGGGGGACAAGGCTTTGG
------

Hordeum_vulgare
ATGGCCCGCACCAAGCACCCCGCCGTGAGGAAGTCC---------AAGGCGCCGCCCAAG
AAGAAGATCGGGTCCGCTAGCTCCCCG???------???---GCGCAGCGCCGGCAGGAG
ACAGATGGC---GCCGGCACGTCC------GAGACTCCGAGGCGG------GCC???CGG
GGGCCGGCCCCAGCGGCGGCTGAAGGG------GCACCTGGGGAACCG---???AAG---
---AGGAAGCCA---------CACCGGTTCAGGCCAGGCACGGTGGCACTGCGGGAGATC
AGGAAGTACCAGAAGTCGGTCAATTTTCTCATCCCGTTTGCACCGTTTGTTCGTCTGGTC
AGGGAGATCACCGAATACTACTGTCCTCGA---------------------------GTC
AAACGCTGG---------------ACTCCCCAGGCGCTCCTCGCAGTTCAAGAGGCTACA
GAGTATCACCTCGTCGACATATTTGAAAGGGCACATCTCTGTGCCATCCATGCAAAGCGT
GTTACCGTCATGCAAAAGGACATGCAACTCGCG???????????????????????????
??????

Oaustraliensis
ATGGCTCGCACGAAGCACCCGGCGGTGAGGTCG???------TCAAGGACGGAGCCCAAG
AAGAAGCTCCGGTTCGACCGCTCCCCTCGG---CCTTCGAAGGTGCAGAGC---------
ACTGGTGGT---GCGGGTACCTCG------GCGACCACGGGGAGC---------GCGGGG
ACCGCGGCT???------------GGG------ACGCCTGGGCAGCAGACAAGGCAG---
---AGGAAGCCA---------CACCGATTCCGTCCAGGCACAGTAGCACTACGGGAGATA
AGGAAATTTCAGAAAACAACTGAACTTCTTATCCCTTTTGCGCCATTTTCCCGTCTGGTC
AGGGAGATCACTGACTTCTATTCGAAGGAT---------------------------GTG
TCACGCTGG---------------ACTCTTGAAGCTCTCCTTGCACTGCAAGAGGCAGCA
GAATACCATTTGGTGGACTTGTTTGAAGTGTCAAACCTTTGCGCCATCCATGCTAAGCGT
GTTACCATCATGCAGAAGGACATACAGCTTGCCAGGCGTATCGGTGGGCGGAGGCCATGG
---GGC

Obrachyantha
ATGGCTCGCACCAAGCACCCGGCGGTGAGGAAGTCG---------AAGCCGGAGCCCAAG
AAGAAGCTCCAGTTCGAGCGCTCCCCGCGGAGG??????---GCGCAGCGC---------
---GGTGGC---GCGGATACCTCG------???---ACGAGGAGC---------GCGCGG
ACTGCGGCCGGAACGTCGGCCGAAGGG------ACGCCTGGACAGCAGTCGAGGCAG---
---AGGAAGCCA---------CACCGATTCCGTCCAGGCACAGTAGCTCTGCGGGAGATC
AGGAGATTCCAGAAATCGACCGAACTGCTTATCCCATTTGCGCCATTTTCCCGTCTGGTG
AGGGAGATCACTGATTTCTATTCCAAGGAT---------------------------GTG
ACGCGCTGG---------------ACTCTTGGAGCTCTCCTTGCACTGCAAGAGGCAGCA
GAATACCACTTAGTGGATTTATTTGAAGTAGCAAATCTCTGCGCCATCCATGCTAAGCGT
GTTACCATCATGCAAAAGGACATACAACTTGCCAGGCGTATCGGTGGACGGAGGCCATGG
------

Osativa
ATGGCTCGCACGAAGCACCCGGCGGTGAGGAAGTCG---------AAGGCGGAGCCCAAG
AAGAAGCTCCAGTTCGAACGCTCCCCTCGG---CCGTCGAAGGCGCAGCGC---------
GCTGGT???GGCACGGGTACCTCG------GCGACCACGAGGAGC---------------
---???GCTGGAACATCGGCTTCAGGG------ACGCCTAGGCAGCAAACGAAGCAG---
---AGGAAGCCA---------CACCGCTTCCGTCCAGGCACAGTGGCACTGCGGGAGATC
AGGAAATTTCAGAAAACCACCGAACTGCTGATCCCGTTTGCACCATTTTCTCGGCTGGTC
AGGGAGATCACTGATTTCTATTCAAAGGAT---------------------------GTG
TCACGGTGG---------------ACCCTTGAAGCTCTCCTTGCATTGCAAGAGGCAGCA
GAATACCACTTAGTGGACATATTTGAAGTGTCAAATCTCTGCGCCATCCATGCTAAGCGT
GTTACCATCATGCAAAAGGACATGCAACTTGCCAGGCGTATCGGTGGGCGGAGGCCATGG
------

Panicum_virgatum
ATGGCTCGCACCAAGCACCCGGCCGTGAGGAAATCG---------AAGGAGCAGCCCAAG
AAGAAGCTCCAGTTCGGGCGCTCCCCGCACGGG??????---ACGCCG------------
ACAGGTGGA---GCGAGCACATCG------GCGACTCCGGCAAGC------GCT???GGG
ACCGGGGAGAGAGCGGCGGCTGGAGGT------ACGGCGGGGCCGCAG---???CAGAAG
GTG???AAACCA---------CACCGTTGGAAGCCAGGGACTGTAGCGCTGCGGGAGATC
AGGAAGTTCCAGAAATCCACCGAGATGCTTATCCCCTTTGCACCATTTGCCCGTCTGGTG
AGGGAGATCACTGAGTTCTACTCAAGG???------------------------AATGTG
ACACGCTGG---------------ACCCCGGAAGCCATCCTTGCAATACAAGAGGCAGCA
GAATTCCACCTGATAGAACTGTTCGAAGTGGCAAATCTTTGTGCCATCCACGCCAAACGT
GTTACCATCATGCAAAGGGACATACAGCTTGCAAGGCGTATCGGTGGA---???CGCTGG
------

Saccharum_hybrid
ATGGCTCGAACCAAGCACCAGGCCGTGAGAAGGCCA---------ACGCAGAAGCCCAAG
AAGAAGCTCCAGTTCGAGCGC???------------------------------------
---GGTGGG---GCGAGTACCTCG------GCGACCCCGGAGAGA------AAT???GGG
ACCGGGGGAGGAGCGGCAGCTCGCGTT------ACACGGGGG???---------------
GTG???AAGAAG---------CTTCGCTGGCGGGCAGGGACTGTAGCGCTGCGGGAGATC
AGGAAGTACCAGAAGTCCACTGAGCCGCTCATCCCCTTTGCGCCCTTCGTACGCGTGGTT
AAGGAGTTAACTGGATTC---------------------------------------???
?????????AGGATAGGACGCTATACCCCTGAAGCCCTCCTTGCGCTGCAAGAGGCAGCA
GAATTCCACTTGATAGAACTGTTTGAAGTGGCGAATCTGTGTGCCATCCATGCCAAGCGT
GTCACAGTCATGCAAAAGGACATACAACTTGCAAGGCGTATCGGAGGA---???CGTTGG
GCG---

Sorghum_bicolor
ATGGCTCGAACCAAGCACCAGGCCGTGAGGAAGCTG---------CCGCAGAAGCCCAAG
AAGAAGCTCCAGTTCGAGCGC???------------------------------------
---GGTGGG---GCGAGTACGTCG------GCGACCCCGGAG???---AGGAAT???GGG
ACCGGGGGAGGAGCCGCGGCTCGCGTT------GCACGGGGG???---------------
GTG???AAGAAG---------CATCGCTGGCGGGCAGGGACTGTAGCGCTGCGGGAGATC
AGGAAGTACCAGAAGTCCACTGAGCCGCTCATCCCCTTTGCGCCCTTCGTACGTGTGGTC
AAAGAGTTAACTGCATTC---------------------------------------???
?????????AGGATAGGGCGCTACACCCCTGAAGCCCTCCTTGCGCTGCAAGAGGCAGCA
GAATTCCACTTGATAGAACTGTTTGAAGTGGCGAATCTGTGTGCCATCCATGCCAAGCGC
GTAACAGTCATGCAAAAGGACATACAACTTGCAAGGCGTATCGGAGGA---???CGTTGG
TCG---

Zea_mays
ATGGCTCGAACCAAGCACCAGGCCGTGAGGAAGACG---------GCGGAGAAGCCCAAG
AAGAAGCTCCAGTTCGAGCGC???------------------------------------
---GGTGGT---GCGAGTACCTCG------GCGACGCCGGAAAGG------GCT???GGG
ACCGGGGGAAGAGCGGCGTCTGGAGGTGACTCA---------------------------
??????AAGACGAAACCACGCCACCGCTGGCGGCCAGGGACTGTAGCGCTGCGGGAGATC
AGGAAGTACCAGAAGTCCACTGAACCGCTCATCCCCTTTGCGCCTTTCGTCCGTGTGGTG
AGGGAGTTAACCAATTTC---------------------------------------???
?????????AAAGTAGAGCGCTATACCGCAGAAGCCCTCCTTGCGCTGCAAGAGGCAGCA
GAATTCCACTTGATAGAACTGTTTGAAATGGCGAATCTGTGTGCCATCCATGCCAAGCGT
GTCACAATCATGCAAAAGGACATACAACTTGCAAGGCGTATCGGAGGA---???CGTTGG
GCA---

;
end;

BEGIN TREES;
			TREE tree = (Brachypodium_distachyon,Hordeum_marinum,(((Panicum_virgatum,(Cenchrus_americanus,(Zea_mays,(Saccharum_hybrid,Sorghum_bicolor)))),(Obrachyantha,(Osativa,Oaustraliensis))),(Hordeum_bulbosum,Hordeum_vulgare)));
end;
```

Primates

```
#NEXUS
[ Title ]
begin taxa;
     dimensions ntax=14;
     taxlabels
          Aotus
          Callithrix
          Colobus
          Gorilla
          Human
          Chlorocebus
          Lemur
          Macaca
          Nomascus
          Otolemur
          Pan
          Papio
          Pongo
          Saimiri
;
end;
begin characters;
     dimensions nchar=420;
     format missing=? gap=- matchchar=. datatype=nucleotide;
     matrix

Aotus
ATGGGCCCGCGCCGCCGGAGCCGCAAGCCTGAGGCCCCGAGGAGGCGCCGCGCGAGTCCG
------ACCCCCAGCCCCCCTCGGCGGGGCCCCTCCTTAGGCGCTTCCTCACGTCCACGT
GGTTATCGAAGGCAAGGTTGGCTAAAGGAGATCCGAAAGCTTCAGAAGAGCACACACCTC
TTGTTAAGGAAGTACCCCTTCAGCCGCCTGGCAAGAGAAATATGTGTTAAATTCACTCGT
GGTGTGGACTTCAATTGGCAAGCCCAGGCCCTATTGGCCCTACAAGAGGCGGCAGAAGCA
TTTCTAGTTCATCTCTTTGAGGATGCCTATCTCCTCACCTTACATGCCGGCCGAGTTACT
CTCTTCCCAAAGGACGTGCAACTGGCCCGGAGGATCCGGGGCATTGAGGAGGGACTCGGC


Callithrix
ATGGGCCCGCGCCGCCGGAGCCGCAAGCCCGAGGCCCCGAGGAGGCGCAGCGCGAGCCCG
------ACCCCCAGCCCCTCCCGGCGGGGCCCCTCCTTAGGTGCTTCCTCACGTCCACGT
GGTTATCGAAGGCAAGGTTGGCTAAAGGAGATCCGAAAGCTTCAGAAGAGCACACACCTC
TTGTTAAGGAAGTACCCCTTCAGCCGCCTGGCAAGAGAAATAAGTGTTAAATTCACTCGT
GGTGTGGACTTCAATTGGCAAGCCCAGGCCCTATTGGCCCTACAAGAGGCTGCAGAAGCA
TTTCTAGTTCATCTCTTTGAGGATGCCTATCTCCTCACCTTACATGCCGGCCGAGTTACT
CTCTTCCCAAAGGATGTGCAACTGGCCCGGAGGATCCGGGGCATTGAGGGAGGACTCGGC


Colobus
ATGGGCCCGCGCCGCCGGATTCGCAAGCCCGAGGCCCCGAGGAGGCGCAGCCCGAGCCCG
------ACCCCCGGCCCCTCCCGGCGGCGCCCCGCCTTAGGCCCTTCCTCCCGTCAACAT
GGTCGGCGGAGACAAGCTTGGCTAAAGGAGATCCGAAAGCTTCAGAAGAGCACACACCTC
TTGATAAGGAAGTACCCCTTCAGCCGCCTGGCAAGAGAAATATGTGTTCAATTCACTCGT
GGTGTGGACTTCAATTGGCAAGCCCAGGCCCTATTGGCCCTACAAGAGGCAGCAGAAGCA
TTTCTAGTTCATCTCTTTGAAGATGCCTATCTCCTCACCTTACATGCCGGCCGAGTTACT
CTCTTCCCAAAGGATGTGCAACTGGCCCGGAGGATCCGGGGCATTGAGGCGGGACTCGGC


Gorilla
ATGGGCCCGCGCCGCCGGAGCCGAAAGCCCGAGGCCCCGAGGAGGCGCAGCCCGAGC???
ACCCCG???CCCGGCCCCTCCCGGCGGGGCCCCTCCTTAGGCGCTTCCTCCCATCAACAC
AGTCGGCGGAGACAAGGTTGGCTAAAGGAGATCCGAAAGCTTCAGAAGAGCACACACCTC
TTGATAAGGAAGCTCCCCTTCAGCCGCCTGGCAAGAGAAATATCTGTTAAATTCACTCGT
GGTGTGGACTTCAATTGGCAAGCCCAGGCCCTATTGGCCCTACAAGAGGCAGCAGAAGCA
TTTCTAGTTCATCTCTTTGAGGACGCCTATCTTCTCACCTTACATGCTGGCCGAGTTACT
CTCTTCCCAAAGGATGTGCAACTGGCCCGGAGGATCCGGGGCCTTGAGGAGGGACTCGGC


Human
ATGGGCCCGCGCCGCCGGAGCCGAAAGCCCGAGGCCCCGAGGAGGCGCAGCCCGAGC???
ACCCCG???CCCGGCCCCTCCCGGCGGGGCCCCTCCTTAGGCGCTTCCTCCCATCAACAC
AGTCGGCGGAGACAAGGTTGGCTAAAGGAGATCCGAAAGCTTCAGAAGAGCACACACCTC
TTGATAAGGAAGCTGCCCTTCAGCCGCCTGGCAAGAGAAATATGTGTTAAATTCACTCGT
GGTGTGGACTTCAATTGGCAAGCCCAGGCCCTATTGGCCCTACAAGAGGCAGCAGAAGCA
TTTCTAGTTCATCTCTTTGAGGACGCCTATCTCCTCACCTTACATGCAGGCCGAGTTACT
CTCTTCCCAAAGGATGTGCAACTGGCCCGGAGGATCCGGGGCCTTGAGGAGGGACTCGGC


Chlorocebus
ATGGGCCCGCGCCGCCGGAGCCGCAAGCCCGAGGCCCCGAGGAGGCGCAGCCCGAGCCCG
------ACCCCCGGCCCCTCCCGGCGGGGCCCCTCCTTAGGCCCTTCCTCCCATCAACAT
GGTCGGCGGAGACAAGCTTGGCTAAAGGAGATCCGAAAGCTTCAGAAGAGCACACACCTC
TTGATTAGGAAGTACCCCTTCAGCCGCTTGGCAAGAGAAATATGTGTTAAATTCACTCGT
GGTGTGGACTTCAATTGGCAAGCCCAGGCCCTATTGGCCCTACAGGAGGCAGCAGAAGCA
TTTCTAGTTCATCTCTTTGAAGATGCCTATCTCCTCACCTTACATGCCGGCCGAGTTACT
CTCTTCCCAAAGGATGTGCAACTGGCCCGGAGGATCCGGGGCATTGAGGCGGGACTCGGC


Lemur
ATGGGCCCGCGCCGCCGGAGGCGCAAGCCCGAGACCCCGACGAGGCGCGTCGAGAGCCCG
------ACCCCCGGCCCCTCGCGGCGGCGGCCCCCCGTAGCCCCTACCCCCCGTCAACAT
CGTCGGAGGACATTTGGATGGTTACAGGAGATCCGAAAACTTCAGAAGAGCACACACCTC
TTATTAAGGAAGGCCCCCTTCAGCCGCGTGGCAAGAGAAGTATGTACTAAATTCACTCGT
GGTGTGGACTTCAATTGGCAAGCCCAGGCCCTGTTGGCCCTACAAGAGGCTGCAGAAGCA
TTTCTAGTCCATCTCTTTGAGGATGCTTATCTCCTCACCCTACATGCCGGCCGAGTTACT
CTTTTCCCAAAGGACGTGCAGCTGGCCAGGAGGATCCGAGGCATTCAGGAAGGGCTCGGC


Macaca
ATGGGCCCGCGCCGCCGGAGCCGCAAGCCCGAGGCCCCGAGGAGGCGCAGCCCGAGCCCG
------ACCCCCGGCCCCTCCCGGCGGGGCCCCTCCTTAGGCCCTTCCTCCCGTCAACAT
GGTCGGCGGAGACAAGCTTGGCTAAAGGAGATCCGAAAGCTTCAGAAGAGCACACACCTC
TTGATAAGGAAGTACCCCTTCAGCCGCCTGGCAAGAGAAATATGTGTTAAATTCACTCGT
GGTGTGGACTTCAATTGGCAAGCCCAGGCCCTATTGGCCCTACAAGAGGCAGCGGAAGCA
TTTCTAGTTCATCTCTTTGAAGATGCCTATCTCCTCGCCTTACATGCCGGCCGAGTTACT
CTCTTCCCAAAGGATGTGCAACTGGCCCGGAGGATCCGGGGCATTGAGGCGGGACTCGGC


Nomascus
ATGGGCCCGCGCCGCCGGATCCGCAAGCCCGAGGCCCCGAGGAGGCGCAGCCCGAGTCCG
------ACCCCCGGCCCCTCCCGGCGGGGCCCCTCCTTAGGCGCTTCCTCCCGTCAACAC
GGTTGGCGGAGACAAGGTTGGCTAAAGGAGATTCGAAAGCTTCAGAAGAGCACACACCTC
TTGATAAGGAAGCTCCCCTTCAGCCGCCTGGCAAGAGAAATATGTGTTAAATTCACGCGT
GGTGTGGACTTCAATTGGCAAGCCCAGGCCCTACTGGCCCTACAAGAGGCAGCAGAAGCA
TTTCTAGTTCATCTCTTTGAGGATGCCTATCTCCTCACCTTACATGCCGGCCGAGTTACT
CTCTTCCCAAAGGATGTGCAACTGGCCCGGAGGATCCGGGGCCTTGAGGAGGGACTCGGC


Otolemur
ATGGGCCCG???---CGGAGACGCAAGCCCGAAACCCCGACGAGGCGCCCCGCGAGCCCG
------AGCCCCGGCCCCTCGCGGCGGGGCCCTTCCCTAGGCACTTCTTCCCGAGGACAT
GGTCAGAGGAGACCTTTGTGGTTAAAGGAGATCCGAAAACTTCAGAAGAGCACAAACCTC
TTGTTAAGGAAAGCCCCCTTCAGCCGCCTGACAAGAGAAATATGTGGTAAATTCACTCGT
GGTGTGGACTACAATTGGCAAGCGCAGGCCCTGTTGGCCCTACAAGAGGCAGCAGAAGCA
TTTCTAGTTCATCTCTTTGAAGATGCTTACCTTCTCACCTTACATGCTGGCCGAGTTACT
CTTTTCCCAAAGGATGTGCAACTGGCAAGAAGGATCCGAGGCATTCAGGAAGGGCTGGGC


Pan
ATGGGCCCGCGCCGCCGGAGCCGCAAGCCCGAGGCCCCGAGGAGGCGCAGCCCGAGCCCG
------ACCCCCGGCCCCTCCCGGCGGGGCCCCTCCTTAGGCGCTTCCTCCCATCAACAC
AGTCGGCGGAGACAAGGTTGGCTAAAGGAGATCCGAAAGCTTCAGAAGAGCACACACCTC
TTGATAAGGAAGCTCCCCTTCAGCCGCCTGGCAAGAGAAATATGTGTTAAATTCACTCGT
GGTGTGGACTTCAATTGGCAAGCCCAGGCCTTATTGGCCCTACAAGAGGCAGCAGAAGCA
TTTCTAGTTCATCTCTTTGAGGACGCCTATCTCCTCACCTTACATGCTGGCCGAGTTACT
CTCTTCCCAAAGGATGTGCAACTGGCCCGGAGGATCCGAGGCCTTGAGGAGGGACTTGGC


Papio
ATGGGCCCGCGCCGCCGGAGCCGCAAGCCCGAGGCCCCGAGGAGGCGCAGCCCGAGCCCG
------ACCCCCGGCCCCTCCCGGCGGGGCCCCTCCTTAGGCCCTTCCTCCCGTCAACAT
GGTCGGCGGAGACAAGCTTGGCTAAAGGAGATCCGAAAGCTTCAGAAGAGCACACACCTC
TTGATAAGGAAGTACCCCTTCAGCCGCCTGGCAAGAGAAATATGTGTTAAATTCACTCGT
GGTGTGGACTTCAATTGGCAAGCCCAGGCCCTATTGGCCCTACAAGAGGCAGCAGAAGCA
TTTCTAGTTCATCTCTTTGAAGATGCCTATCTCCTCACCTTACATGCCGGCCGAGTTACT
CTCTTCCCAAAGGATGTGCAACTGGCCCGGAGGATCCGGGGCATTGAGGCGGGACTCGGC


Pongo
ATGGGCCCGCGCCGCCGGAGCCGCAAGCCCGAGGCCCCGAGGAGGCGCAGCCCGAGC???
ACCCCG???CCCGGCCCCTCCCGGCGGGGCCCCTCCTTAGGCGCTTCCTCCCATCAACAC
AGTCGGCGGAGACAAGCTTGGCTAAAGGAGATCCGAAAGCTTCAGAAGAGCACACACCTC
TTGATAAGGAAGCTCCCCTTCAGCCGCCTGGCAAGAGAAATATGTGTTAAATTCACTCGT
GGTGTGGACTTCAATTGGCAAGCCCAGGCCCTATTGGCCCTACAAGAGGCAGCAGAAGCA
TTTCTAGTTCATCTCTTTGAGGACGCCTATCTCCTCACCTTACATGCCGGCCGAGTTACT
CTCTTCCCAAAGGATGTGCAACTGGCCCGGAGGATCCGGGGCCTTGAGGAGGGACTCGGC


Saimiri
ATGGGCCCGCGCCGCCGGAGTCGCAAGCCCGAGGCCCCGAGGAGGCGCAGCCCGAGCCCG
------ACCCCCGGCCCCTCCCGGCGGGGCCCC???---GGCGCTTCCTCACGTCCACGT
GGTTATCGAAGGCAAGGTTGGCTAAAGGAGATCCGAAACCTTCAGAAGAGCACACACCTC
TTGATAAGGAAGTACCCCTTCAGCCGCCTGGCCAGAGAAATATGTGTTAAATTCACTCGT
GGTGTGGACTTCAGTTGGCAAGCCCAGGCCCTATTGGCCCTACAAGAGGCGGCAGAAGCA
TTTCTAGTTCATCTCTTTGAGGATGCCTATCTCCTCACCTTACATGCCGGCCGAGTTACT
CTCTTCCCAAAGGATGTGCAACTGGCCCGGAGGATCCGGGGCATTGAGGAGGGACTCGGC


;
end;

BEGIN TREES;
                    TREE tree = (Macaca,Papio,(Chlorocebus,(Colobus,(((Lemur,Otolemur),(Saimiri,(Callithrix,Aotus))),(Nomascus,(Pongo,(Gorilla,(Pan,Human))))))));
END
```

Saccharomyces

```
#NEXUS
[ Title ]
begin taxa;
    dimensions ntax=9;
    taxlabels
        S_arb
        S_bay
        S_cas
        S_cer
        S_klu
        S_kud
        S_mik
        S_par
        S_pas
;
end;
begin characters;
    dimensions nchar=1146;
    format missing=? gap=- matchchar=. datatype=nucleotide;
    matrix

S_arb
ATGTCAAGCAAACAGCAATGG???---------ATCCAAAGTGATTCGAGT---------
------------------------------------------------------------
------------------------GGGAGATCTCTCAGTAATGTCAATAGGCTTGCA---
------------------------------------------GGAGAT------------
CAACAGTCTATTAACGACCGTGCGCTATCTTTATTGCAAAGGACAAGAGCAAGGAAGAAT
CTTTTCCCAAGAAGGGAGGAAAAGAGACGATATGAAGGTCCA------------CAGGAT
GACATAGATTTTGAAGCA------------------------------------------
------------------------GAGTATGGAAACCAGGCAGGAAATTTGGAAACTGAG
ACAGAAAATGAGGATGAAACTGAGATGGAAACTGAAGCACCAACTGCCACGATAACTCAT
TCTTATGCCCTAGATAGGTACGTTAGGCAA------------------------------
---------------------------AAAAGAAGACAAAAGCAAAGAAGGCAAGACCTG
AAG------------------------------------------------------CGC
GTCGAA------------------------------------------------------
------------------------------------------------------------
------AAGAAGTATAGCCCTAGTGAGTTAGCTCTGTATGAAATTCGAAAATACCAGCGT
TCTACAGATTTACTTATCTCCAAAATTCCATTCGCAAGGTTAGTGAAGGAAGTTACGGAT
GAATTTACAACTAAAGACCAAGATTTGCGTTGGCAGTCAATGGCGATCATGGCGTTACAA
GAAGCAAGTGAAGCATACTTGGTGGGGTTATTGGAGCACACCAACCTCTTGGCGTTGCAT
GCGAAAAGAATCACCATAATGAAGAAGGATATGCAACTCGCAAGGAGAATCAGAGGACAG
TTTATT

S_bay
ATGTCTAGCAAACAAGAATGGGCTAACTCGGCCATTCAAAGTGAATCGAGT---------
------------------------------------------------------------
------------------------GGAAGATCACTCAGTAATGTCAATAGGCTTGCA---
------------------------------------------GGAGAT------------
CAACAGTCGATTAATGATCGTGCGTTATCCTTGTTACAAAGAACAAGAGCAAGGAAGAAC
CTGTTCCCAAGAAGAGAAGAAAGAAGACGGTATGAAGGTTCA------------CAGGAT
GACGTAGAATTTGAGGCA------------------------------------------
------------------------GATTACGAAGACCATGCAGGAAATCTCGAAACTGAA
GCAGAAAACGAAAATGAAACAGAAGTAGAAGAAGAATTATCGACGGCTAAGCAAACTCAC
TCGTACGCCTTAGATAGATATGTTAGACAA------------------------------
---------------------------AAAAGAAGACAAAAACAAAGGAAGCAGGGCTTG
AAG------------------------------------------------------CGT
GTGGAA------------------------------------------------------
------------------------------------------------------------
------AAGAAATATAGCCCTAGCGAATTAGCTCTTTACGAAATTCGAAAATACCAGCGT
TCCACAGATTTGCTAATCTCCAAAATCCCATTTGCAAGACTAGTCAAGGAAGTCACGGAT
GAGTTTACAACCAAAGATCAAGACCTGCGTTGGCAGTCAATGGCGATCATGGCGCTACAG
GAAGCAAGTGAAGCATATTTAGTGGGATTATTAGAGCATACCAACCTCTTAGCATTGCAT
GCGAAAAGGATAACCATAATGAAAAAAGACATGCAACTGGCAAGGAGAATTAGAGGACAA
TTCATT

S_cas
ATGTCAAGCAGA---------------------------------------AAATTTGTA
GAACAAGGCCATGCTCAGAATTCTAGTAGTCATCTGTTCTCGAATATCCTCGATAATGAT
------------------------???GGATCATTGAGCAATATAAATAGATTA???CTG
GACCCGGATAATACAGAAGATTTATTACAGCAAGAAGTC---------------------
---------ATCAACGAGCGGGCACTTTCTCTTTTACAAAGGACTAGAGAACGTAGAAAC
TTATTACACCGATTTGAAGATAAAAGAAGGTAT---------TATAACCAAGGG??????
???????????????---TCTGTGGCCAGTAGTCATTATCGTTCTAATGATGTGGGTGGG
AACTTTCAATTTTTTGATCAAGAA????????????????????????------------
------------------------------------------------------------
------------------------------GCCATTGATGACGATTATGGAACCCTAGAT
CAATCCAACATTATCGATCGTCACCAA?????????------------------------
---AAAGGAAACAAACATTCTCGTCAAGAAAGACACCATCAAAGAGAACTCAAACAACGT
GTTGAAAAGATTAGAACTCAGAGACAGGTCGGAAATACG---------------------
------------------------------------------------------------
------???AAATTTACTCCAAGTAGTTTAGCCCTATATGAGATAAGGAAATACCAACGA
TCTTCAGAACTGTTGATATCGAAGATTCCCTTTACTAAATTAGTGAAGGAAGTCACTGAT
GAATTTACCGTGGAAGACCAACAACTTCATTGGCAATCGATGGCCATTGTCGCGTTACAG
GAAGCCAGTGAAGCTTATTTAGTGGGTTTATTGGAGCATGCCAATTTATTAGCCATACAT
GCCAAGAGGATCACATTAATGAAGAAGGATATACAGTTGGCCAGAAGAATTAGAGGTCAA
TTCATT

S_cer
ATGTCAAGTAAACAACAATGGGTTAGTTCTGCTATTCAAAGTGATTCGAGT---------
------------------------------------------------------------
------------------------GGAAGATCACTCAGTAACGTCAACAGGCTTGCA---
------------------------------------------GGAGAC------------
CAACAATCTATTAACGATCGTGCGTTATCGTTATTGCAGAGAACAAGAGCGACAAAGAAC
CTGTTTCCAAGAAGAGAGGAAAGAAGACGTTATGAAAGCTCA------------AAAAGT
GACCTAGATATCGAAACA------------------------------------------
------------------------GACTACGAAGACCAAGCAGGTAATCTAGAAATCGAG
ACAGAAAATGAAGAAGAAGCTGAAATGGAAACTGAAGTACCTGCACCAGTGCGAACTCAT
TCATATGCCTTAGACAGATATGTTAGACAG------------------------------
---------------------------AAAAGGAGGGAAAAACAAAGAAAGCAGAGCTTA
AAG------------------------------------------------------CGC
GTCGAA------------------------------------------------------
------------------------------------------------------------
------AAGAAATATACTCCTAGTGAATTAGCTCTGTACGAAATTCGAAAATACCAACGT
TCCACGGATTTATTAATCTCCAAAATTCCATTTGCAAGGCTAGTGAAAGAAGTTACAGAC
GAGTTTACAACTAAAGATCAGGATTTACGTTGGCAGTCAATGGCGATTATGGCGTTACAG
GAAGCAAGCGAAGCGTATCTGGTAGGATTATTGGAACATACAAACCTCTTGGCGCTGCAT
GCAAAAAGAATTACTATAATGAAGAAAGACATGCAACTAGCAAGAAGAATCAGGGGACAG
TTTATT

S_klu
ATG---???????????????------------------------------------------
------------------------------------------------------------
AATGCTAGTATATCCAGCCAGACAGGCAGATCACTCAGTAACACCAATCGTCTG------
---------------------------------------GGC???---CAAGAGCCCTCT
CAACAATCTATCAACGAGAGGGCAATATCGCTTCTACAGCGTAATAGGGCCAGGAGGAAG
ATGTTACAAAGGCAAGAGGATAGAGAGCGATAC---------------------------
------------------------------------------------------------
------------------------------------------------------------
------------------------------------------------------------
------------------------------------------------------------
------------------------------------------------------------
------------------------------------------------------------
---------------------------------------GTAAGGCGTAATGAACTACCA
GAGGAACAGCCTCCAACTGCGATAGGTACCATCAGAAGCAATATAAAAAAATCCAAGAGT
CAGCGT??????TACAAACCAAGTGATGTGGCTTTGCAAGAGATTAGAAAGTACCAACGA
AGCACTGAGCTTTTGATATCGAAAATGCCATTTGCCCGTCTCGTAAAAGAGGTCACTGAA
CAATTTAGTACAGACGAACAGCAGCTCCGGTGGCAGTCTATGGCTATCCTAGCATTACAG
GAGGCTAGTGAAGCGTACTTGGTTGGTCTACTGGAGCACACAAACCTGTTAGCCTTACAC
GCTAAAAGAATTACCATTATGAAGAAAGATATGCAGCTGGCAAGGAGAATTAGGGGCCAG
TTTATT

S_kud
ATGTCAAGCAAACAGCAATGGGCTAATTCGGCCATTCAAAGTGATTCGAGT---------
------------------------------------------------------------
------------------------GGAAGGTCACTCAGCAATGTGAACAGGCTAGTA---
------------------------------------------GAAGAT------------
CAACAGTCTATTAATGACCGTGCACTATCTTTGTTACAAAGAACAAGAGCAAGAAAAAAT
CTTTTTCCAAAAAGAGAAGAAAGAAGACGATATGAAGGTCCA------------CAAGAT
GACATGATTTTTGAAGAA------------------------------------------
------------------------AACCATGAAGGCCAAGCGGAAAACTTGGAAACAGAG
ACAGAAAACGAGGATGAACGTGAAATGGAAACAGAGGTTCCAGACGCTACCCGAACACAT
TCATACGCTCTAGATAGATATGTTAGGCAG------------------------------
---------------------------AAAAGGAGACAAAAACAAATGAAGCAGGGTTTG
AAA------------------------------------------------------CGC
GTAGAA------------------------------------------------------
------------------------------------------------------------
------AAGAAATACAGTCCCAGTGAATTAGCTCTGTACGAAATCAGAAAATATCAACGT
TCTACAGATTTGCTTATCTCTAAAATTCCATTTGCAAGACTGGTAAAAGAAGTTACAGAC
GAGTTTACAACTAAAGACCAAGATTTGCGCTGGCAATCGATGGCGATCATGGCGCTACAG
GAGGCAAGTGAAGCATACTTGGTGGGGCTATTGGAGCATACCAACCTACTTGCGCTGCAT
GCGAAGAGAATAACCATAATGAAGAAGGATATGCAACTAGCAAGGAGAATTAGAGGACAG
TTCATT

S_mik
ATGTCAAGCAAACAGCAATGGGCTAATTCTGCTATTCAAAGTGATTCAAGT---------
------------------------------------------------------------
------------------------GGAAGATCACTCAGCAATGTCAACAGACTTGTT---
------------------------------------------GGAGAG------------
CAACAATCCATTAACGATCGTGCATTATCATTATTGCAAAGGACAAGAGCAAGAAAGAAT
CTATTTCCAAGAAGAGAAGAAAAGAGACGATATGAGAACCCA------------AAGGAT
GACGTAGATTTTGAAACT------------------------------------------
------------------------GACTATGATGGCCAAGCAGATGATTTGGAGATAGAA
ACTGAGAACGAAGAAGAAACTGAAGTGGCTACTGAAGTATCAACGTCCGCGCGAACACAT
TCGTATGCCTTGGACAGATACGTTAGACAG------------------------------
---------------------------AAAAGAAGAGAAAAGCAAAGGAAACAGGGCTTA
AAG------------------------------------------------------CGT
ATTGAA------------------------------------------------------
------------------------------------------------------------
------AAGAAGTACAGTCCAAGTGAATTAGCTCTATATGAAATTCGAAAGTACCAACGT
TCCACGGATTTATTGATTTCCAAAATTCCTTTTGCAAGGCTTGTAAAAGAAGTTACGGAT
GAGTTCACGACTAAGGATCAAGATTTACGCTGGCAGTCAATGGCGATCATGGCACTACAA
GAAGCAAGTGAAGCCTATTTGGTGGGTCTATTGGAGCATACCAACCTTTTGGCATTGCAC
GCGAAAAGGATAACTATAATGAAGAAGGACATGCAACTAGCAAGGAGAATCAGAGGACAA
TTCATT

S_par
ATGTCAAGTAAACAGCAATGGGCTAGTTCTGCTATTCAAAGTGATTCGAGT---------
------------------------------------------------------------
------------------------GGAAGATCACTCAGTAATGTCAACAGGCTTGCA---
------------------------------------------GGAGAC------------
CAGCAGTCTATTAACGATCGTGCGTTATCGTTACTGCAAAGGACGAGAGCAAGAAAGAAC
TTACTACCCAGAAGAGAAGAAAGAAGGCGTTATGAGAGCTCA------------AAAAAT
GACCTAGATTTTGAAACA------------------------------------------
------------------------GACTACGAAGACCAAGCAGGTAATCTGGAAACTGAA
ACAGAGAATGAAGAAGAAGCTGGAATGAAAACCAAGGCACCAATGTCCGCCCGAACTCAT
TCGTATGCTTTAGACAGATACGTTAGGCAG------------------------------
---------------------------AAAAGAAGGGAAAAGCAAAGAAAGCAGGGCTTG
AAG------------------------------------------------------CGT
GTCGAA------------------------------------------------------
------------------------------------------------------------
------AAGAGATATAGTCCTAGTGAATTAGCTCTGTACGAAATTCGAAAATATCAACGT
TCTACAGATTTATTAATTTCCAAAATTCCATTTGCAAGGCTAGTGAAAGAGGTTACAGAT
GAGTTTACAACCAAAGATCAAGATTTACGTTGGCAGTCAATGGCGATTATGGCGCTGCAG
GAGGCAAGTGAAGCATACTTAGTAGGGTTACTGGAACATACCAACCTCTTGGCGTTGCAT
GCGAAAAGAATAACTATAATGAAGAAAGATATGCAACTAGCAAGAAGAATTAGGGGACAG
TTCATT

S_pas
ATGTCTAGCAAACAAGAATGGGCTAACTCGGCCATTCAAAGTGAATCGAGT---------
------------------------------------------------------------
------------------------GGAAGATCACTCAGTAATGTCAACAGGCTTGTA---
------------------------------------------GGAGAC------------
CAACAATCGATTAATGATCGTGCGTTATCCTTGCTGCAAAGAACAAGAGCAAGGAAGAAC
CTGTTTCCAAGAAGAGAAGAAAGAAGACGATATGAAGGTCCT------------CAGGAC
GACGTAGAATTTGAGGCA------------------------------------------
------------------------GATTATGAGGATCATGCAGGAAATCTCGAAACGGAA
ACAGAAAACGGAGATGAAACTGAAGTAGAAGCAGATGTGTCAACGGTTAAGCAAACTCAC
TCGTACGCCTTAGATAGATATGTTAGACAA------------------------------
---------------------------AAACGGAGACAAAAACAAAGGCAGCAGGGCTTG
AAG------------------------------------------------------CGT
GTGGAA------------------------------------------------------
------------------------------------------------------------
------AAGAAATATAGTCCTAGCGAATTAGCTCTCTACGAAATTCGAAAATACCAGCGT
TCTACAGATTTACTAATCTCTAAAATTCCATTTGCAAGATTAGTTAAGGAAGTCACGGAT
GAGTTTACAACCAAAGATCAGGACTTGCGTTGGCAGTCTATGGCGATCATGGCGCTCCAA
GAAGCAAGTGAGGCATATTTGGTGGGATTATTAGAGCATACCAACCTCTTAGCTTTACAT
GCGAAAAGGATAACCATAATGAAAAAAGACATGCAACTAGCAAGGAGAATTAGAGGACAA
TTTATT

;
end;

BEGIN TREES;
                TREE tree = (S_arb,S_kud,(((S_bay,S_pas),(S_cas,S_klu)),(S_mik,(S_cer,S_par))));
END;
```

Tetrahymena

```
#NEXUS
[ Title ]
begin taxa;
     dimensions ntax=13;
     taxlabels
          borealis
          canadensis
          elliotti
          furgasoni
          leucophrys
          limacis
          malaccensis
          mimbres
          mobilis
          rostrata
          silvana
          thermophila
          tropicalis
;
end;
begin characters;
     dimensions nchar=516;
     format missing=? gap=- matchchar=. datatype=nucleotide;
     matrix

borealis
AAGAGGAGAAGCGTTTCTAATTAATCTCAATAAAGATCAAATAATGCTACA???AATAAT
TAAGTA---AATTAAAGATCGAAATCTGCAGGT---GGATAAATGAAT---AAATAAAAA
AACCCA---TCTTAACAGTAAAATAAGCCTGTAACA------AAAAAGAGATAATCCTCT
GGTGAAAAATATGAATCTGCAAGAGATAAAGTAATTAGAGGATTCAGACCAGGAGATAAT
GCTTTGAAAGAACTTAAATAATATAATTAAAATCCATCACTATTTATTCGAAAACTACCT
TTTTAGAGGTTAGTGAGAGAAATATCAACACGATTGTCAGATCAGGATTAAATTAGATGG
ACAAGTATTGCTCTAATCCTATTATAAACAGTTACAGAAGATTATATGATATCTTTTTAT
GAAGATGCGAATGCATGTGCTTTACACGCTAAAAGAGTTACCCTTATGAGCAAAGATATG
ATTCTAGCTGCTAGAATTAGAGGATAAAGGAATGTT

canadensis
AAGAGGAGAAGTGTTTCTAATTAATCTCAACAAAGATCAAATAATGCAACA???AATAAT
TAAGTA---AATTAAAGATCGAAATCTGCAGGT---GGATAAATGAAT---AAATAAAAA
AACCCA---TCTTTACAGTAAAATAAGCCTGTAACT------AAAAAGAGATAATCCTCT
GGTGAAAAATATGAATCTGCAAGAGATAAAGTAATTAGAAGATTTAGACCAGGAGATAAT
GCTTTGAAAGAACTTAAATAATATAATTAAAATCCATCACTATTTATTCGAAAACTACCT
TTTTAGAGGTTAGTGAGAGAAATATCAACACGATTGTCAGATTAGGATTAAATTAGATGG
ACAAGTATTGCTCTAATTCTATTATAAACTGTTACAGAAGATTATATGATATCATTTTAT
GAAGATGCGAATGCATGCGCTTTACATGCTAAAAGAGTTACCCTTATGAGCAAAGATATG
ATTCTTGCTGCTAGAATTAGAGGATAAAGGAATGTT

elliotti
AAAAGAAGAAGCAATTCTAACTAAAATTAAGAGAGGTCTGAGAGTTCT---AAAAAAATT
AAATAAGATAATTAAAGATCTAAATCAGCGGGAATA??????GATAAT---AAA??????
??????---???????????????????????AGCACA???---AAAAGATAATCATCA
GAGGAAAGATATGAATCAGCAAGAGATAAAGTTATCAGAAGATTTAGACCTGGTGATAAT
GCTCTTAAATAATTAAGATAATATAATCAAACTCCTTCGCTGCTTATAAGAAAATTGCCT
TTTTAAAGGCTAATAAGGGAAATTTCTACAAGAATGACAGAAGAAGATCAGTTAAGATGG
ACAAGTTTTGCTTTAGTTTTACTTTAAACTGTTGTAGAAGATTATATGGTTTCATTTTTT
GAAGATGCAAATGCTTGTGCTCTACATGCTAAAAGAGTAACTCTAATGTCTAAGGATCTT
GCTCTTGCAGCAAGAATAAGGGGATAAAAAAATGTT

furgasoni
AAAAGGAGGAGCAATTCTAACCAAAATTAATCATCATCTAATAATGTT---GCTAATAAT
TAATCTGCTAATCAGAGATCAAAATCAGCAGGG---CCATAAGGGAAT---AAATAAAAA
AATTAG---CCTCAATAATAAAAAAAGGCTGTTTCC------AAAAAGAGATAATCATCT
GGTGAGAAATATGAATCGGCAAGAGATAAAGTAATAAGAAGATTTAGGCCAGGGGATAAT
GCATTAAAAGAAATGAGATAATATAATCAAACTCCATCTCTACTAATAAGAAAATTGCCT
TTTTAGAGGCTAGTAAGGGAAATATCAACAAGATTGACAGAGGAAGATAATTTGAGATGG
ACAAGCTTTGCCTTAGTGTTATTATAAACTGTTGTTGAAGATTACATGGTATCATTTTAT
GAAGATGCAAATGCATGTGCTTTACATGCTAAAAGAGTTACCTTAATGACAAAAGATTTA
GCACTAGCTGCAAGAATTAGAGGATAAAAAAATGTT

leucophrys
AGAAGAAGGAGTAATTCAAATCAAACTTAATAAAGATCATAAAGCGCT---TCCAATAAC
CCATAACCACAATAAAGATCAAAATCATCAGGA---AATCAAGAAAAT---AAATAAGCG
AAAGCA---AAGCAATAATAAAAGAAACCAACTACAACAAAGAAAAAGAGATAGTCTTCA
GGTGATAAGTATGAATCTGCTAGGGATAAAGTGATAAGAAGATTTAGACCTGGAGATAAT
GCCTTAAAAGAATTAAGATAATATAACTAAACCCCATCATTATTAATCAGAAAATTACCT
TTTTAGAGACTAGTGAGAGAAATATCAACAAGAATGTCAGAGCAAGAGTAATTAAGATGG
ACCAGTTTTGCTTTGGTTCTATTATAGACAGTTGTGGAGGACTATATGGTCTCTTTCTAT
GAAGATGCTAATGCCTGT??????????????????????????????????????????
????????????????????????????????????

limacis
AAAAAGAGGAGTAATTCTAATTAAAGCTAATAAAGATAGAGTAGTGCA---AATAATGAT
TAATCAACTAATCGAAGATCTAAATCTGCAGGA---AACCAAGGGAAT---AAATAAAAG
GGTTTA---CCTCAATAATAAAAAAAGGCTGTTTCTACGAAAAAAAAGAGATAATCTTCT
GGTGAGAAGTATGAATCTGCTAGAGATAAAGTAATAAGAAGATTTAGACCTGGAGATAAT
GCACTAAAAGAACTCAGATAATATAATTAAACTCCATCATTATTAATTAGAAAATTACCT
TTTTAGAGATTAGTAAGAGAAATATCAACCAGAATGACAGAAGAGGATTCGCTGAGATGG
ACTAGTTTTGCATTAGTATTATTGTAAACAGTTGTTGAAGATTATATGGTATCATTTTAT
GAAGATGCTAATGCATGT??????????????????????????????????????????
????????????????????????????????????

malaccensis
AAAAGGAGGAGTAATTCAAATTAAGATTAATAGAGATCGGGTAGCTAG---AAAAAAAAT
AAATAGGATAATTTAAGATCTAAATCTGCTGGGATA??????GAGAATGAAAAA??????
??????---???????????????????????AGCACAAAAAAGAAAAGAGAATCATCT
GGCGAAAAATATGAATCTGCAAGAGATAAAGTTATAAGAAGATTTAGGCCAGGAGATAAT
GCTCTTAAATAATTAAGATAATATAATTAGACTCCATCATTGCTTATAAGAAAATTACCC
TTTTAGAGGTTGATAAGAGAGATTTCTACAAGAATGACAGAAGAGGATTAGCTAAGATGG
ACTAGTTTTGCTCTTGTTTTACTTTAAACAGTTGTAGAAGATTATATGGTTTCCTTTTTT
GAGGATGCTAATGCTTGTGCTCTCCATGCTAAAAGAGTAACTCTAATGTAAAAAGATCTT
GCCCTTGCTGCAAGAATAAGGGGATAAAAAAATGTT

mimbres
AGCAAAAGAAGTAATTCAAACCAGCCATAGTAAAAATCTAATAAATAAATAAGCAATATT
TAAGAAAATAAAAGACGATCAAAATCTGCAGGT---GGGCCAACTAAT---AAACCAAAA
AATCCA---CCTTAGTAATAAAAAAAGGTAGCCACATCAAATAAAAAGAGATAATCATCT
GGTGAAAAGTATGAATCTGCTAGAGATAAGGTAATAAGAAGATTTAGACCAGGTGATAAT
GCTCTCAAAGAGCTCAGATAATATAATTAGAATCCTTAGTTATTAATAAGAAAATTACCT
TTTTAGAGGTTAGTAAGAGAAATAACGTAAAGGATGACAGTAGAAGAATAGTTGAGATGG
ACAAGTTTTGCCCTAGTTATATTATAAACAATATCTGAAGATTATATGGTCTCATTTTTT
GAAGATGCAAATGCTTGTTGCTATCATGCTAAAAGAGTTACTCTTATGACAAAAGACATG
GCTCTTGCTGCTAGAATTAGAGGATAAAAAAATGTT

mobilis
AAAAGGAGAAGCAATTCTAACCAAAATTAATCAGCATCAAATAATGTT---TCAAATAAT
TAATCTGCAAATCAGAGATCAAAGTCAGCAGGA---CCATAAGGGAAT---AAATAAAAA
AATTAG---CCTCAATAATAAAAAAAGGCTGTTTCC------AAAAAGAGATAATCATCA
GGTGAAAAATATGAATCAGCAAGAGATAAAGTAATCAGAAGATTTAGGCCAGGGGATAAT
GCTTTAAAAGAAATGAGATAATATAATCAAACTCCATCTCTACTAATAAGAAAATTACCT
TTTTAGAGGCTAGTAAGGGAAATATCAACAAGATTGACAGAGGAAGATAACTTGAGATGG
ACAAGCTTTGCTTTAGTGTTATTATAAACTGTTGTTGAAGATTACATGGTATCATTTTAT
GAAGATGCAAATGCATGCGCTTTACATGCAAAAAGAGTTACCTTAATGACAAAAGATTTA
GCACTAGCTGCAAGAATTAGAGGATAAAAAAATGTT

rostrata
AAGAGGAGAAGTGTTTCTAATTAATCTCAATAAAGATCAAATAATGCAACA???AATAAT
TAAGTAAATAATTAAAGATCGAAATCTGCAGGT---GGATAAATGAAT---AAATAAAAA
AACCCA---TCTTTACAGTAAAATAAGCCTGTATCAACTAAGAAAAAGAGATAATCCTCT
GGTGAAAAATATGAATCAGCAAGAGATAAAGTAATTAGAAGATTTAGACCAGGAGATAAT
GCTTTGAAAGAACTTAAATAATATAATTAAAATCCATCACTATTTATTCGAAAACTACCT
TTTTAAAGGTTAGTGAGAGAAATATCAACACGATTGTCAGATTAGGATTAAATTAGATGG
ACAAGTATTGCTCTAATTCTATTATAAACCGTTACAGAAGATTATATGATATCATTTTAT
GAAGATGCGAATGCATGCGCTTTACATGCTAAAAGAGTTACCCTTATGAGCAAAGATATG
ATTCTTGCTGCTAGAATTAGAGGATAAAGGAATGTT

silvana
AAGAGGAGGAGTAATTCAAACTAAAATCAACAAAGATCTACGAGCACA---ACTAATAAT
TAATCGAATTAATAGAGATCAAAATCTACTGGA---AACCAAGGTAAT---AAATAGGTT
CCTGCAACAGCTTAGAAATAAAAAAAGCCAGATACCACAAAGAAAAAGAGATAATCATCT
GGTGAAAAATATGAGTCTACAAGAGATAAAGTTATTAGGAGATTTAGACCTGGTGATAAT
ACATTAAAGGAACTTCGATAATATAATTAAGCTCCAACTCTGATGATTAGGAAACTACCT
TTTTAGAAGTTAGTGAGAGAAATAGCATCTAGAATGACAGAATTAGAGCATTTGAGATGG
ACTAGTTAATCCTTGATGCTCTTACAATCAGTTACTGAGGATTATATGGTATCGTTTTAT
GAGGATGCTAACGCCTGT??????????????????????????????????????????
????????????????????????????????????

thermophila
AAAAGGAGGAGTAATTCAAATCAAAATTAATAGAGATCTGATAGCCTG---AAAAAAAAT
AAATAGGATAATTTGAGATCTAAATCTGCTGGGAAT??????GGGAATGAAAAA??????
??????---???????????????????????AGTACAAAAAAGAAAAGGGAGTCATCA
GGAGAAAAATATGAGTCTGCAAGAGATAAAGTTATTAGAAGATTTAGGCCAGGGGATAAT
GCCCTTAAGCAATTAAGATAATATAATTAGACTCCATCATTGCTAATAAGAAAATTACCC
TTTTAGAGGTTAATAAGAGAGATTTCTACAAGAATGACAGAAGAAGATTCGCTGAGATGG
ACAAGTTTTGCTCTGGTTTTACTTTAAACAGTTGTAGAAGATTATATGGTTTCATTTTTT
GAAGATGCGAATGCTTGTGCTCTCCATGCTAAAAGAGTTACTCTCATGTCTAAAGATCTT
GCTCTTGCGGCAAGAATTAGGGGATAAAAAAATGTT

tropicalis
AAAAGGAGAAGCAATTCTAACCAAAATTAATCAGCATCGAATAATGTT---TAAAATAAT
TAATCTGCAAATCAGAGATCAAAGTCAGCAGGA---CCATAAGGGAAT---AAATAAAAA
AATTAG---CCTCAATAATAAAAAAAGGCTGTCACC------AAAAAGAGATAGTCATCA
GGTGAAAAATATGAATCAGCAAGAGATAAAGTAATTAGAAGATTTAGGCCAGGGGATAAT
GCTTTAAAAGAAATGAGATAATATAATCAAACTCCATCTCTACTAATAAGAAAATTACCT
TTTTAGAGGCTAGTAAGGGAAATATCAACAAGATTGACAGAGGAAGATAACTTGAGATGG
ACAAGCTTTGCCTTAGTATTATTATAAACTGTTGTTGAAGATTACATGGTATCGTTTTAT
GAAGATGCAAATGCATGCGCTTTACATGCAAAAAGAGTTACCTTAATGACAAAAGATTTA
GCACTAGCTGCAAGAATTAGAGGATAAAAAAATGTT

;
end;

BEGIN TREES;
                    TREE tree = (borealis,canadensis,(rostrata,(mimbres,((furgasoni,(mobilis,tropicalis)),((elliotti,(malaccensis,thermophila)),(limacis,(leucophrys,silvana)))))));
END;
```

Trichoderma

```
#NEXUS
[ Title ]
begin taxa;
	dimensions ntax=6;
	taxlabels
		Trichoderma_asperellum
		Trichoderma_atroviride
		Trichoderma_harzianum
		Trichoderma_longibrachiatum
		Trichoderma_reesei
		Trichoderma_virens
;
end;
begin characters;
	dimensions nchar=465;
	format missing=? gap=- matchchar=. datatype=nucleotide;
	matrix

Trichoderma_asperellum
ATGGCT---------------------------------------------CCTCGCCAA
TCAACTTCAGGAACCCAACAACGCTAT---------------???GCCAGACCAAGCGAC
ATTCAGCCGGGCGATCCACTACCTACGAGACAGAAACGTCGATATCGCCCAGGCACCGTT
GCGCTACGAGAAATCAGGCAATATCAAGCCAATACAAAGCTTCTACTTCTAAAACTTCCG
TTTATGCGACTCGTCCGTGAAATTGGATTAAATTGTCGCCCGAGAGGAAAAGAATTTAGA
TGGCAGAGCCAGGCAATCCAGGCACTGCAAGAGGCTGCAGAAGCATTTATGGTACATCTA
TTTGAAGATGCACAGCTATGCGCGGTCCATGCGAAAAGAGTGACACTTATGCAAAAAGAC
ATTCAGCTGGCGAGGAGGATTCGAGGTATATGGGGCGGGCTTGGT

Trichoderma_atroviride
ATGGCT---------------------------------------------CCTCGCCAA
TCAACGGCAGCGCCCAAACAACGATAT---------------???GCCAGACCAAGCGAC
ATACAGCCGGGCGATCCACTGCCTACGAGACAGAAGCGGCGATATCGCCCGGGCACCGTT
GCGCTGCGAGAAATCAGGCAATATCAAGCCAATACAAAGCTTCTACTCCTAAAACTTCCA
TTTATGCGACTCGTTCGCGAAATTGGATTGAATTGTCGGCCGAGAGGGAAAGATTTTCGA
TGGCAGAGTCAGGCCATCCAGGCACTGCAAGAGGCCGCAGAAGCATTCATGGTACATCTT
TTTGAAGATGCGCAGCTATGCGCGGTCCATGCGAAGAGAGTGACACTTATGCAAAAAGAC
ATCCAGCTAGCGAGGAGGATTCGAGGTATATGGGGCGGGCTTGGT

Trichoderma_harzianum
ATGGCT---------------------------------------------CCTCGTCAA
AAAACTTCGGCG---------------GCCTCACAGAAATTGCGCTCCAGACCAAGCGAT
GTACAGCCGGGCGATCCATTACCTACAAGACAGAAGCGACGATATCGGCCGGGCACCGTT
GCGTTGCGAGAGATCAGGCAATACCAGTCAAATACAAAGCTTTTATTGTTAAAACTACCA
TTCATGCGCCTTGTTCGTGAAATTGGATTGAATTGTCGGCCGAGAGGGAAAGACTTCAGA
TGGCAGAGTCAGGCTATTCAGGCACTACAAGAGGCCGCGGAGGCCTTTATGGTACATCTT
TTTGAGGATGCGCAGCTATGCGCTGTCCACGCAAAGAGAGTTACACTTATGCAAAAAGAT
ATACAGCTTGCGAGGAGAATCCGAGGCATCTGGGGCGGCCTTGGT

Trichoderma_longibrachiatum
ATGCCT---------------------------------------------CCTCGTCAG
TCCACGGCGGGA---------------ACCTCACAGAAAGTGCGCTCCCGCCCGAGCGAT
GTACAGCCGGGCGATCCACTGCCTGCCAGACAGCGACGACGATACCGCCCGGGCACCGTC
GCATTGCGGGAAATCAGGCAATACCAGGCCAACACGAAGCTTTTGCTGCTGAAGCTGCCA
TTTATGCGCCTCGTTCGGGAGATTGGATTGAATTGCCGTCCAAGGGGGAAAGAGTTCAGG
TGGCAGAGCCAGGCCATCCAGGCGCTGCAAGAAGCGGCAGAGGCCTTCATGGTACATCTG
TTTGAGGATGCGCAGCTGTGCGCTGTGCACGCCAAGAGGGTTACCCTCATGCAGAAAGAC
ATACAGCTTGCGAGGAGGATTCGAGGCATCTGGGGCGGCCTCGGT

Trichoderma_reesei
ATGCCCTCACCAACCCCAAATCGTCTTCTGCTAACCCCCAACTCTTCAGCG---------
------------------------------------------------------------
---------GGCGACCCACTGCCTACGAGACAACGACGACGATACCGCCCCGGCACTGTC
GCATTGCGGGAAATCAGGCAATACCAGGCCAACACGAAGCTTTTGCTGCTGAAGCTGCCG
TTTATGCGCCTCGTTCGAGAAATTGGATTGAATTGTCGTCCGACGGGCAAAGAGTTTAGA
TGGCAGAGCCAGGCCATCCAGGCGCTGCAAGAAGCCGCAGAGGCCTTCATGGTACATTTG
TTTGAGGACGCGCAGCTGTGCGCTGTACACGCCAAGAGGGTCACCCTGATGCAGAAAGAC
ATACAGCTTGCAAGGAGAATCCGGGGCATCTGGGGCGGCCTTGGC

Trichoderma_virens
ATGGCT---------------------------------------------CCTCGTCAA
TCAACGTCAGGG---------------GCTTCACAAAAATTGCGTTCCAGACCAAGCGAT
GTACAGCCGGGCGATCCATTACCTACGAGACAGAAACGACGATATCGCCCAGGCACCGTT
GCGTTGCGAGAAATCAGGCAATACCAGTCAAATACGAAGCTGTTGCTACTAAAACTGCCA
TTCATGCGCCTTGTTCGTGAAATTGGATTGAATTGTCGCCCGAGAGGAAAAGATTTTAGA
TGGCAGAGCCAGGCCATTCAGGCGCTACAAGAGGCCGCGGAGGCATTCATGGTACATCTT
TTTGAGGATGCGCAGCTGTGCGCTGTCCACGCAAAGAGAGTTACCCTTATGCAAAAAGAC
ATACAACTGGCGAGGAGAATTCGAGGCATCTGGGGCGGCCTTGGT

;
end;

BEGIN TREES;
			TREE tree = (Trichoderma_asperellum,Trichoderma_atroviride,((Trichoderma_harzianum,Trichoderma_virens),(Trichoderma_longibrachiatum,Trichoderma_reesei)));
END;
```
