# Supplementary material for: CenH3 evolution reflects meiotic symmetry as predicted by the centromere drive model
Source: Sci Rep. 2016 Sep 15;6:33308. doi: 10.1038/srep33308 (PMC5024113; doi:10.1038/srep33308)
Supplement: Supplementary File S4 [file srep33308-s4.pdf]

CenH3 evolution reflects meiotic symmetry as predicted by the centromere drive model

František Zedek, Petr Bureš

Supplementary File S4

Phylogenetic tree, data and R script used for pgls analyses

Phylogenetic tree, dated based on TIMETREE divergence times of analyzed clades, used for the pgls analysis

####(((((Aspergillus:55,Penicillium:55):425,(Trichoderma:55,Colletotrichum:55):425):251,Saccharomyces:731):572,(Drosophila:847,(Primates:430,BonyFish:430):417):456):211,(Bryophyta:552,(Lycopodiophyta:432,(Ferns:405,((Asteraceae:121,(Brassicaceae:114,Fabaceae:114):7):34,Poaceae:155):250):27):120):962):211,(Plasmodium:1240,Tetrahymena:1240):485);

Data used for the pgls analysis; meiosis - A=asymmetric, S=symmetric; w = overal omega ratio; brcor = proportion of positively selected branches after correction for multiple testing; br = proportion of positively selected branches before correction for multiple testing; cod = proportion of positively selected codons####

##### clade	meiosis	w	brcor	br	cod
##### Asteraceae	A	0.187	0	0.272	0.013
##### BonyFish	A	0.126	0.053	0.158	0.013
##### Brassicaceae	A	0.433	0	0.132	0.028
##### Drosophila	A	0.268	0.069	0.172	0.024
##### Fabaceae	A	0.262	0.032	0.129	0.015
##### Poaceae	A	0.247	0	0.095	0.020
##### Primates	A	0.412	0.04	0.2	0.007
##### Tetrahymena	A	0.036	0.043	0.087	0.006
##### Bryophyta	S	0.103	0	0.059	0.014
##### Ferns	S	0.067	0	0	0
##### Lycopodiophyta	S	0.024	0	0	0
##### Aspergillus	S	0.062	0	0.121	0.006
##### Colletotrichum	S	0.041	0	0	0.007

##### Penicillium	S	0.056	0	0.053	0.003

##### Saccharomyces	S	0.05	0	0	0.003

##### Trichoderma	S	0.042	0	0.111	0.006

##### Plasmodium	S	0.043	0	0	0

R script used for pgls analyses in caper R package

```
library(caper) # calls caper R package

FF<-read.tree("TreeFile.nwk") # reads the phylogenetic tree provided above
plot(FF); axisPhylo(1)       # displays the phylogenetic tree
is.ultrametric(FF)  # checks whether the tree is ultrametric

dat<-read.table("DataFile.txt", ,sep="\t",header=T) # reads the data table provided above
data<-comparative.data(FF,dat[c(1:16),],names.col="clade",vcv=TRUE,warn.dropped=TRUE)  # combines the phylogeny with the dataset and ensures consistent structure and ordering for use in functions

m.w<-pgls(w~meiosis,data=data,lambda="ML")   # tests whether asymmetrics and symmetrics significantly differ in overal omega ratio
m.brcor<-pgls(brcor~meiosis,data=data,lambda="ML") # tests whether asymmetrics and symmetrics significantly differ in the proportion of positively selected branches (after correction for multiple testing)
```

m.br<-pgls(br~meiosis,data=data,lambda="ML") # tests whether asymmetrics and symmetrics significantly differ in the proportion of positively selected branches (prior correction for multiple testing)

m.cod<-pgls(cod~meiosis,data=data,lambda="ML") # tests whether asymmetrics and symmetrics significantly differ in the proportion of positively selected codons

summary(m.w) # summarizes the results for overall omega ratio

summary(m.brcor) # summarizes the results for the proportion of positively selected branches (after correction for multiple testing)

summary(m.br) # summarizes the results for the proportion of positively selected branches (prior correction for multiple testing)

summary(m.cod) # summarizes the results for the proportion of positively selected codons
